# Cross-species protein sequence and gene structure prediction with fine-tuned Webscipio 2.0 and Scipio

**DOI:** 10.1186/1756-0500-4-265

**Published:** 2011-07-28

**Authors:** Klas Hatje, Oliver Keller, Björn Hammesfahr, Holger Pillmann, Stephan Waack, Martin Kollmar

**Affiliations:** 1 Abteilung NMR basierte Strukturbiologie, Max-Planck-Institut für Biophysikalische Chemie, Am Fassberg 11, D-37077 Göttingen, Germany; 2 Institute of Computer Science, University of Göttingen, Goldschmidtstr. 7, 37077 Göttingen, Germany

## Abstract

**Background:**

Obtaining transcripts of homologs of closely related organisms and retrieving the reconstructed exon-intron patterns of the genes is a very important process during the analysis of the evolution of a protein family and the comparative analysis of the exon-intron structure of a certain gene from different species. Due to the ever-increasing speed of genome sequencing, the gap to genome annotation is growing. Thus, tools for the correct prediction and reconstruction of genes in related organisms become more and more important. The tool Scipio, which can also be used via the graphical interface WebScipio, performs significant hit processing of the output of the Blat program to account for sequencing errors, missing sequence, and fragmented genome assemblies. However, Scipio has so far been limited to high sequence similarity and unable to reconstruct short exons.

**Results:**

Scipio and WebScipio have fundamentally been extended to better reconstruct very short exons and intron splice sites and to be better suited for cross-species gene structure predictions. The Needleman-Wunsch algorithm has been implemented for the search for short parts of the query sequence that were not recognized by Blat. Those regions might either be short exons, divergent sequence at intron splice sites, or very divergent exons. We have shown the benefit and use of new parameters with several protein examples from completely different protein families in searches against species from several kingdoms of the eukaryotes. The performance of the new Scipio version has been tested in comparison with several similar tools.

**Conclusions:**

With the new version of Scipio very short exons, terminal and internal, of even just one amino acid can correctly be reconstructed. Scipio is also able to correctly predict almost all genes in cross-species searches even if the ancestors of the species separated more than 100 Myr ago and if the protein sequence identity is below 80%. For our test cases Scipio outperforms all other software tested. WebScipio has been restructured and provides easy access to the genome assemblies of about 640 eukaryotic species. Scipio and WebScipio are freely accessible at http://www.webscipio.org.

## Background

Whole genome sequences of eukaryotes are generated with increasing speed [1]. While the focus at the beginning of high-throughput DNA sequencing was on model organisms and the human genome, for which tremendous amounts of secondary data was available, the aims have shifted to organisms of medical or economic relevance (e.g. *Plasmodium falciparum *[2] or *Phytophthora ramorum *[3]), to the comparative analysis of entire taxa (e.g. the Drosophila clade [4] or Candida species [5]), and, very recently, to organisms of evolutionary interest (e.g. *Trichoplax adhaerens *[6] or *Volvox carteri *[7]). However, gene catalogues are only available for a small part of the sequenced organisms and a precise and complete set of genes is still unavailable for even a single species. In the first instance the gene annotation is done with automatic gene prediction programs that either predict only isolated exons, or reconstruct the complete exon-intron structures of the protein-coding genes, or even try to predict 5' and 3' untranslated regions. *Ab-initio *gene prediction programs only use the assembled DNA sequences as input, having precomputed models for nucleotide distributions, while evidence-based programs consider alignments of ESTs, cDNAs, or annotated sequences from closely related organisms, with the target sequence (reviewed in [8]). The highest accuracy is reached by programs that combine model-based and alignment-based approaches [9,10].

For many biological applications like the phylogenetic analysis of a protein family (e.g. [11]) or the comparative analysis of the exon-intron structure of a certain gene from different species (e.g. [12]), it is necessary to obtain translated transcripts of homologs of closely related organisms or the reconstructed exon-intron patterns of the genes, respectively. The protein sequences of homologs of a certain protein can be obtained in several ways. Annotations based on *ab-initio *gene predictions, sometimes supplemented by EST data, are available for about half of the sequenced eukaryotic genomes, although it is often tedious to find the corresponding data via the FTP-pages of the sequencing centers. In addition, automatic predictions are not complete and in many cases not correct. For very few eukaryotes, full-length cDNA data can be accessed. However, these data never cover the complete transcriptome of the species. Another possibility is to manually annotate the protein homologs in the genomes of choice by comparative genomics. This is certainly the most accurate way. By this approach a multiple sequence alignment of as many as possible homologs is created, and based on this sequence alignment mispredicted sequence regions (insertions and missing regions) are easily detected. Further homologs are added by manual inspection of the corresponding genomic DNA regions and manual reconstruction of intron splice sites. Splice sites are in most cases conserved throughout the eukaryotes [13] and therefore their position and frame can be used for gene reconstructions by comparing gene structures from known and to be annotated genes.

To assist in the task of the manual annotation of eukaryotic genomes, and to provide options for genomes for which gene prediction data is not available, we have recently developed Scipio [14,15]. Scipio is a post-processing script for the Blat output [16] and maps a protein sequence to a genomic DNA sequence. Blat has been developed for the fast alignment of very similar DNA or protein sequences. However, Blat is not able to identify very short exons (two or three amino acids, or exons of just the N-terminal methionine), it is not able to assemble genes spread on more than one contiguous DNA sequence, it misses exons that are too divergent, it does not apply biological sequence models to determine exact splice site locations on nucleotide level, or to distinguish introns from insertions caused by frameshifts or in-frame stop codons [14,15,17]. Scipio is able to address most of these issues resulting in considerably improved gene structure reconstructions [14,15]. Its initial intention was to cope with sequencing errors, to assemble genes from highly fragmented genome assemblies, and to reconstruct intron splice sites. Scipio was not able to correctly reconstruct very short exons or to correctly reconstruct genes in cross-species searches if these were not highly identical.

Here, we present the fundamentally improved version of the Scipio software that has been extended for the use in cross-species searches. In addition, very short exons and divergent regions at intron borders are now correctly reconstructed. Scipio can be used via the web-interface WebScipio that provides access to 2111 genome assembly files for 592 species (end of February 2011).

## Methods

The presented software consists of two programs that form a pipeline for the output of the external program Blat, which is executed first. The Blat results are post-processed by the Scipio script written in Perl [18]. WebScipio provides a graphical user interface for Scipio that we have developed using the web framework Ruby on Rails [19,20]. The workflow was optimized to direct the user to the necessary input parameters. This was implemented with the technique of Asynchronous Javascript and XML (AJAX). Visual effects were realized with the help of Prototype [21] and script.aculo.us [22] that are JavaScript libraries, which are integral parts of Ruby on Rails.

### Scipio

The Scipio Perl script itself, which can also be run standalone, has undergone numerous extensions that are based on our extensive experience in manual gene annotation [11,23,24]. The general setup of the script that aimed to handle all the various sequencing and assembly errors has already been described [14]; here, we present an implementation of the Needleman-Wunsch algorithm which is the main extension to the previous version.

### The Needleman-Wunsch Algorithm used in Scipio

In the updated version of Scipio, we use a modified Needleman-Wunsch style dynamic programming (DP) algorithm to perform an exhaustive search for the best-scoring spliced alignment between the query and target sequence fragments that were left unmatched by Blat. Like the original Needleman-Wunsch algorithm, it calculates an optimal global alignment between the sequences, but it is adjusted to find an optimal *spliced *alignment between a protein query sequence *s *and a genomic target sequence *t*. Given the computational cost of |*s*| and |*t*|, it is executed only on very short sequence fragments *s *and *t*. We introduce different categories of penalties depending on the type of matching. Any alignment can be represented by a *parse *Φ: a collection of pairs of strings (*s*_1_, *t*_1_), ..., (*s*_*k*_, *t*_*k*_), such that the aligned sequences are the concatenations: *s = s*_1_... *s*_*r*_, *t = t*_1_... *t*_*r*_. A *penalty score p*(*s*_*k*_, *t*_*k*_) is assigned to each pair as follows:

• if *s*_*k *_is a single residue and *t*_*k *_a string of length 3 (codon), then *p*(*s*_*k*_, *t*_*k*_) = *p*_MAP_(*s*_*k*_, *t*_*k*_) is a *match/mismatch *penalty:

• an *insertion *penalty *p*(*s*_*k*_, *t*_*k*_) = *p*_INS _is assigned to them if *s*_*k *_is a single residue and *t*_*k *_is empty

• a *gap *penalty *p*(*s*_*k*_, *t*_*k*_) = *p*_GAP _is assigned to them if *t*_*k *_is a codon and *s*_*k *_is empty

• a *frameshift *penalty *p*(*s*_*k*_, *t*_*k*_) = *p*_FS _is assigned to them if *t*_*k *_consists of 1 or 2 nucleotides, and *s*_*k *_is empty or a single residue

To cover the case of introns, in addition we define *intron *penalties based on the donor and acceptor splice sites:

with a constant value *p*_INT _for any sequence of nucleotides *n*_1_...*n*_*ℓ *_and zero splice site penalties if *n*_1_*n*_2 _= "GT", and *n*_*ℓ-1*_*n*_*ℓ*_ = "AG". We distinguish two cases: in-frame introns, and introns splitting codons:

• if *s*_*k *_is empty and *t*_*k *_exceeds the minimum intron length, then *p*(*s*_*k*_, *t*_*k*_) = *p*_INTRON_(*t*_*k*_) is the intron *penalty*

• if *s*_*k *_is a single residue *n*_1_*n*_2_*n*_3_, and *t*_*k *_= *n*_1_ω*n*_2_*n*_3_, or *t*_*k *_= *n*_1_*n*_2_ω*n*_3 _with single residues and ω a string exceeding the minimum intron length, then the penalty is a combined match/intron penalty: *p*(*s*_*k*_, *t*_*k*_) = *p*_INTRON_(ω) + *p*_MAP_(*s*_*k*_, *n*_1_*n*_2_*n*_3_). Here, two different penalties are defined (depending on the frame of the intron), and thus the minimum of them is taken.

If (*s*_*k*_, *t*_*k*_) does not satisfy any of these conditions, no penalty is defined resulting in an invalid parse. By combining insertions, deletions, and frameshifts, there is always some valid parse for any given pair of sequences. The cost of a parse Φ is the sum of the penalties: *p*(Φ) = *p*(*s*_1_, *t*_1_) + ... + *p*(*s*_*r*_, *t*_*r*_), and we calculate

by computing the DP matrix (*M*_*ij*_) containing the minimal score for an alignment of the subsequences *s*_[0..*j*-1] _and *t*_[0..*i*-1]_, using the following recursions:

where each of these expressions corresponds to one of the possible penalty types for the last segment of the parse.

The last three lines cover introns, one for each reading frame, with *ℓ*_min _denoting the minimum intron length. To avoid having to iterate over all values for *i' *in these cases, we precompute nine variants of the score matrix with partial intron penalties added (indexed by a nucleotide *n *if it splits a codon) as follows:

Note that *n *denotes the nucleotide before the intron in *M*^(1)^, and the nucleotide after it in *M*^(2)^. The latter contains already the mismatch penalty, while the former does not. With *i' *the latest segment start allowed (*i' *= *i *- *ℓ*_min _for an intron scored by *M*^(0)^, and *i' *= *i *- *ℓ*_min_-3 for a codon split by an intron), the intron variables are given recursively by

and then replace the last three lines in the recursion for *M*_*ij*_:

The penalties for the Needleman-Wunsch algorithm can be adjusted manually in the Scipio command-line version but not via the WebScipio web-interface. The penalties need to be well balanced so that the Needleman-Wunsch search does not result for example in a number of artificial short exons where a long exon is missing due to a gap in the genome assembly. Based on extensive tests with in-house test data we set the following values as default: mismatch-penalty: 1.0; insertion-penalty: 1.5; gap-penalty: 1.1; frameshift-penalty: 2.5; intron-penalty: 2.0 + the respective penalties for donor and acceptor splice sites.

### WebScipio

At present, the web interface offers 2272 genome files of 643 eukaryotic organisms. Metadata corresponding to the species, like assembly versions, sequencing centers, and assembly coverage, is available from the diArk database [25]. WebScipio reads the metadata out of a periodically updated text file generated from diArk, or queries the diArk database directly with SQL.

The gene structure schemes resulting from the Scipio run are generated and displayed in the Scalable Vector Graphics (SVG) format [26]. This allows scaling the graphics while retaining their resolution and to show tooltips generated with JavaScript and HTML for each element of the gene structure schemes. For browsers not supporting SVG, a fallback solution is implemented, which uses the Portable Network Graphics (PNG) format. The PNG files are generated by Inkscape [27].

Internally, the sequence data is processed with the help of BioRuby [28]. Results are saved in the YAML format [29], but are also available for download in the GFF format. The web application runs the Blat and Scipio jobs in the background, which was implemented using the Rails plug-in Workling in combination with Spawn [30,31]. The server-side stored session data is increasing with every extension of WebScipio. To make the session storage fast, flexible, and scalable we use a database backend called Tokyo Cabinet [32]. It offers a simple key-value store, also called hash store, for accessing different data objects with the help of a unique key for each object. Tokyo Tyrant is the network interface to Tokyo Cabinet and allows storing data across the network on several servers. It is used in WebScipio for scalability reasons.

### External Tools

We use Hoptoad for error reporting [33]. It is a web application that collects errors generated by WebScipio, aggregates them to the detailed error reports for developer review, and sends email notifications. We use a behaviour-driven testing strategy to validate the functionality and behaviour of WebScipio. For the automation of these tests we use RSpec [34], which is a behaviour-driven development framework for the Ruby programming language. Our intention for this test implementation was the need of reliability and accuracy within the continuously extended software. Application tests are run with Selenium, a test system for web applications [35]. This offers the opportunity to test the web-interface as a whole. Selenium integrates into the Mozilla Firefox browser as a plug-in that records the user interaction in the form of a Ruby script. To run the test scripts without user-interaction, Selenium starts and controls the browser automatically. We integrated the user-interface tests into our automated test environment as additional RSpec test cases.

## Results and Discussion

### Scipio and WebScipio workflow, and general parameters for fine-tuning gene predictions

**Figure 1:**
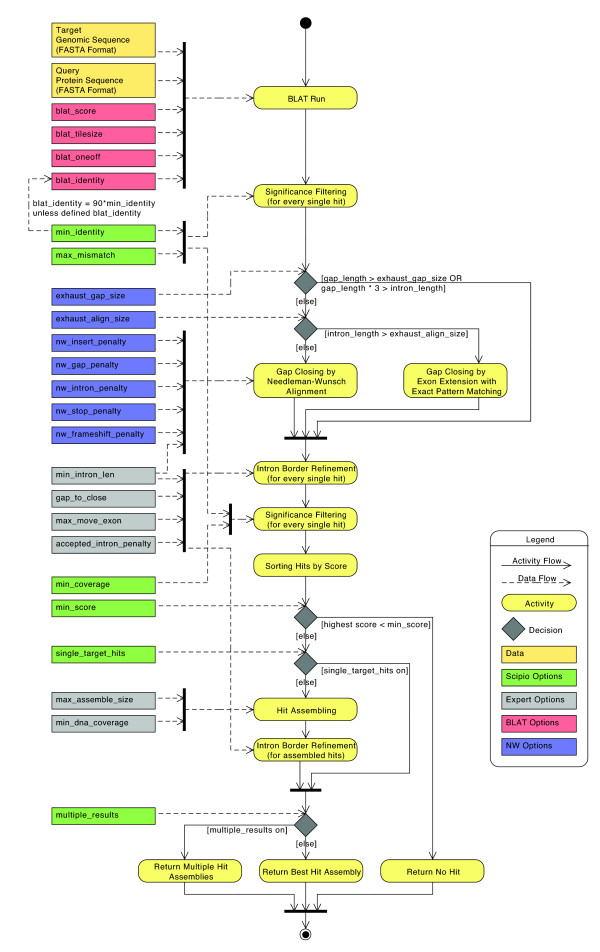
**The extended Scipio workflow**. This diagram depicts the activity and data flow of a Scipio run. Scipio needs a protein and a target genome sequence, both in FASTA format, as input to start a Blat run. Every single Blat hit is subsequently processed and filtered, and assembled in the case of hits on multiple targets. The gap_length describes the number of amino acids of an unmatched query subsequence. The intron_length is the corresponding length of the unmatched target subsequence in nucleotides.

The general workflow of Scipio and WebScipio is similar to that described previously [14,15]. Scipio provides some general search parameters that filter the Blat output for further post-processing, and offers several expert options that influence the post-processing steps. In the new Scipio version, especially the part of the gap-closing (mapping the parts of the query sequence to the target sequence that Blat failed to recognize) and hit extension (modelling the regions at exon borders, including terminal exons, where homology was too low to be identified by Blat) has been improved (Figure 1, see also Additional file 1). This has been done by implementing the Needleman-Wunsch algorithm for the search of unmapped query sequence in respective target regions and by introducing parameters that allow a higher divergence from the exon border regions predicted by Blat. All new parameters are adjustable by the user although the default values should be good enough for most cases. However, especially when searching for very divergent homologs or when searching for homologs of very divergent species, these parameters might need manual adaptation. Figure 1 shows a detailed scheme of the Scipio workflow including all parameters that can manually be adjusted. Also, some of the most important decisions are outlined that Scipio makes to provide the best possible result. The detailed scheme should allow the experienced user to fine-tune the search in especially difficult cases. The rationale for implementing each of the parameters and its consequences are explained below.

### The new web-interface

Because we wanted to offer most of the new parameters to the experienced user via the web-interface WebScipio, and we planned to introduce searches for alternatively spliced exons, we had to redesign the WebScipio workflow. The goal was to keep it well structured, intuitive and clear. We have also improved the usability for new and less experienced users by providing more examples, help pages, and documentation. The general design of selecting one target sequence for the search for multiple query sequences has been retained. Next, the experienced user can adjust many of the Scipio variables, and, also at this stage, many of the parameters for searches for alternative exons (those parameters are described elsewhere). We provide some default values for cross-species searches that are based on our experience in working with and knowledge about eukaryotic genomes [11]. For example, some genomes are known to contain only small numbers of introns while others are known to contain only short introns. Special settings for cross-species searches are provided for several specific taxa but the default cross-species parameters should be applicable for most genomes. Having selected a specific set of parameters every single parameter can still be adjusted individually.

As before, the most important result view is the scheme of the exon-intron structure of the search result. In this scheme, all information regarding the quality of the result (complete versus incomplete, containing gaps, i.e. unmatched parts of the query sequence, questionable introns, mismatches, frame-shifts, in-frame stop-codons, etc.) is included. Opening the "Search details" box provides further information concerning the search parameters, and additional data regarding the aligned query sequence is available from the different result views.

**Figure 2:**
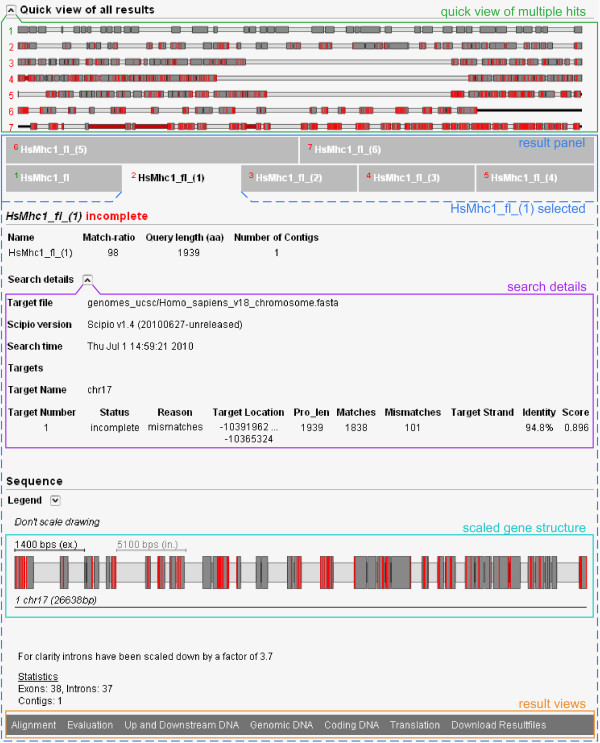
**Screenshot of the multiple results view of WebScipio**. The screenshot shows the result of the search for multiple homologs of one of the muscle class-II myosins from human in the human genome. The search parameters were --min_identity = 60%, --max_mismatch = ∞, and --multiple_results = yes to get as many homologs as possible. On top, the opened quick view of all reconstructed gene structures is shown. Next, a panel with the different results is shown. Green numbers mark complete results (100% of the query sequence reconstructed) while red numbers mark incomplete results (might contain gaps, mismatches, frameshifts, etc.). Result hit number 2 was selected and shows the result for the closest homolog to the query sequence with no gaps (unmapped query sequence) but 101 mismatches.

Due to gene and whole genome duplications during eukaryotic evolution there are often two or more closely related homologs of a certain protein per genome. This might cause some problems for cross-species searches if the paralogs in the target genome are about equally homologous to the query sequence. Therefore, we implemented a --multiple_results parameter. Switching --multiple_results off is the best way to get the exact gene structure for an intra-species search. Switching --multiple_results on (default setting in cross-species searches) allows retrieving all possible results depending on the general search parameters (like --min_score or --min_identity, Figure 2). If multiple hits are found they will be listed separately and can be analysed using the various result views. In addition, we implemented a quick view showing the gene structure schemes as a fast overview. As example for the benefit and limitation of this parameter, we searched for class-II myosin heavy chain homologs in humans (Figure 2). It is known that vertebrate genomes contain several muscle myosin heavy chain genes (belonging to the class-II myosin heavy chains) that are specialised for certain tissues like heart muscles or skeletal muscles [11]. Six of these genes are encoded in a cluster [36]. The example search shows the gene structure corresponding to the query sequence (*Hs*Mhc1_fl) and the gene structures of six homologs of varying degree of divergence. While the closest homolog (*Hs*Mhc1_fl_(1)) only contains mismatches compared to the query sequence, the three next closest homologs have severely deviating gene structures. They contain very long introns in the middle of the genes indicating that they are mixed genes assembled from the N-terminal half from one gene of the muscle myosin heavy chain cluster and the C-terminal half taken from the following gene of the cluster. The next two homologs are already very divergent so that parts of the genes cannot be reconstructed leaving many and long gaps.

### Use of WebScipio to produce publication-quality figures of gene structures

WebScipio can be used to easily produce publication-quality figures of gene structures. Either, these figures can be produced in the described way, or the user can upload an own genomic DNA sequence for use as target sequence. This is interesting in the case that the whole genome sequence is not known but only the genomic sequence of a certain region. SVGs can be downloaded and further processed in many graphics programs.

### New general and expert search parameters

The parameters --min_score (previously: --best_size), --min_identity, and --max_mismatch have already been described [15] and define the threshold for the Blat hits to be processed by Scipio. To reduce or even abolish the artificial assembly of contigs that by chance contain some identical residues we have introduced the parameter --min_coverage that applies to every single Blat hit. The coverage is the number of mapped residues (as match or mismatch) divided by the query length of the (possibly partial) hit. By default, Scipio rejects hits with coverage of less then 60%.

In addition to these general parameters we have introduced several expert options most of which will be described in detail below. One of the parameters is --transtable that allows the user to specify a non-standard translation table, for the use with species like Candida species, *Tetrahymena thermophila *and others that would otherwise lead to mismatches. Another parameter called --accepted_intron_penalty is used to define valid splice sites. By default, GT---AG and GC---AG are accepted, whereas, for example, introns with the pattern AT---AC would be classified as doubtful ("intron?"). By adjusting the --accepted_intron_penalty parameter those introns will also be accepted instead of defining those introns as "intron?".

### Parameters to account for additional/missing bases in predicted exons

Gene homologs even from very closely related species are often too divergent to be completely identified by Blat. While the core building block of the proteins and the functional sites are often strongly conserved, low homology is especially found at the surface of the proteins. Thus, loop regions are often sites of amino acid substitutions, insertions of long stretches of residues, and deletions. In addition, since the terminal regions of most proteins are at the surface, they are also often very divergent. Short stretches of nucleotides whose lengths are multiples of three and whose translations do not result in any in-frame stop codons are most likely to be insertions rather than true introns.

A parameter --min_intron_len has been implemented to distinguish introns from insertions, with a default minimum intron length of 22 nucleotides. A minimum intron length of 22 nucleotides is a rather conservative estimate given the minimum intron length of 35-40 nucleotides based on a test set of about 17,000 introns of genes of 10 model organisms [37]. Thus, by default additional coding sequence for up to seven amino acids (= 21 nucleotides) will be treated as exon sequence and joined with the surrounding exons into a single exon.

**Figure 3:**
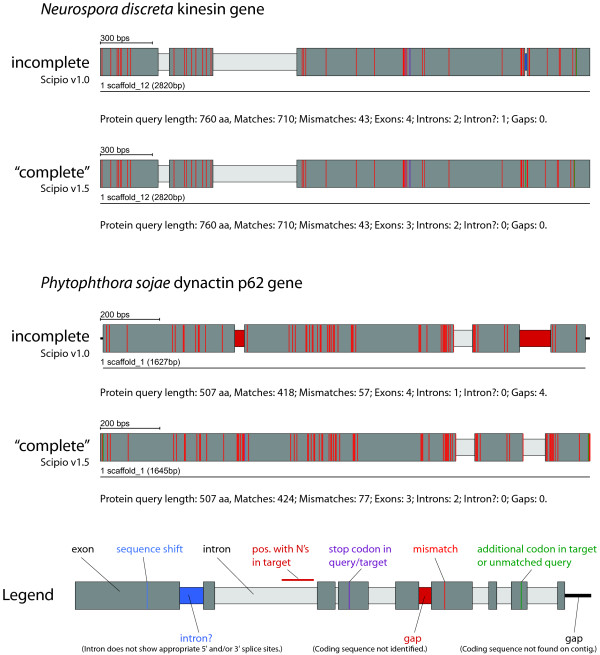
**Modelling of additional/missing bases in gene predictions**. Case A shows the result of the search of a kinesin from *Neurospora crassa *(query sequence) in *Neusrospora discreta *(target sequence) using the old and the new Scipio version. The --min_intron_len parameter has been set to 22. Case B shows the result of a search of the dynactin p62 homolog from *Phytophthora ramorum *(query sequence) in *Phytophthora sojae *(target sequence). To get the correct gene prediction the following Scipio parameters have been used: --min_identity = 60%, --min_score = 0.3, --max_mismatch = ∞, --gap_to_close = 15, --min_intron_length = 22. The colour coding is explained in the legend and applies to all gene structure figures. For further information see Additional file 2.

The opposite case of extra amino acids in the query is dealt with by the parameter --gap_to_close. By default, a mapping of up to six additional amino acids from the query sequence to the exon borders will be enforced at the cost of further mismatches, in order to eliminate a gap (of unmatched query sequence). This parameter also effects the modelling of the intron borders (see below). Figure 3 shows two examples of cross-species searches in which the target sequence contains additional or less amino acids in conserved exons. Case A shows the results of a search for a kinesin homolog from *Neurospora crassa *(query sequence) in the closely related organism *Neurospora discreta *(target sequence, see also Additional file 2). Because of the relatively high homology of the two sequences, Blat has already retained the additional residues of the query sequence so that they are included in the result of the old Scipio version. However, a questionable intron (called intron? in Scipio) was introduced in the region that contained additional nucleotides in the target sequence leading to missing residues in the target translation. With the new parameter --min_intron_len these additional nucleotides are correctly treated as exonic sequence. Case B shows an example of two divergent homologs of the dynactin p62 gene of *Phytophthora ramorum *(query sequence) and *Phytophthora sojae *(target sequence, see also Additional file 2). These two homologs contain a long divergent region with many consecutive mismatches in the first exon that is not identified by Blat and introduces a long gap of unmatched residues. In addition, the N- and C-termini have divergent sequences and different lengths. With the new parameters, Scipio can correctly model the target gene.

### Parameters to identify divergent exons and very short exons ignored by Blat

To identify exons that contain too many mismatches to be identified by Blat, and to correctly annotate very short exons, the Needleman-Wunsch algorithm described above forces an alignment of unmatched query sequence to spare target sequence. Very short exons of one to four amino acids are only reconstructed if they are identical to the query sequence and contain valid splice sites while short exons of five to seven amino acids are also often correctly reconstructed if they contain mismatches between query and target sequence (e.g. in cross-species searches). The maximal lengths of query and target sequence fragments to be aligned with Needleman-Wunsch are controlled by the parameters --exhaust_align_size and --exhaust_gap_size, respectively. By default, the exhaustive search is restricted to query gaps of 21 amino acides (three times the default Blat tilesize), since we expect Blat to successfully discover at least parts of any longer exons, and to a target subsequence of 15,000 bps. The restriction of the latter value is caused by the exponentially increased run time with increased target subsequence so that for example the potentially very long introns in mammalian genomes are only searched after manual increase of this value. Other parameters affecting the Needleman-Wunsch algorithm, such as the penalties mentioned above, can be adjusted by the command line version only, and not via WebScipio. However, the default values have extensively been tested with in-house data and should not require changes in most if not all cases.

**Figure 4:**
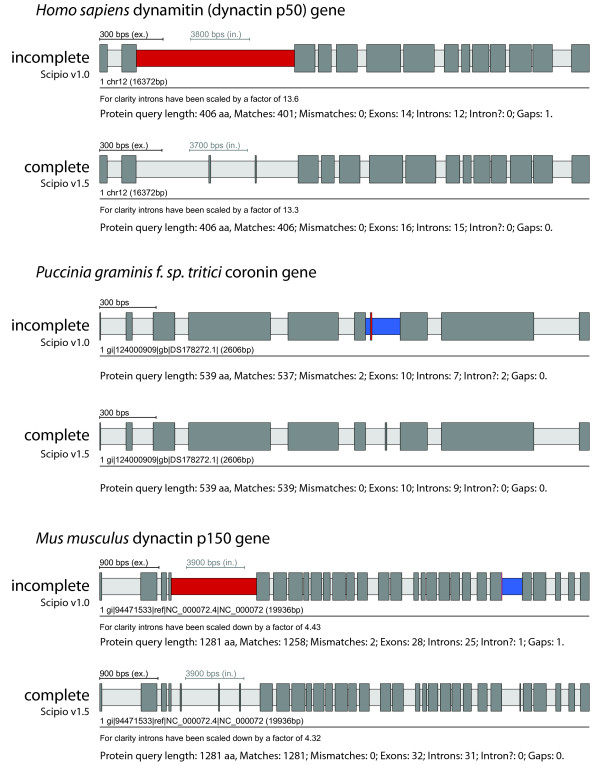
**Reconstruction of very short exons**. Case A shows the result for the reconstruction of the human dynamitin (dynactin p50) gene, that contains a 3 amino acid exon and a following 2 amino acid exon that are differentially included in the final transcript. These exons could not be reconstructed with Blat and the old Scipio version, but using the new Scipio version that enables Needlman-Wunsch searches. The --exhaust_align_size parameter has been set to 15,000 bp because of the length of the intron. Case B shows the result of the reconstruction of the coronin gene from *Puccinia graminis f. sp. tritici*. The small but evolutionarily conserved exon 7 can now correctly be reconstructed. Case C shows the result of the reconstruction of the mouse dynactin p150 gene that contains three short exons of 7, 6, and 7 amino acids close to the 5'-end of the gene. For the correct reconstruction, the --exhaust_align_size parameter has been increased to 10,000 bp, because of the length of the intron, and the --exhaust_gap_size has been set to 21 because of the length of the query that could not be mapped. The colour coding of the scheme is the same as in Figure 3. For further information see Additional file 2.

The effect of the new parameters on the search results is demonstrated with the examples shown Figure 4 (see also Additional file 2). In case A, the human dynactin p50 gene contains two very short exons of 3 and 2 amino acids. These two short exons are conserved in all vertebrates (B. Hammesfahr and M. Kollmar, unpublished data). Case B shows the coronin gene from the basidiomycote fungi *Puccinia graminis *encoding a short 3 amino acid exon (Figure 4, see also Additional file 2). In addition, the codons at the exon/intron junctions of this short exon are split. In most of the other basidiomycotes sequenced so far, this short exon is part of one of the neighbouring exons, or part of a longer exon that includes both neighbouring exons. However, it also exists in the basidiomycote *Melampsora laricis-populina*. Thus, this short exon is not an artificial creation but a true exon. Case C presents the dynactin p150 gene that contains three short exons of 7, 6, and 7 amino acids at the beginning of the gene (Figure 4, see also Additional file 2). Even with the Blat-tilesize set to 5 those exons are not recognized in the search against the chromosome assembly. This example best demonstrates the effect of the --exhaust_align_size (default setting 15,000 bps) and the --exhaust_gap_size (default setting 21 aa) parameters to completely reconstruct the respective part of the gene. At the 3'-end of the p150 gene, there is another very short exon that shows some homology to the beginning of the preceding intron and is therefore added to the 3'-end of the preceding exon although this results in some mismatches. This behaviour has also been corrected in the new Scipio version by some other parameters (see below).

Genes might not only contain very short exons between other exons but also at gene termini. Scipio uses an exact pattern search for N-terminal and C-terminal exons. Terminal exons will only be accepted if they match the query sequence and if the resulting intron borders agree with the two most common splice site patterns (GT---AG and GC---AG). The length of the terminal exons searched for is limited by the --gap_to_close parameter that is by default six residues.

### Parameters to account for low homology at intron borders

**Figure 5:**
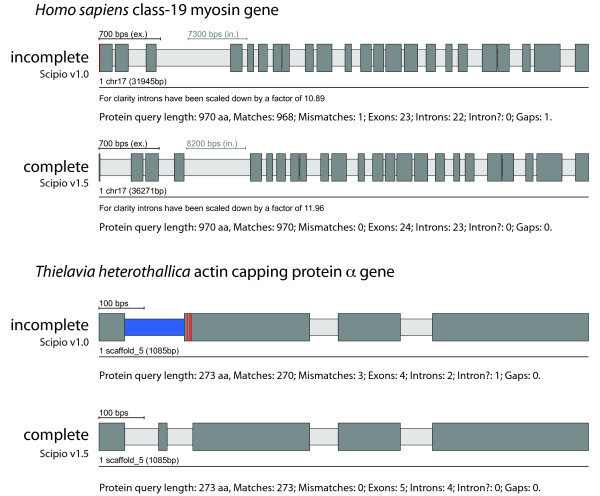
**Reconstructing short exons at low homology intron borders**. The scheme shows two examples for the reconstruction of short exons in regions where the intron borders of the neighbouring exons show some homology to the unmatched query sequence. The value for the --max_move_exon parameter has been set to 3 (case A) and 6 (case B), respectively. The colour coding of the scheme is the same as in Figure 3. For further information see Additional file 2.

The correct prediction of exact intron borders is one of the most difficult tasks in protein-based gene-prediction, especially those intron borders next to small exons, because their residues might be falsely assigned to neighbouring exons, or when homology is low, as in cross-species applications. Here, divergent residues at intron borders are often not recognized by Blat, or conversely, intronic sequence is falsely assigned to the exon. To deal with the latter case, Scipio cuts off the marginal parts of Blat matches and realigns them. The parameter --max_move_exon allows increasing the default value of six residues that are cut off from the marginal parts. Figure 5 shows the effect of this parameter in some representative examples (see also Additional file 2). In the case of the human class-19 myosin gene, Blat and the old Scipio version were not able to reconstruct the 5'-end of the gene correctly, because the intron in front of the second exon of the gene ends with the translated sequence LFQ that is very homologous to the real sequence LQQ. Blat added these residues to exon 2 albeit introducing a mismatch. With the new parameter --max_move_exon (default setting is 6), Scipio is now able to resolve this misalignment and to subsequently identify the correct exon 1. Case B shows the reconstruction of the actin capping protein α from *Theileria heterothallica *(Figure 5, see also Additional file 2). Here, by chance the intergenic region before exon 3 shows some homology to exon 2 (3 matches and 3 mismatches) and thus the exon 2 sequence was erroneously joined to exon 3. This happened irrespectively of lowering the Blat tilesize or adjusting any of the other Scipio parameters. By setting the --max_move_exon to 6 (default setting), the new version of Scipio is now able to correctly reconstruct the CAPα gene.

### Parameters to adjust searches on chromosomes or highly fragmented data

**Figure 6:**
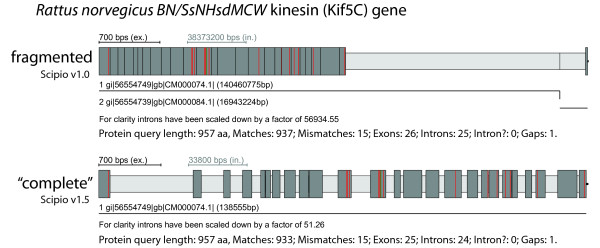
**Reconstructing genes on chromosome assemblies**. The scheme shows an example of the search for the rat homolog (target sequence) of the human Kif5C kinesin motor protein. The C-terminal about 25 amino acids of the rat Kif5C homolog are missing in the respective chromosome assembly. Using Scipio v1.0 a very short identical stretch of four amino acids, found on a different chromosome, has artificially been added to the 3'-end of the gene generating an "intron" of millions of base pairs (Note the scale of the introns!). The new parameter --single_target_hits now prevents this mis-assembly. The colour coding of the scheme is the same as in Figure 3.

Scipio is able to reconstruct genes that are spread on several contigs or supercontigs of highly fragmented genomes. As we have shown, this feature is one of the most important strengths of Scipio [14] that other programs do not offer. However, this feature is not needed in chromosomal assemblies, and might lead, especially in the case of cross-species searches, to composed hits that stretch across multiple chromosomes, one of them being false positive (Figure 6). Hence, it can be switched off with the parameter --single_target_hits (or --chromosome), which is the default setting when selecting a chromosome assembly as genome target file in WebScipio.

For highly fragmented genomes it is still useful to allow gene reconstructions across several contigs. But also in this case one would want to exclude the assembly of hits that would introduce extremely long introns between exons on different contigs. To accomplish for those cases we have introduced the --max_assemble_size parameter that adjusts the maximum size of intron parts at target boundaries. If an intron would have to be created between two partial hits across two contigs that exceeds the given size (default: 75000 nucleotides), the two hits will not appear together as parts of one composed hit; rather, the lower-scoring contig will be discarded unless --multiple_results is enabled. Alternatively, the parameter --min_dna_coverage can be used to limit the length of introns stretching across contig boundaries, by specifying a minimum query/target length ratio for composed hits, in percent.

### Improved gene structure reconstruction in cross-species searches

To test the sensitivity and specificity of the new Scipio version we performed a cross-species search of the dynein heavy chain (DHC) genes of *Homo sapiens *in *Loxodonta africana*. The dynein heavy chain genes have been chosen because they belong to the longest genes in eukaryotic genomes and thus contain many exons spread on several hundred thousands of base pairs (Table 1). In addition, the dynein heavy chain family members show different degrees of identity in mammals and are therefore very suitable to test the limits of Scipio. Afrotheria (to which the elephants belong) and the Euarchontoglires separated about 100 million years ago [38]. The DHC query sequence test set and the longer time the species have split up should be a better test for the cross-species search capabilities of Scipio compared to the cross-species search of human myosin heavy chain genes in the mouse genome that we performed earlier [15].

**Table 1:** Details of the dynein heavy chain genes used for the cross-species search

Protein	*Homo*	*Loxodonta*	status*	*Loxodonta*	*Loxodonta*
Name	Length [aa]	Length [aa]		Length [bp]	Exons
DHC1	4646	4561	P	62248	77
DHC2	4307	4234	P	413085	89
DHC3A	4707	4690	✓	358204	92
DHC3B	4624	4582	P	298827	78
DHC4A	4507	4508	✓	361115	82
DHC4B	4462	4428	P	115251	79
DHC4C	4486	4339	P	486267	69
DHC5	4589	4584	✓	140290	79
DHC6	4509	4457	P	134640	86
DHC7A	4024	4019	✓	253605	62
DHC7B	4070	3966	P	187156	60
DHC7C	3960	3960	✓	201577	73
DHC8	4265	4064	F	80895	73
DHC9A	4158	4062	P	302568	75
DHC9B	4612	4597	P	369531	85
DHC11	4779	4779	✓	81205	43

* Status: P = partial sequence (short part of the sequence missing); F = sequence fragment (large region of the gene missing in the genome); ✓ = sequence complete.

**Figure 7:**
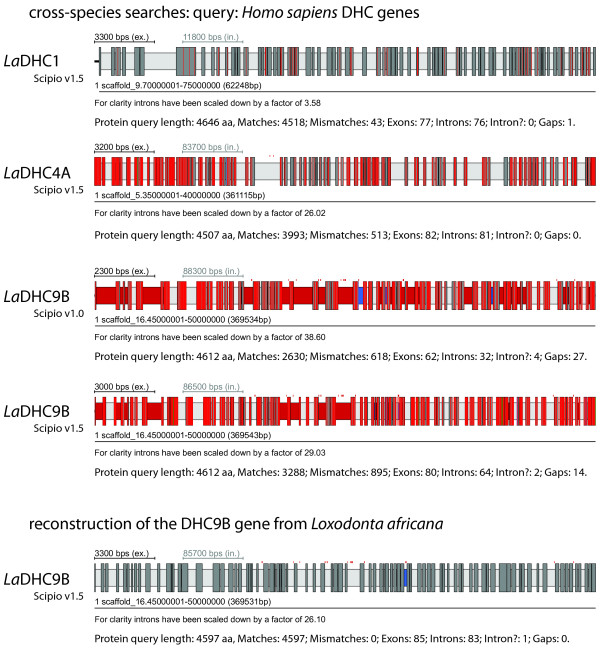
**Example cross-species searches**. The results of four searches with dynein heavy chain sequences from *Homo sapiens *in the elephant (*Loxodonta africana*) genome are shown. All genes are spread on several hundred thousands of base pairs. Statistics to the sequence results are given below the gene structure cartoons. An "intron?" is an intron for which the borders do not correspond to the standard splice sites GT---AG or GC---AG. The colour coding of the scheme is the same as in Figure 3.

**Figure 8:**
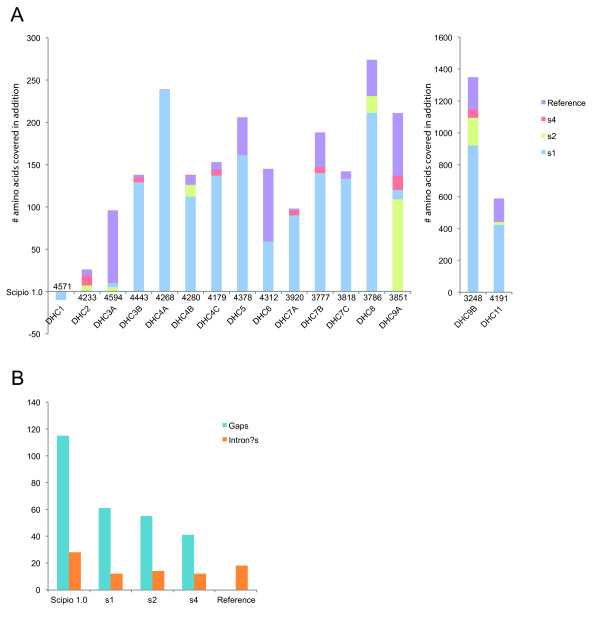
**Diagrams of the improvements introduced with the new Scipio version**. The diagrams describe the improvement of the gene reconstructions of the DHC genes in the cross-species search of the human homologs (query sequences) in elephant (target sequence) using different Scipio versions and parameters. (A) The base-line is the result of the search using the old Scipio v1.0. The maximal possible annotation is represented by the gene reconstructions based on the manually annotated elephant DHC genes (reference dataset, purple). The blue bars show the reconstruction with Scipio v1.5 using --blat_tilesize = 7, --exhaust_align_size = 500 and --exhaust_gap_size = 21 (dataset s1). Green bars are results from the second search (dataset s2) with same parameters as for the first search, except for --blat_tilesize = 6 and --exhaust_gap_size = 18 (three times the tilesize). This dataset represents improvements independent of Scipio. The red bars represent searches with same parameters as for dataset s1, except for the increased parameters --exhaust_align_size = 5,000 and --exhaust_gap_size = 25 (dataset s4). This data takes far longer to compute compared to the first search, because of the Needleman-Wunsch search in longer regions. For the DHC1 gene Scipio v1.0 maps too many amino acids of the human query sequence to the elephant genome. So the negative bar representing the other datasets shows that these datasets cover the right number of 4561 amino acids. (B) This diagram depicts the number of gaps (human query sequence not matched in the elephant genome) and questionable introns (intron?; introns with uncommon splice sites) for the searches with the old Scipio version and the new version applying different parameters as in (A). The detailed values of the diagrams are shown in tables in Additional file 3.

Figure 7 shows some example results of the cross-species search with genes of decreasing identity. The class-1 dynein heavy chain genes (DHC1) are very conserved between mammals, and the *Loxodonta *DHC1 could perfectly be reconstructed (except for the N-terminus that is not covered in the genome assembly). The DHC4A protein of *Loxodonta *has about 88 percent identity to the human homolog, and could also completely be reconstructed. In contrast, the DHC9B protein has only about 78 percent identity to the human homolog and the reconstructed gene still contains several gaps. The figure shows the result of the search using the old Scipio version compared to the result of the search with the new Scipio version. As reference, the result of the manual annotation of the gene is shown. It is very obvious that the new Scipio version provides a dramatically improved reconstruction of the *Loxodonta *DHC9B gene. More than 1,000 additional residues could be mapped corresponding to an increase in completeness by about 25 percent. The number of reconstructed exons increased from 62 to 80, which is close to the optimally reconstructed number of 85. The diagrams in Figure 8 show the improvements in gene reconstruction of the new Scipio version compared to the old version for the complete DHC dataset (see also Additional file 3). The reference for the perfectly reconstructed gene is the manual annotation based on the comparative annotation of more than 2,000 dynein heavy chain genes. The basis in diagram A is the reconstruction with Scipio v1.0, and shown are the improvements in the completeness of the annotation with Scipio v1.5 using different search parameters. In general, with the new Scipio version the reconstructions in these cross-species searches could considerably be improved. Lowering the tilesize, a Blat parameter to search with smaller fragments, further improved the results in only two cases. This corresponds to improvements independent of Scipio. However, extending the search frame for the exon search with the Needleman-Wunsch algorithm (parameter --exhaust_align_size) further completed the reconstruction in almost all cases demonstrating the effect of the newly introduced Needleman-Wunsch search for short or divergent exons.

### Comparison of gene reconstruction and prediction tools

We compared Scipio to other tools that reconstruct and predict genes based on a protein sequence, and to general gene prediction tools. The tools can be ordered in three categories. The tools of the first category reconstruct the exon-intron structure of the protein-coding genes based on a genomic sequence and a provided protein sequence. Scipio [14], Prosplign [39], Exonerate [40], and Prot_map [41] belong to this category. The second category includes the tools Fgenesh+ [42], GeneWise/Wise2 [43], and GenomeScan [44], that combine homology based gene reconstructions taking advantage of given protein sequences, and *ab initio *gene prediction approaches. The third group of software packages consists of *ab initio *gene prediction tools like Augustus [45], Fgenesh [42], and Genscan [46]. The latter tools are not really comparable with the other ones in the task of reconstructing single genes, but the comparison illustrates the differences of *ab initio *and homology based gene predictions. In addition to Blat, we tested Blast [47], which can also be used as an initial search for the Prosplign tool. However, for our test cases this approach did not improve the results of Prosplign (see Additional file 4).

To evaluate the performance of Scipio in comparison to the other tools, four test scenarios have been designed. The DHC proteins have been chosen as a large general test set, while the other examples used for the explanation of the new Scipio parameters have been used as a test set for genes difficult to reconstruct. Both test data sets have been explored in reconstructions/predictions out of whole genome assemblies and respective gene regions. This differentiation has been done because only a few of the above-mentioned tools could be used in searches against whole genomes due to the limited upload possibilities of the respective web-interfaces while command-line versions of the tools were not available for every software. Thus we tested the performance of all tools against the gene regions of the test data that correspond to the nucleotide sequence of the reference annotation plus 2,000 additional base pairs up- and downstream. To make the execution times comparable, the genome wide runs were performed on a dedicated server, which contains four 2.2Ghz AMD Opteron 6174 processors, with 12 cores each, and 128 GByte of memory.

#### Scenario 1

In the first scenario, the tools had to reconstruct the dynein heavy chain genes in the whole *Loxodonta africana *genome assembly based on the human protein sequences (Table 2). Besides Scipio, only Exonerate and Augustus were able to produce reasonable results. Prot_map, Fgenesh, and Fgenesh+ could not be tested in this scenario because the command-line versions are proprietary and it is not possible to upload whole genome sequences via their web-interfaces. WebScipio is the only tool available, which already provides genome sequences. The dynein heavy chain genes contain 1,202 annotated exons including 209,486 nucleotides. The *Loxodonta africana *genome contains 3,271,792,967 nucleotides including N's. For the DHC1 gene the N-terminus cannot be found in the genome sequence because of a gap in the genome assembly. We adjusted the start of the first known exon in the reference annotation to the predicted exon for each tool, because the start depends on whether a tool found an exon in front of the first known exon. The results of the first test scenario are presented in Table 2 (for more data see Additional file 4 and 5). Both Scipio and Exonerate in the standard mode are comparable in exon sensitivity (93.4% and 94.8%, respectively) and missed a similar amount of exons (11 exons and 6 exons, respectively). However, Exonerate predicted many wrong exons (5669 exons) resulting in a low specificity (16.5%, compared to 93.3% exon specificity by Scipio). Exonerate can be configured to report only the best hit by setting the --bestn option to 1. While this option increased the specificity (from 16.5% to 90.2%), the sensitivity decreased (from 94.8% to 73.4%). Also, the number of missing exons increased to 287.

**Table 2:** Test scenario 1: Reconstruction of the *Loxodonta africana *dynein heavy chain genes in the whole genome sequence based on human protein sequences

Tool	Predicted genes	Missing exons^1^	Wrong exons^2^	Exon sens. %	Exon sens. (ov.)^3 ^%	Exon spec. %	Nucl. sens. %	Nucl. spec. %	Execution time per prot. seq.
Scipio 1.5^4^	16	11	6	93.4	99.1	93.3	98.7	99.8	70 m 46s
Exonerate^5^	2145	6	5669	94.8	99.5	16.5	99.7	18.5	123 m 23s
Exonerate^6^	16	287	62	73.4	76.1	90.2	76.1	94.3	121 m 27s
									
Augustus^7^	1374928	0	3909434	47.9	100.0	0.0	100.0	0.3	> 10 days
									
BLAT^8^	-	9	264228	19.6	99.3	0.1	97.4	2.6	7 m 24s
Scipio 1.0^4^	16	14	46	86.1	98.8	83.2	97.9	99.4	8 m 24s

^1 ^Number of annotated exons, which are not overlapped by any predicted exon

^2 ^Number of predicted exons, which are not overlapped by any annotated exon

^3 ^Number annotated exons, which are overlapped by at least one predicted exon divided by the number of annotated exons

^4 ^Mammalia cross species default options (for detailed parameters see Additional file 5)

^5 ^Parameters: --model protein2genome

^6 ^Parameters: --model protein2genome --bestn 1

^7 ^Parameters: --species = human --genemodel = exactlyone (for more parameters see Additional file 5)

^8 ^Parameters as in ^4^: -tileSize = 7 -minIdentity = 54 -minScore = 15 -oneOff = 1

Comparing the results of Scipio and Blat illustrates that Blat found almost all exons, but that Scipio is needed to refine the exon borders as well as to exclude hits not related to the query sequence. Using the new Needleman-Wunsch algorithm Scipio v1.5 closes many gaps by adding and extending exons to the hits found by Blat. The number of missing exons is lower in Blat (9 exons missing) than in Scipio (11 exons missing), because Blat maps parts of the protein sequence to the genomic sequence, although these hits are not in the same order as in the protein sequence. Scipio excludes these hits. The results also show the great improvement of Scipio v1.5 compared to Scipio v1.0 in sensitivity (93.4% and 86.1%, respectively) and specificity,(93.3% and 83.2%, respectively). Altogether, these results show that Scipio v1.5 is the only free tool that is able to reconstruct the genes nearly complete in this scenario.

#### Scenario 2

The results of the second scenario are shown in Table 3. All above-mentioned tools were compared except for GenomeScan. Although GenomeScan produced results with the data provided on the respective webpage it did not work with our protein examples. The data show that Scipio performed in the same range as the other tools with respect to sensitivity and specificity. Scipio, Prosplign, and Exonerate revealed the highest sensitivity (94.7%, 95.7%, and 94.8%, respectively). Although Prosplign missed only one exon it also mis-predicted 41 exons. The homology based *ab initio *tools Fgenesh+ and Wise2 also provided almost complete reconstructions. Especially Fgenesh+ achieved high and balanced values for sensitivity (94.9%) and specificity (94.8%). The number of predicted genes illustrates that Exonerate without the --bestn option and Wise2 tend to divide long genes (32 and 39 genes predicted, respectively, instead of 16). The *ab initio *tools did not show comparable performance to the other tools in this scenario resulting in sensitivities of 76 - 82% and specificities of 58 - 83%. Augustus outperforms Fgenesh and Genscan with (Table 3) or without (see Additional file 4) the option to predict exactly one gene. Augusuts with the restriction to predict exactly one gene resulted in more accurate reconstructions. As in the whole genome scenario, the new Scipio v1.5 (93.1% sensitivity and 93.1% specificity) provides far better gene predictions than Blat and Scipio v1.0 (sensitivity of 19.9% and 86.2%, and specificity of 19.4% and 85.9%, respectively).

**Table 3:** Test scenario 2: Reconstruction of the *Loxodonta africana *dynein heavy chain gene structures in the respective gene regions based on human protein sequences

Tool	Pred. genes	Missing exons^1^	Wrong exons^2^	Exon sens. %	Exon sens. (ov.)^3 ^%	Exon spec. %	Nucl. sens. %	Nucl. spec. %
Scipio 1.5^4^	16	13	6	93.1	98.9	93.1	98.6	99.8
Scipio 1.5^5^	16	4	7	94.7	99.7	93.7	99.2	99.8
Prosplign^6^	16	1	41	95.7	99.9	92.6	99.9	98.7
Exonerate^7^	32	7	6	94.8	99.4	94.6	99.6	99.5
Exonerate^8^	16	255	4	75.7	78.8	95.6	79.2	99.7
Prot_map^9^	16	4	27	91.7	99.7	86.2	99.3	99.7
								
Fgenesh+^10^	16	10	10	94.9	99.2	94.8	99.0	99.7
Wise2^11^	39	3	16	93.3	99.8	91.2	99.7	98.9
								
Augustus^12^	16	132	111	81.9	89.0	83.2	89.9	88.7
Fgenesh^10^	161	111	342	80.2	90.8	67.3	91.8	62.3
Genscan^13^	194	138	520	76.3	88.5	57.9	90.4	55.3
								
BLAT^14^	-	16	19	19.9	98.7	19.4	97.0	98.9
Scipio 1.0^4^	16	16	10	86.2	98.7	85.9	97.8	99.8

^1 ^Number of annotated exons, which are not overlapped by any predicted exon

^2 ^Number of predicted exons, which are not overlapped by any annotated exon

^3 ^Number annotated exons, which are overlapped by at least one predicted exon divided by the number of annotated exons

^4 ^Mammalia cross species default options (for detailed parameters see Additional file 5)

^5 ^Mammalia cross species default options; -tileSize = 6 (for detailed parameters see Additional file 5)

^6 ^Parameters: -full -two_stages

^7 ^Parameters: --model protein2genome

^8 ^Parameters: --model protein2genome --bestn 1

^9 ^Similarity: Weak; Search for one best alignment only (for more parameters see Additional file 5)

^10 ^Organism: Human

^11 ^Parameters: -both

^12 ^Parameters: --species = human --genemodel = exactlyone (for more parameters see Additional file 5)

^13 ^Organism: Vertebrate; Suboptimal exon cutoff: 1.00

^14 ^Parameters as in ^4^: -tileSize = 7 -minIdentity = 54 -minScore = 15 -oneOff = 1

#### Scenario 3 and 4

In the third and forth scenario we compared the tools in their performance to reconstruct the difficult cases, which we introduced above by describing the new parameters of Scipio v1.5. In scenario 3 a search in the whole genome and in scenario 4 a search in the respective gene regions (as in scenario 2) was performed. Table 4 summarizes the results of the third and forth scenario. Only when using the latest version of Scipio the genes of the test data set could correctly be reconstructed and predicted in the whole genome assemblies as well as in the gene region. None of the other tools was able to reconstruct all genes correctly, even if the gene region was given as in the forth scenario.

**Table 4:** Test scenario 3 and 4: Difficult cases for reconstruction of gene structures

Tool	Ned kinesin	Phs dynactin p62	Hs dynactin p50	Pug coronin	Mm dynactin p150	Hs myosin	Th CAPα
Scipio 1.5^1^	✓	✓	✓	✓	✓	✓	✓
Prosplign^2^	o	o	✓	-	✓	-	✓
Exonerate^3^	o	o	-	-	✓	-	-
Prot_map^4^	o	o	-	-	-	o	-
							
Fgenesh+^5^	o	o	-	-	-	o	o
Wise2^6^	o	o	-	-	-	-	-
							
Augustus^7^	o	o	-	-	-	-	-
Fgenesh^5^	o	o	-	-	-	-	-
Genscan^8^	o	o	-	-	-	-	-
							
BLAT^9^	o	o	-	-	-	-	-
Scipio 1.0^1^	o	o	-	-	-	-	-

Ned: *Neurospora discreta*, Phs: *Phytophthora sojae*, Hs: *Homo sapiens*, Pug: *Puccinia graminis*, Mm: *Mus musculus*, Th: *Thielavia heterothallica*

✓ All exons are reconstructed correctly

o All annotated exons are matched by or overlap with predicted exons

- Exons are missing

^1 ^Ned, Phs: cross species default options; Hs, Mm: default options, exhaust_align_size = 15000; Pug, Th: default options (for detailed parameters see Figures 3, 4, and 5 and Additional file 5)

^2 ^Parameters: -full -two_stages

^3 ^Parameters: --model protein2genome

^4 ^Similarity: Weak; Search for one best alignment only (for more parameters see Additional file 5)

^5 ^Organisms: Ned, Th: Neurospora crassa; Phs: Phytophtora; Hs: Human; Pug: Puccina; Mm: Mouse

^6 ^Parameters: -both

^7 ^Parameters: --genemodel = exactlyone; Organisms: Ned: --species = neurospora; Phs, Pug, Th: --species = generic; Hs, Mm: --species = human (for more parameters see Additional file 5)

^8 ^Organism: Vertebrate; Suboptimal exon cutoff: 1.00

^9 ^Parameters: -minScore = 15; Ned, Phs: -tileSize = 5 -minIdentity = 54 -oneOff = 1; Hs, Pug, Mm, Th: -tileSize = 7 -minIdentity = 81

## Conclusions

Scipio and its graphical web-interface WebScipio are tools for the reconstruction of gene structures in eukaryotes. Scipio is based on the widely used program Blat that has been developed for aligning sequences of very high similarity. However, for the correct reconstruction of intron splice sites, very short exons, genes spread on several contigs, and the handling of sequencing errors a lot of post-processing is required. This is done by Scipio. Here, we present the fundamentally updated versions of Scipio and WebScipio, with an improved reconstruction of very short exons and intron splice sites, especially for the case of cross-species searches. To this end, we introduced a version of the Needleman-Wunsch algorithm that was shown to find a higher number of short exons previously missed, and to correct intron boundaries, especially in cases of lower sequence similarity. Furthermore, gaps in the mapping are now more frequently explained by divergent sequences, allowing for longer regions of insertions or deletions predicted on the same exon. Several parameters were introduced that can be used to fine-tune this behaviour if necessary. The sequence similarity between query and target sequence decreases with increasing evolutionary distance. While Blat is in principle able to locate hits for more distant species, the results become more and more incomplete, raising the importance of the post-processing. We could show that Scipio is now able to almost completely reconstruct genes from species whose ancestors separated more than 100 Myr ago. WebScipio allows easy access to Scipio and genome assemblies of about 640 eukaryotic species. This is unique to all gene reconstruction/prediction tools available and allows easy identification and reconstruction of protein homologs in related organisms. We compared the performance of Scipio to many other tools using our test data. While there are only minor differences in the reconstruction of the mammalian dynein heavy chain genes between Scipio, Exonerate, Prosplign, and Fgenesh+, the other software tools were not able to correctly reconstruct the more difficult cases encoding very short exons and showing strong sequence divergence at intron borders or inside of exons. Also unique to Scipio, this is the only tool available that is able to correctly reconstruct and predict genes that are spread on several contigs.

## Availability and requirements

Project name: WebScipio, Scipio

Project home page: http://www.webscipio.org

Operating system: Platform independent

Programming languages: Ruby, Perl

Software requirements: Installation of Blat and BioPerl for using Scipio as command-line tool. WebScipio has been tested with InternetExplorer, Firefox, Chrome, Safari, and Opera.

License: WebScipio and Scipio may be obtained upon request and used under a GNU General Public License.

Any restrictions to use by non-academics: Using WebScipio and Scipio by non-academics requires permission.

## List of abbreviations

Blat: BLAST like alignment tool; FTP: File transfer protocol; HTML: Hypertext markup language; SVG: Scalable vector graphics; PNG: Portable network graphics; YAML: YAML ain't markup language.

## Competing interests

The authors declare that they have no competing interests.

## Authors' contributions

KH and MK set the requirements for the system and wrote the manuscript. KH and BH wrote the WebScipio software. HP implemented the test environment. OK wrote the Scipio source code and assisted in writing the manuscript. SW supervised the implementation of Scipio. KH, HP, OK, and MK performed extensive testing. KH performed the comparative software analysis. All authors read and approved the final version of the manuscript.

## Supplementary Material

Additional file 1 **Activity flow of the hit processing step**. The scheme shows a detailed activity flow of the hit processing step. Here, the experienced user can see, where and how the various expert parameters modulate Scipio's hit processing, and can thus adjust these parameters to get the best result possible. Click here for file

Additional file 2 **Protein - DNA alignments corresponding to the example searches**. Here, additional data corresponding to the example searches is provided. Click here for file

Additional file 3 **Table with detailed data of the results of the cross-species search of the human DHC genes in the elephant genome**. The table provides detailed data to the cross-species searches including numbers of matches and mismatches, gaps and intron?'s, for the searches with different parameters. Click here for file

Additional file 4 **Detailed evaluation values used for **Tables 2, 3, and 4. This file provides a description of each evaluation parameter and the values obtained with each software tool for all sequence predictions. The values highlighted in yellow were used for Tables 2, 3, and 4. Click here for file

Additional file 5 **Software versions and run parameters of the gene reconstruction and prediction tools**. The tables shows the exact versions and run parameters, which were used for the comparison, for each scenario. Click here for file

## References

[B1] Mardis ER A decade's perspective on DNA sequencing technology Nature 2011 470 7333 198 203 10.1038/nature09796 21307932

[B2] Gardner MJ Hall N Fung E White O Berriman M Hyman RW Carlton JM Pain A Nelson KE Bowman S Genome sequence of the human malaria parasite Plasmodium falciparum Nature 2002 419 6906 498 511 10.1038/nature01097 12368864 PMC3836256

[B3] Tyler BM Tripathy S Zhang X Dehal P Jiang RH Aerts A Arredondo FD Baxter L Bensasson D Beynon JL Phytophthora genome sequences uncover evolutionary origins and mechanisms of pathogenesis Science 2006 313 5791 1261 1266 10.1126/science.1128796 16946064

[B4] Clark AG Eisen MB Smith DR Bergman CM Oliver B Markow TA Kaufman TC Kellis M Gelbart W Iyer VN Evolution of genes and genomes on the Drosophila phylogeny Nature 2007 450 7167 203 218 10.1038/nature06341 17994087

[B5] Butler G Rasmussen MD Lin MF Santos MA Sakthikumar S Munro CA Rheinbay E Grabherr M Forche A Reedy JL Evolution of pathogenicity and sexual reproduction in eight Candida genomes Nature 2009 459 7247 657 662 10.1038/nature08064 19465905 PMC2834264

[B6] Srivastava M Begovic E Chapman J Putnam NH Hellsten U Kawashima T Kuo A Mitros T Salamov A Carpenter ML The Trichoplax genome and the nature of placozoans Nature 2008 454 7207 955 960 10.1038/nature07191 18719581

[B7] Prochnik SE Umen J Nedelcu AM Hallmann A Miller SM Nishii I Ferris P Kuo A Mitros T Fritz-Laylin LK Genomic analysis of organismal complexity in the multicellular green alga Volvox carteri Science 2010 329 5988 223 226 10.1126/science.1188800 20616280 PMC2993248

[B8] Picardi E Pesole G Computational methods for ab initio and comparative gene finding Methods Mol Biol 2010 609 269 284 10.1007/978-1-60327-241-4_16 20221925

[B9] Wei C Brent MR Using ESTs to improve the accuracy of de novo gene prediction BMC Bioinformatics 2006 7 327 10.1186/1471-2105-7-327 16817966 PMC1534067

[B10] Stanke M Tzvetkova A Morgenstern B AUGUSTUS at EGASP: using EST, protein and genomic alignments for improved gene prediction in the human genome Genome Biol 2006 7 Suppl 1 S11 11 18 10.1186/gb-2006-7-s1-s11 PMC1810548 16925833

[B11] Odronitz F Kollmar M Drawing the tree of eukaryotic life based on the analysis of 2,269 manually annotated myosins from 328 species Genome Biol 2007 8 9 R196 10.1186/gb-2007-8-9-r196 17877792 PMC2375034

[B12] Babenko VN Rogozin IB Mekhedov SL Koonin EV Prevalence of intron gain over intron loss in the evolution of paralogous gene families Nucleic Acids Res 2004 32 12 3724 3733 10.1093/nar/gkh686 15254274 PMC484173

[B13] Roy SW Gilbert W Rates of intron loss and gain: implications for early eukaryotic evolution Proc Natl Acad Sci USA 2005 102 16 5773 5778 10.1073/pnas.0500383102 15827119 PMC556292

[B14] Keller O Odronitz F Stanke M Kollmar M Waack S Scipio: using protein sequences to determine the precise exon/intron structures of genes and their orthologs in closely related species BMC Bioinformatics 2008 9 278 10.1186/1471-2105-9-278 18554390 PMC2442105

[B15] Odronitz F Pillmann H Keller O Waack S Kollmar M WebScipio: an online tool for the determination of gene structures using protein sequences BMC Genomics 2008 9 422 10.1186/1471-2164-9-422 18801164 PMC2644328

[B16] Kent WJ BLAT--the BLAST-like alignment tool Genome Res 2002 12 4 656 664 11932250 10.1101/gr.229202 PMC187518

[B17] van Nimwegen E Paul N Sheridan R Zavolan M SPA: a probabilistic algorithm for spliced alignment PLoS Genet 2006 2 4 e24 10.1371/journal.pgen.0020024 16683023 PMC1449883

[B18] The Perl Programming Language http://www.perl.org

[B19] Ruby Programming Language http://www.ruby-lang.org/

[B20] Ruby on Rails http://rubyonrails.org

[B21] Prototype JavaScript framework: Easy Ajax and DOM manipulation for dynamic web applications http://www.prototypejs.org

[B22] script.aculo.us - web 2.0 javascript http://script.aculo.us

[B23] Odronitz F Becker S Kollmar M Reconstructing the phylogeny of 21 completely sequenced arthropod species based on their motor proteins BMC Genomics 2009 10 173 10.1186/1471-2164-10-173 19383156 PMC2674883

[B24] CyMoBase http://www.cymobase.org/

[B25] Odronitz F Hellkamp M Kollmar M diArk--a resource for eukaryotic genome research BMC Genomics 2007 8 103 10.1186/1471-2164-8-103 17439643 PMC1868023

[B26] W3C SVG Working Group http://www.w3.org/Graphics/SVG/

[B27] Inkscape. Draw Freely http://inkscape.org

[B28] Goto N Prins P Nakao M Bonnal R Aerts J Katayama T BioRuby: Bioinformatics software for the Ruby programming language Bioinformatics 2010 10.1093/bioinformatics/btq475 PMC2951089 20739307

[B29] The Official YAML Web Site http://www.yaml.org/

[B30] purzelrakete's workling at master - GitHub http://github.com/purzelrakete/workling

[B31] tra's spawn at master - GitHub http://github.com/tra/spawn

[B32] Tokyo Cabinet: a modern implementation of DBM http://fallabs.com/tokyocabinet/

[B33] Hoptoad: The app error app http://hoptoadapp.com

[B34] RSpec.info: Home http://rspec.info

[B35] Selenium web application testing system http://seleniumhq.org

[B36] Yoon SJ Seiler SH Kucherlapati R Leinwand L Organization of the human skeletal myosin heavy chain gene cluster Proc Natl Acad Sci USA 1992 89 24 12078 12082 10.1073/pnas.89.24.12078 1465443 PMC50701

[B37] Deutsch M Long M Intron-exon structures of eukaryotic model organisms Nucleic Acids Res 1999 27 15 3219 3228 10.1093/nar/27.15.3219 10454621 PMC148551

[B38] Benton MJ Donoghue PC Paleontological evidence to date the tree of life Mol Biol Evol 2007 24 1 26 53 17047029 10.1093/molbev/msl150

[B39] Sayers EW Barrett T Benson DA Bolton E Bryant SH Canese K Chetvernin V Church DM DiCuccio M Federhen S Database resources of the National Center for Biotechnology Information Nucleic Acids Res 2011 39 Database D38 51 10.1093/nar/gkq1172 PMC3013733 21097890

[B40] Slater GS Birney E Automated generation of heuristics for biological sequence comparison BMC Bioinformatics 2005 6 31 10.1186/1471-2105-6-31 15713233 PMC553969

[B41] Solovyev V Kosarev P Seledsov I Vorobyev D Automatic annotation of eukaryotic genes, pseudogenes and promoters Genome Biol 2006 7 Suppl 1 S10 11 12 10.1186/gb-2006-7-s1-s10 PMC1810547 16925832

[B42] Salamov AA Solovyev VV Ab initio gene finding in Drosophila genomic DNA Genome Res 2000 10 4 516 522 10.1101/gr.10.4.516 10779491 PMC310882

[B43] Birney E Clamp M Durbin R GeneWise and Genomewise Genome Res 2004 14 5 988 995 10.1101/gr.1865504 15123596 PMC479130

[B44] Yeh RF Lim LP Burge CB Computational inference of homologous gene structures in the human genome Genome Res 2001 11 5 803 816 10.1101/gr.175701 11337476 PMC311055

[B45] Stanke M Waack S Gene prediction with a hidden Markov model and a new intron submodel Bioinformatics 2003 19 Suppl 2 ii215 225 10.1093/bioinformatics/btg1080 14534192

[B46] Burge C Karlin S Prediction of complete gene structures in human genomic DNA J Mol Biol 1997 268 1 78 94 10.1006/jmbi.1997.0951 9149143

[B47] Altschul SF Madden TL Schaffer AA Zhang J Zhang Z Miller W Lipman DJ Gapped BLAST and PSI-BLAST: a new generation of protein database search programs Nucleic Acids Res 1997 25 17 3389 3402 10.1093/nar/25.17.3389 9254694 PMC146917

