# Supplementary figures and images for: Cross-species protein sequence and gene structure prediction with fine-tuned Webscipio 2.0 and Scipio
Source: BMC Res Notes. 2011 Jul 28;4:265. doi: 10.1186/1756-0500-4-265 (PMC3162530; doi:10.1186/1756-0500-4-265)

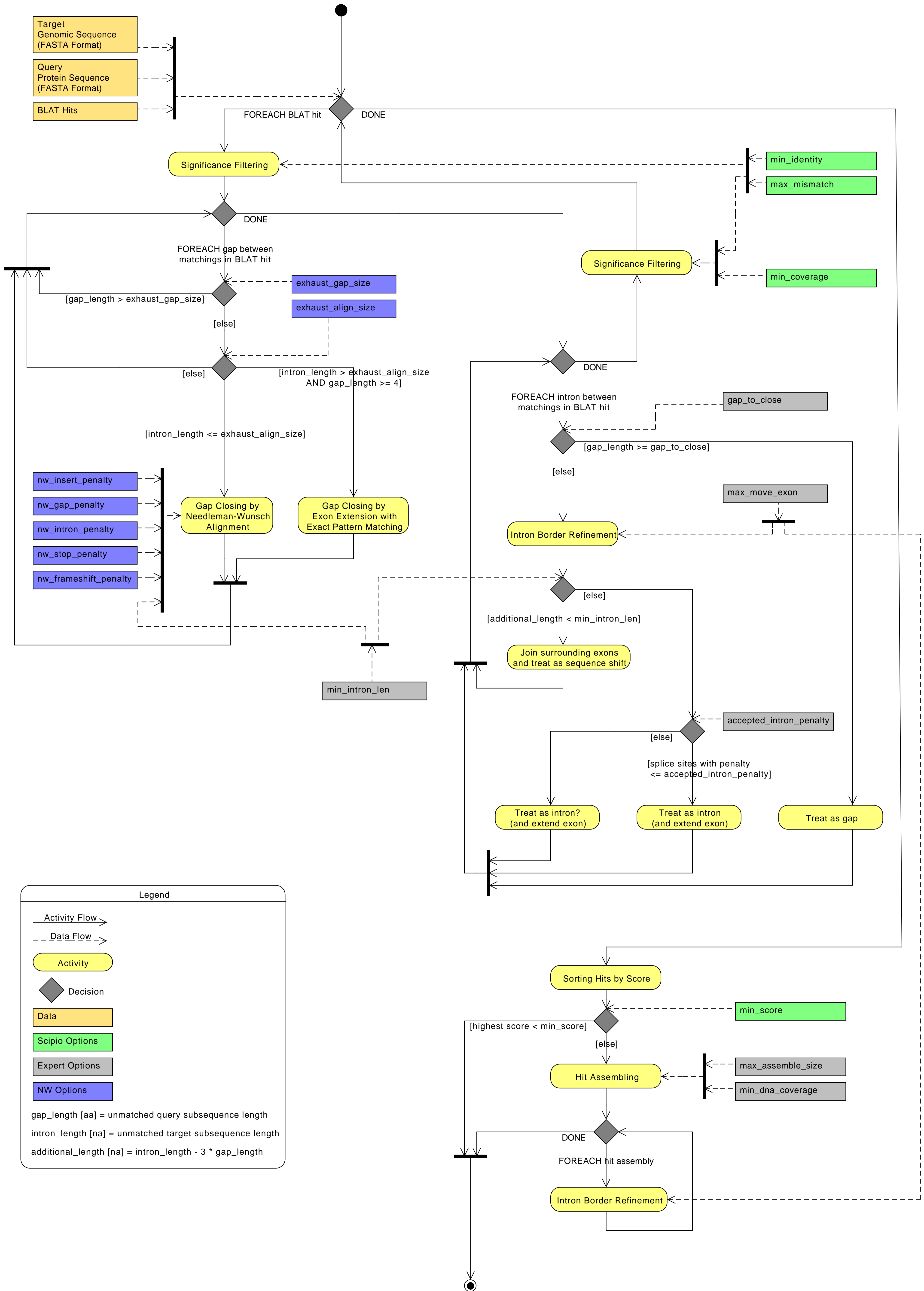

Supplement: Additional file 1 — Activity flow of the hit processing step. The scheme shows a detailed activity flow of the hit processing step. Here, the experienced user can see, where and how the various expert parameters modulate Scipio's hit processing, and can thus adjust these parameters to get the best result possible. [file 1756-0500-4-265-S1.PDF]
