# Supplementary material for: Cross-species protein sequence and gene structure prediction with fine-tuned Webscipio 2.0 and Scipio
Source: BMC Res Notes. 2011 Jul 28;4:265. doi: 10.1186/1756-0500-4-265 (PMC3162530; doi:10.1186/1756-0500-4-265)
Supplement: Additional file 2 — Protein - DNA alignments corresponding to the example searches. Here, additional data corresponding to the example searches is provided. [file 1756-0500-4-265-S2.PDF]

Parameters to account for additional/missing bases in gene predictions

During the evolution of species it happens that homologs of a protein cut off or elongate surface loops, resulting in rather long deletions or insertions in the cross-species alignment. Scipio v1.0 (based on Blat) wrongly interpreted inserted sequence as an intron, splitting the exon into two. Now, in the latest Scipio version, we have introduced parameters that fix the maximum number of additional bases in the target sequence (that will become part of the predicted exon), and that allow additional amino acids in the query not covered by the target DNA, while in the previous version, this caused a gap splitting the exon.

Example A

For the example shown here, the goal was to identify the homolog of a *Neurosopora crassa* kinesin motor protein in *Neurospora discreta*. Figures 1 and 2 show the alignment of the query sequence to the target sequence for the output generated by Scipio v1.0 and v1.5, respectively.

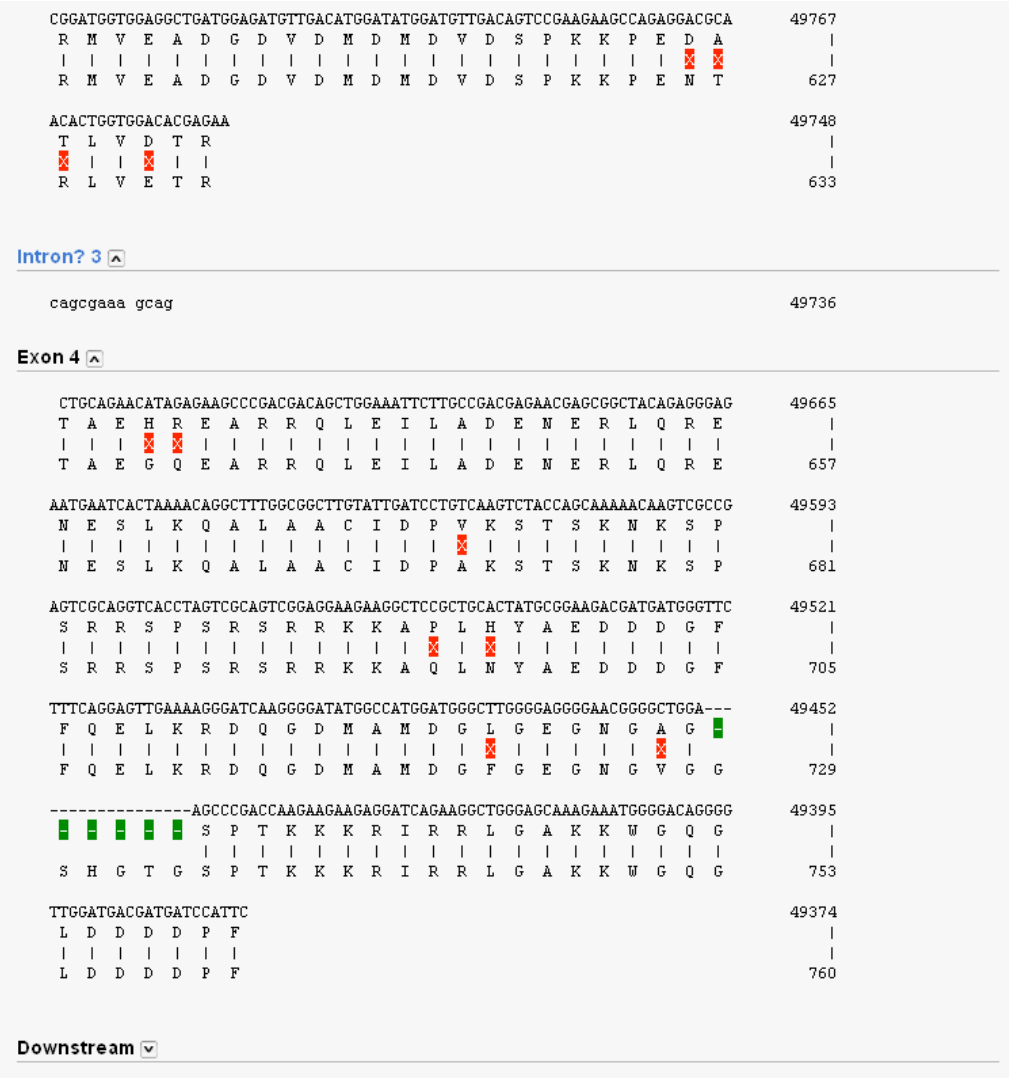

Figure 1: Section of the output generated by Scipio v1.0. Too many additional bases in the target sequence were treated as (insecure) intron.

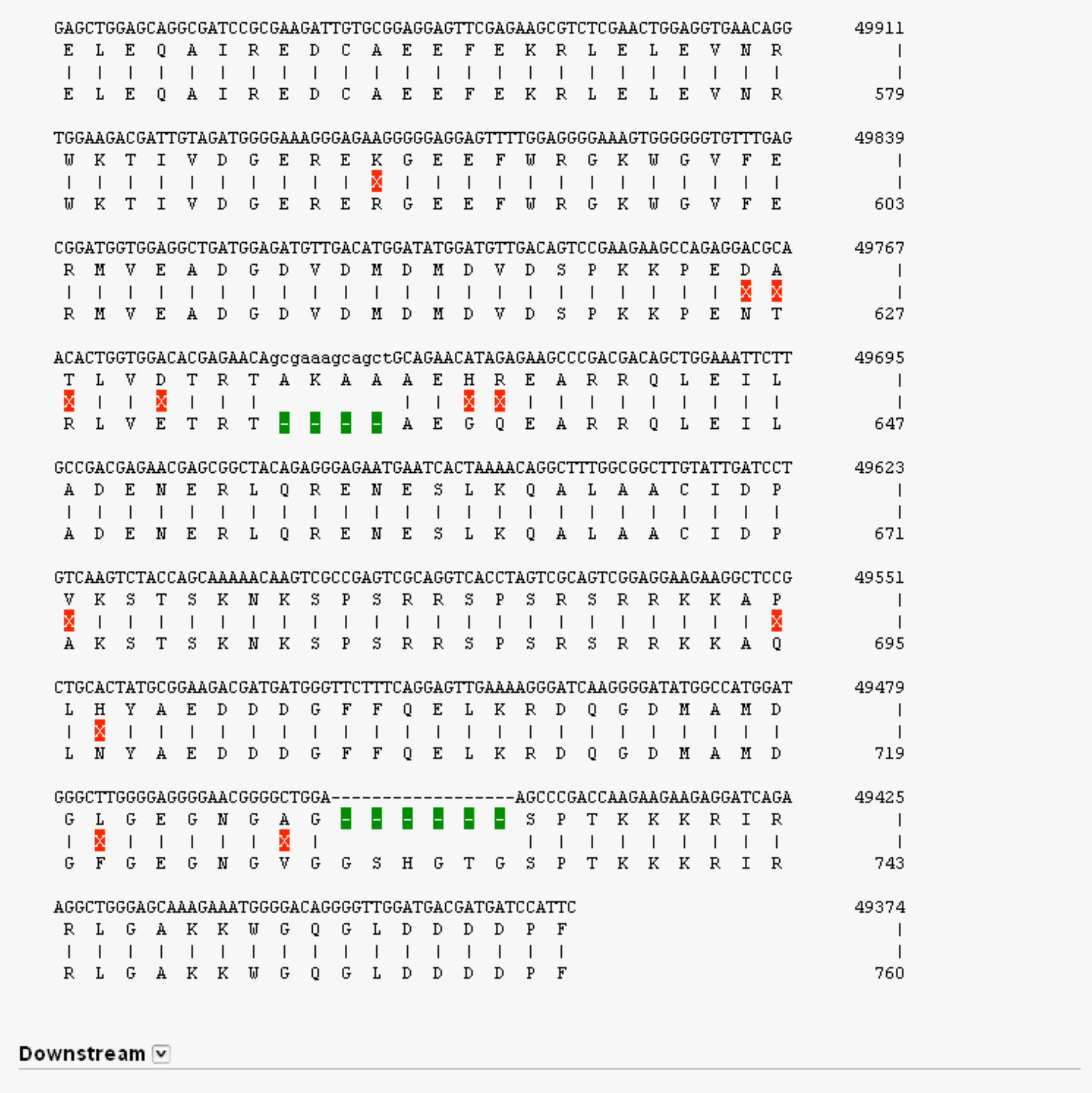

Figure 2: Section of the output generated by Scipio v1.5. Additional and/or missing bases are now treated as part of the same exon. The number of additional bases/nucleotides can be adjusted with the new parameters --min_intron_length [na] and --gap_to_close [aa].

Example B

For the other example shown, the goal was to identify the homolog of a dynactin p62 homolog *from Phytophthora ramorum* in *Phytophthora sojae*. Figures 3 and 4 show the alignment of the query sequence to the target sequence for the output generated by Scipio v1.0 and v1.5, respectively.

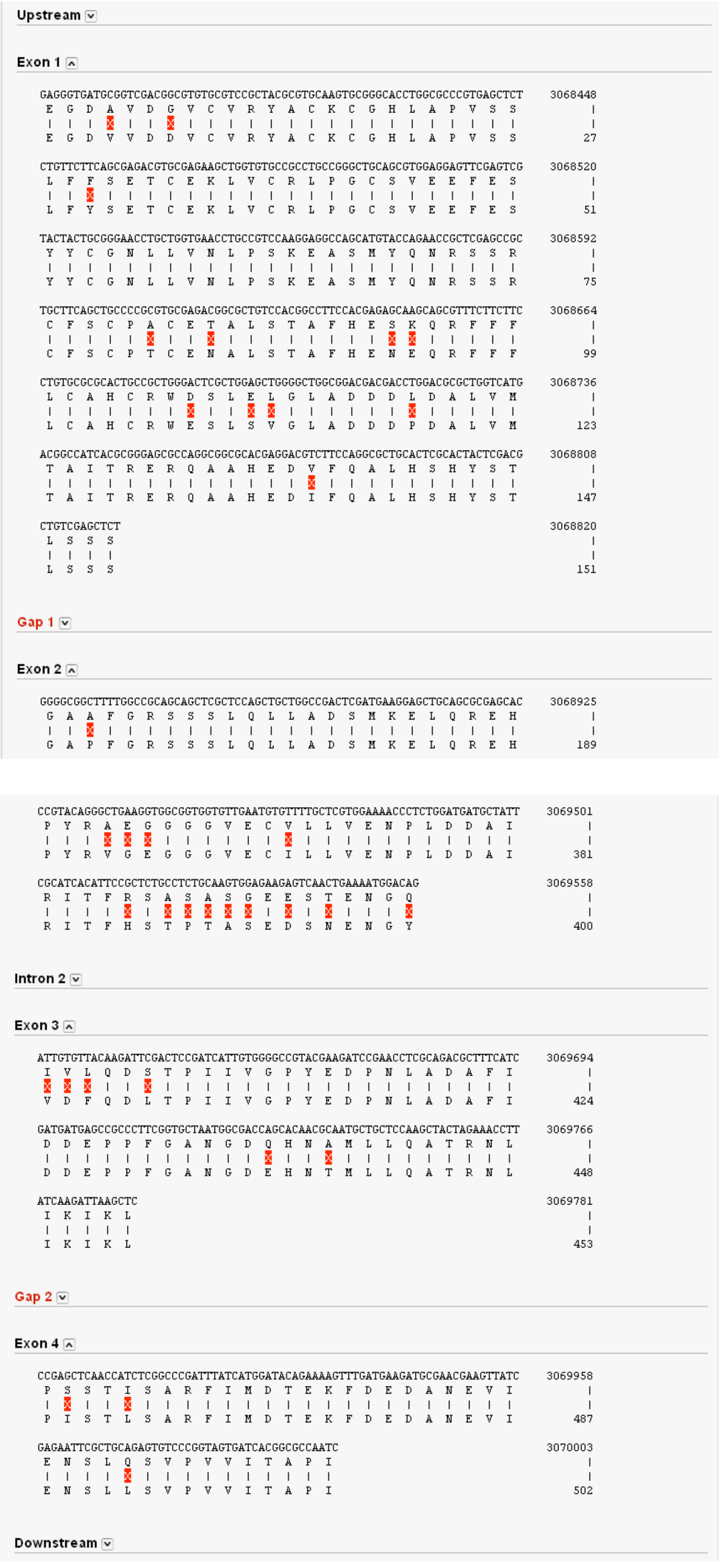

Figure 3: Section of the output generated by Scipio v1.0. The upper figure shows the 5’ end of the gene, the lower figure the 3’ end. Too many mismatches together with additional query sequence (query sequence longer than the target sequence) could not be mapped and were given as gaps. In addition, the N- and the C-termini of the gene could not be resolved.

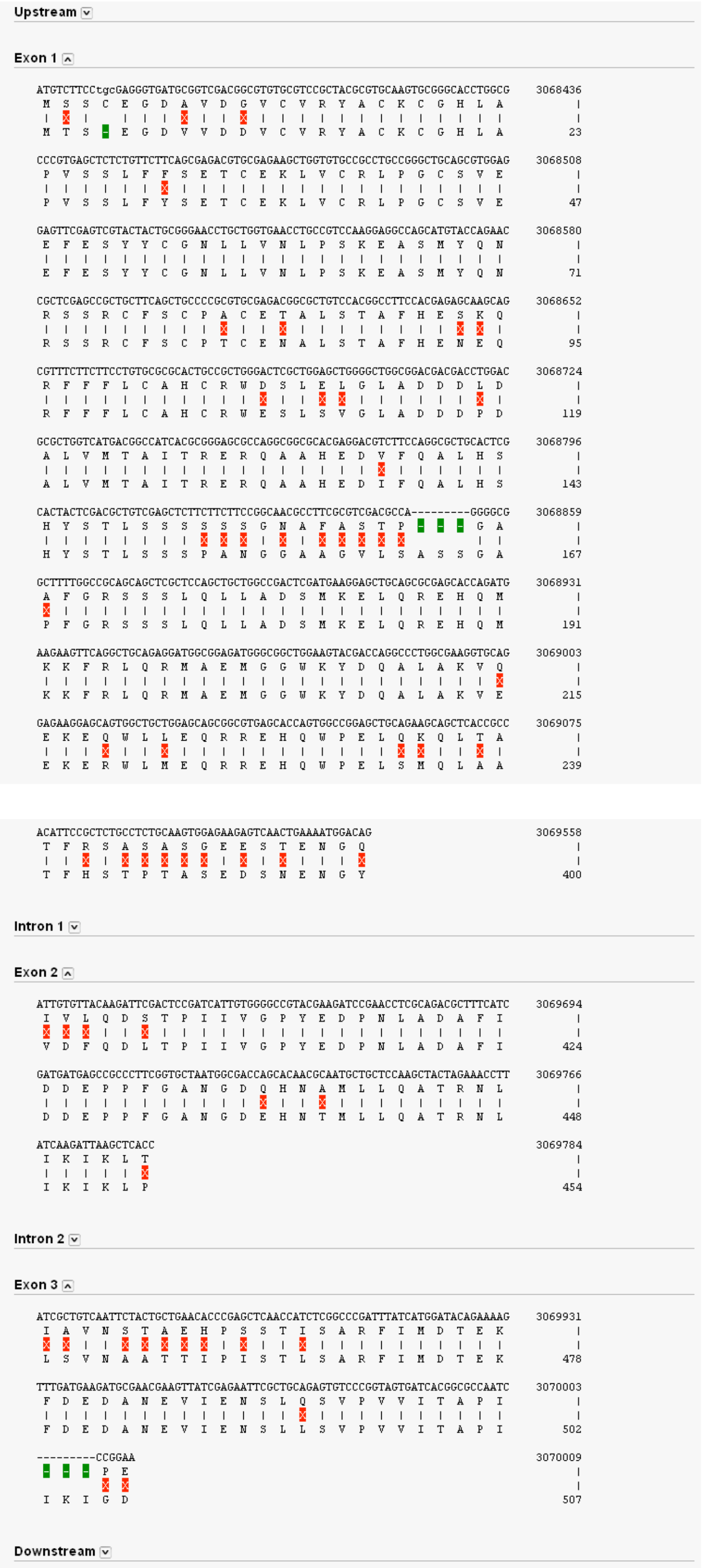

Figure 4: Section of the output generated by Scipio v1.5. The upper figure shows the 5’ end of the gene, the lower figure the 3’ end. Additional and/or missing bases are now treated as part of the exon (marked in green). The number of additional/missing bases can be adjusted with the new Scipio-parameters --min_intron_length [na] and --gap_to_close [aa].

Parameters to account for reconstruction of very short exons

Example A

The dynamitin (p50 subunit of the dynactin complex) gene of *Homo sapiens* contains two very small exons (3 amino acids and 2 amino acids). These exons are differentially included in the mature RNA. Scipio v1.0 was not able to identify internal exons that were not already found by Blat (Figure 5). The new Scipio version, v1.5, successfully identified the small exons using the Needleman-Wunsch algorithm and increasing the --exhaust_align_size to 15,000 bp (Figure 6).

```
Upstream

Exon 1

   ATGGCGGACCCTAAATACGCCGACCTTCCCGGCATT                                     56227077
    M  A  D  P  K  Y  A  D  L  P  G  I                                             |
    |  |  |  |  |  |  |  |  |  |  |  |                                             |
    M  A  D  P  K  Y  A  D  L  P  G  I                                            12

Intron 1

Exon 2

   GCCAGGAATGAGCCAGATGTTTATGAAACTAGCGACCTACCTGAGGATGATCAAGCGGAGTTCGATGCG    56226077
    A  R  N  E  P  D  V  Y  E  T  S  D  L  P  E  D  D  Q  A  E  F  D  A            |
    |  |  |  |  |  |  |  |  |  |  |  |  |  |  |  |  |  |  |  |  |  |  |            |
    A  R  N  E  P  D  V  Y  E  T  S  D  L  P  E  D  D  Q  A  E  F  D  A           35

Gap 1

Exon 3

   GAGGAGCTGACAAGCACAAGTGTGGAACACATCATTGTCAATCCTAATGCTGCCTATGACAAGTTCAAGGAC 56215823
    E  E  L  T  S  T  S  V  E  H  I  I  V  N  P  N  A  A  Y  D  K  F  K  D         |
    |  |  |  |  |  |  |  |  |  |  |  |  |  |  |  |  |  |  |  |  |  |  |  |         |
    E  E  L  T  S  T  S  V  E  H  I  I  V  N  P  N  A  A  Y  D  K  F  K  D        64

   AAGAGAGTGGGGACAAAGGGACTTG                                                56215798
    K  R  V  G  T  K  G  L                                                         |
    |  |  |  |  |  |  |  |                                                         |
    K  R  V  G  T  K  G  L                                                        72

Intron 3

Exon 4

    ATTTCTCAGATCGTATTGGAAAAACCAAGAGGACAGGATATGAATCTGGAGAATATGAGATG          56215533
    D  F  S  D  R  I  G  K  T  K  R  T  G  Y  E  S  G  E  Y  E  M                  |
    |  |  |  |  |  |  |  |  |  |  |  |  |  |  |  |  |  |  |  |  |                  |
    D  F  S  D  R  I  G  K  T  K  R  T  G  Y  E  S  G  E  Y  E  M                 93

Intron 4
```

Figure 5: DNA-protein alignment of the 5'-end of the dynamitin gene of *Homo sapiens* reconstructed with Scipio v1.0. Two small exons were not identified, leaving a gap of five residues.

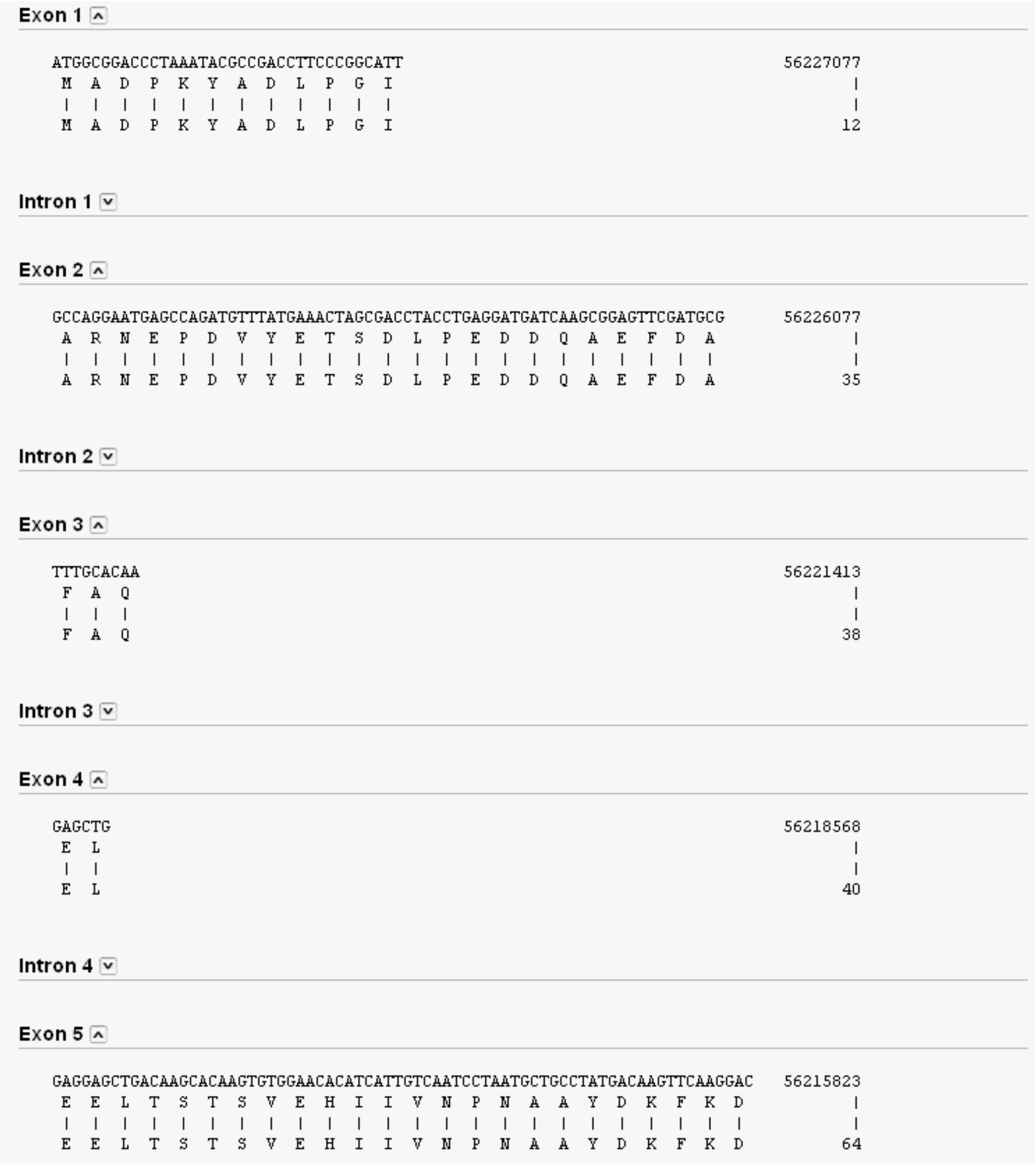

Figure 6: DNA-protein alignment of the 5'-end of the dynamitin gene of *Homo sapiens* reconstructed with Scipio v1.5. The two small exons were correctly identified and the surrounding introns start and end with the most common splice sites.

Example B

The coronin genes of *Puccinia graminis f. sp. tritici* and *Melampsora populina* each contain a very small conserved internal exon, in addition to the N-terminal exon that is encoded by a single methionine. In contrast to the dynamitin example, these coronin gene exons contain split codons at both sites (Figure 7 and 8). Because these three residues are conserved in all coronins of *Basidiomycotes* (but are part of the neighboring exons in these species), and are present but divergent in all fungi, these small exons are true and not artificial exons.

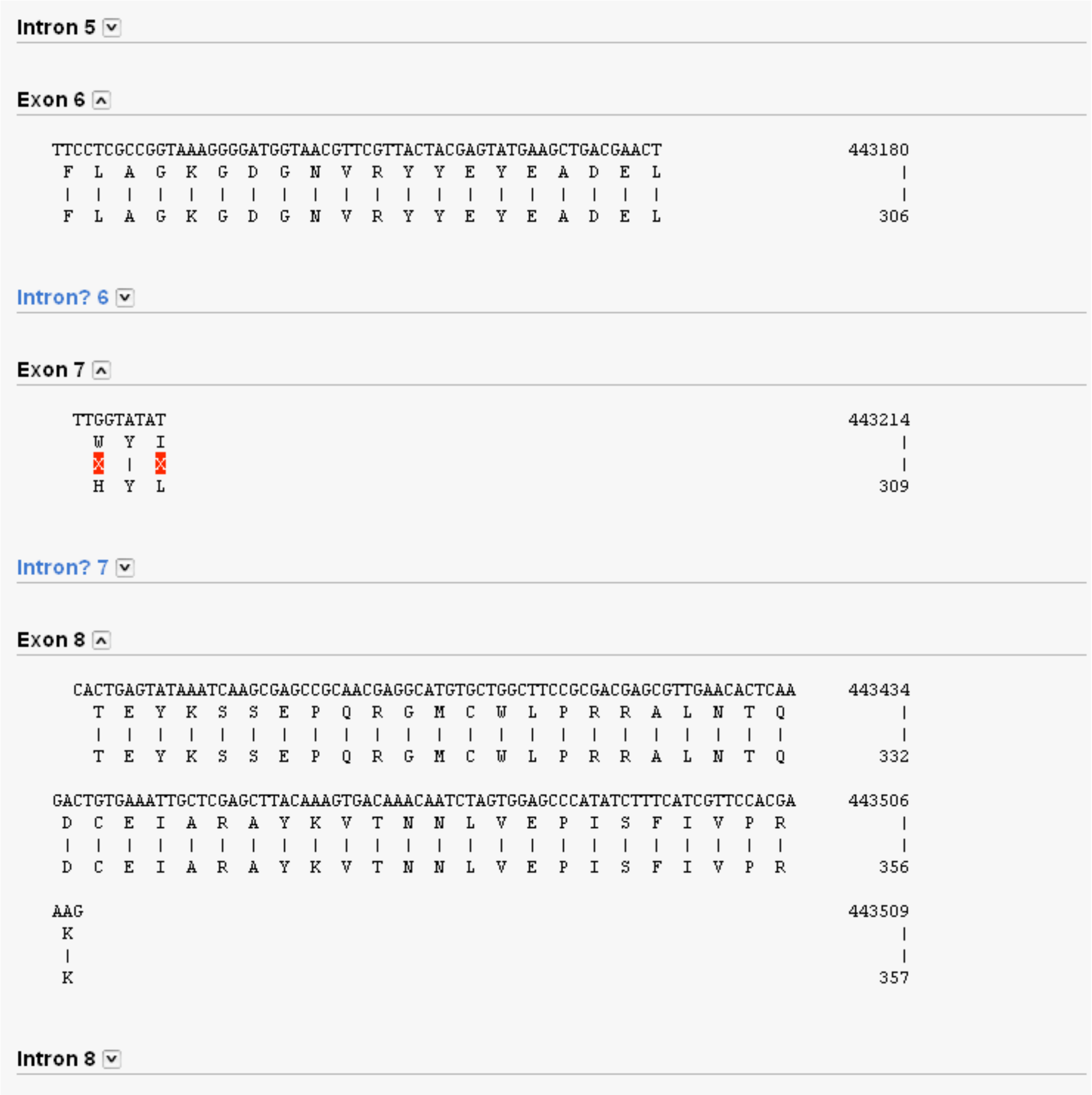

Figure 7: DNA-protein alignment of the middle of the coronin gene of *Puccinia graminis f. sp. Tritici*, with the small exon 7 incorrectly reconstructed with Scipio v1.0.

```
  ATTGATACCAGCTCTGGTACTCTAATGCCCTTTTGGTCTGACAACGACATTTTG                        443035
   I  D  T  S  S  G  T  L  M  P  F  W  S  D  N  D  I  L                               |
   |  |  |  |  |  |  |  |  |  |  |  |  |  |  |  |  |  |                               |
   I  D  T  S  S  G  T  L  M  P  F  W  S  D  N  D  I  L                             286
```

Intron 5

Exon 6

```
  TTCCTCGCCGGTAAAGGGGATGGTAACGTTCGTTACTACGAGTATGAAGCTGACGAACT                   443180
   F  L  A  G  K  G  D  G  N  V  R  Y  Y  E  Y  E  A  D  E  L                         |
   |  |  |  |  |  |  |  |  |  |  |  |  |  |  |  |  |  |  |  |                         |
   F  L  A  G  K  G  D  G  N  V  R  Y  Y  E  Y  E  A  D  E  L                       306
```

Intron 6

Exon 7

```
    ACACTACCT                                                                   443293
      H  Y  L                                                                        |
      |  |  |                                                                        |
      H  Y  L                                                                      309
```

Intron 7

Exon 8

```
    CACTGAGTATAAATCAAGCGAGCCGCAACGAGGCATGTGCTGGCTTCCGCGACGAGCGTTGAACACTCAA      443434
      T  E  Y  K  S  S  E  P  Q  R  G  M  C  W  L  P  R  R  A  L  N  T  Q            |
      |  |  |  |  |  |  |  |  |  |  |  |  |  |  |  |  |  |  |  |  |  |  |            |
      T  E  Y  K  S  S  E  P  Q  R  G  M  C  W  L  P  R  R  A  L  N  T  Q          332

  GACTGTGAAATTGCTCGAGCTTACAAAGTGACAAACAATCTAGTGGAGCCCATATCTTTCATCGTTCCACGA      443506
   D  C  E  I  A  R  A  Y  K  V  T  N  N  L  V  E  P  I  S  F  I  V  P  R            |
   |  |  |  |  |  |  |  |  |  |  |  |  |  |  |  |  |  |  |  |  |  |  |  |            |
   D  C  E  I  A  R  A  Y  K  V  T  N  N  L  V  E  P  I  S  F  I  V  P  R          356

  AAG                                                                           443509
   K                                                                                 |
   |                                                                                 |
   K                                                                               357
```

Figure 8: DNA-protein alignment of the middle of the coronin gene of *Puccinia graminis f. sp. tritici* reconstructed with Scipio v1.5, using the Needleman-Wunsch algorithm with standard parameters. Exon 7 has correctly been reconstructed.

Example C

The dynactin p150 gene of *Mus musculus* contains three very small exons (7, 6, and 7 amino acids, respectively). These exons are differentially included in the mature RNA. Scipio v1.0 was not able to identify internal exons that were not already found by Blat (Figure 9). The new Scipio version, v1.5, successfully identified the small exons using the Needleman-Wunsch algorithm (Figure 10). The --exhaust_align_size had to be increased to 10,000 bp, and the --exhaust_gap_size to 21 aa.

```
Exon 3

ATCCAAGTATTTGAAGATGGAGCAGATACTACTTCCCCAGAGACTCCTGATTCTTCTGCTTCAAAGGTCCTC   83148788
 I  Q  V  F  E  D  G  A  D  T  T  S  P  E  T  P  D  S  S  A  S  K  V  L          |
 |  |  |  |  |  |  |  |  |  |  |  |  |  |  |  |  |  |  |  |  |  |  |  |          |
 I  Q  V  F  E  D  G  A  D  T  T  S  P  E  T  P  D  S  S  A  S  K  V  L        117

AAGAGAG                                                                    83148795
 K  R                                                                            |
 |  |                                                                            |
 K  R                                                                          119

Intron 3

Exon 4

 AGGGAGCCGATGCAGCTGCAAAGACCAGCAAACTG                                       83148991
 E  G  A  D  A  A  A  K  T  S  K  L                                              |
 |  |  |  |  |  |  |  |  |  |  |  |                                              |
 E  G  A  D  A  A  A  K  T  S  K  L                                            131

Gap 1

Exon 5

CCTACTCGCCCAGCCAGCACTGGGGTGGCTGGGCCCAGTAGCTCCCTTGGCCCCTCTGGCTCAGCGTCAGCC   83154806
 P  T  R  P  A  S  T  G  V  A  G  P  S  S  S  L  G  P  S  G  S  A  S  A          |
 |  |  |  |  |  |  |  |  |  |  |  |  |  |  |  |  |  |  |  |  |  |  |  |          |
 P  T  R  P  A  S  T  G  V  A  G  P  S  S  S  L  G  P  S  G  S  A  S  A        175

GGGGAACTAAGCAGCAGTGAGCCCAGCACCCCAGCTCAGACTCCGCTGGCAGCACCCATCATCCCCACACCG   83154878
 G  E  L  S  S  S  E  P  S  T  P  A  Q  T  P  L  A  A  P  I  I  P  T  P          |
 |  |  |  |  |  |  |  |  |  |  |  |  |  |  |  |  |  |  |  |  |  |  |  |          |
 G  E  L  S  S  S  E  P  S  T  P  A  Q  T  P  L  A  A  P  I  I  P  T  P        199

GCCCTCACCTCTCCTGGAGCAGCACCCCCACTTCCATCTCCCTCTAAG                           83154926
 A  L  T  S  P  G  A  A  P  P  L  P  S  P  S  K                                  |
 |  |  |  |  |  |  |  |  |  |  |  |  |  |  |  |                                  |
 A  L  T  S  P  G  A  A  P  P  L  P  S  P  S  K                                215

Intron 5

Exon 6

GAAGAGGAAGGGCTGAGGGCTCAGGTACGGGACCTGGAGGAGAAGCTGGAGACCCTGCGCCTAAAACGCTCA   83155277
 E  E  E  G  L  R  A  Q  V  R  D  L  E  E  K  L  E  T  L  R  L  K  R  S          |
 |  |  |  |  |  |  |  |  |  |  |  |  |  |  |  |  |  |  |  |  |  |  |  |          |
 E  E  E  G  L  R  A  Q  V  R  D  L  E  E  K  L  E  T  L  R  L  K  R  S        239
```

Figure 9: DNA-protein alignment of the 5'-end of the dynactin p150 gene of *Mus musculus* reconstructed with Scipio v1.0. Three small exons were not identified, leaving a gap of 20 residues.

```
Exon 4
  AGGGAGCCGATGCAGCTGCAAAGACCAGCAAACTG                                83148991
  E  G  A  D  A  A  A  K  T  S  K  L                                        |
  |  |  |  |  |  |  |  |  |  |  |  |                                        |
  E  G  A  D  A  A  A  K  T  S  K  L                                      131

Intron 4

Exon 5
 CGGGGACTGAAGCCTAAGAAG                                               83149614
  R  G  L  K  P  K  K                                                       |
  |  |  |  |  |  |  |                                                       |
  R  G  L  K  P  K  K                                                     138

Intron 5

Exon 6
 GCACCGACAGCCCGAAAG                                                  83152103
  A  P  T  A  R  K                                                          |
  |  |  |  |  |  |                                                          |
  A  P  T  A  R  K                                                        144

Intron 6

Exon 7
 ACCACAACTCGACGGCCCAAG                                               83153442
  T  T  T  R  R  P  K                                                       |
  |  |  |  |  |  |  |                                                       |
  T  T  T  R  R  P  K                                                     151

Intron 7

Exon 8
 CCTACTCGCCCAGCCAGCACTGGGGTGGCTGGGCCCAGTAGCTCCCTTGGCCCCTCTGGCTCAGCGTCAGCC   83154806
  P  T  R  P  A  S  T  G  V  A  G  P  S  S  S  L  G  P  S  G  S  A  S  A          |
  |  |  |  |  |  |  |  |  |  |  |  |  |  |  |  |  |  |  |  |  |  |  |  |          |
  P  T  R  P  A  S  T  G  V  A  G  P  S  S  S  L  G  P  S  G  S  A  S  A        175

 GGGGAACTAAGCAGCAGTGAGCCCAGCACCCCAGCTCAGACTCCGCTGGCAGCACCCATCATCCCCACACCG   83154878
  G  E  L  S  S  S  E  P  S  T  P  A  Q  T  P  L  A  A  P  I  I  P  T  P          |
  |  |  |  |  |  |  |  |  |  |  |  |  |  |  |  |  |  |  |  |  |  |  |  |          |
  G  E  L  S  S  S  E  P  S  T  P  A  Q  T  P  L  A  A  P  I  I  P  T  P        199
```

Figure 10: DNA-protein alignment of the 5’-end of the dynactin p150 gene of *Mus musculus* reconstructed with Scipio v1.5. The three small exons were correctly identified and the surrounding introns start and end with the most common splice sites.

Parameters to account for reconstructing short exons at low homology intron borders

Low homology at intron borders is in most cases only a problem for introns next to very short exons. If these short exons are not recognized by Blat, there is often a good chance that there is at least some remaining homology between the sequence of the short exon and the sequence at the intron end. Blat then extends the following exon into the intron region even by introducing one or more mismatches. In Scipio v1.0 these cases could not be resolved. Even with just using the Needleman-Wunsch implementation those cases would not be reconstructed because the remaining unmatched part of the short exons are becoming even shorter and there are no known splice sites at the potential "intron" borders. Therefore, we introduced the parameter --max_move_exon that gives the value of how many residues will be cut from the neighboring exons that will be subsequently added to the remaining part of the exon to search.

Example A

This example shows part of the reconstruction of the class-19 myosin from *Homo sapiens*. The N-terminal exon consists of the amino acids MLQQ. The first intron ends with translated amino acids LFQ that are quite homologous to the query sequence and therefore identified by Blat. Adjusting the --max_move_exon parameter to 3 in Scipio v1.5 resolved the correct terminal exon.

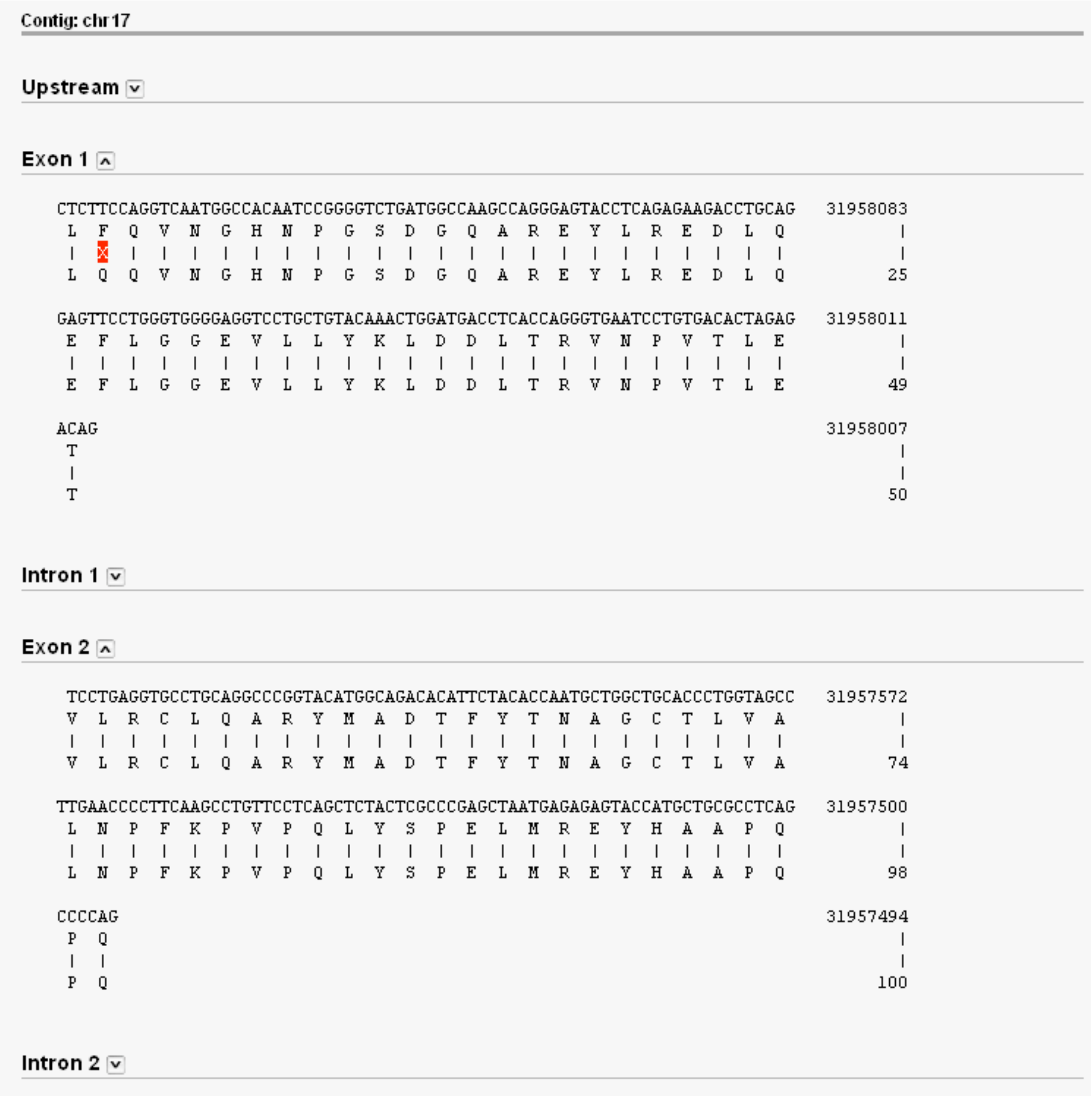

Figure 11: Section of the output generated by Scipio v1.0. The first exon of the gene could not be reconstructed because Blat recognized some homology at the intron end that, however, would have resulted in strange intron splice sites if Scipio had tried to identify the N-terminal methionine.

```
Contig: chr17

Upstream

Exon 1

  ATGCTCCAGCAG                                                             31962469
   M  L  Q  Q                                                                     |
   |  |  |  |                                                                     |
   M  L  Q  Q                                                                     4

Intron 1

Exon 2

  GTCAATGGCCACAATCCGGGGTCTGATGGCCAAGCCAGGGAGTACCTCAGAGAAGACCTGCAGGAGTTCCTG   31958074
   V  N  G  H  N  P  G  S  D  G  Q  A  R  E  Y  L  R  E  D  L  Q  E  F  L          |
   |  |  |  |  |  |  |  |  |  |  |  |  |  |  |  |  |  |  |  |  |  |  |  |          |
   V  N  G  H  N  P  G  S  D  G  Q  A  R  E  Y  L  R  E  D  L  Q  E  F  L         28

  GGTGGGGAGGTCCTGCTGTACAAACTGGATGACCTCACCAGGGTGAATCCTGTGACACTAGAGACAG        31958007
   G  G  E  V  L  L  Y  K  L  D  D  L  T  R  V  N  P  V  T  L  E  T                |
   |  |  |  |  |  |  |  |  |  |  |  |  |  |  |  |  |  |  |  |  |  |                |
   G  G  E  V  L  L  Y  K  L  D  D  L  T  R  V  N  P  V  T  L  E  T               50

Intron 2

Exon 3

   TCCTGAGGTGCCTGCAGGCCCGGTACATGGCAGACACATTCTACACCAATGCTGGCTGCACCCTGGTAGCC  31957572
   V  L  R  C  L  Q  A  R  Y  M  A  D  T  F  Y  T  N  A  G  C  T  L  V  A          |
   |  |  |  |  |  |  |  |  |  |  |  |  |  |  |  |  |  |  |  |  |  |  |  |          |
   V  L  R  C  L  Q  A  R  Y  M  A  D  T  F  Y  T  N  A  G  C  T  L  V  A         74

  TTGAACCCCTTCAAGCCTGTTCCTCAGCTCTACTCGCCCGAGCTAATGAGAGAGTACCATGCTGCGCCTCAG   31957500
   L  N  P  F  K  P  V  P  Q  L  Y  S  P  E  L  M  R  E  Y  H  A  A  P  Q          |
   |  |  |  |  |  |  |  |  |  |  |  |  |  |  |  |  |  |  |  |  |  |  |  |          |
   L  N  P  F  K  P  V  P  Q  L  Y  S  P  E  L  M  R  E  Y  H  A  A  P  Q         98

  CCCCAG                                                                   31957494
   P  Q                                                                           |
   |  |                                                                           |
   P  Q                                                                         100
```

Figure 12: Section of the output generated by Scipio v1.5. With the --max_move_exon parameter set to 3, Scipio is able to identify the N-terminal exon and correctly reconstruct the class-19 myosin gene.

Example B

This example shows a case of an even longer stretch of sequence homology (compared to example A) this time at the end of an internal intron. The reconstruction of the actin capping protein α from *Theileria heterothallica* showed strong homology between the 6 aa exon 2 and the end of intron 2. Scipio v1.0 therefore added this exon to the 5' end of the following exon although this lead to three mismatches and an intron with uncommon intron splice sites. Setting the --max_move_exon parameter to 6 in Scipio v1.5 released the 6 misplaced residues from exon 3 and allowed Scipio to correctly reconstruct exon 2 using the Needleman-Wunsch algorithm.

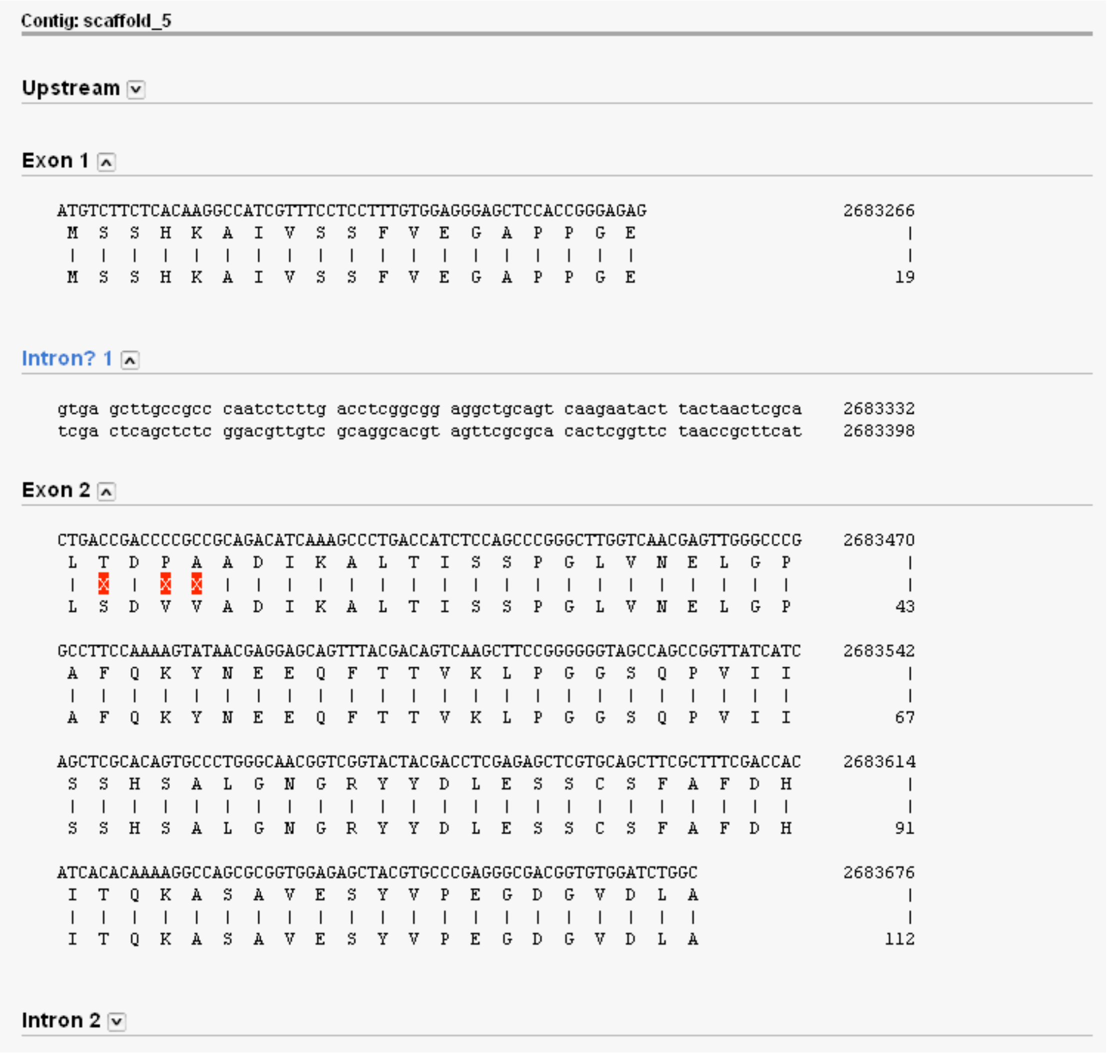

Figure 13: Section of the output generated by Scipio v1.0. The second exon of the gene could not be reconstructed because Blat recognized some homology at the intron end that, however, resulted in three mismatches and a strange intron splice site.

```
Upstream

Exon 1

ATGTCTTCTCACAAGGCCATCGTTTCCTCCTTTGTGGAGGGAGCTCCACCGGGAGAG          2683266
 M  S  S  H  K  A  I  V  S  S  F  V  E  G  A  P  P  G  E                 |
 |  |  |  |  |  |  |  |  |  |  |  |  |  |  |  |  |  |  |                 |
 M  S  S  H  K  A  I  V  S  S  F  V  E  G  A  P  P  G  E                19

Intron 1

gtga gcttgccgcc caatctcttg acctcggcgg aggctgcagt caagaatact tactaactcgca    2683332
tcga ctcag                                                                  2683341

Exon 2

CTCTCGGACGTTGTCGCAG                                                2683360
 L  S  D  V  V  A                                                        |
 |  |  |  |  |  |                                                        |
 L  S  D  V  V  A                                                       25

Intron 2

gcacgtagtt cgcgcacact cggttctaac cgcttcatct gaccgacccc gccgcag      2683417

Exon 3

 ACATCAAAGCCCTGACCATCTCCAGCCCGGGCTTGGTCAACGAGTTGGGCCCGGCCTTCCAAAAGTATAAC    2683488
 D  I  K  A  L  T  I  S  S  P  G  L  V  N  E  L  G  P  A  F  Q  K  Y  N        |
 |  |  |  |  |  |  |  |  |  |  |  |  |  |  |  |  |  |  |  |  |  |  |  |        |
 D  I  K  A  L  T  I  S  S  P  G  L  V  N  E  L  G  P  A  F  Q  K  Y  N       49

GAGGAGCAGTTTACGACAGTCAAGCTTCCGGGGGGTAGCCAGCCGGTTATCATCAGCTCGCACAGTGCCCTG    2683560
 E  E  Q  F  T  T  V  K  L  P  G  G  S  Q  P  V  I  I  S  S  H  S  A  L        |
 |  |  |  |  |  |  |  |  |  |  |  |  |  |  |  |  |  |  |  |  |  |  |  |        |
 E  E  Q  F  T  T  V  K  L  P  G  G  S  Q  P  V  I  I  S  S  H  S  A  L       73

GGCAACGGTCGGTACTACGACCTCGAGAGCTCGTGCAGCTTCGCTTTCGACCACATCACACAAAAGGCCAGC    2683632
 G  N  G  R  Y  Y  D  L  E  S  S  C  S  F  A  F  D  H  I  T  Q  K  A  S        |
 |  |  |  |  |  |  |  |  |  |  |  |  |  |  |  |  |  |  |  |  |  |  |  |        |
 G  N  G  R  Y  Y  D  L  E  S  S  C  S  F  A  F  D  H  I  T  Q  K  A  S       97

GCGGTGGAGAGCTACGTGCCCGAGGGCGACGGTGTGGATCTGGC                                2683676
 A  V  E  S  Y  V  P  E  G  D  G  V  D  L  A                                     |
 |  |  |  |  |  |  |  |  |  |  |  |  |  |  |                                     |
 A  V  E  S  Y  V  P  E  G  D  G  V  D  L  A                                   112
```

Figure 14: Section of the output generated by Scipio v1.5. With the --max_move_exon parameter set to 6, Scipio is able to correctly identify and reconstruct exon 2.
