# Supplementary material for: Cross-species protein sequence and gene structure prediction with fine-tuned Webscipio 2.0 and Scipio
Source: BMC Res Notes. 2011 Jul 28;4:265. doi: 10.1186/1756-0500-4-265 (PMC3162530; doi:10.1186/1756-0500-4-265)
Supplement: Additional file 3 — Table with detailed data of the results of the cross-species search of the human DHC genes in the elephant genome. The table provides detailed data to the cross-species searches including numbers of matches and mismatches, gaps and intron?'s, for the searches with different parameters. [file 1756-0500-4-265-S3.PDF]

Reference

Protein	Matches	Mismatches	Intron?s
DHC1	4561		
DHC2	4253	6	3
DHC3A	4690		
DHC3B	4581		1
DHC4A	4507		
DHC4B	4413	4	2
DHC4C	4323	9	5
DHC5	4584		
DHC6	4457		
DHC7A	4018		
DHC7B	3962	3	1
DHC7C	3960		
DHC8	4059	1	4
DHC9A	4062		1
DHC9B	4597		1
DHC11	4779		

Scipio 1.0

Protein	Matches	Mismatches	Covered	Gaps	Intron?s
DHC1	4528	43	4571	0	0
DHC2	4027	206	4233	2	3
DHC3A	4346	248	4594	3	1
DHC3B	4098	345	4443	6	3
DHC4A	3824	444	4268	5	3
DHC4B	3959	321	4280	6	2
DHC4C	3799	380	4179	7	1
DHC5	4035	343	4378	3	3
DHC6	4024	288	4312	5	0
DHC7A	3637	283	3920	6	0
DHC7B	3418	359	3777	8	0
DHC7C	3535	283	3818	2	0
DHC8	3486	300	3786	13	2
DHC9A	3566	285	3851	6	4
DHC9B	2630	618	3248	27	4
DHC11	3350	841	4191	16	2

s1: Scipio 1.5

blat_tilesize	7
exhaust_align_size	500
exhaust_gap_size	21

Protein	Matches	Mismatches	Covered	In addition	Gaps	Intron?s
DHC1	4518	43	4561	-10	1	0
DHC2	4024	210	4234	1	2	0
DHC3A	4355	249	4604	10	2	1
DHC3B	4179	393	4572	129	2	1
DHC4A	3993	513	4506	238	0	0
DHC4B	4051	341	4392	112	2	1
DHC4C	3894	422	4316	137	6	1
DHC5	4151	388	4539	161	1	1
DHC6	4072	299	4371	59	2	0
DHC7A	3706	304	4010	90	3	0
DHC7B	3521	396	3917	140	4	0
DHC7C	3639	312	3951	133	1	0
DHC8	3649	348	3997	211	6	3
DHC9A	3649	322	3971	120	6	1
DHC9B	3284	884	4168	920	16	1
DHC11	3643	970	4613	422	7	2

s2: Scipio 1.5

blat_tilesize	6
exhaust_align_size	500
exhaust_gap_size	18

Protein	Matches	Mismatches	Covered	In addition	Gaps	Intron?s
DHC1	4518	43	4561	-10	1	0
DHC2	4029	211	4240	7	2	0
DHC3A	4351	248	4599	5	2	1
DHC3B	4179	393	4572	129	2	1
DHC4A	3992	514	4506	238	0	0
DHC4B	4064	342	4406	126	2	1
DHC4C	3894	422	4316	137	6	1
DHC5	4151	388	4539	161	1	1
DHC6	4072	299	4371	59	2	0
DHC7A	3706	304	4010	90	3	0
DHC7B	3521	396	3917	140	4	0
DHC7C	3639	312	3951	133	1	0
DHC8	3661	356	4017	231	5	5
DHC9A	3641	319	3960	109	6	0
DHC9B	3396	946	4342	1094	12	2
DHC11	3651	981	4632	441	6	2

s3: Scipio 1.5

blat_tilesize	7
exhaust_align_size	5000
exhaust_gap_size	21

Protein	Matches	Mismatches	Covered	In addition	Gaps	Intron?s
DHC1	4518	43	4561	-10	1	0
DHC2	4024	210	4234	1	2	0
DHC3A	4364	251	4615	21	1	1
DHC3B	4181	397	4578	135	1	0
DHC4A	3993	513	4506	238	0	0
DHC4B	4061	343	4404	124	1	1
DHC4C	3897	427	4324	145	4	2
DHC5	4153	385	4538	160	1	1
DHC6	4072	299	4371	59	2	0
DHC7A	3708	309	4017	97	2	0
DHC7B	3522	403	3925	148	3	0
DHC7C	3639	312	3951	133	1	0
DHC8	3654	352	4006	220	5	1
DHC9A	3649	327	3976	125	5	1
DHC9B	3288	895	4183	935	14	2
DHC11	3645	967	4612	421	7	1

s4: Scipio 1.5

blat_tilesize	6
exhaust_align_size	5000
exhaust_gap_size	25

Protein	Matches	Mismatches	Covered	In addition	Gaps	Intron?s
DHC1	4518	43	4561	-10	1	0
DHC2	4038	214	4252	19	1	1
DHC3A	4351	248	4599	5	2	1
DHC3B	4181	397	4578	135	1	0
DHC4A	3992	514	4506	238	0	0
DHC4B	4072	346	4418	138	1	0
DHC4C	3897	426	4323	144	4	2
DHC5	4153	385	4538	160	1	1
DHC6	4072	299	4371	59	2	0
DHC7A	3708	308	4016	96	1	0
DHC7B	3522	402	3924	147	3	0
DHC7C	3639	312	3951	133	1	0
DHC8	3663	354	4017	231	5	3
DHC9A	3647	341	3988	137	4	0
DHC9B	3413	981	4394	1146	8	2
DHC11	3658	977	4635	444	6	2
