# Supplementary material for: Cross-species protein sequence and gene structure prediction with fine-tuned Webscipio 2.0 and Scipio
Source: BMC Res Notes. 2011 Jul 28;4:265. doi: 10.1186/1756-0500-4-265 (PMC3162530; doi:10.1186/1756-0500-4-265)
Supplement: Additional file 4 — Detailed evaluation values used for Tables 2, 3, and 4. This file provides a description of each evaluation parameter and the values obtained with each software tool for all sequence predictions. The values highlighted in yellow were used for Tables 2, 3, and 4. [file 1756-0500-4-265-S4.PDF]

## Detailed evaluation values used for Tables 2-4

The evaluation values were calculated based on the GFF (General Feature Format) output of a tool or the conversation of the output to the GFF format. The GFF format gives each exon start and end positions. Only coding exons were used for evaluation. The “DHC all” lines summarize the results of the search for dynein heavy chain genes in *Loxodonta africana* and the lines marked as “others” summarize the difficult cases. Tables 2 and 3 show the values of “DHC all” marked in yellow. For runs, which did not give any results, the lines are left blank, e.g. “Prosplign (Blat)”. Additional File 5 describes the versions and parameters of the tools.

**Exon PE Predicted Exons**

This is the number of exons predicted by the tool.

**Exon AE Annotated Exons**

This is the number of exons annotated in the reference.

**Exon TE True Exons**

This is the number of predicted exons, which have exactly the same exon borders as the annotated exons.

**Exon OE Overlapped Exons**

This is the number of annotated exons, which are overlapped by at least one predicted exon.

**Exon ME Missing Exons**

This is the number of annotated exons, which are not overlapped by any predicted exons.

**Exon WE Wrong Exons**

This is the number of predicted exons, which are not overlapped by any annotated exons.

**Exon Sn Exon Sensitivity = TE / AE**

This is the number of true exons divided by the number of annotated exons.

**Exon Sno Exon Overlapped Sensitivity = OE / AE**

This is the number of overlapped exons divided by the number of annotated exons.

**Exon Sp Exon Specificity = TE / PE**

This is the number of true exons divided by the number of predicted exons.

**Nucl Total Total Number**

This is the total number of nucleotides in the genomic sequence.

**Nucl AP Actual Positive**

This is the number of nucleotides, which are annotated as coding sequence (belonging to a coding exon) in the reference.

**Nucl AN Actual Negative**

This is the number of nucleotides, which are annotated as non-coding sequence (not belonging to a coding exon) in the reference.

**Nucl PP Predicted Positive**

This is the number of nucleotides, which are predicted as coding sequence (belonging to a coding exon) by the tool.

**Nucl PN Predicted Negative**

This is the number of nucleotides, which are predicted as non-coding sequence (not belonging to a coding exon) by the tool.

**Nucl TP True Positive**

This is the number of predicted positivs, which are annotated positive, too.

**Nucl FP False Positive**

This is the number of predicted positives, which are annotated negative.

**Nucl TN True Negative**

This is the number of predicted negatives, which are annotated negative, too.

**Nucl FN False Negative**

This is the number of predicted negatives, which are annotated positive.

**Nucl Sn Sensitivity = TP / AP**

This is the number of true positives divided by actual positives.

**Nucl Sp Specificity = TP / PP**

This is the number of true positives divided by predicted positives.

**Nucl AC Approximate Correlation**

Approximate Correlation = ((TP/(TP+FN)) + (TP/(TP+FP)) + (TN/(TN+FP)) + (TN/(TN+FN))) / 2 - 1

## Scenarios 1 and 3 (whole genome)

### Scipio v1.0

| | Exon PE | Exon AE | Exon TE | Exon OE | Exon ME | Exon WE | Exon Sn | Exon Sno | Exon Sp | Nucl Total | Nucl AP | Nucl AN | Nucl PP | Nucl PN | Nucl TP | Nucl FP | Nucl TN | Nucl FN | Nucl Sn | Nucl Sp | Nucl AC | Genes | Duration in s |
|---|---|---|---|---|---|---|---|---|---|---|---|---|---|---|---|---|---|---|---|---|---|---|---|
| **DHC all** | **1244** | **1202** | **1035** | **1188** | **14** | **46** | **0.861** | **0.988** | **0.832** | **52348687472** | **209486** | **52348477986** | **206320** | **52348481152** | **205147** | **1173** | **52348476813** | **4339** | **0.979** | **0.994** | **0.987** | **16** | **504** |
| **DHC1** | 86 | 77 | 76 | 77 | 0 | 9 | 0.987 | 1.000 | 0.884 | 3271792967 | 13686 | 3271779281 | 13893 | 3271779074 | 13680 | 213 | 3271779068 | 6 | 1.000 | 0.985 | 0.992 | 1 | 484 |
| **DHC2** | 90 | 88 | 83 | 88 | 0 | 2 | 0.943 | 1.000 | 0.922 | 3271792967 | 12702 | 3271780265 | 12702 | 3271780265 | 12678 | 24 | 3271780241 | 24 | 0.998 | 0.998 | 0.998 | 1 | 549 |
| **DHC3A** | 99 | 92 | 88 | 92 | 0 | 7 | 0.957 | 1.000 | 0.889 | 3271792967 | 14070 | 3271778897 | 13977 | 3271778990 | 13797 | 180 | 3271778717 | 273 | 0.981 | 0.987 | 0.984 | 1 | 506 |
| **DHC3B** | 79 | 78 | 67 | 78 | 0 | 1 | 0.859 | 1.000 | 0.848 | 3271792967 | 13745 | 3271779222 | 13639 | 3271779328 | 13601 | 38 | 3271779184 | 144 | 0.990 | 0.997 | 0.993 | 1 | 487 |
| **DHC4A** | 83 | 82 | 74 | 82 | 0 | 1 | 0.902 | 1.000 | 0.892 | 3271792967 | 13523 | 3271779444 | 13500 | 3271779467 | 13481 | 19 | 3271779425 | 42 | 0.997 | 0.999 | 0.998 | 1 | 504 |
| **DHC4B** | 79 | 79 | 70 | 79 | 0 | 0 | 0.886 | 1.000 | 0.886 | 3271792967 | 13284 | 3271779683 | 13122 | 3271779845 | 13099 | 23 | 3271779660 | 185 | 0.986 | 0.998 | 0.992 | 1 | 470 |
| **DHC4C** | 74 | 68 | 61 | 68 | 0 | 6 | 0.897 | 1.000 | 0.824 | 3271792967 | 13014 | 3271779953 | 13004 | 3271779963 | 12875 | 129 | 3271779824 | 139 | 0.989 | 0.990 | 0.990 | 1 | 517 |
| **DHC5** | 79 | 79 | 71 | 78 | 1 | 1 | 0.899 | 0.987 | 0.899 | 3271792967 | 13752 | 3271779215 | 13617 | 3271779350 | 13585 | 32 | 3271779183 | 167 | 0.988 | 0.998 | 0.993 | 1 | 492 |
| **DHC6** | 85 | 86 | 81 | 85 | 1 | 0 | 0.942 | 0.988 | 0.953 | 3271792967 | 13371 | 3271779596 | 13101 | 3271779866 | 13100 | 1 | 3271779595 | 271 | 0.980 | 1.000 | 0.990 | 1 | 512 |
| **DHC7A** | 63 | 64 | 58 | 63 | 1 | 0 | 0.906 | 0.984 | 0.921 | 3271792967 | 12058 | 3271780909 | 11944 | 3271781023 | 11934 | 10 | 3271780899 | 124 | 0.990 | 0.999 | 0.994 | 1 | 458 |
| **DHC7B** | 61 | 60 | 51 | 59 | 1 | 2 | 0.850 | 0.983 | 0.836 | 3271792967 | 11897 | 3271781070 | 11769 | 3271781198 | 11729 | 40 | 3271781030 | 168 | 0.986 | 0.997 | 0.991 | 1 | 516 |
| **DHC7C** | 72 | 73 | 70 | 72 | 1 | 0 | 0.959 | 0.986 | 0.972 | 3271792967 | 11880 | 3271781087 | 11853 | 3271781114 | 11849 | 4 | 3271781083 | 31 | 0.997 | 1.000 | 0.999 | 1 | 472 |
| **DHC8** | 77 | 73 | 58 | 73 | 0 | 4 | 0.795 | 1.000 | 0.753 | 3271792967 | 12190 | 3271780777 | 12082 | 3271780885 | 11919 | 163 | 3271780614 | 271 | 0.978 | 0.987 | 0.982 | 1 | 507 |
| **DHC9A** | 78 | 75 | 59 | 75 | 0 | 2 | 0.787 | 1.000 | 0.756 | 3271792967 | 12186 | 3271780781 | 11937 | 3271781030 | 11875 | 62 | 3271780719 | 311 | 0.974 | 0.995 | 0.985 | 1 | 486 |
| **DHC9B** | 89 | 85 | 44 | 79 | 6 | 7 | 0.518 | 0.929 | 0.494 | 3271792967 | 13791 | 3271779176 | 12563 | 3271780404 | 12408 | 155 | 3271779021 | 1383 | 0.900 | 0.988 | 0.944 | 1 | 547 |
| **DHC11** | 50 | 43 | 24 | 40 | 3 | 4 | 0.558 | 0.930 | 0.480 | 3271792967 | 14337 | 3271778630 | 13617 | 3271779350 | 13537 | 80 | 3271778550 | 800 | 0.944 | 0.994 | 0.969 | 1 | 560 |
| **Others** | **89** | **93** | **77** | **85** | **8** | **1** | **0.828** | **0.914** | **0.865** | **9014070513** | **14186** | **9014056327** | **14020** | **9014056493** | **13969** | **51** | **9014056276** | **217** | **0.985** | **0.996** | **0.991** | **7** | **412** |
| **NedKinesinJ_fl** | 5 | 3 | 2 | 3 | 0 | 0 | 0.667 | 1.000 | 0.400 | 37302679 | 2273 | 37300406 | 2243 | 37300436 | 2243 | 0 | 37300406 | 30 | 0.987 | 1.000 | 0.993 | 1 | 520 |
| **PhsP62_fl** | 4 | 3 | 0 | 3 | 0 | 0 | 0.000 | 1.000 | 0.000 | 77806163 | 1506 | 77804657 | 1428 | 77804735 | 1428 | 0 | 77804657 | 78 | 0.948 | 1.000 | 0.974 | 1 | 1131 |
| **HsP50_fl** | 14 | 16 | 14 | 14 | 2 | 0 | 0.875 | 0.875 | 1.000 | 3095677409 | 1218 | 3095676191 | 1203 | 3095676206 | 1203 | 0 | 3095676191 | 15 | 0.988 | 1.000 | 0.994 | 1 | 434 |
| **PugCor_fl** | 10 | 10 | 9 | 9 | 1 | 1 | 0.900 | 0.900 | 0.900 | 88644628 | 1617 | 88643011 | 1617 | 88643011 | 1608 | 9 | 88643002 | 9 | 0.994 | 0.994 | 0.994 | 1 | 11 |
| **MmP150_fl** | 29 | 32 | 27 | 29 | 3 | 0 | 0.844 | 0.906 | 0.931 | 2580223614 | 3843 | 2580219771 | 3803 | 2580219811 | 3789 | 14 | 2580219757 | 54 | 0.986 | 0.996 | 0.991 | 1 | 350 |
| **HsMyo19_fl** | 23 | 24 | 22 | 23 | 1 | 0 | 0.917 | 0.958 | 0.957 | 3095677409 | 2910 | 3095674499 | 2907 | 3095674502 | 2898 | 9 | 3095674490 | 12 | 0.996 | 0.997 | 0.996 | 1 | 436 |
| **ThCAP1_fl** | 4 | 5 | 3 | 4 | 1 | 0 | 0.600 | 0.800 | 0.750 | 38738611 | 819 | 38737792 | 819 | 38737792 | 800 | 19 | 38737773 | 19 | 0.977 | 0.977 | 0.977 | 1 | 5 |

### Scipio v1.5

| | Exon PE | Exon AE | Exon TE | Exon OE | Exon ME | Exon WE | Exon Sn | Exon Sno | Exon Sp | Nucl Total | Nucl AP | Nucl AN | Nucl PP | Nucl PN | Nucl TP | Nucl FP | Nucl TN | Nucl FN | Nucl Sn | Nucl Sp | Nucl AC | Genes | Duration in s |
|---|---|---|---|---|---|---|---|---|---|---|---|---|---|---|---|---|---|---|---|---|---|---|---|
| **DHC all** | **1442** | **1202** | **1123** | **1191** | **11** | **244** | **0.934** | **0.991** | **0.779** | **52348687472** | **209483** | **52348477989** | **244678** | **52348442794** | **206793** | **37885** | **52348440104** | **2690** | **0.987** | **0.845** | **0.916** | **20** | **4157** |
| **DHC1** | 77 | 77 | 77 | 77 | 0 | 0 | 1.000 | 1.000 | 1.000 | 3271792967 | 13683 | 3271779284 | 13683 | 3271779284 | 13683 | 0 | 3271779284 | 0 | 1.000 | 1.000 | 1.000 | 1 | 3078 |
| **DHC2** | 89 | 88 | 86 | 88 | 0 | 1 | 0.977 | 1.000 | 0.966 | 3271792967 | 12702 | 3271780265 | 12780 | 3271780187 | 12702 | 78 | 3271780187 | 0 | 1.000 | 0.994 | 0.997 | 1 | 4081 |
| **DHC3A** | 151 | 92 | 91 | 92 | 0 | 59 | 0.989 | 1.000 | 0.603 | 3271792967 | 14070 | 3271778897 | 23300 | 3271769667 | 13854 | 9446 | 3271769451 | 216 | 0.985 | 0.595 | 0.790 | 2 | 4346 |
| **DHC3B** | 141 | 78 | 74 | 78 | 0 | 63 | 0.949 | 1.000 | 0.525 | 3271792967 | 13745 | 3271779222 | 22787 | 3271770180 | 13735 | 9052 | 3271770170 | 10 | 0.999 | 0.603 | 0.801 | 2 | 4225 |
| **DHC4A** | 82 | 82 | 80 | 82 | 0 | 0 | 0.976 | 1.000 | 0.976 | 3271792967 | 13523 | 3271779444 | 13523 | 3271779444 | 13519 | 4 | 3271779440 | 4 | 1.000 | 1.000 | 1.000 | 1 | 3201 |
| **DHC4B** | 135 | 79 | 75 | 79 | 0 | 56 | 0.949 | 1.000 | 0.556 | 3271792967 | 13284 | 3271779683 | 22744 | 3271770223 | 13198 | 9546 | 3271770137 | 86 | 0.994 | 0.580 | 0.787 | 2 | 3031 |
| **DHC4C** | 130 | 68 | 63 | 68 | 0 | 62 | 0.926 | 1.000 | 0.485 | 3271792967 | 13014 | 3271779953 | 22584 | 3271770383 | 13014 | 9570 | 3271770383 | 0 | 1.000 | 0.576 | 0.788 | 2 | 3959 |
| **DHC5** | 78 | 79 | 75 | 78 | 1 | 0 | 0.949 | 0.987 | 0.962 | 3271792967 | 13752 | 3271779215 | 13617 | 3271779350 | 13603 | 14 | 3271779201 | 149 | 0.989 | 0.999 | 0.994 | 1 | 2688 |
| **DHC6** | 85 | 86 | 83 | 85 | 1 | 0 | 0.965 | 0.988 | 0.976 | 3271792967 | 13371 | 3271779596 | 13113 | 3271779854 | 13113 | 0 | 3271779596 | 258 | 0.981 | 1.000 | 0.990 | 1 | 3021 |
| **DHC7A** | 64 | 64 | 63 | 64 | 0 | 0 | 0.984 | 1.000 | 0.984 | 3271792967 | 12058 | 3271780909 | 12064 | 3271780903 | 12058 | 6 | 3271780903 | 0 | 1.000 | 1.000 | 1.000 | 1 | 5225 |
| **DHC7B** | 59 | 60 | 55 | 59 | 1 | 0 | 0.917 | 0.983 | 0.932 | 3271792967 | 11897 | 3271781070 | 11775 | 3271781192 | 11772 | 3 | 3271781067 | 125 | 0.989 | 1.000 | 0.995 | 1 | 3696 |
| **DHC7C** | 73 | 73 | 71 | 73 | 0 | 0 | 0.973 | 1.000 | 0.973 | 3271792967 | 11880 | 3271781087 | 11880 | 3271781087 | 11876 | 4 | 3271781083 | 4 | 1.000 | 1.000 | 1.000 | 1 | 3519 |
| **DHC8** | 75 | 73 | 65 | 73 | 0 | 2 | 0.890 | 1.000 | 0.867 | 3271792967 | 12190 | 3271780777 | 12068 | 3271780899 | 11998 | 70 | 3271780707 | 192 | 0.984 | 0.994 | 0.989 | 1 | 4653 |
| **DHC9A** | 75 | 75 | 68 | 75 | 0 | 0 | 0.907 | 1.000 | 0.907 | 3271792967 | 12186 | 3271780781 | 12078 | 3271780889 | 12058 | 20 | 3271780761 | 128 | 0.989 | 0.998 | 0.994 | 1 | 3437 |
| **DHC9B** | 83 | 85 | 65 | 80 | 5 | 1 | 0.765 | 0.941 | 0.783 | 3271792967 | 13791 | 3271779176 | 12960 | 3271780007 | 12898 | 62 | 3271779114 | 893 | 0.935 | 0.995 | 0.965 | 1 | 5625 |
| **DHC11** | 45 | 43 | 32 | 40 | 3 | 0 | 0.744 | 0.930 | 0.711 | 3271792967 | 14337 | 3271778630 | 13722 | 3271779245 | 13712 | 10 | 3271778620 | 625 | 0.956 | 0.999 | 0.978 | 1 | 8735 |
| **Others** | **4237** | **93** | **93** | **93** | **0** | **4140** | **1.000** | **1.000** | **0.022** | **9014070513** | **14186** | **9014056327** | **95767** | **9013974746** | **14186** | **81581** | **9013974746** | **0** | **1.000** | **0.148** | **0.574** | **164** | **12188** |
| **NedKinesinJ_fl** | 3345 | 3 | 3 | 3 | 0 | 3338 | 1.000 | 1.000 | 0.001 | 37302679 | 2273 | 37300406 | 68755 | 37233924 | 2273 | 66482 | 37233924 | 0 | 1.000 | 0.033 | 0.516 | 43 | 46054 |
| **PhsP62_fl** | 805 | 3 | 3 | 3 | 0 | 802 | 1.000 | 1.000 | 0.004 | 77806163 | 1506 | 77804657 | 16605 | 77789558 | 1506 | 15099 | 77789558 | 0 | 1.000 | 0.091 | 0.545 | 116 | 37847 |
| **HsP50_fl** | 16 | 16 | 16 | 16 | 0 | 0 | 1.000 | 1.000 | 1.000 | 3095677409 | 1218 | 3095676191 | 1218 | 3095676191 | 1218 | 0 | 3095676191 | 0 | 1.000 | 1.000 | 1.000 | 1 | 496 |
| **PugCor_fl** | 10 | 10 | 10 | 10 | 0 | 0 | 1.000 | 1.000 | 1.000 | 88644628 | 1617 | 88643011 | 1617 | 88643011 | 1617 | 0 | 88643011 | 0 | 1.000 | 1.000 | 1.000 | 1 | 13 |
| **MmP150_fl** | 32 | 32 | 32 | 32 | 0 | 0 | 1.000 | 1.000 | 1.000 | 2580223614 | 3843 | 2580219771 | 3843 | 2580219771 | 3843 | 0 | 2580219771 | 0 | 1.000 | 1.000 | 1.000 | 1 | 384 |
| **HsMyo19_fl** | 24 | 24 | 24 | 24 | 0 | 0 | 1.000 | 1.000 | 1.000 | 3095677409 | 2910 | 3095674499 | 2910 | 3095674499 | 2910 | 0 | 3095674499 | 0 | 1.000 | 1.000 | 1.000 | 1 | 520 |
| **ThCAP1_fl** | 5 | 5 | 5 | 5 | 0 | 0 | 1.000 | 1.000 | 1.000 | 38738611 | 819 | 38737792 | 819 | 38737792 | 819 | 0 | 38737792 | 0 | 1.000 | 1.000 | 1.000 | 1 | 6 |

### Scipio v1.5 (1 gene)

| | Exon PE | Exon AE | Exon TE | Exon OE | Exon ME | Exon WE | Exon Sn | Exon Sno | Exon Sp | Nucl Total | Nucl AP | Nucl AN | Nucl PP | Nucl PN | Nucl TP | Nucl FP | Nucl TN | Nucl FN | Nucl Sn | Nucl Sp | Nucl AC | Genes | Duration in s |
|---|---|---|---|---|---|---|---|---|---|---|---|---|---|---|---|---|---|---|---|---|---|---|---|
| **DHC all** | **1204** | **1202** | **1123** | **1191** | **11** | **6** | **0.934** | **0.991** | **0.933** | **52348687472** | **209483** | **52348477989** | **207207** | **52348480265** | **206793** | **414** | **52348477575** | **2690** | **0.987** | **0.998** | **0.993** | **16** | **4182** |
| **DHC1** | 77 | 77 | 77 | 77 | 0 | 0 | 1.000 | 1.000 | 1.000 | 3271792967 | 13683 | 3271779284 | 13683 | 3271779284 | 13683 | 0 | 3271779284 | 0 | 1.000 | 1.000 | 1.000 | 1 | 3125 |
| **DHC2** | 89 | 88 | 86 | 88 | 0 | 1 | 0.977 | 1.000 | 0.966 | 3271792967 | 12702 | 3271780265 | 12780 | 3271780187 | 12702 | 78 | 3271780187 | 0 | 1.000 | 0.994 | 0.997 | 1 | 4056 |
| **DHC3A** | 92 | 92 | 91 | 92 | 0 | 0 | 0.989 | 1.000 | 0.989 | 3271792967 | 14070 | 3271778897 | 13854 | 3271779113 | 13854 | 0 | 3271778897 | 216 | 0.985 | 1.000 | 0.992 | 1 | 4162 |
| **DHC3B** | 78 | 78 | 74 | 78 | 0 | 0 | 0.949 | 1.000 | 0.949 | 3271792967 | 13745 | 3271779222 | 13748 | 3271779219 | 13735 | 13 | 3271779209 | 10 | 0.999 | 0.999 | 0.999 | 1 | 4222 |
| **DHC4A** | 82 | 82 | 80 | 82 | 0 | 0 | 0.976 | 1.000 | 0.976 | 3271792967 | 13523 | 3271779444 | 13523 | 3271779444 | 13519 | 4 | 3271779440 | 4 | 1.000 | 1.000 | 1.000 | 1 | 3248 |
| **DHC4B** | 79 | 79 | 75 | 79 | 0 | 0 | 0.949 | 1.000 | 0.949 | 3271792967 | 13284 | 3271779683 | 13223 | 3271779744 | 13198 | 25 | 3271779658 | 86 | 0.994 | 0.998 | 0.996 | 1 | 2974 |
| **DHC4C** | 70 | 68 | 63 | 68 | 0 | 2 | 0.926 | 1.000 | 0.900 | 3271792967 | 13014 | 3271779953 | 13119 | 3271779848 | 13014 | 105 | 3271779848 | 0 | 1.000 | 0.992 | 0.996 | 1 | 3887 |
| **DHC5** | 78 | 79 | 75 | 78 | 1 | 0 | 0.949 | 0.987 | 0.962 | 3271792967 | 13752 | 3271779215 | 13617 | 3271779350 | 13603 | 14 | 3271779201 | 149 | 0.989 | 0.999 | 0.994 | 1 | 2879 |
| **DHC6** | 85 | 86 | 83 | 85 | 1 | 0 | 0.965 | 0.988 | 0.976 | 3271792967 | 13371 | 3271779596 | 13113 | 3271779854 | 13113 | 0 | 3271779596 | 258 | 0.981 | 1.000 | 0.990 | 1 | 2964 |
| **DHC7A** | 64 | 64 | 63 | 64 | 0 | 0 | 0.984 | 1.000 | 0.984 | 3271792967 | 12058 | 3271780909 | 12064 | 3271780903 | 12058 | 6 | 3271780903 | 0 | 1.000 | 1.000 | 1.000 | 1 | 5184 |

| | | | | | | | | | | | | | | | | | | | | | | | |
|---|---|---|---|---|---|---|---|---|---|---|---|---|---|---|---|---|---|---|---|---|---|---|---|
| **DHC7B** | 59 | 60 | 55 | 59 | 1 | 0 | 0.917 | 0.983 | 0.932 | 3271792967 | 11897 | 3271781070 | 11775 | 3271781192 | 11772 | 3 | 3271781067 | 125 | 0.989 | 1.000 | 0.995 | 1 | 3769 |
| **DHC7C** | 73 | 73 | 71 | 73 | 0 | 0 | 0.973 | 1.000 | 0.973 | 3271792967 | 11880 | 3271781087 | 11880 | 3271781087 | 11876 | 4 | 3271781083 | 4 | 1.000 | 1.000 | 1.000 | 1 | 3612 |
| **DHC8** | 75 | 73 | 65 | 73 | 0 | 2 | 0.890 | 1.000 | 0.867 | 3271792967 | 12190 | 3271780777 | 12068 | 3271780899 | 11998 | 70 | 3271780707 | 192 | 0.984 | 0.994 | 0.989 | 1 | 4683 |
| **DHC9A** | 75 | 75 | 68 | 75 | 0 | 0 | 0.907 | 1.000 | 0.907 | 3271792967 | 12186 | 3271780781 | 12078 | 3271780889 | 12058 | 20 | 3271780761 | 128 | 0.989 | 0.998 | 0.994 | 1 | 3434 |
| **DHC9B** | 83 | 85 | 65 | 80 | 5 | 1 | 0.765 | 0.941 | 0.783 | 3271792967 | 13791 | 3271779176 | 12960 | 3271780007 | 12898 | 62 | 3271779114 | 893 | 0.935 | 0.995 | 0.965 | 1 | 5756 |
| **DHC11** | 45 | 43 | 32 | 40 | 3 | 0 | 0.744 | 0.930 | 0.711 | 3271792967 | 14337 | 3271778630 | 13722 | 3271779245 | 13712 | 10 | 3271778620 | 625 | 0.956 | 0.999 | 0.978 | 1 | 8957 |
| **Others** | **93** | **93** | **93** | **93** | **0** | **0** | **1.000** | **1.000** | **1.000** | **9014070513** | **14186** | **9014056327** | **14186** | **9014056327** | **14186** | **0** | **9014056327** | **0** | **1.000** | **1.000** | **1.000** | **7** | **12179** |
| **NedKinesinJ_fl** | 3 | 3 | 3 | 3 | 0 | 0 | 1.000 | 1.000 | 1.000 | 37302679 | 2273 | 37300406 | 2273 | 37300406 | 2273 | 0 | 37300406 | 0 | 1.000 | 1.000 | 1.000 | 1 | 45654 |
| **PhsP62_fl** | 3 | 3 | 3 | 3 | 0 | 0 | 1.000 | 1.000 | 1.000 | 77806163 | 1506 | 77804657 | 1506 | 77804657 | 1506 | 0 | 77804657 | 0 | 1.000 | 1.000 | 1.000 | 1 | 38190 |
| **HsP50_fl** | 16 | 16 | 16 | 16 | 0 | 0 | 1.000 | 1.000 | 1.000 | 3095677409 | 1218 | 3095676191 | 1218 | 3095676191 | 1218 | 0 | 3095676191 | 0 | 1.000 | 1.000 | 1.000 | 1 | 449 |
| **PugCor_fl** | 10 | 10 | 10 | 10 | 0 | 0 | 1.000 | 1.000 | 1.000 | 88644628 | 1617 | 88643011 | 1617 | 88643011 | 1617 | 0 | 88643011 | 0 | 1.000 | 1.000 | 1.000 | 1 | 13 |
| **MmP150_fl** | 32 | 32 | 32 | 32 | 0 | 0 | 1.000 | 1.000 | 1.000 | 2580223614 | 3843 | 2580219771 | 3843 | 2580219771 | 3843 | 0 | 2580219771 | 0 | 1.000 | 1.000 | 1.000 | 1 | 424 |
| **HsMyo19_fl** | 24 | 24 | 24 | 24 | 0 | 0 | 1.000 | 1.000 | 1.000 | 3095677409 | 2910 | 3095674499 | 2910 | 3095674499 | 2910 | 0 | 3095674499 | 0 | 1.000 | 1.000 | 1.000 | 1 | 517 |
| **ThCAP1_fl** | 5 | 5 | 5 | 5 | 0 | 0 | 1.000 | 1.000 | 1.000 | 38738611 | 819 | 38737792 | 819 | 38737792 | 819 | 0 | 38737792 | 0 | 1.000 | 1.000 | 1.000 | 1 | 6 |

**Prosplign**

| | Exon PE | Exon AE | Exon TE | Exon OE | Exon ME | Exon WE | Exon Sn | Exon Sno | Exon Sp | Nucl Total | Nucl AP | Nucl AN | Nucl PP | Nucl PN | Nucl TP | Nucl FP | Nucl TN | Nucl FN | Nucl Sn | Nucl Sp | Nucl AC | Genes | Duration in s |
|---|---|---|---|---|---|---|---|---|---|---|---|---|---|---|---|---|---|---|---|---|---|---|---|
| **DHC all** | **4729** | **1202** | **0** | **17** | **1185** | **4712** | **0.000** | **0.014** | **0.000** | **52348687472** | **209482** | **52348477990** | **216363** | **52348471109** | **585** | **215778** | **52348262212** | **208897** | **0.003** | **0.003** | **0.003** | **16** | **-** |
| **DHC1** | 275 | 77 | 0 | 0 | 77 | 275 | 0.000 | 0.000 | 0.000 | 3271792967 | 13682 | 3271779285 | 14112 | 3271778855 | 0 | 14112 | 3271765173 | 13682 | 0.000 | 0.000 | 0.000 | 1 | - |
| **DHC2** | 300 | 88 | 0 | 0 | 88 | 300 | 0.000 | 0.000 | 0.000 | 3271792967 | 12702 | 3271780265 | 13137 | 3271779830 | 0 | 13137 | 3271767128 | 12702 | 0.000 | 0.000 | 0.000 | 1 | - |
| **DHC3A** | 307 | 92 | 0 | 0 | 92 | 307 | 0.000 | 0.000 | 0.000 | 3271792967 | 14070 | 3271778897 | 14454 | 3271778513 | 0 | 14454 | 3271764443 | 14070 | 0.000 | 0.000 | 0.000 | 1 | - |
| **DHC3B** | 286 | 78 | 0 | 4 | 74 | 282 | 0.000 | 0.051 | 0.000 | 3271792967 | 13745 | 3271779222 | 14187 | 3271778780 | 120 | 14067 | 3271765155 | 13625 | 0.009 | 0.008 | 0.009 | 1 | - |
| **DHC4A** | 322 | 82 | 0 | 7 | 75 | 315 | 0.000 | 0.085 | 0.000 | 3271792967 | 13523 | 3271779444 | 13755 | 3271779212 | 137 | 13618 | 3271765826 | 13386 | 0.010 | 0.010 | 0.010 | 1 | - |
| **DHC4B** | 276 | 79 | 0 | 0 | 79 | 276 | 0.000 | 0.000 | 0.000 | 3271792967 | 13284 | 3271779683 | 13629 | 3271779338 | 0 | 13629 | 3271766054 | 13284 | 0.000 | 0.000 | 0.000 | 1 | - |
| **DHC4C** | 301 | 68 | 0 | 0 | 68 | 301 | 0.000 | 0.000 | 0.000 | 3271792967 | 13014 | 3271779953 | 13809 | 3271779158 | 0 | 13809 | 3271766144 | 13014 | 0.000 | 0.000 | 0.000 | 1 | - |
| **DHC5** | 309 | 79 | 0 | 0 | 79 | 309 | 0.000 | 0.000 | 0.000 | 3271792967 | 13752 | 3271779215 | 14070 | 3271778897 | 0 | 14070 | 3271765145 | 13752 | 0.000 | 0.000 | 0.000 | 1 | - |
| **DHC6** | 303 | 86 | 0 | 0 | 86 | 303 | 0.000 | 0.000 | 0.000 | 3271792967 | 13371 | 3271779596 | 13638 | 3271779329 | 0 | 13638 | 3271765958 | 13371 | 0.000 | 0.000 | 0.000 | 1 | - |
| **DHC7A** | 279 | 64 | 0 | 0 | 64 | 279 | 0.000 | 0.000 | 0.000 | 3271792967 | 12058 | 3271780909 | 12345 | 3271780622 | 0 | 12345 | 3271768564 | 12058 | 0.000 | 0.000 | 0.000 | 1 | - |
| **DHC7B** | 288 | 60 | 0 | 0 | 60 | 288 | 0.000 | 0.000 | 0.000 | 3271792967 | 11897 | 3271781070 | 12327 | 3271780640 | 0 | 12327 | 3271768743 | 11897 | 0.000 | 0.000 | 0.000 | 1 | - |
| **DHC7C** | 282 | 73 | 0 | 0 | 73 | 282 | 0.000 | 0.000 | 0.000 | 3271792967 | 11880 | 3271781087 | 12081 | 3271780886 | 0 | 12081 | 3271769006 | 11880 | 0.000 | 0.000 | 0.000 | 1 | - |
| **DHC8** | 288 | 73 | 0 | 1 | 72 | 287 | 0.000 | 0.014 | 0.000 | 3271792967 | 12190 | 3271780777 | 13143 | 3271779824 | 51 | 13092 | 3271767685 | 12139 | 0.004 | 0.004 | 0.004 | 1 | - |
| **DHC9A** | 261 | 75 | 0 | 2 | 73 | 259 | 0.000 | 0.027 | 0.000 | 3271792967 | 12186 | 3271780781 | 12840 | 3271780127 | 130 | 12710 | 3271768071 | 12056 | 0.011 | 0.010 | 0.010 | 1 | - |
| **DHC9B** | 317 | 85 | 0 | 0 | 85 | 317 | 0.000 | 0.000 | 0.000 | 3271792967 | 13791 | 3271779176 | 14145 | 3271778822 | 0 | 14145 | 3271765031 | 13791 | 0.000 | 0.000 | 0.000 | 1 | - |
| **DHC11** | 335 | 43 | 0 | 3 | 40 | 332 | 0.000 | 0.070 | 0.000 | 3271792967 | 14337 | 3271778630 | 14691 | 3271778276 | 147 | 14544 | 3271764086 | 14190 | 0.010 | 0.010 | 0.010 | 1 | - |
| **Others** | **94** | **21** | **1** | **3** | **18** | **91** | **0.048** | **0.143** | **0.011** | **242492081** | **6215** | **242485866** | **6153** | **242485928** | **1503** | **4650** | **242481216** | **4712** | **0.242** | **0.244** | **0.243** | **4** | **-** |
| **NedKinesinJ_fl** | 48 | 3 | 0 | 0 | 3 | 48 | 0.000 | 0.000 | 0.000 | 37302679 | 2273 | 37300406 | 2265 | 37300414 | 0 | 2265 | 37298141 | 2273 | 0.000 | 0.000 | 0.000 | 1 | - |
| **PhsP62_fl** | 3 | 3 | 1 | 3 | 0 | 0 | 0.333 | 1.000 | 0.333 | 77806163 | 1506 | 77804657 | 1512 | 77804651 | 1503 | 9 | 77804648 | 3 | 0.998 | 0.994 | 0.996 | 1 | - |
| **HsP50_fl** | | | | | | | | | | | | | | | | | | | | | | | |
| **PugCor_fl** | 22 | 10 | 0 | 0 | 10 | 22 | 0.000 | 0.000 | 0.000 | 88644628 | 1617 | 88643011 | 1551 | 88643077 | 0 | 1551 | 88641460 | 1617 | 0.000 | 0.000 | 0.000 | 1 | - |
| **MmP150_fl** | | | | | | | | | | | | | | | | | | | | | | | |
| **HsMyo19_fl** | | | | | | | | | | | | | | | | | | | | | | | |
| **ThCAP1_fl** | 21 | 5 | 0 | 0 | 5 | 21 | 0.000 | 0.000 | 0.000 | 38738611 | 819 | 38737792 | 825 | 38737786 | 0 | 825 | 38736967 | 819 | 0.000 | 0.000 | 0.000 | 1 | - |

**Prosplign (Blast)**

| | Exon PE | Exon AE | Exon TE | Exon OE | Exon ME | Exon WE | Exon Sn | Exon Sno | Exon Sp | Nucl Total | Nucl AP | Nucl AN | Nucl PP | Nucl PN | Nucl TP | Nucl FP | Nucl TN | Nucl FN | Nucl Sn | Nucl Sp | Nucl AC | Genes | Duration in s |
|---|---|---|---|---|---|---|---|---|---|---|---|---|---|---|---|---|---|---|---|---|---|---|---|
| **DHC all** | **8708** | **1202** | **0** | **59** | **1143** | **8645** | **0.000** | **0.049** | **0.000** | **52348687472** | **209482** | **52348477990** | **664431** | **52348023041** | **3100** | **661331** | **52347816659** | **206382** | **0.015** | **0.005** | **0.010** | **263** | **-** |
| **DHC1** | 565 | 77 | 0 | 0 | 77 | 565 | 0.000 | 0.000 | 0.000 | 3271792967 | 13682 | 3271779285 | 46530 | 3271746437 | 0 | 46530 | 3271732755 | 13682 | 0.000 | 0.000 | 0.000 | 15 | - |
| **DHC2** | 499 | 88 | 0 | 0 | 88 | 499 | 0.000 | 0.000 | 0.000 | 3271792967 | 12702 | 3271780265 | 36988 | 3271755979 | 0 | 36988 | 3271743277 | 12702 | 0.000 | 0.000 | 0.000 | 16 | - |
| **DHC3A** | 602 | 92 | 0 | 0 | 92 | 602 | 0.000 | 0.000 | 0.000 | 3271792967 | 14070 | 3271778897 | 44779 | 3271748188 | 0 | 44779 | 3271734118 | 14070 | 0.000 | 0.000 | 0.000 | 18 | - |
| **DHC3B** | 591 | 78 | 0 | 5 | 73 | 584 | 0.000 | 0.064 | 0.000 | 3271792967 | 13745 | 3271779222 | 46317 | 3271746650 | 280 | 46037 | 3271733185 | 13465 | 0.020 | 0.006 | 0.013 | 18 | - |
| **DHC4A** | 549 | 82 | 0 | 13 | 69 | 536 | 0.000 | 0.159 | 0.000 | 3271792967 | 13523 | 3271779444 | 37705 | 3271755262 | 647 | 37058 | 3271742386 | 12876 | 0.048 | 0.017 | 0.032 | 19 | - |
| **DHC4B** | 563 | 79 | 0 | 0 | 79 | 563 | 0.000 | 0.000 | 0.000 | 3271792967 | 13284 | 3271779683 | 41339 | 3271751628 | 0 | 41339 | 3271738344 | 13284 | 0.000 | 0.000 | 0.000 | 18 | - |
| **DHC4C** | 626 | 68 | 0 | 0 | 68 | 626 | 0.000 | 0.000 | 0.000 | 3271792967 | 13014 | 3271779953 | 45429 | 3271747538 | 0 | 45429 | 3271734524 | 13014 | 0.000 | 0.000 | 0.000 | 17 | - |
| **DHC5** | 621 | 79 | 0 | 0 | 79 | 621 | 0.000 | 0.000 | 0.000 | 3271792967 | 13752 | 3271779215 | 46745 | 3271746222 | 0 | 46745 | 3271732470 | 13752 | 0.000 | 0.000 | 0.000 | 19 | - |
| **DHC6** | 562 | 86 | 0 | 0 | 86 | 562 | 0.000 | 0.000 | 0.000 | 3271792967 | 13371 | 3271779596 | 43170 | 3271749797 | 0 | 43170 | 3271736426 | 13371 | 0.000 | 0.000 | 0.000 | 17 | - |
| **DHC7A** | 545 | 64 | 0 | 1 | 63 | 544 | 0.000 | 0.016 | 0.000 | 3271792967 | 12058 | 3271780909 | 45335 | 3271747632 | 6 | 45329 | 3271735580 | 12052 | 0.000 | 0.000 | 0.000 | 18 | - |
| **DHC7B** | 551 | 60 | 0 | 0 | 60 | 551 | 0.000 | 0.000 | 0.000 | 3271792967 | 11897 | 3271781070 | 42271 | 3271750696 | 0 | 42271 | 3271738799 | 11897 | 0.000 | 0.000 | 0.000 | 18 | - |
| **DHC7C** | 556 | 73 | 0 | 0 | 73 | 556 | 0.000 | 0.000 | 0.000 | 3271792967 | 11880 | 3271781087 | 41553 | 3271751414 | 0 | 41553 | 3271739534 | 11880 | 0.000 | 0.000 | 0.000 | 16 | - |
| **DHC8** | 592 | 73 | 0 | 18 | 55 | 574 | 0.000 | 0.247 | 0.000 | 3271792967 | 12190 | 3271780777 | 45620 | 3271747347 | 892 | 44728 | 3271736049 | 11298 | 0.073 | 0.020 | 0.046 | 18 | - |
| **DHC9A** | 574 | 75 | 0 | 10 | 65 | 564 | 0.000 | 0.133 | 0.000 | 3271792967 | 12186 | 3271780781 | 44179 | 3271748788 | 436 | 43743 | 3271737038 | 11750 | 0.036 | 0.010 | 0.023 | 17 | - |
| **DHC9B** | 612 | 85 | 0 | 0 | 85 | 612 | 0.000 | 0.000 | 0.000 | 3271792967 | 13791 | 3271779176 | 47001 | 3271745966 | 0 | 47001 | 3271732175 | 13791 | 0.000 | 0.000 | 0.000 | 17 | - |
| **DHC11** | 100 | 43 | 0 | 12 | 31 | 86 | 0.000 | 0.279 | 0.000 | 3271792967 | 14337 | 3271778630 | 9470 | 3271783497 | 839 | 8631 | 3271769999 | 13498 | 0.059 | 0.089 | 0.074 | 2 | - |
| **Others** | **598** | **83** | **1** | **18** | **65** | **580** | **0.012** | **0.217** | **0.002** | **8925425885** | **12569** | **8925413316** | **46886** | **8925378999** | **2190** | **44696** | **8925368620** | **10379** | **0.174** | **0.047** | **0.110** | **27** | **-** |
| **NedKinesinJ_fl** | 54 | 3 | 0 | 1 | 2 | 53 | 0.000 | 0.333 | 0.000 | 37302679 | 2273 | 37300406 | 4690 | 37297989 | 40 | 4650 | 37295756 | 2233 | 0.018 | 0.009 | 0.013 | 3 | - |
| **PhsP62_fl** | 39 | 3 | 1 | 3 | 0 | 36 | 0.333 | 1.000 | 0.026 | 77806163 | 1506 | 77804657 | 4427 | 77801736 | 1503 | 2924 | 77801733 | 3 | 0.998 | 0.340 | 0.669 | 3 | - |
| **HsP50_fl** | 17 | 16 | 0 | 1 | 15 | 16 | 0.000 | 0.063 | 0.000 | 3095677409 | 1218 | 3095676191 | 1035 | 3095676374 | 1 | 1034 | 3095675157 | 1217 | 0.001 | 0.001 | 0.001 | 1 | - |
| **PugCor_fl** | | | | | | | | | | | | | | | | | | | | | | | |
| **MmP150_fl** | 82 | 32 | 0 | 6 | 26 | 76 | 0.000 | 0.188 | 0.000 | 2580223614 | 3843 | 2580219771 | 6837 | 2580216777 | 307 | 6530 | 2580213241 | 3536 | 0.080 | 0.045 | 0.062 | 6 | - |
| **HsMyo19_fl** | 395 | 24 | 0 | 4 | 20 | 390 | 0.000 | 0.167 | 0.000 | 3095677409 | 2910 | 3095674499 | 29048 | 3095648361 | 274 | 28774 | 3095645725 | 2636 | 0.094 | 0.009 | 0.052 | 13 | - |
| **ThCAP1_fl** | 11 | 5 | 0 | 3 | 2 | 9 | 0.000 | 0.600 | 0.000 | 38738611 | 819 | 38737792 | 849 | 38737762 | 65 | 784 | 38737008 | 754 | 0.079 | 0.077 | 0.078 | 1 | - |

**Prosplign (Blat)**

| | Exon PE | Exon AE | Exon TE | Exon OE | Exon ME | Exon WE | Exon Sn | Exon Sno | Exon Sp | Nucl Total | Nucl AP | Nucl AN | Nucl PP | Nucl PN | Nucl TP | Nucl FP | Nucl TN | Nucl FN | Nucl Sn | Nucl Sp | Nucl AC | Genes | Duration in s |
|---|---|---|---|---|---|---|---|---|---|---|---|---|---|---|---|---|---|---|---|---|---|---|---|
| **DHC all** | | | | | | | | | | | | | | | | | | | | | | | |
| **DHC1** | | | | | | | | | | | | | | | | | | | | | | | |
| **DHC2** | | | | | | | | | | | | | | | | | | | | | | | |
| **DHC3A** | | | | | | | | | | | | | | | | | | | | | | | |
| **DHC3B** | | | | | | | | | | | | | | | | | | | | | | | |
| **DHC4A** | | | | | | | | | | | | | | | | | | | | | | | |
| **DHC4B** | | | | | | | | | | | | | | | | | | | | | | | |
| **DHC4C** | | | | | | | | | | | | | | | | | | | | | | | |
| **DHC5** | | | | | | | | | | | | | | | | | | | | | | | |
| **DHC6** | | | | | | | | | | | | | | | | | | | | | | | |
| **DHC7A** | | | | | | | | | | | | | | | | | | | | | | | |
| **DHC7B** | | | | | | | | | | | | | | | | | | | | | | | |
| **DHC7C** | | | | | | | | | | | | | | | | | | | | | | | |
| **DHC8** | | | | | | | | | | | | | | | | | | | | | | | |
| **DHC9A** | | | | | | | | | | | | | | | | | | | | | | | |
| **DHC9B** | | | | | | | | | | | | | | | | | | | | | | | |
| **DHC11** | | | | | | | | | | | | | | | | | | | | | | | |
| **Others** | **3934** | **83** | **1** | **18** | **65** | **3905** | **0.012** | **0.217** | **0.000** | **8925425885** | **12569** | **8925413316** | **134875** | **8925291010** | **2308** | **132567** | **8925280749** | **10261** | **0.184** | **0.017** | **0.100** | **146** | **-** |
| **NedKinesinJ_fl** | 1403 | 3 | 0 | 1 | 2 | 1400 | 0.000 | 0.333 | 0.000 | 37302679 | 2273 | 37300406 | 39406 | 37263273 | 158 | 39248 | 37261158 | 2115 | 0.070 | 0.004 | 0.036 | 47 | - |
| **PhsP62_fl** | 2110 | 3 | 1 | 3 | 0 | 2098 | 0.333 | 1.000 | 0.000 | 77806163 | 1506 | 77804657 | 64316 | 77741847 | 1503 | 62813 | 77741844 | 3 | 0.998 | 0.023 | 0.510 | 71 | - |
| **HsP50_fl** | 220 | 16 | 0 | 1 | 15 | 219 | 0.000 | 0.063 | 0.000 | 3095677409 | 1218 | 3095676191 | 16126 | 3095661283 | 1 | 16125 | 3095660066 | 1217 | 0.001 | 0.000 | 0.000 | 18 | - |
| **PugCor_fl** | | | | | | | | | | | | | | | | | | | | | | | |
| **MmP150_fl** | 53 | 32 | 0 | 6 | 26 | 47 | 0.000 | 0.188 | 0.000 | 2580223614 | 3843 | 2580219771 | 4297 | 2580219317 | 307 | 3990 | 2580215781 | 3536 | 0.080 | 0.071 | 0.076 | 3 | - |
| **HsMyo19_fl** | 137 | 24 | 0 | 4 | 20 | 132 | 0.000 | 0.167 | 0.000 | 3095677409 | 2910 | 3095674499 | 9881 | 3095667528 | 274 | 9607 | 3095664892 | 2636 | 0.094 | 0.028 | 0.061 | 6 | - |
| **ThCAP1_fl** | 11 | 5 | 0 | 3 | 2 | 9 | 0.000 | 0.600 | 0.000 | 38738611 | 819 | 38737792 | 849 | 38737762 | 65 | 784 | 38737008 | 754 | 0.079 | 0.077 | 0.078 | 1 | - |

**Exonerate**

| | Exon PE | Exon AE | Exon TE | Exon OE | Exon ME | Exon WE | Exon Sn | Exon Sno | Exon Sp | Nucl Total | Nucl AP | Nucl AN | Nucl PP | Nucl PN | Nucl TP | Nucl FP | Nucl TN | Nucl FN | Nucl Sn | Nucl Sp | Nucl AC | Genes | Duration in s |
|---|---|---|---|---|---|---|---|---|---|---|---|---|---|---|---|---|---|---|---|---|---|---|---|
| **DHC all** | **6896** | **1202** | **1140** | **1196** | **6** | **5669** | **0.948** | **0.995** | **0.165** | **52348687472** | **209483** | **52348477989** | **1129581** | **52347557891** | **208790** | **920791** | **52347557198** | **693** | **0.997** | **0.185** | **0.591** | **2145** | **7403** |
| **DHC1** | 242 | 77 | 77 | 77 | 0 | 165 | 1.000 | 1.000 | 0.318 | 3271792967 | 13683 | 3271779284 | 40757 | 3271752210 | 13683 | 27074 | 3271752210 | 0 | 1.000 | 0.336 | 0.668 | 81 | 6994 |
| **DHC2** | 271 | 88 | 86 | 88 | 0 | 183 | 0.977 | 1.000 | 0.317 | 3271792967 | 12702 | 3271780265 | 43812 | 3271749155 | 12702 | 31110 | 3271749155 | 0 | 1.000 | 0.290 | 0.645 | 80 | 7726 |
| **DHC3A** | 441 | 92 | 91 | 92 | 0 | 349 | 0.989 | 1.000 | 0.206 | 3271792967 | 14070 | 3271778897 | 73309 | 3271719658 | 14013 | 59296 | 3271719601 | 57 | 0.996 | 0.191 | 0.594 | 112 | 7485 |
| **DHC3B** | 449 | 78 | 75 | 78 | 0 | 371 | 0.962 | 1.000 | 0.167 | 3271792967 | 13745 | 3271779222 | 75766 | 3271717201 | 13745 | 62021 | 3271717201 | 0 | 1.000 | 0.181 | 0.591 | 132 | 7224 |
| **DHC4A** | 455 | 82 | 82 | 82 | 0 | 373 | 1.000 | 1.000 | 0.180 | 3271792967 | 13523 | 3271779444 | 78218 | 3271714749 | 13523 | 64695 | 3271714749 | 0 | 1.000 | 0.173 | 0.586 | 123 | 7264 |
| **DHC4B** | 475 | 79 | 74 | 79 | 0 | 395 | 0.937 | 1.000 | 0.156 | 3271792967 | 13284 | 3271779683 | 81263 | 3271711704 | 13265 | 67998 | 3271711685 | 19 | 0.999 | 0.163 | 0.581 | 135 | 7075 |
| **DHC4C** | 498 | 68 | 62 | 68 | 0 | 427 | 0.912 | 1.000 | 0.124 | 3271792967 | 13014 | 3271779953 | 83925 | 3271709042 | 13014 | 70911 | 3271709042 | 0 | 1.000 | 0.155 | 0.578 | 138 | 7328 |
| **DHC5** | 444 | 79 | 78 | 79 | 0 | 361 | 0.987 | 1.000 | 0.176 | 3271792967 | 13752 | 3271779215 | 75299 | 3271717668 | 13752 | 61547 | 3271717668 | 0 | 1.000 | 0.183 | 0.591 | 136 | 7356 |
| **DHC6** | 462 | 86 | 83 | 85 | 1 | 376 | 0.965 | 0.988 | 0.180 | 3271792967 | 13371 | 3271779596 | 78429 | 3271714538 | 13305 | 65124 | 3271714472 | 66 | 0.995 | 0.170 | 0.582 | 150 | 7396 |
| **DHC7A** | 498 | 64 | 63 | 64 | 0 | 430 | 0.984 | 1.000 | 0.127 | 3271792967 | 12058 | 3271780909 | 82492 | 3271710475 | 12058 | 70434 | 3271710475 | 0 | 1.000 | 0.146 | 0.573 | 125 | 6592 |
| **DHC7B** | 475 | 60 | 55 | 60 | 0 | 407 | 0.917 | 1.000 | 0.116 | 3271792967 | 11897 | 3271781070 | 79948 | 3271713019 | 11885 | 68063 | 3271713007 | 12 | 0.999 | 0.149 | 0.574 | 121 | 6445 |
| **DHC7C** | 489 | 73 | 68 | 72 | 1 | 410 | 0.932 | 0.986 | 0.139 | 3271792967 | 11880 | 3271781087 | 81201 | 3271711766 | 11849 | 69352 | 3271711735 | 31 | 0.997 | 0.146 | 0.572 | 130 | 6577 |
| **DHC8** | 481 | 73 | 65 | 73 | 0 | 407 | 0.890 | 1.000 | 0.135 | 3271792967 | 12190 | 3271780777 | 80889 | 3271712078 | 12067 | 68822 | 3271711955 | 123 | 0.990 | 0.149 | 0.570 | 145 | 7093 |
| **DHC9A** | 480 | 75 | 71 | 75 | 0 | 405 | 0.947 | 1.000 | 0.148 | 3271792967 | 12186 | 3271780781 | 80389 | 3271712578 | 12186 | 68203 | 3271712578 | 0 | 1.000 | 0.152 | 0.576 | 137 | 6255 |
| **DHC9B** | 363 | 85 | 72 | 82 | 3 | 280 | 0.847 | 0.965 | 0.198 | 3271792967 | 13791 | 3271779176 | 58707 | 3271734260 | 13500 | 45207 | 3271733969 | 291 | 0.979 | 0.230 | 0.604 | 133 | 8969 |
| **DHC11** | 373 | 43 | 38 | 42 | 1 | 330 | 0.884 | 0.977 | 0.102 | 3271792967 | 14337 | 3271778630 | 35177 | 3271757790 | 14243 | 20934 | 3271757696 | 94 | 0.993 | 0.405 | 0.699 | 267 | 10672 |
| **Others** | **519** | **93** | **77** | **87** | **6** | **431** | **0.828** | **0.935** | **0.148** | **9014070513** | **14186** | **9014056327** | **83611** | **9013986902** | **14107** | **69504** | **9013986823** | **79** | **0.994** | **0.169** | **0.582** | **175** | **887** |
| **NedKinesinJ_fl** | 15 | 3 | 2 | 3 | 0 | 11 | 0.667 | 1.000 | 0.133 | 37302679 | 2273 | 37300406 | 4064 | 37298615 | 2273 | 1791 | 37298615 | 0 | 1.000 | 0.559 | 0.780 | 7 | 37 |
| **PhsP62_fl** | 3 | 3 | 1 | 3 | 0 | 0 | 0.333 | 1.000 | 0.333 | 77806163 | 1506 | 77804657 | 1494 | 77804669 | 1494 | 0 | 77804657 | 12 | 0.992 | 1.000 | 0.996 | 1 | 49 |
| **HsP50_fl** | 14 | 16 | 13 | 14 | 2 | 0 | 0.813 | 0.875 | 0.929 | 3095677409 | 1218 | 3095676191 | 1218 | 3095676191 | 1203 | 15 | 3095676176 | 15 | 0.988 | 0.988 | 0.988 | 1 | 962 |
| **PugCor_fl** | 9 | 10 | 4 | 8 | 2 | 1 | 0.400 | 0.800 | 0.444 | 88644628 | 1617 | 88643011 | 1893 | 88642735 | 1596 | 297 | 88642714 | 21 | 0.987 | 0.843 | 0.915 | 2 | 61 |
| **MmP150_fl** | 242 | 32 | 32 | 32 | 0 | 210 | 1.000 | 1.000 | 0.132 | 2580223614 | 3843 | 2580219771 | 45307 | 2580178307 | 3843 | 41464 | 2580178307 | 0 | 1.000 | 0.085 | 0.542 | 84 | 3198 |
| **HsMyo19_fl** | 232 | 24 | 22 | 23 | 1 | 209 | 0.917 | 0.958 | 0.095 | 3095677409 | 2910 | 3095674499 | 28816 | 3095648593 | 2898 | 25918 | 3095648581 | 12 | 0.996 | 0.101 | 0.548 | 79 | 1886 |
| **ThCAP1_fl** | 4 | 5 | 3 | 4 | 1 | 0 | 0.600 | 0.800 | 0.750 | 38738611 | 819 | 38737792 | 819 | 38737792 | 800 | 19 | 38737773 | 19 | 0.977 | 0.977 | 0.977 | 1 | 13 |

**Exonerate (1 gene)**

| | Exon PE | Exon AE | Exon TE | Exon OE | Exon ME | Exon WE | Exon Sn | Exon Sno | Exon Sp | Nucl Total | Nucl AP | Nucl AN | Nucl PP | Nucl PN | Nucl TP | Nucl FP | Nucl TN | Nucl FN | Nucl Sn | Nucl Sp | Nucl AC | Genes | Duration in s |
|---|---|---|---|---|---|---|---|---|---|---|---|---|---|---|---|---|---|---|---|---|---|---|---|
| **DHC all** | **978** | **1202** | **882** | **915** | **287** | **62** | **0.734** | **0.761** | **0.902** | **52348687472** | **209483** | **52348477989** | **169074** | **52348518398** | **159502** | **9572** | **52348468417** | **49981** | **0.761** | **0.943** | **0.852** | **16** | **7287** |
| **DHC1** | 77 | 77 | 77 | 77 | 0 | 0 | 1.000 | 1.000 | 1.000 | 3271792967 | 13683 | 3271779284 | 13683 | 3271779284 | 13683 | 0 | 3271779284 | 0 | 1.000 | 1.000 | 1.000 | 1 | 6996 |
| **DHC2** | 53 | 88 | 50 | 51 | 37 | 2 | 0.568 | 0.580 | 0.943 | 3271792967 | 12702 | 3271780265 | 8382 | 3271784585 | 8310 | 72 | 3271780193 | 4392 | 0.654 | 0.991 | 0.823 | 1 | 7714 |
| **DHC3A** | 92 | 92 | 91 | 92 | 0 | 0 | 0.989 | 1.000 | 0.989 | 3271792967 | 14070 | 3271778897 | 14013 | 3271778954 | 14013 | 0 | 3271778897 | 57 | 0.996 | 1.000 | 0.998 | 1 | 7225 |
| **DHC3B** | 61 | 78 | 59 | 61 | 17 | 0 | 0.756 | 0.782 | 0.967 | 3271792967 | 13745 | 3271779222 | 10439 | 3271782528 | 10428 | 11 | 3271779211 | 3317 | 0.759 | 0.999 | 0.879 | 1 | 7154 |
| **DHC4A** | 82 | 82 | 82 | 82 | 0 | 0 | 1.000 | 1.000 | 1.000 | 3271792967 | 13523 | 3271779444 | 13523 | 3271779444 | 13523 | 0 | 3271779444 | 0 | 1.000 | 1.000 | 1.000 | 1 | 6990 |
| **DHC4B** | 49 | 79 | 45 | 49 | 30 | 0 | 0.570 | 0.620 | 0.918 | 3271792967 | 13284 | 3271779683 | 7881 | 3271785086 | 7871 | 10 | 3271779673 | 5413 | 0.593 | 0.999 | 0.796 | 1 | 6698 |
| **DHC4C** | 58 | 68 | 0 | 0 | 68 | 58 | 0.000 | 0.000 | 0.000 | 3271792967 | 13014 | 3271779953 | 9078 | 3271783889 | 0 | 9078 | 3271770875 | 13014 | 0.000 | 0.000 | 0.000 | 1 | 6917 |
| **DHC5** | 79 | 79 | 78 | 79 | 0 | 0 | 0.987 | 1.000 | 0.987 | 3271792967 | 13752 | 3271779215 | 13767 | 3271779200 | 13752 | 15 | 3271779200 | 0 | 1.000 | 0.999 | 0.999 | 1 | 6844 |
| **DHC6** | 48 | 86 | 46 | 48 | 38 | 0 | 0.535 | 0.558 | 0.958 | 3271792967 | 13371 | 3271779596 | 7623 | 3271785344 | 7599 | 24 | 3271779572 | 5772 | 0.568 | 0.997 | 0.783 | 1 | 7462 |
| **DHC7A** | 64 | 64 | 63 | 64 | 0 | 0 | 0.984 | 1.000 | 0.984 | 3271792967 | 12058 | 3271780909 | 12067 | 3271780900 | 12058 | 9 | 3271780900 | 0 | 1.000 | 0.999 | 1.000 | 1 | 6923 |
| **DHC7B** | 44 | 60 | 41 | 42 | 18 | 2 | 0.683 | 0.700 | 0.932 | 3271792967 | 11897 | 3271781070 | 6579 | 3271786388 | 6448 | 131 | 3271780939 | 5449 | 0.542 | 0.980 | 0.761 | 1 | 6444 |
| **DHC7C** | 72 | 73 | 68 | 72 | 1 | 0 | 0.932 | 0.986 | 0.944 | 3271792967 | 11880 | 3271781087 | 11880 | 3271781087 | 11849 | 31 | 3271781056 | 31 | 0.997 | 0.997 | 0.997 | 1 | 6276 |
| **DHC8** | 62 | 73 | 57 | 62 | 11 | 0 | 0.781 | 0.849 | 0.919 | 3271792967 | 12190 | 3271780777 | 10165 | 3271782802 | 10092 | 73 | 3271780704 | 2098 | 0.828 | 0.993 | 0.910 | 1 | 7053 |
| **DHC9A** | 50 | 75 | 48 | 50 | 25 | 0 | 0.640 | 0.667 | 0.960 | 3271792967 | 12186 | 3271780781 | 8364 | 3271784603 | 8357 | 7 | 3271780774 | 3829 | 0.686 | 0.999 | 0.842 | 1 | 6275 |
| **DHC9B** | 44 | 85 | 39 | 44 | 41 | 0 | 0.459 | 0.518 | 0.886 | 3271792967 | 13791 | 3271779176 | 7335 | 3271785632 | 7276 | 59 | 3271779117 | 6515 | 0.528 | 0.992 | 0.760 | 1 | 9456 |

| | | | | | | | | | | | | | | | | | | | | | | | |
|---|---|---|---|---|---|---|---|---|---|---|---|---|---|---|---|---|---|---|---|---|---|---|---|
| **DHC11** | 43 | 43 | 38 | 42 | 1 | 0 | 0.884 | 0.977 | 0.884 | 3271792967 | 14337 | 3271778630 | 14295 | 3271778672 | 14243 | 52 | 3271778578 | 94 | 0.993 | 0.996 | 0.995 | 1 | 10165 |
| **Others** | **87** | **93** | **77** | **87** | **6** | **0** | **0.828** | **0.935** | **0.885** | **9014070513** | **14186** | **9014056327** | **14090** | **9014056423** | **14029** | **61** | **9014056266** | **157** | **0.989** | **0.996** | **0.992** | **7** | **866** |
| **NedKinesinJ_fl** | 3 | 3 | 2 | 3 | 0 | 0 | 0.667 | 1.000 | 0.667 | 37302679 | 2273 | 37300406 | 2195 | 37300484 | 2195 | 0 | 37300406 | 78 | 0.966 | 1.000 | 0.983 | 1 | 35 |
| **PhsP62_fl** | 3 | 3 | 1 | 3 | 0 | 0 | 0.333 | 1.000 | 0.333 | 77806163 | 1506 | 77804657 | 1494 | 77804669 | 1494 | 0 | 77804657 | 12 | 0.992 | 1.000 | 0.996 | 1 | 49 |
| **HsP50_fl** | 14 | 16 | 13 | 14 | 2 | 0 | 0.813 | 0.875 | 0.929 | 3095677409 | 1218 | 3095676191 | 1218 | 3095676191 | 1203 | 15 | 3095676176 | 15 | 0.988 | 0.988 | 0.988 | 1 | 937 |
| **PugCor_fl** | 8 | 10 | 4 | 8 | 2 | 0 | 0.400 | 0.800 | 0.500 | 88644628 | 1617 | 88643011 | 1614 | 88643014 | 1596 | 18 | 88642993 | 21 | 0.987 | 0.989 | 0.988 | 1 | 61 |
| **MmP150_fl** | 32 | 32 | 32 | 32 | 0 | 0 | 1.000 | 1.000 | 1.000 | 2580223614 | 3843 | 2580219771 | 3843 | 2580219771 | 3843 | 0 | 2580219771 | 0 | 1.000 | 1.000 | 1.000 | 1 | 3096 |
| **HsMyo19_fl** | 23 | 24 | 22 | 23 | 1 | 0 | 0.917 | 0.958 | 0.957 | 3095677409 | 2910 | 3095674499 | 2907 | 3095674502 | 2898 | 9 | 3095674490 | 12 | 0.996 | 0.997 | 0.996 | 1 | 1872 |
| **ThCAP1_fl** | 4 | 5 | 3 | 4 | 1 | 0 | 0.600 | 0.800 | 0.750 | 38738611 | 819 | 38737792 | 819 | 38737792 | 800 | 19 | 38737773 | 19 | 0.977 | 0.977 | 0.977 | 1 | 13 |

**Wise2**

| | Exon PE | Exon AE | Exon TE | Exon OE | Exon ME | Exon WE | Exon Sn | Exon Sno | Exon Sp | Nucl Total | Nucl AP | Nucl AN | Nucl PP | Nucl PN | Nucl TP | Nucl FP | Nucl TN | Nucl FN | Nucl Sn | Nucl Sp | Nucl AC | Genes | Duration in s |
|---|---|---|---|---|---|---|---|---|---|---|---|---|---|---|---|---|---|---|---|---|---|---|---|
| **DHC all** | **34** | **1202** | **0** | **0** | **1202** | **34** | **0.000** | **0.000** | **0.000** | **52348687472** | **209483** | **52348477989** | **3267** | **52348684205** | **0** | **3267** | **52348474722** | **209483** | **0.000** | **0.000** | **0.000** | **32** | **-** |
| **DHC1** | 2 | 77 | 0 | 0 | 77 | 2 | 0.000 | 0.000 | 0.000 | 3271792967 | 13683 | 3271779284 | 177 | 3271792790 | 0 | 177 | 3271779107 | 13683 | 0.000 | 0.000 | 0.000 | 2 | - |
| **DHC2** | 2 | 88 | 0 | 0 | 88 | 2 | 0.000 | 0.000 | 0.000 | 3271792967 | 12702 | 3271780265 | 282 | 3271792685 | 0 | 282 | 3271779983 | 12702 | 0.000 | 0.000 | 0.000 | 2 | - |
| **DHC3A** | 2 | 92 | 0 | 0 | 92 | 2 | 0.000 | 0.000 | 0.000 | 3271792967 | 14070 | 3271778897 | 162 | 3271792805 | 0 | 162 | 3271778735 | 14070 | 0.000 | 0.000 | 0.000 | 2 | - |
| **DHC3B** | 2 | 78 | 0 | 0 | 78 | 2 | 0.000 | 0.000 | 0.000 | 3271792967 | 13745 | 3271779222 | 126 | 3271792841 | 0 | 126 | 3271779096 | 13745 | 0.000 | 0.000 | 0.000 | 2 | - |
| **DHC4A** | 2 | 82 | 0 | 0 | 82 | 2 | 0.000 | 0.000 | 0.000 | 3271792967 | 13523 | 3271779444 | 306 | 3271792661 | 0 | 306 | 3271779138 | 13523 | 0.000 | 0.000 | 0.000 | 2 | - |
| **DHC4B** | 3 | 79 | 0 | 0 | 79 | 3 | 0.000 | 0.000 | 0.000 | 3271792967 | 13284 | 3271779683 | 354 | 3271792613 | 0 | 354 | 3271779329 | 13284 | 0.000 | 0.000 | 0.000 | 2 | - |
| **DHC4C** | 2 | 68 | 0 | 0 | 68 | 2 | 0.000 | 0.000 | 0.000 | 3271792967 | 13014 | 3271779953 | 84 | 3271792883 | 0 | 84 | 3271779869 | 13014 | 0.000 | 0.000 | 0.000 | 2 | - |
| **DHC5** | 2 | 79 | 0 | 0 | 79 | 2 | 0.000 | 0.000 | 0.000 | 3271792967 | 13752 | 3271779215 | 123 | 3271792844 | 0 | 123 | 3271779092 | 13752 | 0.000 | 0.000 | 0.000 | 2 | - |
| **DHC6** | 3 | 86 | 0 | 0 | 86 | 3 | 0.000 | 0.000 | 0.000 | 3271792967 | 13371 | 3271779596 | 276 | 3271792691 | 0 | 276 | 3271779320 | 13371 | 0.000 | 0.000 | 0.000 | 2 | - |
| **DHC7A** | 2 | 64 | 0 | 0 | 64 | 2 | 0.000 | 0.000 | 0.000 | 3271792967 | 12058 | 3271780909 | 141 | 3271792826 | 0 | 141 | 3271780768 | 12058 | 0.000 | 0.000 | 0.000 | 2 | - |
| **DHC7B** | 2 | 60 | 0 | 0 | 60 | 2 | 0.000 | 0.000 | 0.000 | 3271792967 | 11897 | 3271781070 | 132 | 3271792835 | 0 | 132 | 3271780938 | 11897 | 0.000 | 0.000 | 0.000 | 2 | - |
| **DHC7C** | 2 | 73 | 0 | 0 | 73 | 2 | 0.000 | 0.000 | 0.000 | 3271792967 | 11880 | 3271781087 | 162 | 3271792805 | 0 | 162 | 3271780925 | 11880 | 0.000 | 0.000 | 0.000 | 2 | - |
| **DHC8** | 2 | 73 | 0 | 0 | 73 | 2 | 0.000 | 0.000 | 0.000 | 3271792967 | 12190 | 3271780777 | 141 | 3271792826 | 0 | 141 | 3271780636 | 12190 | 0.000 | 0.000 | 0.000 | 2 | - |
| **DHC9A** | 2 | 75 | 0 | 0 | 75 | 2 | 0.000 | 0.000 | 0.000 | 3271792967 | 12186 | 3271780781 | 297 | 3271792670 | 0 | 297 | 3271780484 | 12186 | 0.000 | 0.000 | 0.000 | 2 | - |
| **DHC9B** | 2 | 85 | 0 | 0 | 85 | 2 | 0.000 | 0.000 | 0.000 | 3271792967 | 13791 | 3271779176 | 225 | 3271792742 | 0 | 225 | 3271778951 | 13791 | 0.000 | 0.000 | 0.000 | 2 | - |
| **DHC11** | 2 | 43 | 0 | 0 | 43 | 2 | 0.000 | 0.000 | 0.000 | 3271792967 | 14337 | 3271778630 | 279 | 3271792688 | 0 | 279 | 3271778351 | 14337 | 0.000 | 0.000 | 0.000 | 2 | - |
| **Others** | **113** | **93** | **1** | **3** | **90** | **110** | **0.011** | **0.032** | **0.009** | **9014070513** | **14186** | **9014056327** | **10413** | **9014060100** | **1488** | **8925** | **9014047402** | **12698** | **0.105** | **0.143** | **0.124** | **14** | **-** |
| **NedKinesinJ_fl** | 3 | 3 | 0 | 0 | 3 | 3 | 0.000 | 0.000 | 0.000 | 37302679 | 2273 | 37300406 | 960 | 37301719 | 0 | 960 | 37299446 | 2273 | 0.000 | 0.000 | 0.000 | 2 | - |
| **PhsP62_fl** | 4 | 3 | 1 | 3 | 0 | 1 | 0.333 | 1.000 | 0.250 | 77806163 | 1506 | 77804657 | 1551 | 77804612 | 1488 | 63 | 77804594 | 18 | 0.988 | 0.959 | 0.974 | 2 | - |
| **HsP50_fl** | 22 | 16 | 0 | 0 | 16 | 22 | 0.000 | 0.000 | 0.000 | 3095677409 | 1218 | 3095676191 | 1209 | 3095676200 | 0 | 1209 | 3095674982 | 1218 | 0.000 | 0.000 | 0.000 | 2 | - |
| **PugCor_fl** | 2 | 10 | 0 | 0 | 10 | 2 | 0.000 | 0.000 | 0.000 | 88644628 | 1617 | 88643011 | 108 | 88644520 | 0 | 108 | 88642903 | 1617 | 0.000 | 0.000 | 0.000 | 2 | - |
| **MmP150_fl** | 30 | 32 | 0 | 0 | 32 | 30 | 0.000 | 0.000 | 0.000 | 2580223614 | 3843 | 2580219771 | 3183 | 2580220431 | 0 | 3183 | 2580216588 | 3843 | 0.000 | 0.000 | 0.000 | 2 | - |
| **HsMyo19_fl** | 48 | 24 | 0 | 0 | 24 | 48 | 0.000 | 0.000 | 0.000 | 3095677409 | 2910 | 3095674499 | 3144 | 3095674265 | 0 | 3144 | 3095671355 | 2910 | 0.000 | 0.000 | 0.000 | 2 | - |
| **ThCAP1_fl** | 4 | 5 | 0 | 0 | 5 | 4 | 0.000 | 0.000 | 0.000 | 38738611 | 819 | 38737792 | 258 | 38738353 | 0 | 258 | 38737534 | 819 | 0.000 | 0.000 | 0.000 | 2 | - |

**Wise2 (global)**

| | Exon PE | Exon AE | Exon TE | Exon OE | Exon ME | Exon WE | Exon Sn | Exon Sno | Exon Sp | Nucl Total | Nucl AP | Nucl AN | Nucl PP | Nucl PN | Nucl TP | Nucl FP | Nucl TN | Nucl FN | Nucl Sn | Nucl Sp | Nucl AC | Genes | Duration in s |
|---|---|---|---|---|---|---|---|---|---|---|---|---|---|---|---|---|---|---|---|---|---|---|---|
| **DHC all** | **39** | **1202** | **0** | **0** | **1202** | **39** | **0.000** | **0.000** | **0.000** | **52348687472** | **209483** | **52348477989** | **2625** | **52348684847** | **0** | **2625** | **52348475364** | **209483** | **0.000** | **0.000** | **0.000** | **32** | **-** |
| **DHC1** | 2 | 77 | 0 | 0 | 77 | 2 | 0.000 | 0.000 | 0.000 | 3271792967 | 13683 | 3271779284 | 126 | 3271792841 | 0 | 126 | 3271779158 | 13683 | 0.000 | 0.000 | 0.000 | 2 | - |
| **DHC2** | 2 | 88 | 0 | 0 | 88 | 2 | 0.000 | 0.000 | 0.000 | 3271792967 | 12702 | 3271780265 | 117 | 3271792850 | 0 | 117 | 3271780148 | 12702 | 0.000 | 0.000 | 0.000 | 2 | - |
| **DHC3A** | 5 | 92 | 0 | 0 | 92 | 5 | 0.000 | 0.000 | 0.000 | 3271792967 | 14070 | 3271778897 | 318 | 3271792649 | 0 | 318 | 3271778579 | 14070 | 0.000 | 0.000 | 0.000 | 2 | - |
| **DHC3B** | 2 | 78 | 0 | 0 | 78 | 2 | 0.000 | 0.000 | 0.000 | 3271792967 | 13745 | 3271779222 | 81 | 3271792886 | 0 | 81 | 3271779141 | 13745 | 0.000 | 0.000 | 0.000 | 2 | - |
| **DHC4A** | 3 | 82 | 0 | 0 | 82 | 3 | 0.000 | 0.000 | 0.000 | 3271792967 | 13523 | 3271779444 | 231 | 3271792736 | 0 | 231 | 3271779213 | 13523 | 0.000 | 0.000 | 0.000 | 2 | - |
| **DHC4B** | 2 | 79 | 0 | 0 | 79 | 2 | 0.000 | 0.000 | 0.000 | 3271792967 | 13284 | 3271779683 | 138 | 3271792829 | 0 | 138 | 3271779545 | 13284 | 0.000 | 0.000 | 0.000 | 2 | - |
| **DHC4C** | 3 | 68 | 0 | 0 | 68 | 3 | 0.000 | 0.000 | 0.000 | 3271792967 | 13014 | 3271779953 | 216 | 3271792751 | 0 | 216 | 3271779737 | 13014 | 0.000 | 0.000 | 0.000 | 2 | - |
| **DHC5** | 2 | 79 | 0 | 0 | 79 | 2 | 0.000 | 0.000 | 0.000 | 3271792967 | 13752 | 3271779215 | 78 | 3271792889 | 0 | 78 | 3271779137 | 13752 | 0.000 | 0.000 | 0.000 | 2 | - |
| **DHC6** | 2 | 86 | 0 | 0 | 86 | 2 | 0.000 | 0.000 | 0.000 | 3271792967 | 13371 | 3271779596 | 159 | 3271792808 | 0 | 159 | 3271779437 | 13371 | 0.000 | 0.000 | 0.000 | 2 | - |
| **DHC7A** | 2 | 64 | 0 | 0 | 64 | 2 | 0.000 | 0.000 | 0.000 | 3271792967 | 12058 | 3271780909 | 135 | 3271792832 | 0 | 135 | 3271780774 | 12058 | 0.000 | 0.000 | 0.000 | 2 | - |
| **DHC7B** | 3 | 60 | 0 | 0 | 60 | 3 | 0.000 | 0.000 | 0.000 | 3271792967 | 11897 | 3271781070 | 261 | 3271792706 | 0 | 261 | 3271780809 | 11897 | 0.000 | 0.000 | 0.000 | 2 | - |
| **DHC7C** | 2 | 73 | 0 | 0 | 73 | 2 | 0.000 | 0.000 | 0.000 | 3271792967 | 11880 | 3271781087 | 117 | 3271792850 | 0 | 117 | 3271780970 | 11880 | 0.000 | 0.000 | 0.000 | 2 | - |
| **DHC8** | 2 | 73 | 0 | 0 | 73 | 2 | 0.000 | 0.000 | 0.000 | 3271792967 | 12190 | 3271780777 | 171 | 3271792796 | 0 | 171 | 3271780606 | 12190 | 0.000 | 0.000 | 0.000 | 2 | - |
| **DHC9A** | 2 | 75 | 0 | 0 | 75 | 2 | 0.000 | 0.000 | 0.000 | 3271792967 | 12186 | 3271780781 | 192 | 3271792775 | 0 | 192 | 3271780589 | 12186 | 0.000 | 0.000 | 0.000 | 2 | - |
| **DHC9B** | 2 | 85 | 0 | 0 | 85 | 2 | 0.000 | 0.000 | 0.000 | 3271792967 | 13791 | 3271779176 | 135 | 3271792832 | 0 | 135 | 3271779041 | 13791 | 0.000 | 0.000 | 0.000 | 2 | - |
| **DHC11** | 3 | 43 | 0 | 0 | 43 | 3 | 0.000 | 0.000 | 0.000 | 3271792967 | 14337 | 3271778630 | 150 | 3271792817 | 0 | 150 | 3271778480 | 14337 | 0.000 | 0.000 | 0.000 | 2 | - |
| **Others** | **269** | **93** | **2** | **3** | **90** | **266** | **0.022** | **0.032** | **0.007** | **9014070513** | **14186** | **9014056327** | **28044** | **9014042469** | **1503** | **26541** | **9014029786** | **12683** | **0.106** | **0.054** | **0.080** | **14** | **-** |
| **NedKinesinJ_fl** | 22 | 3 | 0 | 0 | 3 | 22 | 0.000 | 0.000 | 0.000 | 37302679 | 2273 | 37300406 | 4416 | 37298263 | 0 | 4416 | 37295990 | 2273 | 0.000 | 0.000 | 0.000 | 2 | - |
| **PhsP62_fl** | 15 | 3 | 2 | 3 | 0 | 12 | 0.667 | 1.000 | 0.133 | 77806163 | 1506 | 77804657 | 3033 | 77803130 | 1503 | 1530 | 77803127 | 3 | 0.998 | 0.496 | 0.747 | 2 | - |
| **HsP50_fl** | 38 | 16 | 0 | 0 | 16 | 38 | 0.000 | 0.000 | 0.000 | 3095677409 | 1218 | 3095676191 | 2439 | 3095674970 | 0 | 2439 | 3095673752 | 1218 | 0.000 | 0.000 | 0.000 | 2 | - |
| **PugCor_fl** | 10 | 10 | 0 | 0 | 10 | 10 | 0.000 | 0.000 | 0.000 | 88644628 | 1617 | 88643011 | 3018 | 88641610 | 0 | 3018 | 88639993 | 1617 | 0.000 | 0.000 | 0.000 | 2 | - |
| **MmP150_fl** | 83 | 32 | 0 | 0 | 32 | 83 | 0.000 | 0.000 | 0.000 | 2580223614 | 3843 | 2580219771 | 7647 | 2580215967 | 0 | 7647 | 2580212124 | 3843 | 0.000 | 0.000 | 0.000 | 2 | - |
| **HsMyo19_fl** | 89 | 24 | 0 | 0 | 24 | 89 | 0.000 | 0.000 | 0.000 | 3095677409 | 2910 | 3095674499 | 5883 | 3095671526 | 0 | 5883 | 3095668616 | 2910 | 0.000 | 0.000 | 0.000 | 2 | - |
| **ThCAP1_fl** | 12 | 5 | 0 | 0 | 5 | 12 | 0.000 | 0.000 | 0.000 | 38738611 | 819 | 38737792 | 1608 | 38737003 | 0 | 1608 | 38736184 | 819 | 0.000 | 0.000 | 0.000 | 2 | - |

**Augustus**

| | Exon PE | Exon AE | Exon TE | Exon OE | Exon ME | Exon WE | Exon Sn | Exon Sno | Exon Sp | Nucl Total | Nucl AP | Nucl AN | Nucl PP | Nucl PN | Nucl TP | Nucl FP | Nucl TN | Nucl FN | Nucl Sn | Nucl Sp | Nucl AC | Genes | Duration in s |
|---|---|---|---|---|---|---|---|---|---|---|---|---|---|---|---|---|---|---|---|---|---|---|---|
| **DHC all** | **3929952** | **1202** | **576** | **1202** | **0** | **3909434** | **0.479** | **1.000** | **0.000** | **52348687472** | **209482** | **52348477990** | **79993616** | **52268693856** | **209476** | **79784140** | **52268693850** | **6** | **1.000** | **0.003** | **0.501** | **1374928** | **871977** |
| **DHC1** | 245622 | 77 | 73 | 77 | 0 | 244119 | 0.948 | 1.000 | 0.000 | 3271792967 | 13682 | 3271779285 | 4999601 | 3266793366 | 13682 | 4985919 | 3266793366 | 0 | 1.000 | 0.003 | 0.501 | 85933 | 871977 |
| **DHC2** | 245622 | 88 | 69 | 88 | 0 | 243901 | 0.784 | 1.000 | 0.000 | 3271792967 | 12702 | 3271780265 | 4999601 | 3266793366 | 12702 | 4986899 | 3266793366 | 0 | 1.000 | 0.003 | 0.501 | 85933 | 871977 |
| **DHC3A** | 245622 | 92 | 0 | 92 | 0 | 244008 | 0.000 | 1.000 | 0.000 | 3271792967 | 14070 | 3271778897 | 4999601 | 3266793366 | 14070 | 4985531 | 3266793366 | 0 | 1.000 | 0.003 | 0.501 | 85933 | 871977 |

| | | | | | | | | | | | | | | | | | | | | | | |
|---|---|---|---|---|---|---|---|---|---|---|---|---|---|---|---|---|---|---|---|---|---|---|
| **DHC3B** | 245622 | 78 | 71 | 78 | 0 | 244313 | 0.910 | 1.000 | 0.000 | 3271792967 | 13745 | 3271779222 | 4999601 | 3266793366 | 13745 | 4985856 | 3266793366 | 0 | 1.000 | 0.003 | 0.501 | 85933 | 871977 |
| **DHC4A** | 245622 | 82 | 67 | 82 | 0 | 244270 | 0.817 | 1.000 | 0.000 | 3271792967 | 13523 | 3271779444 | 4999601 | 3266793366 | 13523 | 4986078 | 3266793366 | 0 | 1.000 | 0.003 | 0.501 | 85933 | 871977 |
| **DHC4B** | 245622 | 79 | 0 | 79 | 0 | 244438 | 0.000 | 1.000 | 0.000 | 3271792967 | 13284 | 3271779683 | 4999601 | 3266793366 | 13278 | 4986323 | 3266793360 | 6 | 1.000 | 0.003 | 0.500 | 85933 | 871977 |
| **DHC4C** | 245622 | 68 | 0 | 68 | 0 | 244354 | 0.000 | 1.000 | 0.000 | 3271792967 | 13014 | 3271779953 | 4999601 | 3266793366 | 13014 | 4986587 | 3266793366 | 0 | 1.000 | 0.003 | 0.501 | 85933 | 871977 |
| **DHC5** | 245622 | 79 | 63 | 79 | 0 | 244241 | 0.797 | 1.000 | 0.000 | 3271792967 | 13752 | 3271779215 | 4999601 | 3266793366 | 13752 | 4985849 | 3266793366 | 0 | 1.000 | 0.003 | 0.501 | 85933 | 871977 |
| **DHC6** | 245622 | 86 | 0 | 86 | 0 | 244446 | 0.000 | 1.000 | 0.000 | 3271792967 | 13371 | 3271779596 | 4999601 | 3266793366 | 13371 | 4986230 | 3266793366 | 0 | 1.000 | 0.003 | 0.501 | 85933 | 871977 |
| **DHC7A** | 245622 | 64 | 45 | 64 | 0 | 244467 | 0.703 | 1.000 | 0.000 | 3271792967 | 12058 | 3271780909 | 4999601 | 3266793366 | 12058 | 4987543 | 3266793366 | 0 | 1.000 | 0.002 | 0.500 | 85933 | 871977 |
| **DHC7B** | 245622 | 60 | 51 | 60 | 0 | 244499 | 0.850 | 1.000 | 0.000 | 3271792967 | 11897 | 3271781070 | 4999601 | 3266793366 | 11897 | 4987704 | 3266793366 | 0 | 1.000 | 0.002 | 0.500 | 85933 | 871977 |
| **DHC7C** | 245622 | 73 | 0 | 73 | 0 | 244549 | 0.000 | 1.000 | 0.000 | 3271792967 | 11880 | 3271781087 | 4999601 | 3266793366 | 11880 | 4987721 | 3266793366 | 0 | 1.000 | 0.002 | 0.500 | 85933 | 871977 |
| **DHC8** | 245622 | 73 | 0 | 73 | 0 | 244489 | 0.000 | 1.000 | 0.000 | 3271792967 | 12190 | 3271780777 | 4999601 | 3266793366 | 12190 | 4987411 | 3266793366 | 0 | 1.000 | 0.002 | 0.500 | 85933 | 871977 |
| **DHC9A** | 245622 | 75 | 56 | 75 | 0 | 244459 | 0.747 | 1.000 | 0.000 | 3271792967 | 12186 | 3271780781 | 4999601 | 3266793366 | 12186 | 4987415 | 3266793366 | 0 | 1.000 | 0.002 | 0.500 | 85933 | 871977 |
| **DHC9B** | 245622 | 85 | 52 | 85 | 0 | 244161 | 0.612 | 1.000 | 0.000 | 3271792967 | 13791 | 3271779176 | 4999601 | 3266793366 | 13791 | 4985810 | 3266793366 | 0 | 1.000 | 0.003 | 0.501 | 85933 | 871977 |
| **DHC11** | 245622 | 43 | 29 | 43 | 0 | 244720 | 0.674 | 1.000 | 0.000 | 3271792967 | 14337 | 3271778630 | 4999601 | 3266793366 | 14337 | 4985264 | 3266793366 | 0 | 1.000 | 0.003 | 0.501 | 85933 | 871977 |
| **Others** | **966478** | **93** | **47** | **76** | **17** | **966183** | **0.505** | **0.817** | **0.000** | **9014070513** | **14186** | **9014056327** | **141130853** | **8872939660** | **12734** | **141118119** | **8872938208** | **1452** | **0.898** | **0.000** | **0.441** | **191112** | **0** |
| **NedKinesinJ_fl** | 21597 | 3 | 1 | 3 | 0 | 21547 | 0.333 | 1.000 | 0.000 | 37302679 | 2273 | 37300406 | 3676562 | 33626117 | 2273 | 3674289 | 33626117 | 0 | 1.000 | 0.001 | 0.451 | 8308 | 7549 |
| **PhsP62_fl** | 77735 | 3 | 0 | 3 | 0 | 77716 | 0.000 | 1.000 | 0.000 | 77806163 | 1506 | 77804657 | 8900749 | 68905414 | 1506 | 8899243 | 68905414 | 0 | 1.000 | 0.000 | 0.443 | 41970 | 10978 |
| **HsP50_fl** | 253715 | 16 | 4 | 8 | 8 | 253705 | 0.250 | 0.500 | 0.000 | 3095677409 | 1218 | 3095676191 | 38267936 | 3057409473 | 549 | 38267387 | 3057408804 | 669 | 0.451 | 0.000 | 0.219 | 33321 | 985981 |
| **PugCor_fl** | 69968 | 10 | 0 | 10 | 0 | 69826 | 0.000 | 1.000 | 0.000 | 88644628 | 1617 | 88643011 | 2550607 | 86094021 | 1617 | 2548990 | 86094021 | 0 | 1.000 | 0.001 | 0.486 | 24474 | 20188 |
| **MmP150_fl** | 262186 | 32 | 23 | 26 | 6 | 262149 | 0.719 | 0.813 | 0.000 | 2580223614 | 3843 | 2580219771 | 43333946 | 2536889668 | 3275 | 43330671 | 2536889100 | 568 | 0.852 | 0.000 | 0.418 | 41498 | 985981 |
| **HsMyo19_fl** | 253715 | 24 | 19 | 21 | 3 | 253687 | 0.792 | 0.875 | 0.000 | 3095677409 | 2910 | 3095674499 | 38267936 | 3057409473 | 2695 | 38265241 | 3057409258 | 215 | 0.926 | 0.000 | 0.457 | 33321 | 985981 |
| **ThCAP1_fl** | 27562 | 5 | 0 | 5 | 0 | 27553 | 0.000 | 1.000 | 0.000 | 38738611 | 819 | 38737792 | 6133117 | 32605494 | 819 | 6132298 | 32605494 | 0 | 1.000 | 0.000 | 0.421 | 8220 | 5442 |

**Blat**

| | Exon PE | Exon AE | Exon TE | Exon OE | Exon ME | Exon WE | Exon Sn | Exon Sno | Exon Sp | Nucl Total | Nucl AP | Nucl AN | Nucl PP | Nucl PN | Nucl TP | Nucl FP | Nucl TN | Nucl FN | Nucl Sn | Nucl Sp | Nucl AC | Genes | Duration in s |
|---|---|---|---|---|---|---|---|---|---|---|---|---|---|---|---|---|---|---|---|---|---|---|---|
| **DHC all** | **266197** | **1202** | **236** | **1193** | **9** | **264228** | **0.196** | **0.993** | **0.001** | **52348687472** | **209483** | **52348477989** | **7709272** | **52340978200** | **204091** | **7505181** | **52340972808** | **5392** | **0.974** | **0.026** | **0.500** | **-** | **444** |
| **DHC1** | 15874 | 77 | 21 | 77 | 0 | 15747 | 0.273 | 1.000 | 0.001 | 3271792967 | 13683 | 3271779284 | 438271 | 3271354696 | 13609 | 424662 | 3271354622 | 74 | 0.995 | 0.031 | 0.513 | - | 430 |
| **DHC2** | 22428 | 88 | 18 | 88 | 0 | 22255 | 0.205 | 1.000 | 0.001 | 3271792967 | 12702 | 3271780265 | 603847 | 3271189120 | 12591 | 591256 | 3271189009 | 111 | 0.991 | 0.021 | 0.506 | - | 492 |
| **DHC3A** | 16840 | 92 | 23 | 92 | 0 | 16699 | 0.250 | 1.000 | 0.001 | 3271792967 | 14070 | 3271778897 | 481511 | 3271311456 | 13696 | 467815 | 3271311082 | 374 | 0.973 | 0.028 | 0.501 | - | 456 |
| **DHC3B** | 14398 | 78 | 12 | 78 | 0 | 14283 | 0.154 | 1.000 | 0.001 | 3271792967 | 13745 | 3271779222 | 420914 | 3271372053 | 13516 | 407398 | 3271371824 | 229 | 0.983 | 0.032 | 0.508 | - | 421 |
| **DHC4A** | 14928 | 82 | 29 | 82 | 0 | 14795 | 0.354 | 1.000 | 0.002 | 3271792967 | 13523 | 3271779444 | 441194 | 3271351773 | 13389 | 427805 | 3271351639 | 134 | 0.990 | 0.030 | 0.510 | - | 437 |
| **DHC4B** | 11981 | 79 | 18 | 79 | 0 | 11873 | 0.228 | 1.000 | 0.002 | 3271792967 | 13284 | 3271779683 | 360638 | 3271432329 | 12996 | 347642 | 3271432041 | 288 | 0.978 | 0.036 | 0.507 | - | 410 |
| **DHC4C** | 15223 | 68 | 6 | 68 | 0 | 15107 | 0.088 | 1.000 | 0.000 | 3271792967 | 13014 | 3271779953 | 443298 | 3271349669 | 12781 | 430517 | 3271349436 | 233 | 0.982 | 0.029 | 0.505 | - | 427 |
| **DHC5** | 12339 | 79 | 14 | 79 | 0 | 12219 | 0.177 | 1.000 | 0.001 | 3271792967 | 13752 | 3271779215 | 362517 | 3271430450 | 13472 | 349045 | 3271430170 | 280 | 0.980 | 0.037 | 0.508 | - | 445 |
| **DHC6** | 15742 | 86 | 12 | 85 | 1 | 15610 | 0.140 | 0.988 | 0.001 | 3271792967 | 13371 | 3271779596 | 448068 | 3271344899 | 13006 | 435062 | 3271344534 | 365 | 0.973 | 0.029 | 0.501 | - | 446 |
| **DHC7A** | 16383 | 64 | 21 | 63 | 1 | 16281 | 0.328 | 0.984 | 0.001 | 3271792967 | 12058 | 3271780909 | 497257 | 3271295710 | 11909 | 485348 | 3271295561 | 149 | 0.988 | 0.024 | 0.506 | - | 448 |
| **DHC7B** | 14144 | 60 | 6 | 59 | 1 | 14039 | 0.100 | 0.983 | 0.000 | 3271792967 | 11897 | 3271781070 | 412977 | 3271379990 | 11692 | 401285 | 3271379785 | 205 | 0.983 | 0.028 | 0.505 | - | 449 |
| **DHC7C** | 12472 | 73 | 16 | 72 | 1 | 12356 | 0.219 | 0.986 | 0.001 | 3271792967 | 11880 | 3271781087 | 371141 | 3271421826 | 11755 | 359386 | 3271421701 | 125 | 0.989 | 0.032 | 0.511 | - | 381 |
| **DHC8** | 16617 | 73 | 15 | 73 | 0 | 16504 | 0.205 | 1.000 | 0.001 | 3271792967 | 12190 | 3271780777 | 481252 | 3271311715 | 11819 | 469433 | 3271311344 | 371 | 0.970 | 0.025 | 0.497 | - | 453 |
| **DHC9A** | 13421 | 75 | 14 | 75 | 0 | 13318 | 0.187 | 1.000 | 0.001 | 3271792967 | 12186 | 3271780781 | 389167 | 3271403800 | 11824 | 377343 | 3271403438 | 362 | 0.970 | 0.030 | 0.500 | - | 390 |
| **DHC9B** | 24120 | 85 | 8 | 82 | 3 | 23967 | 0.094 | 0.965 | 0.000 | 3271792967 | 13791 | 3271779176 | 769477 | 3271023490 | 12496 | 756981 | 3271022195 | 1295 | 0.906 | 0.016 | 0.461 | - | 472 |
| **DHC11** | 29287 | 43 | 3 | 41 | 2 | 29175 | 0.070 | 0.953 | 0.000 | 3271792967 | 14337 | 3271778630 | 787743 | 3271005224 | 13540 | 774203 | 3271004427 | 797 | 0.944 | 0.017 | 0.481 | - | 541 |
| **Others** | **39018** | **93** | **23** | **84** | **9** | **38891** | **0.247** | **0.903** | **0.001** | **22902550769** | **14186** | **22902536583** | **697742** | **22901853027** | **13887** | **683855** | **22901852728** | **299** | **0.979** | **0.020** | **0.499** | **-** | **372** |
| **NedKinesinJ_fl** | 19092 | 3 | 1 | 3 | 0 | 19058 | 0.333 | 1.000 | 0.000 | 3271792967 | 2273 | 3271790694 | 328419 | 3271464548 | 2243 | 326176 | 3271464518 | 30 | 0.987 | 0.007 | 0.497 | - | 511 |
| **PhsP62_fl** | 18956 | 3 | 0 | 3 | 0 | 18941 | 0.000 | 1.000 | 0.000 | 3271792967 | 1506 | 3271791461 | 332668 | 3271460299 | 1419 | 331249 | 3271460212 | 87 | 0.942 | 0.004 | 0.473 | - | 1093 |
| **HsP50_fl** | 96 | 16 | 4 | 14 | 2 | 82 | 0.250 | 0.875 | 0.042 | 3271792967 | 1218 | 3271791749 | 3483 | 3271789484 | 1194 | 2289 | 3271789460 | 24 | 0.980 | 0.343 | 0.662 | - | 322 |
| **PugCor_fl** | 11 | 10 | 2 | 8 | 2 | 3 | 0.200 | 0.800 | 0.182 | 3271792967 | 1617 | 3271791350 | 1683 | 3271791284 | 1597 | 86 | 3271791264 | 20 | 0.988 | 0.949 | 0.968 | - | 9 |
| **MmP150_fl** | 558 | 32 | 9 | 29 | 3 | 529 | 0.281 | 0.906 | 0.016 | 3271792967 | 3843 | 3271789124 | 18985 | 3271773982 | 3762 | 15223 | 3271773901 | 81 | 0.979 | 0.198 | 0.589 | - | 292 |
| **HsMyo19_fl** | 300 | 24 | 6 | 23 | 1 | 277 | 0.250 | 0.958 | 0.020 | 3271792967 | 2910 | 3271790057 | 11661 | 3271781306 | 2874 | 8787 | 3271781270 | 36 | 0.988 | 0.246 | 0.617 | - | 373 |
| **ThCAP1_fl** | 5 | 5 | 1 | 4 | 1 | 1 | 0.200 | 0.800 | 0.200 | 3271792967 | 819 | 3271792148 | 843 | 3271792124 | 798 | 45 | 3271792103 | 21 | 0.974 | 0.947 | 0.960 | - | 4 |

**Blast**

| | Exon PE | Exon AE | Exon TE | Exon OE | Exon ME | Exon WE | Exon Sn | Exon Sno | Exon Sp | Nucl Total | Nucl AP | Nucl AN | Nucl PP | Nucl PN | Nucl TP | Nucl FP | Nucl TN | Nucl FN | Nucl Sn | Nucl Sp | Nucl AC | Genes | Duration in s |
|---|---|---|---|---|---|---|---|---|---|---|---|---|---|---|---|---|---|---|---|---|---|---|---|
| **DHC all** | **6794** | **1202** | **48** | **1175** | **27** | **5619** | **0.040** | **0.978** | **0.007** | **52348687472** | **209483** | **52348477989** | **1397463** | **52347290009** | **204038** | **1193425** | **52347284564** | **5445** | **0.974** | **0.146** | **0.560** | **-** | **-** |
| **DHC1** | 296 | 77 | 0 | 75 | 2 | 230 | 0.000 | 0.974 | 0.000 | 3271792967 | 13683 | 3271779284 | 65551 | 3271727416 | 13494 | 52057 | 3271727227 | 189 | 0.986 | 0.206 | 0.596 | - | - |
| **DHC2** | 271 | 88 | 6 | 86 | 2 | 187 | 0.068 | 0.977 | 0.022 | 3271792967 | 12702 | 3271780265 | 55830 | 3271737137 | 12466 | 43364 | 3271736901 | 236 | 0.981 | 0.223 | 0.602 | - | - |
| **DHC3A** | 464 | 92 | 4 | 91 | 1 | 375 | 0.043 | 0.989 | 0.009 | 3271792967 | 14070 | 3271778897 | 94512 | 3271698455 | 13724 | 80788 | 3271698109 | 346 | 0.975 | 0.145 | 0.560 | - | - |
| **DHC3B** | 454 | 78 | 2 | 77 | 1 | 376 | 0.026 | 0.987 | 0.004 | 3271792967 | 13745 | 3271779222 | 92931 | 3271700036 | 13457 | 79474 | 3271699748 | 288 | 0.979 | 0.145 | 0.562 | - | - |
| **DHC4A** | 490 | 82 | 6 | 80 | 2 | 412 | 0.073 | 0.976 | 0.012 | 3271792967 | 13523 | 3271779444 | 99927 | 3271693040 | 13099 | 86828 | 3271692616 | 424 | 0.969 | 0.131 | 0.550 | - | - |
| **DHC4B** | 490 | 79 | 2 | 77 | 2 | 412 | 0.025 | 0.975 | 0.004 | 3271792967 | 13284 | 3271779683 | 99482 | 3271693485 | 12823 | 86659 | 3271693024 | 461 | 0.965 | 0.129 | 0.547 | - | - |
| **DHC4C** | 485 | 68 | 1 | 68 | 0 | 417 | 0.015 | 1.000 | 0.002 | 3271792967 | 13014 | 3271779953 | 99254 | 3271693713 | 12633 | 86621 | 3271693332 | 381 | 0.971 | 0.127 | 0.549 | - | - |
| **DHC5** | 462 | 79 | 2 | 77 | 2 | 382 | 0.025 | 0.975 | 0.004 | 3271792967 | 13752 | 3271779215 | 94363 | 3271698604 | 13508 | 80855 | 3271698360 | 244 | 0.982 | 0.143 | 0.563 | - | - |
| **DHC6** | 491 | 86 | 3 | 84 | 2 | 413 | 0.035 | 0.977 | 0.006 | 3271792967 | 13371 | 3271779596 | 102507 | 3271690460 | 12944 | 89563 | 3271690033 | 427 | 0.968 | 0.126 | 0.547 | - | - |
| **DHC7A** | 480 | 64 | 7 | 63 | 1 | 413 | 0.109 | 0.984 | 0.015 | 3271792967 | 12058 | 3271780909 | 95152 | 3271697815 | 11836 | 83316 | 3271697593 | 222 | 0.982 | 0.124 | 0.553 | - | - |
| **DHC7B** | 489 | 60 | 2 | 60 | 0 | 420 | 0.033 | 1.000 | 0.004 | 3271792967 | 11897 | 3271781070 | 98785 | 3271694182 | 11760 | 87025 | 3271694045 | 137 | 0.988 | 0.119 | 0.554 | - | - |
| **DHC7C** | 499 | 73 | 4 | 72 | 1 | 419 | 0.055 | 0.986 | 0.008 | 3271792967 | 11880 | 3271781087 | 100113 | 3271692854 | 11677 | 88436 | 3271692651 | 203 | 0.983 | 0.117 | 0.550 | - | - |
| **DHC8** | 511 | 73 | 2 | 72 | 1 | 440 | 0.027 | 0.986 | 0.004 | 3271792967 | 12190 | 3271780777 | 102630 | 3271690337 | 11862 | 90768 | 3271690009 | 328 | 0.973 | 0.116 | 0.544 | - | - |
| **DHC9A** | 498 | 75 | 5 | 75 | 0 | 423 | 0.067 | 1.000 | 0.010 | 3271792967 | 12186 | 3271780781 | 101592 | 3271691375 | 12087 | 89505 | 3271691276 | 99 | 0.992 | 0.119 | 0.555 | - | - |
| **DHC9B** | 373 | 85 | 2 | 79 | 6 | 294 | 0.024 | 0.929 | 0.005 | 3271792967 | 13791 | 3271779176 | 77608 | 3271715359 | 13020 | 64588 | 3271714588 | 771 | 0.944 | 0.168 | 0.556 | - | - |
| **DHC11** | 41 | 43 | 0 | 39 | 4 | 6 | 0.000 | 0.907 | 0.000 | 3271792967 | 14337 | 3271778630 | 17226 | 3271775741 | 13648 | 3578 | 3271775052 | 689 | 0.952 | 0.792 | 0.872 | - | - |
| **Others** | **279** | **93** | **5** | **79** | **14** | **209** | **0.054** | **0.849** | **0.018** | **9014070513** | **14186** | **9014056327** | **56764** | **9014013749** | **13470** | **43294** | **9014013033** | **716** | **0.950** | **0.237** | **0.593** | **-** | **-** |
| **NedKinesinJ_fl** | 20 | 3 | 0 | 3 | 0 | 16 | 0.000 | 1.000 | 0.000 | 37302679 | 2273 | 37300406 | 9501 | 37293178 | 2271 | 7230 | 37293176 | 2 | 0.999 | 0.239 | 0.619 | - | - |
| **PhsP62_fl** | 5 | 3 | 0 | 3 | 0 | 3 | 0.000 | 1.000 | 0.000 | 77806163 | 1506 | 77804657 | 1938 | 77804225 | 1488 | 450 | 77804207 | 18 | 0.988 | 0.768 | 0.878 | - | - |
| **HsP50_fl** | 12 | 16 | 2 | 12 | 4 | 0 | 0.125 | 0.750 | 0.167 | 3095677409 | 1218 | 3095676191 | 1107 | 3095676302 | 1013 | 94 | 3095676097 | 205 | 0.832 | 0.915 | 0.873 | - | - |

| | | | | | | | | | | | | | | | | | | | | | | | |
|---|---|---|---|---|---|---|---|---|---|---|---|---|---|---|---|---|---|---|---|---|---|---|---|
| **PugCor_fl** | 14 | 10 | 0 | 8 | 2 | 10 | 0.000 | 0.800 | 0.000 | 88644628 | 1617 | 88643011 | 5700 | 88638928 | 1600 | 4100 | 88638911 | 17 | 0.989 | 0.281 | 0.635 | - | - |
| **MmP150_fl** | 41 | 32 | 2 | 25 | 7 | 17 | 0.063 | 0.781 | 0.049 | 2580223614 | 3843 | 2580219771 | 7120 | 2580216494 | 3400 | 3720 | 2580216051 | 443 | 0.885 | 0.478 | 0.681 | - | - |
| **HsMyo19_fl** | 185 | 24 | 1 | 23 | 1 | 163 | 0.042 | 0.958 | 0.005 | 3095677409 | 2910 | 3095674499 | 30375 | 3095647034 | 2880 | 27495 | 3095647004 | 30 | 0.990 | 0.095 | 0.542 | - | - |
| **ThCAP1_fl** | 2 | 5 | 0 | 5 | 0 | 0 | 0.000 | 1.000 | 0.000 | 38738611 | 819 | 38737792 | 1023 | 38737588 | 818 | 205 | 38737587 | 1 | 0.999 | 0.800 | 0.899 | - | - |

## Scenarios 2 and 4 (gene region)

### Scipio v1.0

| | Exon PE | Exon AE | Exon TE | Exon OE | Exon ME | Exon WE | Exon Sn | Exon Sno | Exon Sp | Nucl Total | Nucl AP | Nucl AN | Nucl PP | Nucl PN | Nucl TP | Nucl FP | Nucl TN | Nucl FN | Nucl Sn | Nucl Sp | Nucl AC | Genes |
|---|---|---|---|---|---|---|---|---|---|---|---|---|---|---|---|---|---|---|---|---|---|---|
| **DHC all** | **1206** | **1202** | **1036** | **1186** | **16** | **10** | **0.862** | **0.987** | **0.859** | **3910464** | **209483** | **3700981** | **205217** | **3705247** | **204853** | **364** | **3700617** | **4630** | **0.978** | **0.998** | **0.987** | **16** |
| **DHC1** | 77 | 77 | 76 | 77 | 0 | 0 | 0.987 | 1.000 | 0.987 | 66248 | 13683 | 52565 | 13686 | 52562 | 13683 | 3 | 52562 | 0 | 1.000 | 1.000 | 1.000 | 1 |
| **DHC2** | 90 | 88 | 83 | 88 | 0 | 2 | 0.943 | 1.000 | 0.922 | 417085 | 12702 | 404383 | 12702 | 404383 | 12678 | 24 | 404359 | 24 | 0.998 | 0.998 | 0.998 | 1 |
| **DHC3A** | 93 | 92 | 89 | 92 | 0 | 0 | 0.967 | 1.000 | 0.957 | 362204 | 14070 | 348134 | 13815 | 348389 | 13815 | 0 | 348134 | 255 | 0.982 | 1.000 | 0.991 | 1 |
| **DHC3B** | 79 | 78 | 67 | 78 | 0 | 1 | 0.859 | 1.000 | 0.848 | 302827 | 13745 | 289082 | 13639 | 289188 | 13601 | 38 | 289044 | 144 | 0.990 | 0.997 | 0.993 | 1 |
| **DHC4A** | 83 | 82 | 78 | 82 | 0 | 1 | 0.951 | 1.000 | 0.940 | 365115 | 13523 | 351592 | 13523 | 351592 | 13511 | 12 | 351580 | 12 | 0.999 | 0.999 | 0.999 | 1 |
| **DHC4B** | 79 | 79 | 70 | 79 | 0 | 0 | 0.886 | 1.000 | 0.886 | 119251 | 13284 | 105967 | 13122 | 106129 | 13099 | 23 | 105944 | 185 | 0.986 | 0.998 | 0.991 | 1 |
| **DHC4C** | 68 | 68 | 61 | 68 | 0 | 0 | 0.897 | 1.000 | 0.897 | 490267 | 13014 | 477253 | 12895 | 477372 | 12886 | 9 | 477244 | 128 | 0.990 | 0.999 | 0.995 | 1 |
| **DHC5** | 78 | 79 | 71 | 78 | 1 | 0 | 0.899 | 0.987 | 0.910 | 144290 | 13752 | 130538 | 13617 | 130673 | 13584 | 33 | 130505 | 168 | 0.988 | 0.998 | 0.992 | 1 |
| **DHC6** | 84 | 86 | 80 | 84 | 2 | 0 | 0.930 | 0.977 | 0.952 | 138640 | 13371 | 125269 | 13056 | 125584 | 13055 | 1 | 125268 | 316 | 0.976 | 1.000 | 0.987 | 1 |
| **DHC7A** | 63 | 64 | 58 | 63 | 1 | 0 | 0.906 | 0.984 | 0.921 | 257605 | 12058 | 245547 | 11944 | 245661 | 11934 | 10 | 245537 | 124 | 0.990 | 0.999 | 0.994 | 1 |
| **DHC7B** | 59 | 60 | 50 | 59 | 1 | 0 | 0.833 | 0.983 | 0.847 | 191156 | 11897 | 179259 | 11730 | 179426 | 11726 | 4 | 179255 | 171 | 0.986 | 1.000 | 0.992 | 1 |
| **DHC7C** | 72 | 73 | 69 | 72 | 1 | 0 | 0.945 | 0.986 | 0.958 | 205577 | 11880 | 193697 | 11799 | 193778 | 11794 | 5 | 193692 | 86 | 0.993 | 1.000 | 0.996 | 1 |
| **DHC8** | 73 | 73 | 58 | 73 | 0 | 0 | 0.795 | 1.000 | 0.795 | 84895 | 12190 | 72705 | 11962 | 72933 | 11922 | 40 | 72665 | 268 | 0.978 | 0.997 | 0.985 | 1 |
| **DHC9A** | 75 | 75 | 59 | 74 | 1 | 1 | 0.787 | 0.987 | 0.787 | 306568 | 12186 | 294382 | 11733 | 294835 | 11693 | 40 | 294342 | 493 | 0.960 | 0.997 | 0.977 | 1 |
| **DHC9B** | 86 | 85 | 43 | 79 | 6 | 4 | 0.506 | 0.929 | 0.500 | 373531 | 13791 | 359740 | 12452 | 361079 | 12344 | 108 | 359632 | 1447 | 0.895 | 0.991 | 0.941 | 1 |
| **DHC11** | 47 | 43 | 24 | 40 | 3 | 1 | 0.558 | 0.930 | 0.511 | 85205 | 14337 | 70868 | 13542 | 71663 | 13528 | 14 | 70854 | 809 | 0.944 | 0.999 | 0.966 | 1 |
| **Others** | **87** | **93** | **75** | **83** | **10** | **1** | **0.806** | **0.892** | **0.862** | **108735** | **14186** | **94549** | **13957** | **94778** | **13900** | **57** | **94492** | **286** | **0.980** | **0.996** | **0.986** | **7** |
| **NedKinesinJ_fl** | 5 | 3 | 2 | 3 | 0 | 0 | 0.667 | 1.000 | 0.400 | 6820 | 2273 | 4547 | 2243 | 4577 | 2243 | 0 | 4547 | 30 | 0.987 | 1.000 | 0.990 | 1 |
| **PhsP62_fl** | 4 | 3 | 0 | 3 | 0 | 0 | 0.000 | 1.000 | 0.000 | 5645 | 1506 | 4139 | 1434 | 4211 | 1428 | 6 | 4133 | 78 | 0.948 | 0.996 | 0.962 | 1 |
| **HsP50_fl** | 13 | 16 | 13 | 13 | 3 | 0 | 0.813 | 0.813 | 1.000 | 20372 | 1218 | 19154 | 1167 | 19205 | 1167 | 0 | 19154 | 51 | 0.958 | 1.000 | 0.978 | 1 |
| **PugCor_fl** | 10 | 10 | 9 | 9 | 1 | 1 | 0.900 | 0.900 | 0.900 | 6606 | 1617 | 4989 | 1617 | 4989 | 1608 | 9 | 4980 | 9 | 0.994 | 0.994 | 0.993 | 1 |
| **MmP150_fl** | 28 | 32 | 26 | 28 | 4 | 0 | 0.813 | 0.875 | 0.929 | 23936 | 3843 | 20093 | 3770 | 20166 | 3756 | 14 | 20079 | 87 | 0.977 | 0.996 | 0.984 | 1 |
| **HsMyo19_fl** | 23 | 24 | 22 | 23 | 1 | 0 | 0.917 | 0.958 | 0.957 | 40271 | 2910 | 37361 | 2907 | 37364 | 2898 | 9 | 37352 | 12 | 0.996 | 0.997 | 0.996 | 1 |
| **ThCAP1_fl** | 4 | 5 | 3 | 4 | 1 | 0 | 0.600 | 0.800 | 0.750 | 5085 | 819 | 4266 | 819 | 4266 | 800 | 19 | 4247 | 19 | 0.977 | 0.977 | 0.972 | 1 |

### Scipio v1.5

| | Exon PE | Exon AE | Exon TE | Exon OE | Exon ME | Exon WE | Exon Sn | Exon Sno | Exon Sp | Nucl Total | Nucl AP | Nucl AN | Nucl PP | Nucl PN | Nucl TP | Nucl FP | Nucl TN | Nucl FN | Nucl Sn | Nucl Sp | Nucl AC | Genes |
|---|---|---|---|---|---|---|---|---|---|---|---|---|---|---|---|---|---|---|---|---|---|---|
| **DHC all** | **1202** | **1202** | **1119** | **1189** | **13** | **6** | **0.931** | **0.989** | **0.931** | **3910464** | **209486** | **3700978** | **206955** | **3703509** | **206529** | **426** | **3700552** | **2957** | **0.986** | **0.998** | **0.991** | **16** |
| **DHC1** | 77 | 77 | 77 | 77 | 0 | 0 | 1.000 | 1.000 | 1.000 | 66248 | 13686 | 52562 | 13686 | 52562 | 13686 | 0 | 52562 | 0 | 1.000 | 1.000 | 1.000 | 1 |
| **DHC2** | 89 | 88 | 86 | 88 | 0 | 1 | 0.977 | 1.000 | 0.966 | 417085 | 12702 | 404383 | 12780 | 404305 | 12702 | 78 | 404305 | 0 | 1.000 | 0.994 | 0.997 | 1 |
| **DHC3A** | 92 | 92 | 91 | 92 | 0 | 0 | 0.989 | 1.000 | 0.989 | 362204 | 14070 | 348134 | 13854 | 348350 | 13854 | 0 | 348134 | 216 | 0.985 | 1.000 | 0.992 | 1 |
| **DHC3B** | 78 | 78 | 74 | 78 | 0 | 0 | 0.949 | 1.000 | 0.949 | 302827 | 13745 | 289082 | 13748 | 289079 | 13735 | 13 | 289069 | 10 | 0.999 | 0.999 | 0.999 | 1 |
| **DHC4A** | 82 | 82 | 80 | 82 | 0 | 0 | 0.976 | 1.000 | 0.976 | 365115 | 13523 | 351592 | 13523 | 351592 | 13519 | 4 | 351588 | 4 | 1.000 | 1.000 | 1.000 | 1 |
| **DHC4B** | 79 | 79 | 75 | 79 | 0 | 0 | 0.949 | 1.000 | 0.949 | 119251 | 13284 | 105967 | 13223 | 106028 | 13198 | 25 | 105942 | 86 | 0.994 | 0.998 | 0.995 | 1 |
| **DHC4C** | 70 | 68 | 62 | 68 | 0 | 2 | 0.912 | 1.000 | 0.886 | 490267 | 13014 | 477253 | 13122 | 477145 | 13014 | 108 | 477145 | 0 | 1.000 | 0.992 | 0.996 | 1 |
| **DHC5** | 78 | 79 | 75 | 78 | 1 | 0 | 0.949 | 0.987 | 0.962 | 144290 | 13752 | 130538 | 13617 | 130673 | 13603 | 14 | 130524 | 149 | 0.989 | 0.999 | 0.993 | 1 |
| **DHC6** | 84 | 86 | 83 | 84 | 2 | 0 | 0.965 | 0.977 | 0.988 | 138640 | 13371 | 125269 | 13071 | 125569 | 13071 | 0 | 125269 | 300 | 0.978 | 1.000 | 0.988 | 1 |
| **DHC7A** | 64 | 64 | 63 | 64 | 0 | 0 | 0.984 | 1.000 | 0.984 | 257605 | 12058 | 245547 | 12064 | 245541 | 12058 | 6 | 245541 | 0 | 1.000 | 1.000 | 1.000 | 1 |
| **DHC7B** | 59 | 60 | 54 | 59 | 1 | 0 | 0.900 | 0.983 | 0.915 | 191156 | 11897 | 179259 | 11772 | 179384 | 11769 | 3 | 179256 | 128 | 0.989 | 1.000 | 0.994 | 1 |
| **DHC7C** | 73 | 73 | 71 | 73 | 0 | 0 | 0.973 | 1.000 | 0.973 | 205577 | 11880 | 193697 | 11880 | 193697 | 11876 | 4 | 193693 | 4 | 1.000 | 1.000 | 1.000 | 1 |
| **DHC8** | 75 | 73 | 65 | 73 | 0 | 2 | 0.890 | 1.000 | 0.867 | 84895 | 12190 | 72705 | 12068 | 72827 | 11998 | 70 | 72635 | 192 | 0.984 | 0.994 | 0.987 | 1 |
| **DHC9A** | 74 | 75 | 66 | 74 | 1 | 0 | 0.880 | 0.987 | 0.892 | 306568 | 12186 | 294382 | 11874 | 294694 | 11845 | 29 | 294353 | 341 | 0.972 | 0.998 | 0.984 | 1 |
| **DHC9B** | 83 | 85 | 65 | 80 | 5 | 1 | 0.765 | 0.941 | 0.783 | 373531 | 13791 | 359740 | 12960 | 360571 | 12898 | 62 | 359678 | 893 | 0.935 | 0.995 | 0.964 | 1 |
| **DHC11** | 45 | 43 | 32 | 40 | 3 | 0 | 0.744 | 0.930 | 0.711 | 85205 | 14337 | 70868 | 13713 | 71492 | 13703 | 10 | 70858 | 634 | 0.956 | 0.999 | 0.973 | 1 |
| **Others** | **91** | **93** | **91** | **91** | **2** | **0** | **0.978** | **0.978** | **1.000** | **108735** | **14186** | **94549** | **14117** | **94618** | **14117** | **0** | **94549** | **69** | **0.995** | **1.000** | **0.997** | **7** |
| **NedKinesinJ_fl** | 3 | 3 | 3 | 3 | 0 | 0 | 1.000 | 1.000 | 1.000 | 6820 | 2273 | 4547 | 2273 | 4547 | 2273 | 0 | 4547 | 0 | 1.000 | 1.000 | 1.000 | 1 |
| **PhsP62_fl** | 3 | 3 | 3 | 3 | 0 | 0 | 1.000 | 1.000 | 1.000 | 5645 | 1506 | 4139 | 1506 | 4139 | 1506 | 0 | 4139 | 0 | 1.000 | 1.000 | 1.000 | 1 |
| **HsP50_fl** | 15 | 16 | 15 | 15 | 1 | 0 | 0.938 | 0.938 | 1.000 | 20372 | 1218 | 19154 | 1182 | 19190 | 1182 | 0 | 19154 | 36 | 0.970 | 1.000 | 0.984 | 1 |
| **PugCor_fl** | 10 | 10 | 10 | 10 | 0 | 0 | 1.000 | 1.000 | 1.000 | 6606 | 1617 | 4989 | 1617 | 4989 | 1617 | 0 | 4989 | 0 | 1.000 | 1.000 | 1.000 | 1 |
| **MmP150_fl** | 31 | 32 | 31 | 31 | 1 | 0 | 0.969 | 0.969 | 1.000 | 23936 | 3843 | 20093 | 3810 | 20126 | 3810 | 0 | 20093 | 33 | 0.991 | 1.000 | 0.995 | 1 |
| **HsMyo19_fl** | 24 | 24 | 24 | 24 | 0 | 0 | 1.000 | 1.000 | 1.000 | 40271 | 2910 | 37361 | 2910 | 37361 | 2910 | 0 | 37361 | 0 | 1.000 | 1.000 | 1.000 | 1 |
| **ThCAP1_fl** | 5 | 5 | 5 | 5 | 0 | 0 | 1.000 | 1.000 | 1.000 | 5085 | 819 | 4266 | 819 | 4266 | 819 | 0 | 4266 | 0 | 1.000 | 1.000 | 1.000 | 1 |

### Scipio v1.5 (Blat tilesize 6)

| | Exon PE | Exon AE | Exon TE | Exon OE | Exon ME | Exon WE | Exon Sn | Exon Sno | Exon Sp | Nucl Total | Nucl AP | Nucl AN | Nucl PP | Nucl PN | Nucl TP | Nucl FP | Nucl TN | Nucl FN | Nucl Sn | Nucl Sp | Nucl AC | Genes |
|---|---|---|---|---|---|---|---|---|---|---|---|---|---|---|---|---|---|---|---|---|---|---|
| **DHC all** | **1215** | **1202** | **1138** | **1198** | **4** | **7** | **0.947** | **0.997** | **0.937** | **3910464** | **209483** | **3700981** | **208343** | **3702121** | **207834** | **509** | **3700472** | **1649** | **0.992** | **0.998** | **0.995** | **16** |
| **DHC1** | 77 | 77 | 77 | 77 | 0 | 0 | 1.000 | 1.000 | 1.000 | 66248 | 13683 | 52565 | 13683 | 52565 | 13683 | 0 | 52565 | 0 | 1.000 | 1.000 | 1.000 | 1 |
| **DHC2** | 89 | 88 | 87 | 88 | 0 | 1 | 0.989 | 1.000 | 0.978 | 417085 | 12702 | 404383 | 12777 | 404308 | 12702 | 75 | 404308 | 0 | 1.000 | 0.994 | 0.997 | 1 |
| **DHC3A** | 92 | 92 | 91 | 92 | 0 | 0 | 0.989 | 1.000 | 0.989 | 362204 | 14070 | 348134 | 13944 | 348260 | 13944 | 0 | 348134 | 126 | 0.991 | 1.000 | 0.995 | 1 |
| **DHC3B** | 78 | 78 | 74 | 78 | 0 | 0 | 0.949 | 1.000 | 0.949 | 302827 | 13745 | 289082 | 13751 | 289076 | 13735 | 16 | 289066 | 10 | 0.999 | 0.999 | 0.999 | 1 |
| **DHC4A** | 82 | 82 | 80 | 82 | 0 | 0 | 0.976 | 1.000 | 0.976 | 365115 | 13523 | 351592 | 13523 | 351592 | 13519 | 4 | 351588 | 4 | 1.000 | 1.000 | 1.000 | 1 |
| **DHC4B** | 79 | 79 | 75 | 79 | 0 | 0 | 0.949 | 1.000 | 0.949 | 119251 | 13284 | 105967 | 13220 | 106031 | 13198 | 22 | 105945 | 86 | 0.994 | 0.998 | 0.995 | 1 |
| **DHC4C** | 71 | 68 | 62 | 68 | 0 | 2 | 0.912 | 1.000 | 0.873 | 490267 | 13014 | 477253 | 13076 | 477191 | 12968 | 108 | 477145 | 46 | 0.996 | 0.992 | 0.994 | 1 |
| **DHC5** | 79 | 79 | 76 | 79 | 0 | 0 | 0.962 | 1.000 | 0.962 | 144290 | 13752 | 130538 | 13653 | 130637 | 13639 | 14 | 130524 | 113 | 0.992 | 0.999 | 0.995 | 1 |
| **DHC6** | 85 | 86 | 84 | 85 | 1 | 0 | 0.977 | 0.988 | 0.988 | 138640 | 13371 | 125269 | 13116 | 125524 | 13116 | 0 | 125269 | 255 | 0.981 | 1.000 | 0.989 | 1 |
| **DHC7A** | 64 | 64 | 63 | 64 | 0 | 0 | 0.984 | 1.000 | 0.984 | 257605 | 12058 | 245547 | 12064 | 245541 | 12058 | 6 | 245541 | 0 | 1.000 | 1.000 | 1.000 | 1 |
| **DHC7B** | 60 | 60 | 58 | 60 | 0 | 0 | 0.967 | 1.000 | 0.967 | 191156 | 11897 | 179259 | 11892 | 179264 | 11889 | 3 | 179256 | 8 | 0.999 | 1.000 | 1.000 | 1 |
| **DHC7C** | 73 | 73 | 71 | 73 | 0 | 0 | 0.973 | 1.000 | 0.973 | 205577 | 11880 | 193697 | 11880 | 193697 | 11876 | 4 | 193693 | 4 | 1.000 | 1.000 | 1.000 | 1 |
| **DHC8** | 75 | 73 | 65 | 73 | 0 | 2 | 0.890 | 1.000 | 0.867 | 84895 | 12190 | 72705 | 12068 | 72827 | 11998 | 70 | 72635 | 192 | 0.984 | 0.994 | 0.987 | 1 |
| **DHC9A** | 75 | 75 | 68 | 75 | 0 | 0 | 0.907 | 1.000 | 0.907 | 306568 | 12186 | 294382 | 12078 | 294490 | 12058 | 20 | 294362 | 128 | 0.989 | 0.998 | 0.994 | 1 |
| **DHC9B** | 89 | 85 | 72 | 83 | 2 | 2 | 0.847 | 0.976 | 0.809 | 373531 | 13791 | 359740 | 13623 | 359908 | 13470 | 153 | 359587 | 321 | 0.977 | 0.989 | 0.982 | 1 |
| **DHC11** | 47 | 43 | 35 | 42 | 1 | 0 | 0.814 | 0.977 | 0.745 | 85205 | 14337 | 70868 | 13995 | 71210 | 13981 | 14 | 70854 | 356 | 0.975 | 0.999 | 0.984 | 1 |
| **Others** | **87** | **87** | **87** | **87** | **0** | **0** | **1.000** | **1.000** | **1.000** | **96270** | **10407** | **85863** | **10407** | **85863** | **10407** | **0** | **85863** | **0** | **1.000** | **1.000** | **1.000** | **5** |
| **HsP50_fl** | 16 | 16 | 16 | 16 | 0 | 0 | 1.000 | 1.000 | 1.000 | 20372 | 1218 | 19154 | 1218 | 19154 | 1218 | 0 | 19154 | 0 | 1.000 | 1.000 | 1.000 | 1 |
| **PugCor_fl** | 10 | 10 | 10 | 10 | 0 | 0 | 1.000 | 1.000 | 1.000 | 6606 | 1617 | 4989 | 1617 | 4989 | 1617 | 0 | 4989 | 0 | 1.000 | 1.000 | 1.000 | 1 |
| **MmP150_fl** | 32 | 32 | 32 | 32 | 0 | 0 | 1.000 | 1.000 | 1.000 | 23936 | 3843 | 20093 | 3843 | 20093 | 3843 | 0 | 20093 | 0 | 1.000 | 1.000 | 1.000 | 1 |
| **HsMyo19_fl** | 24 | 24 | 24 | 24 | 0 | 0 | 1.000 | 1.000 | 1.000 | 40271 | 2910 | 37361 | 2910 | 37361 | 2910 | 0 | 37361 | 0 | 1.000 | 1.000 | 1.000 | 1 |
| **ThCAP1_fl** | 5 | 5 | 5 | 5 | 0 | 0 | 1.000 | 1.000 | 1.000 | 5085 | 819 | 4266 | 819 | 4266 | 819 | 0 | 4266 | 0 | 1.000 | 1.000 | 1.000 | 1 |

**Scipio v1.5 (Blat tilesize 5)**

| | Exon PE | Exon AE | Exon TE | Exon OE | Exon ME | Exon WE | Exon Sn | Exon Sno | Exon Sp | Nucl Total | Nucl AP | Nucl AN | Nucl PP | Nucl PN | Nucl TP | Nucl FP | Nucl TN | Nucl FN | Nucl Sn | Nucl Sp | Nucl AC | Genes |
|---|---|---|---|---|---|---|---|---|---|---|---|---|---|---|---|---|---|---|---|---|---|---|
| **DHC all** | **1231** | **1202** | **1134** | **1199** | **3** | **23** | **0.943** | **0.998** | **0.921** | **3910464** | **209486** | **3700978** | **209026** | **3701438** | **208251** | **775** | **3700203** | **1235** | **0.994** | **0.996** | **0.995** | **16** |
| **DHC1** | 77 | 77 | 77 | 77 | 0 | 0 | 1.000 | 1.000 | 1.000 | 66248 | 13686 | 52562 | 13686 | 52562 | 13686 | 0 | 52562 | 0 | 1.000 | 1.000 | 1.000 | 1 |
| **DHC2** | 90 | 88 | 86 | 88 | 0 | 2 | 0.977 | 1.000 | 0.956 | 417085 | 12702 | 404383 | 12786 | 404299 | 12702 | 84 | 404299 | 0 | 1.000 | 0.993 | 0.997 | 1 |
| **DHC3A** | 92 | 92 | 91 | 92 | 0 | 0 | 0.989 | 1.000 | 0.989 | 362204 | 14070 | 348134 | 13941 | 348263 | 13941 | 0 | 348134 | 129 | 0.991 | 1.000 | 0.995 | 1 |
| **DHC3B** | 80 | 78 | 73 | 78 | 0 | 2 | 0.936 | 1.000 | 0.913 | 302827 | 13745 | 289082 | 13763 | 289064 | 13724 | 39 | 289043 | 21 | 0.998 | 0.997 | 0.998 | 1 |
| **DHC4A** | 82 | 82 | 80 | 82 | 0 | 0 | 0.976 | 1.000 | 0.976 | 365115 | 13523 | 351592 | 13523 | 351592 | 13519 | 4 | 351588 | 4 | 1.000 | 1.000 | 1.000 | 1 |
| **DHC4B** | 80 | 79 | 75 | 79 | 0 | 0 | 0.949 | 1.000 | 0.938 | 119251 | 13284 | 105967 | 13247 | 106004 | 13225 | 22 | 105945 | 59 | 0.996 | 0.998 | 0.997 | 1 |
| **DHC4C** | 72 | 68 | 63 | 68 | 0 | 3 | 0.926 | 1.000 | 0.875 | 490267 | 13014 | 477253 | 13088 | 477179 | 12968 | 120 | 477133 | 46 | 0.996 | 0.991 | 0.993 | 1 |
| **DHC5** | 80 | 79 | 76 | 79 | 0 | 1 | 0.962 | 1.000 | 0.950 | 144290 | 13752 | 130538 | 13656 | 130634 | 13636 | 20 | 130518 | 116 | 0.992 | 0.999 | 0.995 | 1 |
| **DHC6** | 86 | 86 | 83 | 85 | 1 | 1 | 0.965 | 0.988 | 0.965 | 138640 | 13371 | 125269 | 13122 | 125518 | 13113 | 9 | 125260 | 258 | 0.981 | 0.999 | 0.989 | 1 |
| **DHC7A** | 64 | 64 | 63 | 64 | 0 | 0 | 0.984 | 1.000 | 0.984 | 257605 | 12058 | 245547 | 12064 | 245541 | 12058 | 6 | 245541 | 0 | 1.000 | 1.000 | 1.000 | 1 |
| **DHC7B** | 61 | 60 | 57 | 60 | 0 | 1 | 0.950 | 1.000 | 0.934 | 191156 | 11897 | 179259 | 11969 | 179187 | 11894 | 75 | 179184 | 3 | 1.000 | 0.994 | 0.997 | 1 |
| **DHC7C** | 73 | 73 | 71 | 73 | 0 | 0 | 0.973 | 1.000 | 0.973 | 205577 | 11880 | 193697 | 11880 | 193697 | 11876 | 4 | 193693 | 4 | 1.000 | 1.000 | 1.000 | 1 |
| **DHC8** | 76 | 73 | 64 | 73 | 0 | 3 | 0.877 | 1.000 | 0.842 | 84895 | 12190 | 72705 | 12263 | 72632 | 12172 | 91 | 72614 | 18 | 0.999 | 0.993 | 0.995 | 1 |
| **DHC9A** | 76 | 75 | 68 | 75 | 0 | 1 | 0.907 | 1.000 | 0.895 | 306568 | 12186 | 294382 | 12204 | 294364 | 12169 | 35 | 294347 | 17 | 0.999 | 0.997 | 0.998 | 1 |
| **DHC9B** | 95 | 85 | 73 | 84 | 1 | 8 | 0.859 | 0.988 | 0.768 | 373531 | 13791 | 359740 | 13704 | 359827 | 13467 | 237 | 359503 | 324 | 0.977 | 0.983 | 0.979 | 1 |
| **DHC11** | 47 | 43 | 34 | 42 | 1 | 1 | 0.791 | 0.977 | 0.723 | 85205 | 14337 | 70868 | 14130 | 71075 | 14101 | 29 | 70839 | 236 | 0.984 | 0.998 | 0.989 | 1 |
| **Others** | **87** | **87** | **87** | **87** | **0** | **0** | **1.000** | **1.000** | **1.000** | **96270** | **10407** | **85863** | **10407** | **85863** | **10407** | **0** | **85863** | **0** | **1.000** | **1.000** | **1.000** | **5** |
| **HsP50_fl** | 16 | 16 | 16 | 16 | 0 | 0 | 1.000 | 1.000 | 1.000 | 20372 | 1218 | 19154 | 1218 | 19154 | 1218 | 0 | 19154 | 0 | 1.000 | 1.000 | 1.000 | 1 |
| **PugCor_fl** | 10 | 10 | 10 | 10 | 0 | 0 | 1.000 | 1.000 | 1.000 | 6606 | 1617 | 4989 | 1617 | 4989 | 1617 | 0 | 4989 | 0 | 1.000 | 1.000 | 1.000 | 1 |
| **MmP150_fl** | 32 | 32 | 32 | 32 | 0 | 0 | 1.000 | 1.000 | 1.000 | 23936 | 3843 | 20093 | 3843 | 20093 | 3843 | 0 | 20093 | 0 | 1.000 | 1.000 | 1.000 | 1 |
| **HsMyo19_fl** | 24 | 24 | 24 | 24 | 0 | 0 | 1.000 | 1.000 | 1.000 | 40271 | 2910 | 37361 | 2910 | 37361 | 2910 | 0 | 37361 | 0 | 1.000 | 1.000 | 1.000 | 1 |
| **ThCAP1_fl** | 5 | 5 | 5 | 5 | 0 | 0 | 1.000 | 1.000 | 1.000 | 5085 | 819 | 4266 | 819 | 4266 | 819 | 0 | 4266 | 0 | 1.000 | 1.000 | 1.000 | 1 |

**Prosplign**

| | Exon PE | Exon AE | Exon TE | Exon OE | Exon ME | Exon WE | Exon Sn | Exon Sno | Exon Sp | Nucl Total | Nucl AP | Nucl AN | Nucl PP | Nucl PN | Nucl TP | Nucl FP | Nucl TN | Nucl FN | Nucl Sn | Nucl Sp | Nucl AC | Genes |
|---|---|---|---|---|---|---|---|---|---|---|---|---|---|---|---|---|---|---|---|---|---|---|
| **DHC all** | **1242** | **1202** | **1150** | **1201** | **1** | **41** | **0.957** | **0.999** | **0.926** | **3910464** | **209482** | **3700982** | **211921** | **3698543** | **209179** | **2742** | **3698240** | **303** | **0.999** | **0.987** | **0.992** | **16** |
| **DHC1** | 79 | 77 | 77 | 77 | 0 | 2 | 1.000 | 1.000 | 0.975 | 66248 | 13682 | 52566 | 13746 | 52502 | 13682 | 64 | 52502 | 0 | 1.000 | 0.995 | 0.997 | 1 |
| **DHC2** | 93 | 88 | 86 | 88 | 0 | 5 | 0.977 | 1.000 | 0.925 | 417085 | 12702 | 404383 | 12912 | 404173 | 12702 | 210 | 404173 | 0 | 1.000 | 0.984 | 0.992 | 1 |
| **DHC3A** | 93 | 92 | 91 | 92 | 0 | 1 | 0.989 | 1.000 | 0.978 | 362204 | 14070 | 348134 | 14145 | 348059 | 14070 | 75 | 348059 | 0 | 1.000 | 0.995 | 0.997 | 1 |
| **DHC3B** | 79 | 78 | 74 | 78 | 0 | 1 | 0.949 | 1.000 | 0.937 | 302827 | 13745 | 289082 | 13895 | 288932 | 13735 | 160 | 288922 | 10 | 0.999 | 0.988 | 0.994 | 1 |
| **DHC4A** | 82 | 82 | 80 | 82 | 0 | 0 | 0.976 | 1.000 | 0.976 | 365115 | 13523 | 351592 | 13523 | 351592 | 13519 | 4 | 351588 | 4 | 1.000 | 1.000 | 1.000 | 1 |
| **DHC4B** | 82 | 79 | 75 | 79 | 0 | 3 | 0.949 | 1.000 | 0.915 | 119251 | 13284 | 105967 | 13425 | 105826 | 13226 | 199 | 105768 | 58 | 0.996 | 0.985 | 0.989 | 1 |

| | | | | | | | | | | | | | | | | | | | | | | |
|---|---|---|---|---|---|---|---|---|---|---|---|---|---|---|---|---|---|---|---|---|---|---|
| **DHC4C** | 76 | 68 | 61 | 68 | 0 | 8 | 0.897 | 1.000 | 0.803 | 490267 | 13014 | 477253 | 13498 | 476769 | 13000 | 498 | 476755 | 14 | 0.999 | 0.963 | 0.980 | 1 |
| **DHC5** | 79 | 79 | 79 | 79 | 0 | 0 | 1.000 | 1.000 | 1.000 | 144290 | 13752 | 130538 | 13752 | 130538 | 13752 | 0 | 130538 | 0 | 1.000 | 1.000 | 1.000 | 1 |
| **DHC6** | 90 | 86 | 85 | 86 | 0 | 4 | 0.988 | 1.000 | 0.944 | 138640 | 13371 | 125269 | 13518 | 125122 | 13309 | 209 | 125060 | 62 | 0.995 | 0.985 | 0.989 | 1 |
| **DHC7A** | 64 | 64 | 63 | 64 | 0 | 0 | 0.984 | 1.000 | 0.984 | 257605 | 12058 | 245547 | 12073 | 245532 | 12058 | 15 | 245532 | 0 | 1.000 | 0.999 | 0.999 | 1 |
| **DHC7B** | 62 | 60 | 57 | 60 | 0 | 2 | 0.950 | 1.000 | 0.919 | 191156 | 11897 | 179259 | 12168 | 178988 | 11890 | 278 | 178981 | 7 | 0.999 | 0.977 | 0.987 | 1 |
| **DHC7C** | 73 | 73 | 71 | 73 | 0 | 0 | 0.973 | 1.000 | 0.973 | 205577 | 11880 | 193697 | 11880 | 193697 | 11876 | 4 | 193693 | 4 | 1.000 | 1.000 | 1.000 | 1 |
| **DHC8** | 81 | 73 | 64 | 73 | 0 | 8 | 0.877 | 1.000 | 0.790 | 84895 | 12190 | 72705 | 12739 | 72156 | 12189 | 550 | 72155 | 1 | 1.000 | 0.957 | 0.975 | 1 |
| **DHC9A** | 79 | 75 | 70 | 75 | 0 | 4 | 0.933 | 1.000 | 0.886 | 306568 | 12186 | 294382 | 12471 | 294097 | 12184 | 287 | 294095 | 2 | 1.000 | 0.977 | 0.988 | 1 |
| **DHC9B** | 87 | 85 | 77 | 84 | 1 | 3 | 0.906 | 0.988 | 0.885 | 373531 | 13791 | 359740 | 13821 | 359710 | 13650 | 171 | 359569 | 141 | 0.990 | 0.988 | 0.988 | 1 |
| **DHC11** | 43 | 43 | 40 | 43 | 0 | 0 | 0.930 | 1.000 | 0.930 | 85205 | 14337 | 70868 | 14355 | 70850 | 14337 | 18 | 70850 | 0 | 1.000 | 0.999 | 0.999 | 1 |
| **Others** | **91** | **93** | **86** | **91** | **2** | **0** | **0.925** | **0.978** | **0.945** | **108735** | **14186** | **94549** | **12504** | **96231** | **12478** | **26** | **94523** | **1708** | **0.880** | **0.998** | **0.930** | **7** |
| **NedKinesinJ_fl** | 3 | 3 | 2 | 3 | 0 | 0 | 0.667 | 1.000 | 0.667 | 6820 | 2273 | 4547 | 585 | 6235 | 585 | 0 | 4547 | 1688 | 0.257 | 1.000 | 0.493 | 1 |
| **PhsP62_fl** | 3 | 3 | 1 | 3 | 0 | 0 | 0.333 | 1.000 | 0.333 | 5645 | 1506 | 4139 | 1512 | 4133 | 1503 | 9 | 4130 | 3 | 0.998 | 0.994 | 0.995 | 1 |
| **HsP50_fl** | 16 | 16 | 16 | 16 | 0 | 0 | 1.000 | 1.000 | 1.000 | 20372 | 1218 | 19154 | 1218 | 19154 | 1218 | 0 | 19154 | 0 | 1.000 | 1.000 | 1.000 | 1 |
| **PugCor_fl** | 9 | 10 | 8 | 9 | 1 | 0 | 0.800 | 0.900 | 0.889 | 6606 | 1617 | 4989 | 1617 | 4989 | 1612 | 5 | 4984 | 5 | 0.997 | 0.997 | 0.996 | 1 |
| **MmP150_fl** | 32 | 32 | 32 | 32 | 0 | 0 | 1.000 | 1.000 | 1.000 | 23936 | 3843 | 20093 | 3843 | 20093 | 3843 | 0 | 20093 | 0 | 1.000 | 1.000 | 1.000 | 1 |
| **HsMyo19_fl** | 23 | 24 | 22 | 23 | 1 | 0 | 0.917 | 0.958 | 0.957 | 40271 | 2910 | 37361 | 2910 | 37361 | 2898 | 12 | 37349 | 12 | 0.996 | 0.996 | 0.996 | 1 |
| **ThCAP1_fl** | 5 | 5 | 5 | 5 | 0 | 0 | 1.000 | 1.000 | 1.000 | 5085 | 819 | 4266 | 819 | 4266 | 819 | 0 | 4266 | 0 | 1.000 | 1.000 | 1.000 | 1 |

**Prosplign (Blast)**

| | Exon PE | Exon AE | Exon TE | Exon OE | Exon ME | Exon WE | Exon Sn | Exon Sno | Exon Sp | Nucl Total | Nucl AP | Nucl AN | Nucl PP | Nucl PN | Nucl TP | Nucl FP | Nucl TN | Nucl FN | Nucl Sn | Nucl Sp | Nucl AC | Genes |
|---|---|---|---|---|---|---|---|---|---|---|---|---|---|---|---|---|---|---|---|---|---|---|
| **DHC all** | **1242** | **1202** | **1150** | **1201** | **1** | **41** | **0.957** | **0.999** | **0.926** | **3910464** | **209482** | **3700982** | **212058** | **3698406** | **209179** | **2879** | **3698103** | **303** | **0.999** | **0.986** | **0.992** | **16** |
| **DHC1** | 79 | 77 | 77 | 77 | 0 | 2 | 1.000 | 1.000 | 0.975 | 66248 | 13682 | 52566 | 13883 | 52365 | 13682 | 201 | 52365 | 0 | 1.000 | 0.986 | 0.991 | 1 |
| **DHC2** | 93 | 88 | 86 | 88 | 0 | 5 | 0.977 | 1.000 | 0.925 | 417085 | 12702 | 404383 | 12912 | 404173 | 12702 | 210 | 404173 | 0 | 1.000 | 0.984 | 0.992 | 1 |
| **DHC3A** | 93 | 92 | 91 | 92 | 0 | 1 | 0.989 | 1.000 | 0.978 | 362204 | 14070 | 348134 | 14145 | 348059 | 14070 | 75 | 348059 | 0 | 1.000 | 0.995 | 0.997 | 1 |
| **DHC3B** | 79 | 78 | 74 | 78 | 0 | 1 | 0.949 | 1.000 | 0.937 | 302827 | 13745 | 289082 | 13895 | 288932 | 13735 | 160 | 288922 | 10 | 0.999 | 0.988 | 0.994 | 1 |
| **DHC4A** | 82 | 82 | 80 | 82 | 0 | 0 | 0.976 | 1.000 | 0.976 | 365115 | 13523 | 351592 | 13523 | 351592 | 13519 | 4 | 351588 | 4 | 1.000 | 1.000 | 1.000 | 1 |
| **DHC4B** | 82 | 79 | 75 | 79 | 0 | 3 | 0.949 | 1.000 | 0.915 | 119251 | 13284 | 105967 | 13425 | 105826 | 13226 | 199 | 105768 | 58 | 0.996 | 0.985 | 0.989 | 1 |
| **DHC4C** | 76 | 68 | 61 | 68 | 0 | 8 | 0.897 | 1.000 | 0.803 | 490267 | 13014 | 477253 | 13498 | 476769 | 13000 | 498 | 476755 | 14 | 0.999 | 0.963 | 0.980 | 1 |
| **DHC5** | 79 | 79 | 79 | 79 | 0 | 0 | 1.000 | 1.000 | 1.000 | 144290 | 13752 | 130538 | 13752 | 130538 | 13752 | 0 | 130538 | 0 | 1.000 | 1.000 | 1.000 | 1 |
| **DHC6** | 90 | 86 | 85 | 86 | 0 | 4 | 0.988 | 1.000 | 0.944 | 138640 | 13371 | 125269 | 13518 | 125122 | 13309 | 209 | 125060 | 62 | 0.995 | 0.985 | 0.989 | 1 |
| **DHC7A** | 64 | 64 | 63 | 64 | 0 | 0 | 0.984 | 1.000 | 0.984 | 257605 | 12058 | 245547 | 12073 | 245532 | 12058 | 15 | 245532 | 0 | 1.000 | 0.999 | 0.999 | 1 |
| **DHC7B** | 62 | 60 | 57 | 60 | 0 | 2 | 0.950 | 1.000 | 0.919 | 191156 | 11897 | 179259 | 12168 | 178988 | 11890 | 278 | 178981 | 7 | 0.999 | 0.977 | 0.987 | 1 |
| **DHC7C** | 73 | 73 | 71 | 73 | 0 | 0 | 0.973 | 1.000 | 0.973 | 205577 | 11880 | 193697 | 11880 | 193697 | 11876 | 4 | 193693 | 4 | 1.000 | 1.000 | 1.000 | 1 |
| **DHC8** | 81 | 73 | 64 | 73 | 0 | 8 | 0.877 | 1.000 | 0.790 | 84895 | 12190 | 72705 | 12739 | 72156 | 12189 | 550 | 72155 | 1 | 1.000 | 0.957 | 0.975 | 1 |
| **DHC9A** | 79 | 75 | 70 | 75 | 0 | 4 | 0.933 | 1.000 | 0.886 | 306568 | 12186 | 294382 | 12471 | 294097 | 12184 | 287 | 294095 | 2 | 1.000 | 0.977 | 0.988 | 1 |
| **DHC9B** | 87 | 85 | 77 | 84 | 1 | 3 | 0.906 | 0.988 | 0.885 | 373531 | 13791 | 359740 | 13821 | 359710 | 13650 | 171 | 359569 | 141 | 0.990 | 0.988 | 0.988 | 1 |
| **DHC11** | 43 | 43 | 40 | 43 | 0 | 0 | 0.930 | 1.000 | 0.930 | 85205 | 14337 | 70868 | 14355 | 70850 | 14337 | 18 | 70850 | 0 | 1.000 | 0.999 | 0.999 | 1 |
| **Others** | **91** | **93** | **86** | **91** | **2** | **0** | **0.925** | **0.978** | **0.945** | **108735** | **14186** | **94549** | **12504** | **96231** | **12478** | **26** | **94523** | **1708** | **0.880** | **0.998** | **0.930** | **7** |
| **NedKinesinJ_fl** | 3 | 3 | 2 | 3 | 0 | 0 | 0.667 | 1.000 | 0.667 | 6820 | 2273 | 4547 | 585 | 6235 | 585 | 0 | 4547 | 1688 | 0.257 | 1.000 | 0.493 | 1 |
| **PhsP62_fl** | 3 | 3 | 1 | 3 | 0 | 0 | 0.333 | 1.000 | 0.333 | 5645 | 1506 | 4139 | 1512 | 4133 | 1503 | 9 | 4130 | 3 | 0.998 | 0.994 | 0.995 | 1 |
| **HsP50_fl** | 16 | 16 | 16 | 16 | 0 | 0 | 1.000 | 1.000 | 1.000 | 20372 | 1218 | 19154 | 1218 | 19154 | 1218 | 0 | 19154 | 0 | 1.000 | 1.000 | 1.000 | 1 |
| **PugCor_fl** | 9 | 10 | 8 | 9 | 1 | 0 | 0.800 | 0.900 | 0.889 | 6606 | 1617 | 4989 | 1617 | 4989 | 1612 | 5 | 4984 | 5 | 0.997 | 0.997 | 0.996 | 1 |
| **MmP150_fl** | 32 | 32 | 32 | 32 | 0 | 0 | 1.000 | 1.000 | 1.000 | 23936 | 3843 | 20093 | 3843 | 20093 | 3843 | 0 | 20093 | 0 | 1.000 | 1.000 | 1.000 | 1 |
| **HsMyo19_fl** | 23 | 24 | 22 | 23 | 1 | 0 | 0.917 | 0.958 | 0.957 | 40271 | 2910 | 37361 | 2910 | 37361 | 2898 | 12 | 37349 | 12 | 0.996 | 0.996 | 0.996 | 1 |
| **ThCAP1_fl** | 5 | 5 | 5 | 5 | 0 | 0 | 1.000 | 1.000 | 1.000 | 5085 | 819 | 4266 | 819 | 4266 | 819 | 0 | 4266 | 0 | 1.000 | 1.000 | 1.000 | 1 |

**Prosplign (Blat)**

| | Exon PE | Exon AE | Exon TE | Exon OE | Exon ME | Exon WE | Exon Sn | Exon Sno | Exon Sp | Nucl Total | Nucl AP | Nucl AN | Nucl PP | Nucl PN | Nucl TP | Nucl FP | Nucl TN | Nucl FN | Nucl Sn | Nucl Sp | Nucl AC | Genes |
|---|---|---|---|---|---|---|---|---|---|---|---|---|---|---|---|---|---|---|---|---|---|---|
| **DHC all** | **1244** | **1202** | **1148** | **1199** | **3** | **45** | **0.955** | **0.998** | **0.923** | **3910464** | **209482** | **3700982** | **211838** | **3698626** | **208781** | **3057** | **3697925** | **701** | **0.997** | **0.986** | **0.991** | **17** |
| **DHC1** | 79 | 77 | 77 | 77 | 0 | 2 | 1.000 | 1.000 | 0.975 | 66248 | 13682 | 52566 | 13738 | 52510 | 13682 | 56 | 52510 | 0 | 1.000 | 0.996 | 0.997 | 1 |
| **DHC2** | 93 | 88 | 86 | 88 | 0 | 5 | 0.977 | 1.000 | 0.925 | 417085 | 12702 | 404383 | 12912 | 404173 | 12702 | 210 | 404173 | 0 | 1.000 | 0.984 | 0.992 | 1 |
| **DHC3A** | 93 | 92 | 91 | 92 | 0 | 1 | 0.989 | 1.000 | 0.978 | 362204 | 14070 | 348134 | 14145 | 348059 | 14070 | 75 | 348059 | 0 | 1.000 | 0.995 | 0.997 | 1 |
| **DHC3B** | 79 | 78 | 74 | 78 | 0 | 1 | 0.949 | 1.000 | 0.937 | 302827 | 13745 | 289082 | 13895 | 288932 | 13735 | 160 | 288922 | 10 | 0.999 | 0.988 | 0.994 | 1 |
| **DHC4A** | 82 | 82 | 80 | 82 | 0 | 0 | 0.976 | 1.000 | 0.976 | 365115 | 13523 | 351592 | 13523 | 351592 | 13519 | 4 | 351588 | 4 | 1.000 | 1.000 | 1.000 | 1 |
| **DHC4B** | 82 | 79 | 75 | 79 | 0 | 3 | 0.949 | 1.000 | 0.915 | 119251 | 13284 | 105967 | 13425 | 105826 | 13226 | 199 | 105768 | 58 | 0.996 | 0.985 | 0.989 | 1 |
| **DHC4C** | 76 | 68 | 61 | 68 | 0 | 8 | 0.897 | 1.000 | 0.803 | 490267 | 13014 | 477253 | 13498 | 476769 | 13000 | 498 | 476755 | 14 | 0.999 | 0.963 | 0.980 | 1 |
| **DHC5** | 79 | 79 | 79 | 79 | 0 | 0 | 1.000 | 1.000 | 1.000 | 144290 | 13752 | 130538 | 13752 | 130538 | 13752 | 0 | 130538 | 0 | 1.000 | 1.000 | 1.000 | 1 |
| **DHC6** | 89 | 86 | 84 | 85 | 1 | 4 | 0.977 | 0.988 | 0.944 | 138640 | 13371 | 125269 | 13437 | 125203 | 13136 | 301 | 124968 | 235 | 0.982 | 0.978 | 0.978 | 2 |
| **DHC7A** | 64 | 64 | 63 | 64 | 0 | 0 | 0.984 | 1.000 | 0.984 | 257605 | 12058 | 245547 | 12073 | 245532 | 12058 | 15 | 245532 | 0 | 1.000 | 0.999 | 0.999 | 1 |

| | | | | | | | | | | | | | | | | | | | | | | |
|---|---|---|---|---|---|---|---|---|---|---|---|---|---|---|---|---|---|---|---|---|---|---|
| **DHC7B** | 62 | 60 | 57 | 60 | 0 | 2 | 0.950 | 1.000 | 0.919 | 191156 | 11897 | 179259 | 12168 | 178988 | 11890 | 278 | 178981 | 7 | 0.999 | 0.977 | 0.987 | 1 |
| **DHC7C** | 73 | 73 | 71 | 73 | 0 | 0 | 0.973 | 1.000 | 0.973 | 205577 | 11880 | 193697 | 11880 | 193697 | 11876 | 4 | 193693 | 4 | 1.000 | 1.000 | 1.000 | 1 |
| **DHC8** | 81 | 73 | 64 | 73 | 0 | 8 | 0.877 | 1.000 | 0.790 | 84895 | 12190 | 72705 | 12739 | 72156 | 12189 | 550 | 72155 | 1 | 1.000 | 0.957 | 0.975 | 1 |
| **DHC9A** | 82 | 75 | 69 | 74 | 1 | 8 | 0.920 | 0.987 | 0.841 | 306568 | 12186 | 294382 | 12477 | 294091 | 11959 | 518 | 293864 | 227 | 0.981 | 0.958 | 0.969 | 1 |
| **DHC9B** | 87 | 85 | 77 | 84 | 1 | 3 | 0.906 | 0.988 | 0.885 | 373531 | 13791 | 359740 | 13821 | 359710 | 13650 | 171 | 359569 | 141 | 0.990 | 0.988 | 0.988 | 1 |
| **DHC11** | 43 | 43 | 40 | 43 | 0 | 0 | 0.930 | 1.000 | 0.930 | 85205 | 14337 | 70868 | 14355 | 70850 | 14337 | 18 | 70850 | 0 | 1.000 | 0.999 | 0.999 | 1 |
| **Others** | **90** | **93** | **84** | **90** | **3** | **0** | **0.903** | **0.968** | **0.933** | **108735** | **14186** | **94549** | **14992** | **93743** | **12446** | **2546** | **92003** | **1740** | **0.877** | **0.830** | **0.831** | **7** |
| **NedKinesinJ_fl** | 3 | 3 | 2 | 3 | 0 | 0 | 0.667 | 1.000 | 0.667 | 6820 | 2273 | 4547 | 3040 | 3780 | 586 | 2454 | 2093 | 1687 | 0.258 | 0.193 | -0.268 | 1 |
| **PhsP62_fl** | 3 | 3 | 1 | 3 | 0 | 0 | 0.333 | 1.000 | 0.333 | 5645 | 1506 | 4139 | 1512 | 4133 | 1503 | 9 | 4130 | 3 | 0.998 | 0.994 | 0.995 | 1 |
| **HsP50_fl** | 16 | 16 | 16 | 16 | 0 | 0 | 1.000 | 1.000 | 1.000 | 20372 | 1218 | 19154 | 1218 | 19154 | 1218 | 0 | 19154 | 0 | 1.000 | 1.000 | 1.000 | 1 |
| **PugCor_fl** | 9 | 10 | 8 | 9 | 1 | 0 | 0.800 | 0.900 | 0.889 | 6606 | 1617 | 4989 | 1617 | 4989 | 1612 | 5 | 4984 | 5 | 0.997 | 0.997 | 0.996 | 1 |
| **MmP150_fl** | 31 | 32 | 30 | 31 | 1 | 0 | 0.938 | 0.969 | 0.968 | 23936 | 3843 | 20093 | 3876 | 20060 | 3810 | 66 | 20027 | 33 | 0.991 | 0.983 | 0.985 | 1 |
| **HsMyo19_fl** | 23 | 24 | 22 | 23 | 1 | 0 | 0.917 | 0.958 | 0.957 | 40271 | 2910 | 37361 | 2910 | 37361 | 2898 | 12 | 37349 | 12 | 0.996 | 0.996 | 0.996 | 1 |
| **ThCAP1_fl** | 5 | 5 | 5 | 5 | 0 | 0 | 1.000 | 1.000 | 1.000 | 5085 | 819 | 4266 | 819 | 4266 | 819 | 0 | 4266 | 0 | 1.000 | 1.000 | 1.000 | 1 |

**Exonerate**

| | Exon PE | Exon AE | Exon TE | Exon OE | Exon ME | Exon WE | Exon Sn | Exon Sno | Exon Sp | Nucl Total | Nucl AP | Nucl AN | Nucl PP | Nucl PN | Nucl TP | Nucl FP | Nucl TN | Nucl FN | Nucl Sn | Nucl Sp | Nucl AC | Genes |
|---|---|---|---|---|---|---|---|---|---|---|---|---|---|---|---|---|---|---|---|---|---|---|
| **DHC all** | **1205** | **1202** | **1140** | **1195** | **7** | **6** | **0.948** | **0.994** | **0.946** | **3910464** | **209483** | **3700981** | **209870** | **3700594** | **208717** | **1153** | **3699828** | **766** | **0.996** | **0.995** | **0.995** | **32** |
| **DHC1** | 77 | 77 | 77 | 77 | 0 | 0 | 1.000 | 1.000 | 1.000 | 66248 | 13683 | 52565 | 13683 | 52565 | 13683 | 0 | 52565 | 0 | 1.000 | 1.000 | 1.000 | 1 |
| **DHC2** | 90 | 88 | 86 | 88 | 0 | 2 | 0.977 | 1.000 | 0.956 | 417085 | 12702 | 404383 | 12846 | 404239 | 12702 | 144 | 404239 | 0 | 1.000 | 0.989 | 0.994 | 2 |
| **DHC3A** | 92 | 92 | 91 | 92 | 0 | 0 | 0.989 | 1.000 | 0.989 | 362204 | 14070 | 348134 | 14013 | 348191 | 14013 | 0 | 348134 | 57 | 0.996 | 1.000 | 0.998 | 1 |
| **DHC3B** | 78 | 78 | 75 | 78 | 0 | 0 | 0.962 | 1.000 | 0.962 | 302827 | 13745 | 289082 | 13763 | 289064 | 13745 | 18 | 289064 | 0 | 1.000 | 0.999 | 0.999 | 2 |
| **DHC4A** | 82 | 82 | 82 | 82 | 0 | 0 | 1.000 | 1.000 | 1.000 | 365115 | 13523 | 351592 | 13523 | 351592 | 13523 | 0 | 351592 | 0 | 1.000 | 1.000 | 1.000 | 1 |
| **DHC4B** | 79 | 79 | 74 | 79 | 0 | 0 | 0.937 | 1.000 | 0.937 | 119251 | 13284 | 105967 | 13278 | 105973 | 13265 | 13 | 105954 | 19 | 0.999 | 0.999 | 0.999 | 2 |
| **DHC4C** | 71 | 68 | 62 | 68 | 0 | 2 | 0.912 | 1.000 | 0.873 | 490267 | 13014 | 477253 | 13138 | 477129 | 13014 | 124 | 477129 | 0 | 1.000 | 0.991 | 0.995 | 3 |
| **DHC5** | 79 | 79 | 78 | 79 | 0 | 0 | 0.987 | 1.000 | 0.987 | 144290 | 13752 | 130538 | 13767 | 130523 | 13752 | 15 | 130523 | 0 | 1.000 | 0.999 | 0.999 | 1 |
| **DHC6** | 87 | 86 | 83 | 85 | 1 | 1 | 0.965 | 0.988 | 0.954 | 138640 | 13371 | 125269 | 13605 | 125035 | 13305 | 300 | 124969 | 66 | 0.995 | 0.978 | 0.985 | 3 |
| **DHC7A** | 64 | 64 | 63 | 64 | 0 | 0 | 0.984 | 1.000 | 0.984 | 257605 | 12058 | 245547 | 12067 | 245538 | 12058 | 9 | 245538 | 0 | 1.000 | 0.999 | 1.000 | 1 |
| **DHC7B** | 60 | 60 | 55 | 59 | 1 | 1 | 0.917 | 0.983 | 0.917 | 191156 | 11897 | 179259 | 11913 | 179243 | 11812 | 101 | 179158 | 85 | 0.993 | 0.992 | 0.992 | 3 |
| **DHC7C** | 72 | 73 | 68 | 72 | 1 | 0 | 0.932 | 0.986 | 0.944 | 205577 | 11880 | 193697 | 11880 | 193697 | 11849 | 31 | 193666 | 31 | 0.997 | 0.997 | 0.997 | 1 |
| **DHC8** | 73 | 73 | 65 | 73 | 0 | 0 | 0.890 | 1.000 | 0.890 | 84895 | 12190 | 72705 | 12158 | 72737 | 12067 | 91 | 72614 | 123 | 0.990 | 0.993 | 0.990 | 4 |
| **DHC9A** | 75 | 75 | 71 | 75 | 0 | 0 | 0.947 | 1.000 | 0.947 | 306568 | 12186 | 294382 | 12228 | 294340 | 12186 | 42 | 294340 | 0 | 1.000 | 0.997 | 0.998 | 3 |
| **DHC9B** | 83 | 85 | 72 | 82 | 3 | 0 | 0.847 | 0.965 | 0.867 | 373531 | 13791 | 359740 | 13713 | 359818 | 13500 | 213 | 359527 | 291 | 0.979 | 0.984 | 0.981 | 3 |
| **DHC11** | 43 | 43 | 38 | 42 | 1 | 0 | 0.884 | 0.977 | 0.884 | 85205 | 14337 | 70868 | 14295 | 70910 | 14243 | 52 | 70816 | 94 | 0.993 | 0.996 | 0.994 | 1 |
| **Others** | **88** | **93** | **77** | **87** | **6** | **0** | **0.828** | **0.935** | **0.875** | **108735** | **14186** | **94549** | **14168** | **94567** | **14107** | **61** | **94488** | **79** | **0.994** | **0.996** | **0.994** | **8** |
| **NedKinesinJ_fl** | 4 | 3 | 2 | 3 | 0 | 0 | 0.667 | 1.000 | 0.500 | 6820 | 2273 | 4547 | 2273 | 4547 | 2273 | 0 | 4547 | 0 | 1.000 | 1.000 | 1.000 | 2 |
| **PhsP62_fl** | 3 | 3 | 1 | 3 | 0 | 0 | 0.333 | 1.000 | 0.333 | 5645 | 1506 | 4139 | 1494 | 4151 | 1494 | 0 | 4139 | 12 | 0.992 | 1.000 | 0.995 | 1 |
| **HsP50_fl** | 14 | 16 | 13 | 14 | 2 | 0 | 0.813 | 0.875 | 0.929 | 20372 | 1218 | 19154 | 1218 | 19154 | 1203 | 15 | 19139 | 15 | 0.988 | 0.988 | 0.987 | 1 |
| **PugCor_fl** | 8 | 10 | 4 | 8 | 2 | 0 | 0.400 | 0.800 | 0.500 | 6606 | 1617 | 4989 | 1614 | 4992 | 1596 | 18 | 4971 | 21 | 0.987 | 0.989 | 0.984 | 1 |
| **MmP150_fl** | 32 | 32 | 32 | 32 | 0 | 0 | 1.000 | 1.000 | 1.000 | 23936 | 3843 | 20093 | 3843 | 20093 | 3843 | 0 | 20093 | 0 | 1.000 | 1.000 | 1.000 | 1 |
| **HsMyo19_fl** | 23 | 24 | 22 | 23 | 1 | 0 | 0.917 | 0.958 | 0.957 | 40271 | 2910 | 37361 | 2907 | 37364 | 2898 | 9 | 37352 | 12 | 0.996 | 0.997 | 0.996 | 1 |
| **ThCAP1_fl** | 4 | 5 | 3 | 4 | 1 | 0 | 0.600 | 0.800 | 0.750 | 5085 | 819 | 4266 | 819 | 4266 | 800 | 19 | 4247 | 19 | 0.977 | 0.977 | 0.972 | 1 |

**Exonerate (1 gene)**

| | Exon PE | Exon AE | Exon TE | Exon OE | Exon ME | Exon WE | Exon Sn | Exon Sno | Exon Sp | Nucl Total | Nucl AP | Nucl AN | Nucl PP | Nucl PN | Nucl TP | Nucl FP | Nucl TN | Nucl FN | Nucl Sn | Nucl Sp | Nucl AC | Genes |
|---|---|---|---|---|---|---|---|---|---|---|---|---|---|---|---|---|---|---|---|---|---|---|
| **DHC all** | **952** | **1202** | **910** | **947** | **255** | **4** | **0.757** | **0.788** | **0.956** | **3910464** | **209483** | **3700981** | **166336** | **3744128** | **165824** | **512** | **3700469** | **43659** | **0.792** | **0.997** | **0.888** | **16** |
| **DHC1** | 77 | 77 | 77 | 77 | 0 | 0 | 1.000 | 1.000 | 1.000 | 66248 | 13683 | 52565 | 13683 | 52565 | 13683 | 0 | 52565 | 0 | 1.000 | 1.000 | 1.000 | 1 |
| **DHC2** | 53 | 88 | 50 | 51 | 37 | 2 | 0.568 | 0.580 | 0.943 | 417085 | 12702 | 404383 | 8382 | 408703 | 8310 | 72 | 404311 | 4392 | 0.654 | 0.991 | 0.817 | 1 |
| **DHC3A** | 92 | 92 | 91 | 92 | 0 | 0 | 0.989 | 1.000 | 0.989 | 362204 | 14070 | 348134 | 14013 | 348191 | 14013 | 0 | 348134 | 57 | 0.996 | 1.000 | 0.998 | 1 |
| **DHC3B** | 61 | 78 | 59 | 61 | 17 | 0 | 0.756 | 0.782 | 0.967 | 302827 | 13745 | 289082 | 10439 | 292388 | 10428 | 11 | 289071 | 3317 | 0.759 | 0.999 | 0.873 | 1 |
| **DHC4A** | 82 | 82 | 82 | 82 | 0 | 0 | 1.000 | 1.000 | 1.000 | 365115 | 13523 | 351592 | 13523 | 351592 | 13523 | 0 | 351592 | 0 | 1.000 | 1.000 | 1.000 | 1 |
| **DHC4B** | 49 | 79 | 45 | 49 | 30 | 0 | 0.570 | 0.620 | 0.918 | 119251 | 13284 | 105967 | 7881 | 111370 | 7871 | 10 | 105957 | 5413 | 0.593 | 0.999 | 0.771 | 1 |
| **DHC4C** | 33 | 68 | 28 | 32 | 36 | 1 | 0.412 | 0.471 | 0.848 | 490267 | 13014 | 477253 | 6382 | 483885 | 6322 | 60 | 477193 | 6692 | 0.486 | 0.991 | 0.731 | 1 |
| **DHC5** | 79 | 79 | 78 | 79 | 0 | 0 | 0.987 | 1.000 | 0.987 | 144290 | 13752 | 130538 | 13767 | 130523 | 13752 | 15 | 130523 | 0 | 1.000 | 0.999 | 0.999 | 1 |
| **DHC6** | 48 | 86 | 46 | 48 | 38 | 0 | 0.535 | 0.558 | 0.958 | 138640 | 13371 | 125269 | 7623 | 131017 | 7599 | 24 | 125245 | 5772 | 0.568 | 0.997 | 0.760 | 1 |
| **DHC7A** | 64 | 64 | 63 | 64 | 0 | 0 | 0.984 | 1.000 | 0.984 | 257605 | 12058 | 245547 | 12067 | 245538 | 12058 | 9 | 245538 | 0 | 1.000 | 0.999 | 1.000 | 1 |
| **DHC7B** | 43 | 60 | 41 | 42 | 18 | 1 | 0.683 | 0.700 | 0.953 | 191156 | 11897 | 179259 | 6537 | 184619 | 6448 | 89 | 179170 | 5449 | 0.542 | 0.986 | 0.749 | 1 |
| **DHC7C** | 72 | 73 | 68 | 72 | 1 | 0 | 0.932 | 0.986 | 0.944 | 205577 | 11880 | 193697 | 11880 | 193697 | 11849 | 31 | 193666 | 31 | 0.997 | 0.997 | 0.997 | 1 |
| **DHC8** | 62 | 73 | 57 | 62 | 11 | 0 | 0.781 | 0.849 | 0.919 | 84895 | 12190 | 72705 | 10165 | 74730 | 10092 | 73 | 72632 | 2098 | 0.828 | 0.993 | 0.896 | 1 |
| **DHC9A** | 50 | 75 | 48 | 50 | 25 | 0 | 0.640 | 0.667 | 0.960 | 306568 | 12186 | 294382 | 8364 | 298204 | 8357 | 7 | 294375 | 3829 | 0.686 | 0.999 | 0.836 | 1 |

| | | | | | | | | | | | | | | | | | | | | | | |
|---|---|---|---|---|---|---|---|---|---|---|---|---|---|---|---|---|---|---|---|---|---|---|
| **DHC9B** | 44 | 85 | 39 | 44 | 41 | 0 | 0.459 | 0.518 | 0.886 | 373531 | 13791 | 359740 | 7335 | 366196 | 7276 | 59 | 359681 | 6515 | 0.528 | 0.992 | 0.751 | 1 |
| **DHC11** | 43 | 43 | 38 | 42 | 1 | 0 | 0.884 | 0.977 | 0.884 | 85205 | 14337 | 70868 | 14295 | 70910 | 14243 | 52 | 70816 | 94 | 0.993 | 0.996 | 0.994 | 1 |
| **Others** | **87** | **93** | **77** | **87** | **6** | **0** | **0.828** | **0.935** | **0.885** | **108735** | **14186** | **94549** | **14090** | **94645** | **14029** | **61** | **94488** | **157** | **0.989** | **0.996** | **0.991** | **7** |
| **NedKinesinJ_fl** | 3 | 3 | 2 | 3 | 0 | 0 | 0.667 | 1.000 | 0.667 | 6820 | 2273 | 4547 | 2195 | 4625 | 2195 | 0 | 4547 | 78 | 0.966 | 1.000 | 0.974 | 1 |
| **PhsP62_fl** | 3 | 3 | 1 | 3 | 0 | 0 | 0.333 | 1.000 | 0.333 | 5645 | 1506 | 4139 | 1494 | 4151 | 1494 | 0 | 4139 | 12 | 0.992 | 1.000 | 0.995 | 1 |
| **HsP50_fl** | 14 | 16 | 13 | 14 | 2 | 0 | 0.813 | 0.875 | 0.929 | 20372 | 1218 | 19154 | 1218 | 19154 | 1203 | 15 | 19139 | 15 | 0.988 | 0.988 | 0.987 | 1 |
| **PugCor_fl** | 8 | 10 | 4 | 8 | 2 | 0 | 0.400 | 0.800 | 0.500 | 6606 | 1617 | 4989 | 1614 | 4992 | 1596 | 18 | 4971 | 21 | 0.987 | 0.989 | 0.984 | 1 |
| **MmP150_fl** | 32 | 32 | 32 | 32 | 0 | 0 | 1.000 | 1.000 | 1.000 | 23936 | 3843 | 20093 | 3843 | 20093 | 3843 | 0 | 20093 | 0 | 1.000 | 1.000 | 1.000 | 1 |
| **HsMyo19_fl** | 23 | 24 | 22 | 23 | 1 | 0 | 0.917 | 0.958 | 0.957 | 40271 | 2910 | 37361 | 2907 | 37364 | 2898 | 9 | 37352 | 12 | 0.996 | 0.997 | 0.996 | 1 |
| **ThCAP1_fl** | 4 | 5 | 3 | 4 | 1 | 0 | 0.600 | 0.800 | 0.750 | 5085 | 819 | 4266 | 819 | 4266 | 800 | 19 | 4247 | 19 | 0.977 | 0.977 | 0.972 | 1 |

**Prot_map**

| | Exon PE | Exon AE | Exon TE | Exon OE | Exon ME | Exon WE | Exon Sn | Exon Sno | Exon Sp | Nucl Total | Nucl AP | Nucl AN | Nucl PP | Nucl PN | Nucl TP | Nucl FP | Nucl TN | Nucl FN | Nucl Sn | Nucl Sp | Nucl AC | Genes |
|---|---|---|---|---|---|---|---|---|---|---|---|---|---|---|---|---|---|---|---|---|---|---|
| **DHC all** | **1278** | **1202** | **1102** | **1198** | **4** | **27** | **0.917** | **0.997** | **0.862** | **3910464** | **209482** | **3700982** | **208618** | **3701846** | **208031** | **587** | **3700395** | **1451** | **0.993** | **0.997** | **0.995** | **16** |
| **DHC1** | 78 | 77 | 76 | 77 | 0 | 1 | 0.987 | 1.000 | 0.974 | 66248 | 13682 | 52566 | 13716 | 52532 | 13682 | 34 | 52532 | 0 | 1.000 | 0.998 | 0.998 | 1 |
| **DHC2** | 89 | 88 | 84 | 88 | 0 | 1 | 0.955 | 1.000 | 0.944 | 417085 | 12702 | 404383 | 12717 | 404368 | 12686 | 31 | 404352 | 16 | 0.999 | 0.998 | 0.998 | 1 |
| **DHC3A** | 95 | 92 | 88 | 92 | 0 | 0 | 0.957 | 1.000 | 0.926 | 362204 | 14070 | 348134 | 13998 | 348206 | 13995 | 3 | 348131 | 75 | 0.995 | 1.000 | 0.997 | 1 |
| **DHC3B** | 81 | 78 | 74 | 78 | 0 | 0 | 0.949 | 1.000 | 0.914 | 302827 | 13745 | 289082 | 13712 | 289115 | 13709 | 3 | 289079 | 36 | 0.997 | 1.000 | 0.999 | 1 |
| **DHC4A** | 84 | 82 | 78 | 82 | 0 | 0 | 0.951 | 1.000 | 0.929 | 365115 | 13523 | 351592 | 13508 | 351607 | 13505 | 3 | 351589 | 18 | 0.999 | 1.000 | 0.999 | 1 |
| **DHC4B** | 86 | 79 | 72 | 79 | 0 | 2 | 0.911 | 1.000 | 0.837 | 119251 | 13284 | 105967 | 13261 | 105990 | 13229 | 32 | 105935 | 55 | 0.996 | 0.998 | 0.996 | 1 |
| **DHC4C** | 77 | 68 | 61 | 68 | 0 | 3 | 0.897 | 1.000 | 0.792 | 490267 | 13014 | 477253 | 13044 | 477223 | 12981 | 63 | 477190 | 33 | 0.997 | 0.995 | 0.996 | 1 |
| **DHC5** | 80 | 79 | 75 | 79 | 0 | 0 | 0.949 | 1.000 | 0.938 | 144290 | 13752 | 130538 | 13701 | 130589 | 13692 | 9 | 130529 | 60 | 0.996 | 0.999 | 0.997 | 1 |
| **DHC6** | 88 | 86 | 83 | 86 | 0 | 0 | 0.965 | 1.000 | 0.943 | 138640 | 13371 | 125269 | 13204 | 125436 | 13201 | 3 | 125266 | 170 | 0.987 | 1.000 | 0.993 | 1 |
| **DHC7A** | 65 | 64 | 61 | 64 | 0 | 0 | 0.953 | 1.000 | 0.938 | 257605 | 12058 | 245547 | 12063 | 245542 | 12057 | 6 | 245541 | 1 | 1.000 | 1.000 | 1.000 | 1 |
| **DHC7B** | 63 | 60 | 52 | 59 | 1 | 1 | 0.867 | 0.983 | 0.825 | 191156 | 11897 | 179259 | 11854 | 179302 | 11801 | 53 | 179206 | 96 | 0.992 | 0.996 | 0.993 | 1 |
| **DHC7C** | 73 | 73 | 70 | 73 | 0 | 0 | 0.959 | 1.000 | 0.959 | 205577 | 11880 | 193697 | 11883 | 193694 | 11876 | 7 | 193690 | 4 | 1.000 | 0.999 | 1.000 | 1 |
| **DHC8** | 90 | 73 | 61 | 73 | 0 | 14 | 0.836 | 1.000 | 0.678 | 84895 | 12190 | 72705 | 12246 | 72649 | 12029 | 217 | 72488 | 161 | 0.987 | 0.982 | 0.982 | 1 |
| **DHC9A** | 78 | 75 | 71 | 75 | 0 | 0 | 0.947 | 1.000 | 0.910 | 306568 | 12186 | 294382 | 12118 | 294450 | 12115 | 3 | 294379 | 71 | 0.994 | 1.000 | 0.997 | 1 |
| **DHC9B** | 98 | 85 | 64 | 83 | 2 | 1 | 0.753 | 0.976 | 0.653 | 373531 | 13791 | 359740 | 13353 | 360178 | 13307 | 46 | 359694 | 484 | 0.965 | 0.997 | 0.980 | 1 |
| **DHC11** | 53 | 43 | 32 | 42 | 1 | 4 | 0.744 | 0.977 | 0.604 | 85205 | 14337 | 70868 | 14240 | 70965 | 14166 | 74 | 70794 | 171 | 0.988 | 0.995 | 0.990 | 1 |
| **Others** | **96** | **93** | **74** | **88** | **5** | **3** | **0.796** | **0.946** | **0.771** | **108735** | **14186** | **94549** | **14134** | **94601** | **14043** | **91** | **94458** | **143** | **0.990** | **0.994** | **0.991** | **7** |
| **NedKinesinJ_fl** | 6 | 3 | 2 | 3 | 0 | 0 | 0.667 | 1.000 | 0.333 | 6820 | 2273 | 4547 | 2259 | 4561 | 2256 | 3 | 4544 | 17 | 0.993 | 0.999 | 0.993 | 1 |
| **PhsP62_fl** | 8 | 3 | 0 | 3 | 0 | 3 | 0.000 | 1.000 | 0.000 | 5645 | 1506 | 4139 | 1490 | 4155 | 1444 | 46 | 4093 | 62 | 0.959 | 0.969 | 0.951 | 1 |
| **HsP50_fl** | 14 | 16 | 13 | 14 | 2 | 0 | 0.813 | 0.875 | 0.929 | 20372 | 1218 | 19154 | 1206 | 19166 | 1203 | 3 | 19151 | 15 | 0.988 | 0.998 | 0.992 | 1 |
| **PugCor_fl** | 9 | 10 | 6 | 9 | 1 | 0 | 0.600 | 0.900 | 0.667 | 6606 | 1617 | 4989 | 1609 | 4997 | 1604 | 5 | 4984 | 13 | 0.992 | 0.997 | 0.993 | 1 |
| **MmP150_fl** | 31 | 32 | 29 | 31 | 1 | 0 | 0.906 | 0.969 | 0.935 | 23936 | 3843 | 20093 | 3834 | 20102 | 3828 | 6 | 20087 | 15 | 0.996 | 0.998 | 0.997 | 1 |
| **HsMyo19_fl** | 24 | 24 | 22 | 24 | 0 | 0 | 0.917 | 1.000 | 0.917 | 40271 | 2910 | 37361 | 2916 | 37355 | 2910 | 6 | 37355 | 0 | 1.000 | 0.998 | 0.999 | 1 |
| **ThCAP1_fl** | 4 | 5 | 2 | 4 | 1 | 0 | 0.400 | 0.800 | 0.500 | 5085 | 819 | 4266 | 820 | 4265 | 798 | 22 | 4244 | 21 | 0.974 | 0.973 | 0.969 | 1 |

**Fgenesh+**

| | Exon PE | Exon AE | Exon TE | Exon OE | Exon ME | Exon WE | Exon Sn | Exon Sno | Exon Sp | Nucl Total | Nucl AP | Nucl AN | Nucl PP | Nucl PN | Nucl TP | Nucl FP | Nucl TN | Nucl FN | Nucl Sn | Nucl Sp | Nucl AC | Genes |
|---|---|---|---|---|---|---|---|---|---|---|---|---|---|---|---|---|---|---|---|---|---|---|
| **DHC all** | **1203** | **1202** | **1141** | **1192** | **10** | **10** | **0.949** | **0.992** | **0.948** | **3910464** | **209482** | **3700982** | **207914** | **3702550** | **207321** | **593** | **3700389** | **2161** | **0.990** | **0.997** | **0.993** | **16** |
| **DHC1** | 78 | 77 | 76 | 77 | 0 | 1 | 0.987 | 1.000 | 0.974 | 66248 | 13682 | 52566 | 13740 | 52508 | 13682 | 58 | 52508 | 0 | 1.000 | 0.996 | 0.997 | 1 |
| **DHC2** | 90 | 88 | 85 | 88 | 0 | 2 | 0.966 | 1.000 | 0.944 | 417085 | 12702 | 404383 | 12717 | 404368 | 12702 | 15 | 404368 | 0 | 1.000 | 0.999 | 0.999 | 1 |
| **DHC3A** | 92 | 92 | 91 | 92 | 0 | 0 | 0.989 | 1.000 | 0.989 | 362204 | 14070 | 348134 | 14073 | 348131 | 14070 | 3 | 348131 | 0 | 1.000 | 1.000 | 1.000 | 1 |
| **DHC3B** | 79 | 78 | 75 | 78 | 0 | 1 | 0.962 | 1.000 | 0.949 | 302827 | 13745 | 289082 | 13659 | 289168 | 13652 | 7 | 289075 | 93 | 0.993 | 0.999 | 0.996 | 1 |
| **DHC4A** | 82 | 82 | 78 | 82 | 0 | 0 | 0.951 | 1.000 | 0.951 | 365115 | 13523 | 351592 | 13497 | 351618 | 13490 | 7 | 351585 | 33 | 0.998 | 0.999 | 0.998 | 1 |
| **DHC4B** | 77 | 79 | 74 | 77 | 2 | 0 | 0.937 | 0.975 | 0.961 | 119251 | 13284 | 105967 | 12972 | 106279 | 12969 | 3 | 105964 | 315 | 0.976 | 1.000 | 0.987 | 1 |
| **DHC4C** | 67 | 68 | 61 | 65 | 3 | 2 | 0.897 | 0.956 | 0.910 | 490267 | 13014 | 477253 | 12275 | 477992 | 12104 | 171 | 477082 | 910 | 0.930 | 0.986 | 0.957 | 1 |
| **DHC5** | 79 | 79 | 78 | 79 | 0 | 0 | 0.987 | 1.000 | 0.987 | 144290 | 13752 | 130538 | 13755 | 130535 | 13752 | 3 | 130535 | 0 | 1.000 | 1.000 | 1.000 | 1 |
| **DHC6** | 85 | 86 | 83 | 85 | 1 | 0 | 0.965 | 0.988 | 0.976 | 138640 | 13371 | 125269 | 13335 | 125305 | 13309 | 26 | 125243 | 62 | 0.995 | 0.998 | 0.996 | 1 |
| **DHC7A** | 65 | 64 | 60 | 64 | 0 | 0 | 0.938 | 1.000 | 0.923 | 257605 | 12058 | 245547 | 12012 | 245593 | 12003 | 9 | 245538 | 55 | 0.995 | 0.999 | 0.997 | 1 |
| **DHC7B** | 61 | 60 | 57 | 60 | 0 | 1 | 0.950 | 1.000 | 0.934 | 191156 | 11897 | 179259 | 11931 | 179225 | 11883 | 48 | 179211 | 14 | 0.999 | 0.996 | 0.997 | 1 |
| **DHC7C** | 73 | 73 | 70 | 73 | 0 | 0 | 0.959 | 1.000 | 0.959 | 205577 | 11880 | 193697 | 11883 | 193694 | 11876 | 7 | 193690 | 4 | 1.000 | 0.999 | 1.000 | 1 |
| **DHC8** | 75 | 73 | 65 | 73 | 0 | 2 | 0.890 | 1.000 | 0.867 | 84895 | 12190 | 72705 | 12102 | 72793 | 12033 | 69 | 72636 | 157 | 0.987 | 0.994 | 0.989 | 1 |
| **DHC9A** | 74 | 75 | 71 | 74 | 1 | 0 | 0.947 | 0.987 | 0.959 | 306568 | 12186 | 294382 | 12057 | 294511 | 12036 | 21 | 294361 | 150 | 0.988 | 0.998 | 0.993 | 1 |
| **DHC9B** | 83 | 85 | 77 | 82 | 3 | 1 | 0.906 | 0.965 | 0.928 | 373531 | 13791 | 359740 | 13557 | 359974 | 13423 | 134 | 359606 | 368 | 0.973 | 0.990 | 0.981 | 1 |
| **DHC11** | 43 | 43 | 40 | 43 | 0 | 0 | 0.930 | 1.000 | 0.930 | 85205 | 14337 | 70868 | 14349 | 70856 | 14337 | 12 | 70856 | 0 | 1.000 | 0.999 | 0.999 | 1 |
| **Others** | **90** | **93** | **79** | **88** | **5** | **1** | **0.849** | **0.946** | **0.878** | **108735** | **14186** | **94549** | **14100** | **94635** | **14067** | **33** | **94516** | **119** | **0.992** | **0.998** | **0.994** | **7** |
| **NedKinesinJ_fl** | 4 | 3 | 2 | 3 | 0 | 0 | 0.667 | 1.000 | 0.500 | 6820 | 2273 | 4547 | 2226 | 4594 | 2223 | 3 | 4544 | 50 | 0.978 | 0.999 | 0.983 | 1 |

| | | | | | | | | | | | | | | | | | | | | | | |
|---|---|---|---|---|---|---|---|---|---|---|---|---|---|---|---|---|---|---|---|---|---|---|
| **PhsP62_fl** | 3 | 3 | 2 | 3 | 0 | 0 | 0.667 | 1.000 | 0.667 | 5645 | 1506 | 4139 | 1509 | 4136 | 1506 | 3 | 4136 | 0 | 1.000 | 0.998 | 0.999 | 1 |
| **HsP50_fl** | 14 | 16 | 13 | 14 | 2 | 0 | 0.813 | 0.875 | 0.929 | 20372 | 1218 | 19154 | 1206 | 19166 | 1203 | 3 | 19151 | 15 | 0.988 | 0.998 | 0.992 | 1 |
| **PugCor_fl** | 9 | 10 | 7 | 8 | 2 | 1 | 0.700 | 0.800 | 0.778 | 6606 | 1617 | 4989 | 1611 | 4995 | 1603 | 8 | 4981 | 14 | 0.991 | 0.995 | 0.991 | 1 |
| **MmP150_fl** | 31 | 32 | 30 | 31 | 1 | 0 | 0.938 | 0.969 | 0.968 | 23936 | 3843 | 20093 | 3831 | 20105 | 3828 | 3 | 20090 | 15 | 0.996 | 0.999 | 0.997 | 1 |
| **HsMyo19_fl** | 24 | 24 | 23 | 24 | 0 | 0 | 0.958 | 1.000 | 0.958 | 40271 | 2910 | 37361 | 2913 | 37358 | 2910 | 3 | 37358 | 0 | 1.000 | 0.999 | 0.999 | 1 |
| **ThCAP1_fl** | 5 | 5 | 2 | 5 | 0 | 0 | 0.400 | 1.000 | 0.400 | 5085 | 819 | 4266 | 804 | 4281 | 794 | 10 | 4256 | 25 | 0.969 | 0.988 | 0.974 | 1 |

**Wise2**

| | Exon PE | Exon AE | Exon TE | Exon OE | Exon ME | Exon WE | Exon Sn | Exon Sno | Exon Sp | Nucl Total | Nucl AP | Nucl AN | Nucl PP | Nucl PN | Nucl TP | Nucl FP | Nucl TN | Nucl FN | Nucl Sn | Nucl Sp | Nucl AC | Genes |
|---|---|---|---|---|---|---|---|---|---|---|---|---|---|---|---|---|---|---|---|---|---|---|
| **DHC all** | **1229** | **1202** | **1121** | **1199** | **3** | **16** | **0.933** | **0.998** | **0.912** | **3910464** | **209483** | **3700981** | **211260** | **3699204** | **208864** | **2396** | **3698585** | **619** | **0.997** | **0.989** | **0.992** | **39** |
| **DHC1** | 78 | 77 | 74 | 77 | 0 | 0 | 0.961 | 1.000 | 0.949 | 66248 | 13683 | 52565 | 13728 | 52520 | 13682 | 46 | 52519 | 1 | 1.000 | 0.997 | 0.998 | 2 |
| **DHC2** | 91 | 88 | 86 | 88 | 0 | 3 | 0.977 | 1.000 | 0.945 | 417085 | 12702 | 404383 | 12891 | 404194 | 12702 | 189 | 404194 | 0 | 1.000 | 0.985 | 0.992 | 2 |
| **DHC3A** | 92 | 92 | 89 | 92 | 0 | 0 | 0.967 | 1.000 | 0.967 | 362204 | 14070 | 348134 | 14100 | 348104 | 14070 | 30 | 348104 | 0 | 1.000 | 0.998 | 0.999 | 1 |
| **DHC3B** | 79 | 78 | 75 | 78 | 0 | 0 | 0.962 | 1.000 | 0.949 | 302827 | 13745 | 289082 | 13845 | 288982 | 13743 | 102 | 288980 | 2 | 1.000 | 0.993 | 0.996 | 2 |
| **DHC4A** | 84 | 82 | 74 | 82 | 0 | 1 | 0.902 | 1.000 | 0.881 | 365115 | 13523 | 351592 | 13551 | 351564 | 13509 | 42 | 351550 | 14 | 0.999 | 0.997 | 0.998 | 2 |
| **DHC4B** | 85 | 79 | 72 | 79 | 0 | 2 | 0.911 | 1.000 | 0.847 | 119251 | 13284 | 105967 | 13425 | 105826 | 13221 | 204 | 105763 | 63 | 0.995 | 0.985 | 0.989 | 7 |
| **DHC4C** | 74 | 68 | 61 | 68 | 0 | 3 | 0.897 | 1.000 | 0.824 | 490267 | 13014 | 477253 | 13254 | 477013 | 13008 | 246 | 477007 | 6 | 1.000 | 0.981 | 0.990 | 5 |
| **DHC5** | 79 | 79 | 73 | 79 | 0 | 0 | 0.924 | 1.000 | 0.924 | 144290 | 13752 | 130538 | 13797 | 130493 | 13739 | 58 | 130480 | 13 | 0.999 | 0.996 | 0.997 | 1 |
| **DHC6** | 85 | 86 | 80 | 84 | 2 | 1 | 0.930 | 0.977 | 0.941 | 138640 | 13371 | 125269 | 13293 | 125347 | 13067 | 226 | 125043 | 304 | 0.977 | 0.983 | 0.978 | 2 |
| **DHC7A** | 65 | 64 | 61 | 64 | 0 | 0 | 0.953 | 1.000 | 0.938 | 257605 | 12058 | 245547 | 12057 | 245548 | 12048 | 9 | 245538 | 10 | 0.999 | 0.999 | 0.999 | 2 |
| **DHC7B** | 62 | 60 | 57 | 60 | 0 | 2 | 0.950 | 1.000 | 0.919 | 191156 | 11897 | 179259 | 12132 | 179024 | 11883 | 249 | 179010 | 14 | 0.999 | 0.979 | 0.988 | 3 |
| **DHC7C** | 73 | 73 | 73 | 73 | 0 | 0 | 1.000 | 1.000 | 1.000 | 205577 | 11880 | 193697 | 11880 | 193697 | 11880 | 0 | 193697 | 0 | 1.000 | 1.000 | 1.000 | 1 |
| **DHC8** | 79 | 73 | 60 | 73 | 0 | 3 | 0.822 | 1.000 | 0.759 | 84895 | 12190 | 72705 | 12774 | 72121 | 12168 | 606 | 72099 | 22 | 0.998 | 0.953 | 0.971 | 6 |
| **DHC9A** | 75 | 75 | 69 | 75 | 0 | 0 | 0.920 | 1.000 | 0.920 | 306568 | 12186 | 294382 | 12405 | 294163 | 12176 | 229 | 294153 | 10 | 0.999 | 0.982 | 0.990 | 1 |
| **DHC9B** | 85 | 85 | 76 | 84 | 1 | 1 | 0.894 | 0.988 | 0.894 | 373531 | 13791 | 359740 | 13782 | 359749 | 13631 | 151 | 359589 | 160 | 0.988 | 0.989 | 0.988 | 1 |
| **DHC11** | 43 | 43 | 41 | 43 | 0 | 0 | 0.953 | 1.000 | 0.953 | 85205 | 14337 | 70868 | 14346 | 70859 | 14337 | 9 | 70859 | 0 | 1.000 | 0.999 | 1.000 | 1 |
| **Others** | **87** | **93** | **75** | **86** | **7** | **0** | **0.806** | **0.925** | **0.862** | **108735** | **14186** | **94549** | **14142** | **94593** | **14078** | **64** | **94485** | **108** | **0.992** | **0.995** | **0.993** | **8** |
| **NedKinesinJ_fl** | 4 | 3 | 2 | 3 | 0 | 0 | 0.667 | 1.000 | 0.500 | 6820 | 2273 | 4547 | 2271 | 4549 | 2271 | 0 | 4547 | 2 | 0.999 | 1.000 | 0.999 | 2 |
| **PhsP62_fl** | 3 | 3 | 1 | 3 | 0 | 0 | 0.333 | 1.000 | 0.333 | 5645 | 1506 | 4139 | 1488 | 4157 | 1488 | 0 | 4139 | 18 | 0.988 | 1.000 | 0.992 | 1 |
| **HsP50_fl** | 14 | 16 | 14 | 14 | 2 | 0 | 0.875 | 0.875 | 1.000 | 20372 | 1218 | 19154 | 1203 | 19169 | 1203 | 0 | 19154 | 15 | 0.988 | 1.000 | 0.993 | 1 |
| **PugCor_fl** | 8 | 10 | 5 | 8 | 2 | 0 | 0.500 | 0.800 | 0.625 | 6606 | 1617 | 4989 | 1605 | 5001 | 1596 | 9 | 4980 | 21 | 0.987 | 0.994 | 0.988 | 1 |
| **MmP150_fl** | 31 | 32 | 29 | 31 | 1 | 0 | 0.906 | 0.969 | 0.935 | 23936 | 3843 | 20093 | 3843 | 20093 | 3822 | 21 | 20072 | 21 | 0.995 | 0.995 | 0.993 | 1 |
| **HsMyo19_fl** | 23 | 24 | 21 | 23 | 1 | 0 | 0.875 | 0.958 | 0.913 | 40271 | 2910 | 37361 | 2913 | 37358 | 2898 | 15 | 37346 | 12 | 0.996 | 0.995 | 0.995 | 1 |
| **ThCAP1_fl** | 4 | 5 | 3 | 4 | 1 | 0 | 0.600 | 0.800 | 0.750 | 5085 | 819 | 4266 | 819 | 4266 | 800 | 19 | 4247 | 19 | 0.977 | 0.977 | 0.972 | 1 |

**Wise2 (global)**

| | Exon PE | Exon AE | Exon TE | Exon OE | Exon ME | Exon WE | Exon Sn | Exon Sno | Exon Sp | Nucl Total | Nucl AP | Nucl AN | Nucl PP | Nucl PN | Nucl TP | Nucl FP | Nucl TN | Nucl FN | Nucl Sn | Nucl Sp | Nucl AC | Genes |
|---|---|---|---|---|---|---|---|---|---|---|---|---|---|---|---|---|---|---|---|---|---|---|
| **DHC all** | **1233** | **1202** | **1119** | **1200** | **2** | **19** | **0.931** | **0.998** | **0.908** | **3910464** | **209483** | **3700981** | **211938** | **3698526** | **209095** | **2843** | **3698138** | **388** | **0.998** | **0.987** | **0.992** | **39** |
| **DHC1** | 80 | 77 | 73 | 77 | 0 | 2 | 0.948 | 1.000 | 0.913 | 66248 | 13683 | 52565 | 13968 | 52280 | 13681 | 287 | 52278 | 2 | 1.000 | 0.979 | 0.987 | 2 |
| **DHC2** | 91 | 88 | 86 | 88 | 0 | 3 | 0.977 | 1.000 | 0.945 | 417085 | 12702 | 404383 | 12891 | 404194 | 12702 | 189 | 404194 | 0 | 1.000 | 0.985 | 0.992 | 2 |
| **DHC3A** | 92 | 92 | 89 | 92 | 0 | 0 | 0.967 | 1.000 | 0.967 | 362204 | 14070 | 348134 | 14103 | 348101 | 14070 | 33 | 348101 | 0 | 1.000 | 0.998 | 0.999 | 1 |
| **DHC3B** | 79 | 78 | 75 | 78 | 0 | 0 | 0.962 | 1.000 | 0.949 | 302827 | 13745 | 289082 | 13845 | 288982 | 13743 | 102 | 288980 | 2 | 1.000 | 0.993 | 0.996 | 2 |
| **DHC4A** | 84 | 82 | 74 | 82 | 0 | 1 | 0.902 | 1.000 | 0.881 | 365115 | 13523 | 351592 | 13551 | 351564 | 13509 | 42 | 351550 | 14 | 0.999 | 0.997 | 0.998 | 2 |
| **DHC4B** | 85 | 79 | 72 | 79 | 0 | 2 | 0.911 | 1.000 | 0.847 | 119251 | 13284 | 105967 | 13425 | 105826 | 13221 | 204 | 105763 | 63 | 0.995 | 0.985 | 0.989 | 7 |
| **DHC4C** | 74 | 68 | 61 | 68 | 0 | 3 | 0.897 | 1.000 | 0.824 | 490267 | 13014 | 477253 | 13398 | 476869 | 13008 | 390 | 476863 | 6 | 1.000 | 0.971 | 0.985 | 5 |
| **DHC5** | 79 | 79 | 73 | 79 | 0 | 0 | 0.924 | 1.000 | 0.924 | 144290 | 13752 | 130538 | 13797 | 130493 | 13739 | 58 | 130480 | 13 | 0.999 | 0.996 | 0.997 | 1 |
| **DHC6** | 87 | 86 | 80 | 85 | 1 | 2 | 0.930 | 0.988 | 0.920 | 138640 | 13371 | 125269 | 13533 | 125107 | 13302 | 231 | 125038 | 69 | 0.995 | 0.983 | 0.988 | 2 |
| **DHC7A** | 65 | 64 | 61 | 64 | 0 | 0 | 0.953 | 1.000 | 0.938 | 257605 | 12058 | 245547 | 12063 | 245542 | 12048 | 15 | 245532 | 10 | 0.999 | 0.999 | 0.999 | 2 |
| **DHC7B** | 62 | 60 | 57 | 60 | 0 | 2 | 0.950 | 1.000 | 0.919 | 191156 | 11897 | 179259 | 12159 | 178997 | 11883 | 276 | 178983 | 14 | 0.999 | 0.977 | 0.987 | 3 |
| **DHC7C** | 73 | 73 | 73 | 73 | 0 | 0 | 1.000 | 1.000 | 1.000 | 205577 | 11880 | 193697 | 11880 | 193697 | 11880 | 0 | 193697 | 0 | 1.000 | 1.000 | 1.000 | 1 |
| **DHC8** | 79 | 73 | 60 | 73 | 0 | 3 | 0.822 | 1.000 | 0.759 | 84895 | 12190 | 72705 | 12774 | 72121 | 12168 | 606 | 72099 | 22 | 0.998 | 0.953 | 0.971 | 6 |
| **DHC9A** | 75 | 75 | 69 | 75 | 0 | 0 | 0.920 | 1.000 | 0.920 | 306568 | 12186 | 294382 | 12405 | 294163 | 12176 | 229 | 294153 | 10 | 0.999 | 0.982 | 0.990 | 1 |
| **DHC9B** | 85 | 85 | 75 | 84 | 1 | 1 | 0.882 | 0.988 | 0.882 | 373531 | 13791 | 359740 | 13800 | 359731 | 13628 | 172 | 359568 | 163 | 0.988 | 0.988 | 0.987 | 1 |
| **DHC11** | 43 | 43 | 41 | 43 | 0 | 0 | 0.953 | 1.000 | 0.953 | 85205 | 14337 | 70868 | 14346 | 70859 | 14337 | 9 | 70859 | 0 | 1.000 | 0.999 | 1.000 | 1 |
| **Others** | **87** | **93** | **76** | **86** | **7** | **0** | **0.817** | **0.925** | **0.874** | **108735** | **14186** | **94549** | **14163** | **94572** | **14093** | **70** | **94479** | **93** | **0.993** | **0.995** | **0.993** | **8** |
| **NedKinesinJ_fl** | 4 | 3 | 2 | 3 | 0 | 0 | 0.667 | 1.000 | 0.500 | 6820 | 2273 | 4547 | 2271 | 4549 | 2271 | 0 | 4547 | 2 | 0.999 | 1.000 | 0.999 | 2 |
| **PhsP62_fl** | 3 | 3 | 2 | 3 | 0 | 0 | 0.667 | 1.000 | 0.667 | 5645 | 1506 | 4139 | 1503 | 4142 | 1503 | 0 | 4139 | 3 | 0.998 | 1.000 | 0.999 | 1 |
| **HsP50_fl** | 14 | 16 | 14 | 14 | 2 | 0 | 0.875 | 0.875 | 1.000 | 20372 | 1218 | 19154 | 1203 | 19169 | 1203 | 0 | 19154 | 15 | 0.988 | 1.000 | 0.993 | 1 |
| **PugCor_fl** | 8 | 10 | 5 | 8 | 2 | 0 | 0.500 | 0.800 | 0.625 | 6606 | 1617 | 4989 | 1608 | 4998 | 1596 | 12 | 4977 | 21 | 0.987 | 0.993 | 0.986 | 1 |
| **MmP150_fl** | 31 | 32 | 29 | 31 | 1 | 0 | 0.906 | 0.969 | 0.935 | 23936 | 3843 | 20093 | 3843 | 20093 | 3822 | 21 | 20072 | 21 | 0.995 | 0.995 | 0.993 | 1 |

| | | | | | | | | | | | | | | | | | | | | | | |
|---|---|---|---|---|---|---|---|---|---|---|---|---|---|---|---|---|---|---|---|---|---|---|
| **HsMyo19_fl** | 23 | 24 | 21 | 23 | 1 | 0 | 0.875 | 0.958 | 0.913 | 40271 | 2910 | 37361 | 2916 | 37355 | 2898 | 18 | 37343 | 12 | 0.996 | 0.994 | 0.994 | 1 |
| **ThCAP1_fl** | 4 | 5 | 3 | 4 | 1 | 0 | 0.600 | 0.800 | 0.750 | 5085 | 819 | 4266 | 819 | 4266 | 800 | 19 | 4247 | 19 | 0.977 | 0.977 | 0.972 | 1 |

**Augustus**

| | Exon PE | Exon AE | Exon TE | Exon OE | Exon ME | Exon WE | Exon Sn | Exon Sno | Exon Sp | Nucl Total | Nucl AP | Nucl AN | Nucl PP | Nucl PN | Nucl TP | Nucl FP | Nucl TN | Nucl FN | Nucl Sn | Nucl Sp | Nucl AC | Genes |
|---|---|---|---|---|---|---|---|---|---|---|---|---|---|---|---|---|---|---|---|---|---|---|
| **DHC all** | **1247** | **1202** | **960** | **1063** | **139** | **183** | **0.799** | **0.884** | **0.770** | **3910464** | **209482** | **3700982** | **234433** | **3676031** | **187038** | **47395** | **3653587** | **22444** | **0.893** | **0.798** | **0.836** | **84** |
| **DHC1** | 76 | 77 | 72 | 75 | 2 | 1 | 0.935 | 0.974 | 0.947 | 66248 | 13682 | 52566 | 13530 | 52718 | 13370 | 160 | 52406 | 312 | 0.977 | 0.988 | 0.978 | 1 |
| **DHC2** | 96 | 88 | 75 | 79 | 9 | 17 | 0.852 | 0.898 | 0.781 | 417085 | 12702 | 404383 | 16272 | 400813 | 11601 | 4671 | 399712 | 1101 | 0.913 | 0.713 | 0.806 | 9 |
| **DHC3A** | 87 | 92 | 76 | 79 | 13 | 8 | 0.826 | 0.859 | 0.874 | 362204 | 14070 | 348134 | 13494 | 348710 | 12340 | 1154 | 346980 | 1730 | 0.877 | 0.914 | 0.892 | 2 |
| **DHC3B** | 89 | 78 | 71 | 75 | 3 | 14 | 0.910 | 0.962 | 0.798 | 302827 | 13745 | 289082 | 16140 | 286687 | 13232 | 2908 | 286174 | 513 | 0.963 | 0.820 | 0.885 | 4 |
| **DHC4A** | 77 | 82 | 67 | 70 | 12 | 7 | 0.817 | 0.854 | 0.870 | 365115 | 13523 | 351592 | 12666 | 352449 | 11487 | 1179 | 350413 | 2036 | 0.849 | 0.907 | 0.874 | 2 |
| **DHC4B** | 75 | 79 | 69 | 74 | 5 | 1 | 0.873 | 0.937 | 0.920 | 119251 | 13284 | 105967 | 12981 | 106270 | 12659 | 322 | 105645 | 625 | 0.953 | 0.975 | 0.960 | 1 |
| **DHC4C** | 89 | 68 | 55 | 61 | 7 | 28 | 0.809 | 0.897 | 0.618 | 490267 | 13014 | 477253 | 21749 | 468518 | 11900 | 9849 | 467404 | 1114 | 0.914 | 0.547 | 0.719 | 11 |
| **DHC5** | 86 | 79 | 63 | 74 | 5 | 12 | 0.797 | 0.937 | 0.733 | 144290 | 13752 | 130538 | 16188 | 128102 | 12929 | 3259 | 127279 | 823 | 0.940 | 0.799 | 0.854 | 7 |
| **DHC6** | 97 | 86 | 69 | 80 | 6 | 17 | 0.802 | 0.930 | 0.711 | 138640 | 13371 | 125269 | 17343 | 121297 | 12286 | 5057 | 120212 | 1085 | 0.919 | 0.708 | 0.789 | 11 |
| **DHC7A** | 66 | 64 | 47 | 52 | 12 | 13 | 0.734 | 0.813 | 0.712 | 257605 | 12058 | 245547 | 14550 | 243055 | 10322 | 4228 | 241319 | 1736 | 0.856 | 0.709 | 0.771 | 6 |
| **DHC7B** | 66 | 60 | 52 | 58 | 2 | 8 | 0.867 | 0.967 | 0.788 | 191156 | 11897 | 179259 | 13959 | 177197 | 11529 | 2430 | 176829 | 368 | 0.969 | 0.826 | 0.890 | 5 |
| **DHC7C** | 68 | 73 | 51 | 55 | 18 | 13 | 0.699 | 0.753 | 0.750 | 205577 | 11880 | 193697 | 12237 | 193340 | 9351 | 2886 | 190811 | 2529 | 0.787 | 0.764 | 0.762 | 3 |
| **DHC8** | 72 | 73 | 56 | 70 | 3 | 2 | 0.767 | 0.959 | 0.778 | 84895 | 12190 | 72705 | 11604 | 73291 | 11287 | 317 | 72388 | 903 | 0.926 | 0.973 | 0.941 | 3 |
| **DHC9A** | 80 | 75 | 56 | 64 | 11 | 16 | 0.747 | 0.853 | 0.700 | 306568 | 12186 | 294382 | 14156 | 292412 | 9809 | 4347 | 290035 | 2377 | 0.805 | 0.693 | 0.737 | 9 |
| **DHC9B** | 79 | 85 | 51 | 58 | 27 | 21 | 0.600 | 0.682 | 0.646 | 373531 | 13791 | 359740 | 13098 | 360433 | 9521 | 3577 | 356163 | 4270 | 0.690 | 0.727 | 0.698 | 7 |
| **DHC11** | 44 | 43 | 30 | 39 | 4 | 5 | 0.698 | 0.907 | 0.682 | 85205 | 14337 | 70868 | 14466 | 70739 | 13415 | 1051 | 69817 | 922 | 0.936 | 0.927 | 0.918 | 3 |
| **Others** | **91** | **93** | **55** | **71** | **22** | **21** | **0.591** | **0.763** | **0.604** | **108735** | **14186** | **94549** | **17858** | **90877** | **12538** | **5320** | **89229** | **1648** | **0.884** | **0.702** | **0.756** | **13** |
| **NedKinesinJ_fl** | 5 | 3 | 1 | 3 | 0 | 1 | 0.333 | 1.000 | 0.200 | 6820 | 2273 | 4547 | 2853 | 3967 | 2193 | 660 | 3887 | 80 | 0.965 | 0.769 | 0.784 | 2 |
| **PhsP62_fl** | 15 | 3 | 0 | 3 | 0 | 13 | 0.000 | 1.000 | 0.000 | 5645 | 1506 | 4139 | 3845 | 1800 | 1506 | 2339 | 1800 | 0 | 1.000 | 0.392 | 0.413 | 4 |
| **HsP50_fl** | 12 | 16 | 11 | 12 | 4 | 0 | 0.688 | 0.750 | 0.917 | 20372 | 1218 | 19154 | 1098 | 19274 | 1095 | 3 | 19151 | 123 | 0.899 | 0.997 | 0.945 | 1 |
| **PugCor_fl** | 4 | 10 | 0 | 4 | 6 | 1 | 0.000 | 0.400 | 0.000 | 6606 | 1617 | 4989 | 1344 | 5262 | 1172 | 172 | 4817 | 445 | 0.725 | 0.872 | 0.739 | 1 |
| **MmP150_fl** | 25 | 32 | 23 | 25 | 7 | 0 | 0.719 | 0.781 | 0.920 | 23936 | 3843 | 20093 | 3366 | 20570 | 3363 | 3 | 20090 | 480 | 0.875 | 0.999 | 0.925 | 1 |
| **HsMyo19_fl** | 23 | 24 | 20 | 22 | 2 | 1 | 0.833 | 0.917 | 0.870 | 40271 | 2910 | 37361 | 2730 | 37541 | 2604 | 126 | 37235 | 306 | 0.895 | 0.954 | 0.919 | 1 |
| **ThCAP1_fl** | 7 | 5 | 0 | 2 | 3 | 5 | 0.000 | 0.400 | 0.000 | 5085 | 819 | 4266 | 2622 | 2463 | 605 | 2017 | 2249 | 214 | 0.739 | 0.231 | 0.205 | 3 |

**Augustus (1 gene)**

| | Exon PE | Exon AE | Exon TE | Exon OE | Exon ME | Exon WE | Exon Sn | Exon Sno | Exon Sp | Nucl Total | Nucl AP | Nucl AN | Nucl PP | Nucl PN | Nucl TP | Nucl FP | Nucl TN | Nucl FN | Nucl Sn | Nucl Sp | Nucl AC | Genes |
|---|---|---|---|---|---|---|---|---|---|---|---|---|---|---|---|---|---|---|---|---|---|---|
| **DHC all** | **1182** | **1202** | **984** | **1070** | **132** | **111** | **0.819** | **0.890** | **0.832** | **3910464** | **209482** | **3700982** | **212364** | **3698100** | **188384** | **23980** | **3677002** | **21098** | **0.899** | **0.887** | **0.887** | **16** |
| **DHC1** | 76 | 77 | 72 | 75 | 2 | 1 | 0.935 | 0.974 | 0.947 | 66248 | 13682 | 52566 | 13530 | 52718 | 13370 | 160 | 52406 | 312 | 0.977 | 0.988 | 0.978 | 1 |
| **DHC2** | 85 | 88 | 77 | 80 | 8 | 5 | 0.875 | 0.909 | 0.906 | 417085 | 12702 | 404383 | 13635 | 403450 | 11711 | 1924 | 402459 | 991 | 0.922 | 0.859 | 0.887 | 1 |
| **DHC3A** | 87 | 92 | 76 | 79 | 13 | 8 | 0.826 | 0.859 | 0.874 | 362204 | 14070 | 348134 | 13569 | 348635 | 12340 | 1229 | 346905 | 1730 | 0.877 | 0.909 | 0.889 | 1 |
| **DHC3B** | 88 | 78 | 71 | 75 | 3 | 13 | 0.910 | 0.962 | 0.807 | 302827 | 13745 | 289082 | 15408 | 287419 | 13324 | 2084 | 286998 | 421 | 0.969 | 0.865 | 0.913 | 1 |
| **DHC4A** | 76 | 82 | 67 | 70 | 12 | 6 | 0.817 | 0.854 | 0.882 | 365115 | 13523 | 351592 | 12303 | 352812 | 11487 | 816 | 350776 | 2036 | 0.849 | 0.934 | 0.888 | 1 |
| **DHC4B** | 75 | 79 | 69 | 74 | 5 | 1 | 0.873 | 0.937 | 0.920 | 119251 | 13284 | 105967 | 12981 | 106270 | 12659 | 322 | 105645 | 625 | 0.953 | 0.975 | 0.960 | 1 |
| **DHC4C** | 77 | 68 | 57 | 62 | 6 | 15 | 0.838 | 0.912 | 0.740 | 490267 | 13014 | 477253 | 17637 | 472630 | 12049 | 5588 | 471665 | 965 | 0.926 | 0.683 | 0.798 | 1 |
| **DHC5** | 82 | 79 | 66 | 74 | 5 | 8 | 0.835 | 0.937 | 0.805 | 144290 | 13752 | 130538 | 14832 | 129458 | 12972 | 1860 | 128678 | 780 | 0.943 | 0.875 | 0.899 | 1 |
| **DHC6** | 87 | 86 | 73 | 80 | 6 | 7 | 0.849 | 0.930 | 0.839 | 138640 | 13371 | 125269 | 13647 | 124993 | 12301 | 1346 | 123923 | 1070 | 0.920 | 0.901 | 0.901 | 1 |
| **DHC7A** | 60 | 64 | 46 | 49 | 15 | 10 | 0.719 | 0.766 | 0.767 | 257605 | 12058 | 245547 | 13401 | 244204 | 9768 | 3633 | 241914 | 2290 | 0.810 | 0.729 | 0.757 | 1 |
| **DHC7B** | 61 | 60 | 55 | 58 | 2 | 3 | 0.917 | 0.967 | 0.902 | 191156 | 11897 | 179259 | 11892 | 179264 | 11609 | 283 | 178976 | 288 | 0.976 | 0.976 | 0.974 | 1 |
| **DHC7C** | 67 | 73 | 53 | 57 | 16 | 10 | 0.726 | 0.781 | 0.791 | 205577 | 11880 | 193697 | 11511 | 194066 | 9655 | 1856 | 191841 | 2225 | 0.813 | 0.839 | 0.815 | 1 |
| **DHC8** | 72 | 73 | 56 | 69 | 4 | 3 | 0.767 | 0.945 | 0.778 | 84895 | 12190 | 72705 | 11628 | 73267 | 11149 | 479 | 72226 | 1041 | 0.915 | 0.959 | 0.926 | 1 |
| **DHC9A** | 72 | 75 | 60 | 68 | 7 | 4 | 0.800 | 0.907 | 0.833 | 306568 | 12186 | 294382 | 10953 | 295615 | 10350 | 603 | 293779 | 1836 | 0.849 | 0.945 | 0.893 | 1 |
| **DHC9B** | 76 | 85 | 56 | 61 | 24 | 15 | 0.659 | 0.718 | 0.737 | 373531 | 13791 | 359740 | 11697 | 361834 | 10225 | 1472 | 358268 | 3566 | 0.741 | 0.874 | 0.801 | 1 |
| **DHC11** | 41 | 43 | 30 | 39 | 4 | 2 | 0.698 | 0.907 | 0.732 | 85205 | 14337 | 70868 | 13740 | 71465 | 13415 | 325 | 70543 | 922 | 0.936 | 0.976 | 0.947 | 1 |
| **Others** | **85** | **93** | **57** | **72** | **21** | **14** | **0.613** | **0.774** | **0.671** | **108735** | **14186** | **94549** | **17034** | **91701** | **12648** | **4386** | **90163** | **1538** | **0.892** | **0.743** | **0.785** | **7** |
| **NedKinesinJ_fl** | 4 | 3 | 1 | 3 | 0 | 0 | 0.333 | 1.000 | 0.250 | 6820 | 2273 | 4547 | 2568 | 4252 | 2193 | 375 | 4172 | 80 | 0.965 | 0.854 | 0.859 | 1 |
| **PhsP62_fl** | 9 | 3 | 0 | 3 | 0 | 7 | 0.000 | 1.000 | 0.000 | 5645 | 1506 | 4139 | 3207 | 2438 | 1479 | 1728 | 2411 | 27 | 0.982 | 0.461 | 0.507 | 1 |
| **HsP50_fl** | 12 | 16 | 11 | 12 | 4 | 0 | 0.688 | 0.750 | 0.917 | 20372 | 1218 | 19154 | 1098 | 19274 | 1095 | 3 | 19151 | 123 | 0.899 | 0.997 | 0.945 | 1 |
| **PugCor_fl** | 4 | 10 | 0 | 4 | 6 | 1 | 0.000 | 0.400 | 0.000 | 6606 | 1617 | 4989 | 1344 | 5262 | 1172 | 172 | 4817 | 445 | 0.725 | 0.872 | 0.739 | 1 |
| **MmP150_fl** | 25 | 32 | 23 | 25 | 7 | 0 | 0.719 | 0.781 | 0.920 | 23936 | 3843 | 20093 | 3366 | 20570 | 3363 | 3 | 20090 | 480 | 0.875 | 0.999 | 0.925 | 1 |
| **HsMyo19_fl** | 23 | 24 | 20 | 22 | 2 | 1 | 0.833 | 0.917 | 0.870 | 40271 | 2910 | 37361 | 2730 | 37541 | 2604 | 126 | 37235 | 306 | 0.895 | 0.954 | 0.919 | 1 |
| **ThCAP1_fl** | 8 | 5 | 2 | 3 | 2 | 5 | 0.400 | 0.600 | 0.250 | 5085 | 819 | 4266 | 2721 | 2364 | 742 | 1979 | 2287 | 77 | 0.906 | 0.273 | 0.341 | 1 |

**Fgenesh**

| | Exon PE | Exon AE | Exon TE | Exon OE | Exon ME | Exon WE | Exon Sn | Exon Sno | Exon Sp | Nucl Total | Nucl AP | Nucl AN | Nucl PP | Nucl PN | Nucl TP | Nucl FP | Nucl TN | Nucl FN | Nucl Sn | Nucl Sp | Nucl AC | Genes |
|---|---|---|---|---|---|---|---|---|---|---|---|---|---|---|---|---|---|---|---|---|---|---|
| **DHC all** | **1433** | **1202** | **964** | **1091** | **111** | **342** | **0.802** | **0.908** | **0.673** | **3910464** | **209482** | **3700982** | **308610** | **3601854** | **192258** | **116352** | **3584630** | **17224** | **0.918** | **0.623** | **0.752** | **161** |
| DHC1 | 82 | 77 | 72 | 77 | 0 | 5 | 0.935 | 1.000 | 0.878 | 66248 | 13682 | 52566 | 14253 | 51995 | 13682 | 571 | 51995 | 0 | 1.000 | 0.960 | 0.975 | 1 |
| DHC2 | 116 | 88 | 67 | 70 | 18 | 46 | 0.761 | 0.795 | 0.578 | 417085 | 12702 | 404383 | 29583 | 387502 | 10701 | 18882 | 385501 | 2001 | 0.842 | 0.362 | 0.576 | 25 |
| DHC3A | 116 | 92 | 74 | 77 | 15 | 39 | 0.804 | 0.837 | 0.638 | 362204 | 14070 | 348134 | 22224 | 339980 | 11940 | 10284 | 337850 | 2130 | 0.849 | 0.537 | 0.675 | 12 |
| DHC3B | 106 | 78 | 62 | 72 | 6 | 34 | 0.795 | 0.923 | 0.585 | 302827 | 13745 | 289082 | 24501 | 278326 | 12881 | 11620 | 277462 | 864 | 0.937 | 0.526 | 0.710 | 20 |
| DHC4A | 105 | 82 | 70 | 76 | 6 | 29 | 0.854 | 0.927 | 0.667 | 365115 | 13523 | 351592 | 18363 | 346752 | 12604 | 5759 | 345833 | 919 | 0.932 | 0.686 | 0.800 | 11 |
| DHC4B | 80 | 79 | 67 | 79 | 0 | 2 | 0.848 | 1.000 | 0.838 | 119251 | 13284 | 105967 | 13302 | 105949 | 12984 | 318 | 105649 | 300 | 0.977 | 0.976 | 0.974 | 2 |
| DHC4C | 119 | 68 | 55 | 66 | 2 | 53 | 0.809 | 0.971 | 0.462 | 490267 | 13014 | 477253 | 31712 | 458555 | 12583 | 19129 | 458124 | 431 | 0.967 | 0.397 | 0.661 | 24 |
| DHC5 | 80 | 79 | 66 | 72 | 7 | 8 | 0.835 | 0.911 | 0.825 | 144290 | 13752 | 130538 | 15050 | 129240 | 12833 | 2217 | 128321 | 919 | 0.933 | 0.853 | 0.881 | 2 |
| DHC6 | 85 | 86 | 74 | 82 | 4 | 3 | 0.860 | 0.953 | 0.871 | 138640 | 13371 | 125269 | 14175 | 124465 | 12608 | 1567 | 123702 | 763 | 0.943 | 0.889 | 0.907 | 3 |
| DHC7A | 79 | 64 | 49 | 54 | 10 | 24 | 0.766 | 0.844 | 0.620 | 257605 | 12058 | 245547 | 18645 | 238960 | 10325 | 8320 | 237227 | 1733 | 0.856 | 0.554 | 0.684 | 8 |
| DHC7B | 75 | 60 | 50 | 59 | 1 | 16 | 0.833 | 0.983 | 0.667 | 191156 | 11897 | 179259 | 16782 | 174374 | 11404 | 5378 | 173881 | 493 | 0.959 | 0.680 | 0.803 | 6 |
| DHC7C | 83 | 73 | 61 | 67 | 6 | 16 | 0.836 | 0.918 | 0.735 | 205577 | 11880 | 193697 | 19539 | 186038 | 11271 | 8268 | 185429 | 609 | 0.949 | 0.577 | 0.740 | 12 |
| DHC8 | 77 | 73 | 55 | 72 | 1 | 5 | 0.753 | 0.986 | 0.714 | 84895 | 12190 | 72705 | 12273 | 72622 | 11505 | 768 | 71937 | 685 | 0.944 | 0.937 | 0.931 | 2 |
| DHC9A | 87 | 75 | 57 | 66 | 9 | 21 | 0.760 | 0.880 | 0.655 | 306568 | 12186 | 294382 | 21536 | 285032 | 10990 | 10546 | 283836 | 1196 | 0.902 | 0.510 | 0.686 | 14 |
| DHC9B | 99 | 85 | 53 | 61 | 24 | 38 | 0.624 | 0.718 | 0.535 | 373531 | 13791 | 359740 | 22602 | 350929 | 10095 | 12507 | 347233 | 3696 | 0.732 | 0.447 | 0.567 | 18 |
| DHC11 | 44 | 43 | 32 | 41 | 2 | 3 | 0.744 | 0.953 | 0.727 | 85205 | 14337 | 70868 | 14070 | 71135 | 13852 | 218 | 70650 | 485 | 0.966 | 0.985 | 0.970 | 1 |
| **Others** | **103** | **93** | **66** | **78** | **15** | **25** | **0.710** | **0.839** | **0.641** | **108735** | **14186** | **94549** | **18227** | **90508** | **13549** | **4678** | **89871** | **637** | **0.955** | **0.743** | **0.821** | **10** |
| NedKinesinJ_fl | 4 | 3 | 1 | 3 | 0 | 1 | 0.333 | 1.000 | 0.250 | 6820 | 2273 | 4547 | 3054 | 3766 | 2223 | 831 | 3716 | 50 | 0.978 | 0.728 | 0.755 | 2 |
| PhsP62_fl | 13 | 3 | 2 | 3 | 0 | 10 | 0.667 | 1.000 | 0.154 | 5645 | 1506 | 4139 | 2598 | 3047 | 1506 | 1092 | 3047 | 0 | 1.000 | 0.580 | 0.658 | 2 |
| HsP50_fl | 15 | 16 | 13 | 14 | 2 | 1 | 0.813 | 0.875 | 0.867 | 20372 | 1218 | 19154 | 1239 | 19133 | 1202 | 37 | 19117 | 16 | 0.987 | 0.970 | 0.977 | 1 |
| PugCor_fl | 11 | 10 | 5 | 7 | 3 | 4 | 0.500 | 0.700 | 0.455 | 6606 | 1617 | 4989 | 1881 | 4725 | 1513 | 368 | 4621 | 104 | 0.936 | 0.804 | 0.822 | 2 |
| MmP150_fl | 26 | 32 | 25 | 26 | 6 | 0 | 0.781 | 0.813 | 0.962 | 23936 | 3843 | 20093 | 3657 | 20279 | 3654 | 3 | 20090 | 189 | 0.951 | 0.999 | 0.970 | 1 |
| HsMyo19_fl | 24 | 24 | 18 | 22 | 2 | 2 | 0.750 | 0.917 | 0.750 | 40271 | 2910 | 37361 | 3024 | 37247 | 2725 | 299 | 37062 | 185 | 0.936 | 0.901 | 0.912 | 1 |
| ThCAP1_fl | 10 | 5 | 2 | 3 | 2 | 7 | 0.400 | 0.600 | 0.200 | 5085 | 819 | 4266 | 2774 | 2311 | 726 | 2048 | 2218 | 93 | 0.886 | 0.262 | 0.314 | 1 |

**Genscan**

| | Exon PE | Exon AE | Exon TE | Exon OE | Exon ME | Exon WE | Exon Sn | Exon Sno | Exon Sp | Nucl Total | Nucl AP | Nucl AN | Nucl PP | Nucl PN | Nucl TP | Nucl FP | Nucl TN | Nucl FN | Nucl Sn | Nucl Sp | Nucl AC | Genes |
|---|---|---|---|---|---|---|---|---|---|---|---|---|---|---|---|---|---|---|---|---|---|---|
| **DHC all** | **1583** | **1202** | **917** | **1064** | **138** | **520** | **0.763** | **0.885** | **0.579** | **3910464** | **209482** | **3700982** | **342235** | **3568229** | **189362** | **152873** | **3548109** | **20120** | **0.904** | **0.553** | **0.705** | **194** |
| DHC1 | 77 | 77 | 71 | 77 | 0 | 1 | 0.922 | 1.000 | 0.922 | 66248 | 13682 | 52566 | 14076 | 52172 | 13682 | 394 | 52172 | 0 | 1.000 | 0.972 | 0.982 | 1 |
| DHC2 | 131 | 88 | 62 | 64 | 24 | 67 | 0.705 | 0.727 | 0.473 | 417085 | 12702 | 404383 | 32403 | 384682 | 9987 | 22416 | 381967 | 2715 | 0.786 | 0.308 | 0.516 | 30 |
| DHC3A | 133 | 92 | 68 | 76 | 16 | 57 | 0.739 | 0.826 | 0.511 | 362204 | 14070 | 348134 | 24259 | 337945 | 11961 | 12298 | 335836 | 2109 | 0.850 | 0.493 | 0.651 | 14 |
| DHC3B | 114 | 78 | 61 | 75 | 3 | 39 | 0.782 | 0.962 | 0.535 | 302827 | 13745 | 289082 | 28092 | 274735 | 13107 | 14985 | 274097 | 638 | 0.954 | 0.467 | 0.683 | 23 |
| DHC4A | 121 | 82 | 67 | 74 | 8 | 47 | 0.817 | 0.902 | 0.554 | 365115 | 13523 | 351592 | 21102 | 344013 | 12208 | 8894 | 342698 | 1315 | 0.903 | 0.579 | 0.726 | 11 |
| DHC4B | 82 | 79 | 65 | 78 | 1 | 5 | 0.823 | 0.987 | 0.793 | 119251 | 13284 | 105967 | 13806 | 105445 | 12893 | 913 | 105054 | 391 | 0.971 | 0.934 | 0.946 | 2 |
| DHC4C | 152 | 68 | 43 | 60 | 8 | 92 | 0.632 | 0.882 | 0.283 | 490267 | 13014 | 477253 | 37662 | 452605 | 11760 | 25902 | 451351 | 1254 | 0.904 | 0.312 | 0.579 | 26 |
| DHC5 | 87 | 79 | 71 | 75 | 4 | 12 | 0.899 | 0.949 | 0.816 | 144290 | 13752 | 130538 | 16431 | 127859 | 13268 | 3163 | 127375 | 484 | 0.965 | 0.807 | 0.872 | 2 |
| DHC6 | 102 | 86 | 78 | 86 | 0 | 16 | 0.907 | 1.000 | 0.765 | 138640 | 13371 | 125269 | 20214 | 118426 | 13255 | 6959 | 118310 | 116 | 0.991 | 0.656 | 0.795 | 7 |
| DHC7A | 86 | 64 | 50 | 54 | 10 | 31 | 0.781 | 0.844 | 0.581 | 257605 | 12058 | 245547 | 19986 | 237619 | 10602 | 9384 | 236163 | 1456 | 0.879 | 0.530 | 0.683 | 8 |
| DHC7B | 75 | 60 | 51 | 60 | 0 | 15 | 0.850 | 1.000 | 0.680 | 191156 | 11897 | 179259 | 17766 | 173390 | 11879 | 5887 | 173372 | 18 | 0.998 | 0.669 | 0.817 | 6 |
| DHC7C | 89 | 73 | 58 | 64 | 9 | 25 | 0.795 | 0.877 | 0.652 | 205577 | 11880 | 193697 | 19521 | 186056 | 10729 | 8792 | 184905 | 1151 | 0.903 | 0.550 | 0.701 | 13 |
| DHC8 | 83 | 73 | 56 | 73 | 0 | 10 | 0.767 | 1.000 | 0.675 | 84895 | 12190 | 72705 | 13347 | 71548 | 11749 | 1598 | 71107 | 441 | 0.964 | 0.880 | 0.908 | 2 |
| DHC9A | 100 | 75 | 45 | 59 | 16 | 41 | 0.600 | 0.787 | 0.450 | 306568 | 12186 | 294382 | 24768 | 281800 | 9819 | 14949 | 279433 | 2367 | 0.806 | 0.396 | 0.572 | 22 |
| DHC9B | 110 | 85 | 37 | 49 | 36 | 61 | 0.435 | 0.576 | 0.336 | 373531 | 13791 | 359740 | 24504 | 349027 | 8339 | 16165 | 343575 | 5452 | 0.605 | 0.340 | 0.442 | 26 |
| DHC11 | 41 | 43 | 34 | 40 | 3 | 1 | 0.791 | 0.930 | 0.829 | 85205 | 14337 | 70868 | 14298 | 70907 | 14124 | 174 | 70694 | 213 | 0.985 | 0.988 | 0.984 | 1 |
| **Others** | **104** | **93** | **61** | **78** | **15** | **29** | **0.656** | **0.839** | **0.587** | **108735** | **14186** | **94549** | **20473** | **88262** | **13497** | **6976** | **87573** | **689** | **0.951** | **0.659** | **0.765** | **11** |
| NedKinesinJ_fl | 3 | 3 | 0 | 3 | 0 | 1 | 0.000 | 1.000 | 0.000 | 6820 | 2273 | 4547 | 3436 | 3384 | 2201 | 1235 | 3312 | 72 | 0.968 | 0.641 | 0.658 | 3 |
| PhsP62_fl | 16 | 3 | 1 | 3 | 0 | 14 | 0.333 | 1.000 | 0.063 | 5645 | 1506 | 4139 | 3828 | 1817 | 1479 | 2349 | 1790 | 27 | 0.982 | 0.386 | 0.393 | 2 |
| HsP50_fl | 15 | 16 | 11 | 13 | 3 | 2 | 0.688 | 0.813 | 0.733 | 20372 | 1218 | 19154 | 1548 | 18824 | 1163 | 385 | 18769 | 55 | 0.955 | 0.751 | 0.842 | 1 |
| PugCor_fl | 5 | 10 | 1 | 5 | 5 | 1 | 0.100 | 0.500 | 0.200 | 6606 | 1617 | 4989 | 1767 | 4839 | 1375 | 392 | 4597 | 242 | 0.850 | 0.778 | 0.750 | 1 |
| MmP150_fl | 32 | 32 | 26 | 29 | 3 | 3 | 0.813 | 0.906 | 0.813 | 23936 | 3843 | 20093 | 4020 | 19916 | 3725 | 295 | 19798 | 118 | 0.969 | 0.927 | 0.938 | 1 |
| HsMyo19_fl | 25 | 24 | 20 | 22 | 2 | 3 | 0.833 | 0.917 | 0.800 | 40271 | 2910 | 37361 | 3279 | 36992 | 2828 | 451 | 36910 | 82 | 0.972 | 0.862 | 0.910 | 2 |
| ThCAP1_fl | 8 | 5 | 2 | 3 | 2 | 5 | 0.400 | 0.600 | 0.250 | 5085 | 819 | 4266 | 2595 | 2490 | 726 | 1869 | 2397 | 93 | 0.886 | 0.280 | 0.345 | 1 |

**Blat**

| | Exon PE | Exon AE | Exon TE | Exon OE | Exon ME | Exon WE | Exon Sn | Exon Sno | Exon Sp | Nucl Total | Nucl AP | Nucl AN | Nucl PP | Nucl PN | Nucl TP | Nucl FP | Nucl TN | Nucl FN | Nucl Sn | Nucl Sp | Nucl AC | Genes |
|---|---|---|---|---|---|---|---|---|---|---|---|---|---|---|---|---|---|---|---|---|---|---|
| **DHC all** | **1231** | **1202** | **239** | **1186** | **16** | **19** | **0.199** | **0.987** | **0.194** | **3910464** | **209483** | **3700981** | **205554** | **3704910** | **203208** | **2346** | **3698635** | **6275** | **0.970** | **0.989** | **0.978** | **-** |
| DHC1 | 79 | 77 | 21 | 77 | 0 | 1 | 0.273 | 1.000 | 0.266 | 66248 | 13683 | 52565 | 13713 | 52535 | 13605 | 108 | 52457 | 78 | 0.994 | 0.992 | 0.991 | - |

| | | | | | | | | | | | | | | | | | | | | | | |
|---|---|---|---|---|---|---|---|---|---|---|---|---|---|---|---|---|---|---|---|---|---|---|
| **DHC2** | 90 | 88 | 18 | 88 | 0 | 2 | 0.205 | 1.000 | 0.200 | 417085 | 12702 | 404383 | 12708 | 404377 | 12571 | 137 | 404246 | 131 | 0.990 | 0.989 | 0.989 | - |
| **DHC3A** | 95 | 92 | 23 | 92 | 0 | 1 | 0.250 | 1.000 | 0.242 | 362204 | 14070 | 348134 | 13821 | 348383 | 13706 | 115 | 348019 | 364 | 0.974 | 0.992 | 0.982 | - |
| **DHC3B** | 79 | 78 | 12 | 78 | 0 | 0 | 0.154 | 1.000 | 0.152 | 302827 | 13745 | 289082 | 13605 | 289222 | 13512 | 93 | 288989 | 233 | 0.983 | 0.993 | 0.988 | - |
| **DHC4A** | 86 | 82 | 30 | 82 | 0 | 3 | 0.366 | 1.000 | 0.349 | 365115 | 13523 | 351592 | 13566 | 351549 | 13405 | 161 | 351431 | 118 | 0.991 | 0.988 | 0.989 | - |
| **DHC4B** | 80 | 79 | 18 | 79 | 0 | 1 | 0.228 | 1.000 | 0.225 | 119251 | 13284 | 105967 | 13113 | 106138 | 12985 | 128 | 105839 | 299 | 0.977 | 0.990 | 0.982 | - |
| **DHC4C** | 72 | 68 | 6 | 68 | 0 | 2 | 0.088 | 1.000 | 0.083 | 490267 | 13014 | 477253 | 12912 | 477355 | 12758 | 154 | 477099 | 256 | 0.980 | 0.988 | 0.984 | - |
| **DHC5** | 81 | 79 | 14 | 78 | 1 | 2 | 0.177 | 0.987 | 0.173 | 144290 | 13752 | 130538 | 13638 | 130652 | 13439 | 199 | 130339 | 313 | 0.977 | 0.985 | 0.979 | - |
| **DHC6** | 85 | 86 | 12 | 84 | 2 | 1 | 0.140 | 0.977 | 0.141 | 138640 | 13371 | 125269 | 13272 | 125368 | 12959 | 313 | 124956 | 412 | 0.969 | 0.976 | 0.970 | - |
| **DHC7A** | 67 | 64 | 21 | 63 | 1 | 1 | 0.328 | 0.984 | 0.313 | 257605 | 12058 | 245547 | 12114 | 245491 | 11882 | 232 | 245315 | 176 | 0.985 | 0.981 | 0.982 | - |
| **DHC7B** | 60 | 60 | 6 | 59 | 1 | 0 | 0.100 | 0.983 | 0.100 | 191156 | 11897 | 179259 | 11712 | 179444 | 11644 | 68 | 179191 | 253 | 0.979 | 0.994 | 0.986 | - |
| **DHC7C** | 72 | 73 | 17 | 72 | 1 | 0 | 0.233 | 0.986 | 0.236 | 205577 | 11880 | 193697 | 11754 | 193823 | 11676 | 78 | 193619 | 204 | 0.983 | 0.993 | 0.987 | - |
| **DHC8** | 76 | 73 | 15 | 73 | 0 | 1 | 0.205 | 1.000 | 0.197 | 84895 | 12190 | 72705 | 11952 | 72943 | 11817 | 135 | 72570 | 373 | 0.969 | 0.989 | 0.976 | - |
| **DHC9A** | 74 | 75 | 14 | 74 | 1 | 0 | 0.187 | 0.987 | 0.189 | 306568 | 12186 | 294382 | 11703 | 294865 | 11604 | 99 | 294283 | 582 | 0.952 | 0.992 | 0.971 | - |
| **DHC9B** | 85 | 85 | 9 | 79 | 6 | 2 | 0.106 | 0.929 | 0.106 | 373531 | 13791 | 359740 | 12417 | 361114 | 12214 | 203 | 359537 | 1577 | 0.886 | 0.984 | 0.932 | - |
| **DHC11** | 50 | 43 | 3 | 40 | 3 | 2 | 0.070 | 0.930 | 0.060 | 85205 | 14337 | 70868 | 13554 | 71651 | 13431 | 123 | 70745 | 906 | 0.937 | 0.991 | 0.957 | - |
| **Others** | **86** | **93** | **21** | **82** | **11** | **0** | **0.226** | **0.882** | **0.244** | **108735** | **14186** | **94549** | **13917** | **94818** | **13810** | **107** | **94442** | **376** | **0.973** | **0.992** | **0.980** | **-** |
| **NedKinesinJ_fl** | 6 | 3 | 1 | 3 | 0 | 0 | 0.333 | 1.000 | 0.167 | 6820 | 2273 | 4547 | 2238 | 4582 | 2235 | 3 | 4544 | 38 | 0.983 | 0.999 | 0.986 | - |
| **PhsP62_fl** | 4 | 3 | 0 | 3 | 0 | 0 | 0.000 | 1.000 | 0.000 | 5645 | 1506 | 4139 | 1428 | 4217 | 1419 | 9 | 4130 | 87 | 0.942 | 0.994 | 0.957 | - |
| **HsP50_fl** | 13 | 16 | 3 | 13 | 3 | 0 | 0.188 | 0.813 | 0.231 | 20372 | 1218 | 19154 | 1167 | 19205 | 1158 | 9 | 19145 | 60 | 0.951 | 0.992 | 0.970 | - |
| **PugCor_fl** | 8 | 10 | 2 | 8 | 2 | 0 | 0.200 | 0.800 | 0.250 | 6606 | 1617 | 4989 | 1602 | 5004 | 1597 | 5 | 4984 | 20 | 0.988 | 0.997 | 0.990 | - |
| **MmP150_fl** | 28 | 32 | 8 | 28 | 4 | 0 | 0.250 | 0.875 | 0.286 | 23936 | 3843 | 20093 | 3759 | 20177 | 3729 | 30 | 20063 | 114 | 0.970 | 0.992 | 0.978 | - |
| **HsMyo19_fl** | 23 | 24 | 6 | 23 | 1 | 0 | 0.250 | 0.958 | 0.261 | 40271 | 2910 | 37361 | 2904 | 37367 | 2874 | 30 | 37331 | 36 | 0.988 | 0.990 | 0.988 | - |
| **ThCAP1_fl** | 4 | 5 | 1 | 4 | 1 | 0 | 0.200 | 0.800 | 0.250 | 5085 | 819 | 4266 | 819 | 4266 | 798 | 21 | 4245 | 21 | 0.974 | 0.974 | 0.969 | - |

**Blat (tilesize 6)**

| | Exon PE | Exon AE | Exon TE | Exon OE | Exon ME | Exon WE | Exon Sn | Exon Sno | Exon Sp | Nucl Total | Nucl AP | Nucl AN | Nucl PP | Nucl PN | Nucl TP | Nucl FP | Nucl TN | Nucl FN | Nucl Sn | Nucl Sp | Nucl AC | Genes |
|---|---|---|---|---|---|---|---|---|---|---|---|---|---|---|---|---|---|---|---|---|---|---|
| **DHC all** | **1349** | **1202** | **236** | **1196** | **6** | **105** | **0.196** | **0.995** | **0.175** | **3910464** | **209483** | **3700981** | **209223** | **3701241** | **204724** | **4499** | **3696482** | **4759** | **0.977** | **0.978** | **0.977** | **-** |
| **DHC1** | 80 | 77 | 21 | 77 | 0 | 2 | 0.273 | 1.000 | 0.263 | 66248 | 13683 | 52565 | 13725 | 52523 | 13605 | 120 | 52445 | 78 | 0.994 | 0.991 | 0.991 | - |
| **DHC2** | 102 | 88 | 16 | 88 | 0 | 13 | 0.182 | 1.000 | 0.157 | 417085 | 12702 | 404383 | 13008 | 404077 | 12567 | 441 | 403942 | 135 | 0.989 | 0.966 | 0.977 | - |
| **DHC3A** | 99 | 92 | 23 | 92 | 0 | 5 | 0.250 | 1.000 | 0.232 | 362204 | 14070 | 348134 | 14004 | 348200 | 13802 | 202 | 347932 | 268 | 0.981 | 0.986 | 0.983 | - |
| **DHC3B** | 84 | 78 | 12 | 78 | 0 | 4 | 0.154 | 1.000 | 0.143 | 302827 | 13745 | 289082 | 13722 | 289105 | 13545 | 177 | 288905 | 200 | 0.985 | 0.987 | 0.986 | - |
| **DHC4A** | 96 | 82 | 28 | 82 | 0 | 9 | 0.341 | 1.000 | 0.292 | 365115 | 13523 | 351592 | 13722 | 351393 | 13405 | 317 | 351275 | 118 | 0.991 | 0.977 | 0.983 | - |
| **DHC4B** | 83 | 79 | 19 | 79 | 0 | 2 | 0.241 | 1.000 | 0.229 | 119251 | 13284 | 105967 | 13164 | 106087 | 13006 | 158 | 105809 | 278 | 0.979 | 0.988 | 0.981 | - |
| **DHC4C** | 90 | 68 | 6 | 68 | 0 | 19 | 0.088 | 1.000 | 0.067 | 490267 | 13014 | 477253 | 13422 | 476845 | 12787 | 635 | 476618 | 227 | 0.983 | 0.953 | 0.967 | - |
| **DHC5** | 83 | 79 | 14 | 79 | 0 | 3 | 0.177 | 1.000 | 0.169 | 144290 | 13752 | 130538 | 13704 | 130586 | 13469 | 235 | 130303 | 283 | 0.979 | 0.983 | 0.979 | - |
| **DHC6** | 86 | 86 | 12 | 85 | 1 | 1 | 0.140 | 0.988 | 0.140 | 138640 | 13371 | 125269 | 13311 | 125329 | 13003 | 308 | 124961 | 368 | 0.972 | 0.977 | 0.972 | - |
| **DHC7A** | 78 | 64 | 22 | 64 | 0 | 12 | 0.344 | 1.000 | 0.282 | 257605 | 12058 | 245547 | 12450 | 245155 | 11978 | 472 | 245075 | 80 | 0.993 | 0.962 | 0.977 | - |
| **DHC7B** | 64 | 60 | 6 | 60 | 0 | 3 | 0.100 | 1.000 | 0.094 | 191156 | 11897 | 179259 | 11844 | 179312 | 11709 | 135 | 179124 | 188 | 0.984 | 0.989 | 0.985 | - |
| **DHC7C** | 77 | 73 | 17 | 73 | 0 | 4 | 0.233 | 1.000 | 0.221 | 205577 | 11880 | 193697 | 11958 | 193619 | 11760 | 198 | 193499 | 120 | 0.990 | 0.983 | 0.986 | - |
| **DHC8** | 81 | 73 | 14 | 73 | 0 | 5 | 0.192 | 1.000 | 0.173 | 84895 | 12190 | 72705 | 12027 | 72868 | 11817 | 210 | 72495 | 373 | 0.969 | 0.983 | 0.972 | - |
| **DHC9A** | 78 | 75 | 14 | 75 | 0 | 2 | 0.187 | 1.000 | 0.179 | 306568 | 12186 | 294382 | 11925 | 294643 | 11786 | 139 | 294243 | 400 | 0.967 | 0.988 | 0.977 | - |
| **DHC9B** | 108 | 85 | 8 | 81 | 4 | 15 | 0.094 | 0.953 | 0.074 | 373531 | 13791 | 359740 | 13353 | 360178 | 12782 | 571 | 359169 | 1009 | 0.927 | 0.957 | 0.940 | - |
| **DHC11** | 60 | 43 | 4 | 42 | 1 | 6 | 0.093 | 0.977 | 0.067 | 85205 | 14337 | 70868 | 13884 | 71321 | 13703 | 181 | 70687 | 634 | 0.956 | 0.987 | 0.966 | - |
| **Others** | **78** | **87** | **22** | **78** | **9** | **0** | **0.253** | **0.897** | **0.282** | **96270** | **10407** | **85863** | **10320** | **85950** | **10225** | **95** | **85768** | **182** | **0.983** | **0.991** | **0.985** | **5** |
| **HsP50_fl** | 14 | 16 | 4 | 14 | 2 | 0 | 0.250 | 0.875 | 0.286 | 20372 | 1218 | 19154 | 1203 | 19169 | 1194 | 9 | 19145 | 24 | 0.980 | 0.993 | 0.986 | 1 |
| **PugCor_fl** | 8 | 10 | 2 | 8 | 2 | 0 | 0.200 | 0.800 | 0.250 | 6606 | 1617 | 4989 | 1602 | 5004 | 1597 | 5 | 4984 | 20 | 0.988 | 0.997 | 0.990 | 1 |
| **MmP150_fl** | 29 | 32 | 9 | 29 | 3 | 0 | 0.281 | 0.906 | 0.310 | 23936 | 3843 | 20093 | 3792 | 20144 | 3762 | 30 | 20063 | 81 | 0.979 | 0.992 | 0.983 | 1 |
| **HsMyo19_fl** | 23 | 24 | 6 | 23 | 1 | 0 | 0.250 | 0.958 | 0.261 | 40271 | 2910 | 37361 | 2904 | 37367 | 2874 | 30 | 37331 | 36 | 0.988 | 0.990 | 0.988 | 1 |
| **ThCAP1_fl** | 4 | 5 | 1 | 4 | 1 | 0 | 0.200 | 0.800 | 0.250 | 5085 | 819 | 4266 | 819 | 4266 | 798 | 21 | 4245 | 21 | 0.974 | 0.974 | 0.969 | 1 |

**Blat (tilesize 5)**

| | Exon PE | Exon AE | Exon TE | Exon OE | Exon ME | Exon WE | Exon Sn | Exon Sno | Exon Sp | Nucl Total | Nucl AP | Nucl AN | Nucl PP | Nucl PN | Nucl TP | Nucl FP | Nucl TN | Nucl FN | Nucl Sn | Nucl Sp | Nucl AC | Genes |
|---|---|---|---|---|---|---|---|---|---|---|---|---|---|---|---|---|---|---|---|---|---|---|
| **DHC all** | **3292** | **1202** | **239** | **1199** | **3** | **1846** | **0.199** | **0.998** | **0.073** | **3910464** | **209486** | **3700978** | **242926** | **3667538** | **205432** | **37494** | **3663484** | **4054** | **0.981** | **0.846** | **0.908** | **-** |
| **DHC1** | 89 | 77 | 21 | 77 | 0 | 10 | 0.273 | 1.000 | 0.236 | 66248 | 13686 | 52562 | 13886 | 52362 | 13607 | 279 | 52283 | 79 | 0.994 | 0.980 | 0.984 | - |
| **DHC2** | 341 | 88 | 17 | 88 | 0 | 233 | 0.193 | 1.000 | 0.050 | 417085 | 12702 | 404383 | 17099 | 399986 | 12567 | 4532 | 399851 | 135 | 0.989 | 0.735 | 0.856 | - |
| **DHC3A** | 272 | 92 | 21 | 92 | 0 | 169 | 0.228 | 1.000 | 0.077 | 362204 | 14070 | 348134 | 17160 | 345044 | 13790 | 3370 | 344764 | 280 | 0.980 | 0.804 | 0.887 | - |
| **DHC3B** | 249 | 78 | 12 | 78 | 0 | 156 | 0.154 | 1.000 | 0.048 | 302827 | 13745 | 289082 | 16690 | 286137 | 13577 | 3113 | 285969 | 168 | 0.988 | 0.813 | 0.895 | - |
| **DHC4A** | 264 | 82 | 31 | 82 | 0 | 172 | 0.378 | 1.000 | 0.117 | 365115 | 13523 | 351592 | 16708 | 348407 | 13405 | 3303 | 348289 | 118 | 0.991 | 0.802 | 0.892 | - |
| **DHC4B** | 111 | 79 | 19 | 79 | 0 | 21 | 0.241 | 1.000 | 0.171 | 119251 | 13284 | 105967 | 13662 | 105589 | 13078 | 584 | 105383 | 206 | 0.984 | 0.957 | 0.967 | - |
| **DHC4C** | 357 | 68 | 6 | 68 | 0 | 277 | 0.088 | 1.000 | 0.017 | 490267 | 13014 | 477253 | 18084 | 472183 | 12803 | 5281 | 471972 | 211 | 0.984 | 0.708 | 0.840 | - |

| | | | | | | | | | | | | | | | | | | | | | |
|---|---|---|---|---|---|---|---|---|---|---|---|---|---|---|---|---|---|---|---|---|---|
| **DHC5** | 133 | 79 | 15 | 79 | 0 | 49 | 0.190 | 1.000 | 0.113 | 144290 | 13752 | 130538 | 14685 | 129605 | 13475 | 1210 | 129328 | 277 | 0.980 | 0.918 | 0.943 | - |
| **DHC6** | 132 | 86 | 12 | 85 | 1 | 43 | 0.140 | 0.988 | 0.091 | 138640 | 13371 | 125269 | 14153 | 124487 | 12999 | 1154 | 124115 | 372 | 0.972 | 0.918 | 0.939 | - |
| **DHC7A** | 158 | 64 | 21 | 64 | 0 | 83 | 0.328 | 1.000 | 0.133 | 257605 | 12058 | 245547 | 13837 | 243768 | 11960 | 1877 | 243670 | 98 | 0.992 | 0.864 | 0.924 | - |
| **DHC7B** | 134 | 60 | 6 | 60 | 0 | 66 | 0.100 | 1.000 | 0.045 | 191156 | 11897 | 179259 | 13173 | 177983 | 11741 | 1432 | 177827 | 156 | 0.987 | 0.891 | 0.935 | - |
| **DHC7C** | 127 | 73 | 17 | 73 | 0 | 49 | 0.233 | 1.000 | 0.134 | 205577 | 11880 | 193697 | 12754 | 192823 | 11763 | 991 | 192706 | 117 | 0.990 | 0.922 | 0.953 | - |
| **DHC8** | 145 | 73 | 15 | 73 | 0 | 56 | 0.205 | 1.000 | 0.103 | 84895 | 12190 | 72705 | 13183 | 71712 | 11945 | 1238 | 71467 | 245 | 0.980 | 0.906 | 0.933 | - |
| **DHC9A** | 231 | 75 | 15 | 75 | 0 | 142 | 0.200 | 1.000 | 0.065 | 306568 | 12186 | 294382 | 14620 | 291948 | 11895 | 2725 | 291657 | 291 | 0.976 | 0.814 | 0.890 | - |
| **DHC9B** | 326 | 85 | 7 | 84 | 1 | 220 | 0.082 | 0.988 | 0.021 | 373531 | 13791 | 359740 | 17453 | 356078 | 12956 | 4497 | 355243 | 835 | 0.939 | 0.742 | 0.833 | - |
| **DHC11** | 223 | 43 | 4 | 42 | 1 | 100 | 0.093 | 0.977 | 0.018 | 85205 | 14337 | 70868 | 15779 | 69426 | 13871 | 1908 | 68960 | 466 | 0.967 | 0.879 | 0.906 | - |
| **Others** | **77** | **87** | **21** | **77** | **10** | **0** | **0.241** | **0.885** | **0.273** | **96270** | **10407** | **85863** | **10299** | **85971** | **10204** | **95** | **85768** | **203** | **0.980** | **0.991** | **0.984** | **5** |
| **HsP50_fl** | 14 | 16 | 4 | 14 | 2 | 0 | 0.250 | 0.875 | 0.286 | 20372 | 1218 | 19154 | 1203 | 19169 | 1194 | 9 | 19145 | 24 | 0.980 | 0.993 | 0.986 | 1 |
| **PugCor_fl** | 8 | 10 | 2 | 8 | 2 | 0 | 0.200 | 0.800 | 0.250 | 6606 | 1617 | 4989 | 1602 | 5004 | 1597 | 5 | 4984 | 20 | 0.988 | 0.997 | 0.990 | 1 |
| **MmP150_fl** | 28 | 32 | 8 | 28 | 4 | 0 | 0.250 | 0.875 | 0.286 | 23936 | 3843 | 20093 | 3771 | 20165 | 3741 | 30 | 20063 | 102 | 0.973 | 0.992 | 0.979 | 1 |
| **HsMyo19_fl** | 23 | 24 | 6 | 23 | 1 | 0 | 0.250 | 0.958 | 0.261 | 40271 | 2910 | 37361 | 2904 | 37367 | 2874 | 30 | 37331 | 36 | 0.988 | 0.990 | 0.988 | 1 |
| **ThCAP1_fl** | 4 | 5 | 1 | 4 | 1 | 0 | 0.200 | 0.800 | 0.250 | 5085 | 819 | 4266 | 819 | 4266 | 798 | 21 | 4245 | 21 | 0.974 | 0.974 | 0.969 | 1 |

**Blast**

| | **Exon PE** | **Exon AE** | **Exon TE** | **Exon OE** | **Exon ME** | **Exon WE** | **Exon Sn** | **Exon Sno** | **Exon Sp** | **Nucl Total** | **Nucl AP** | **Nucl AN** | **Nucl PP** | **Nucl PN** | **Nucl TP** | **Nucl FP** | **Nucl TN** | **Nucl FN** | **Nucl Sn** | **Nucl Sp** | **Nucl AC** | **Genes** |
|---|---|---|---|---|---|---|---|---|---|---|---|---|---|---|---|---|---|---|---|---|---|---|
| **DHC all** | **1201** | **1202** | **48** | **1189** | **13** | **30** | **0.040** | **0.989** | **0.040** | **3910464** | **209483** | **3700981** | **230601** | **3679863** | **205228** | **25373** | **3675608** | **4255** | **0.980** | **0.890** | **0.931** | **-** |
| **DHC1** | 67 | 77 | 0 | 76 | 1 | 0 | 0.000 | 0.987 | 0.000 | 66248 | 13683 | 52565 | 16167 | 50081 | 13545 | 2622 | 49943 | 138 | 0.990 | 0.838 | 0.888 | - |
| **DHC2** | 86 | 88 | 6 | 87 | 1 | 1 | 0.068 | 0.989 | 0.070 | 417085 | 12702 | 404383 | 13566 | 403519 | 12499 | 1067 | 403316 | 203 | 0.984 | 0.921 | 0.951 | - |
| **DHC3A** | 91 | 92 | 4 | 92 | 0 | 1 | 0.043 | 1.000 | 0.044 | 362204 | 14070 | 348134 | 15480 | 346724 | 13766 | 1714 | 346420 | 304 | 0.978 | 0.889 | 0.931 | - |
| **DHC3B** | 84 | 78 | 2 | 78 | 0 | 5 | 0.026 | 1.000 | 0.024 | 302827 | 13745 | 289082 | 15536 | 287291 | 13565 | 1971 | 287111 | 180 | 0.987 | 0.873 | 0.926 | - |
| **DHC4A** | 82 | 82 | 6 | 81 | 1 | 2 | 0.073 | 0.988 | 0.073 | 365115 | 13523 | 351592 | 14718 | 350397 | 13201 | 1517 | 350075 | 322 | 0.976 | 0.897 | 0.934 | - |
| **DHC4B** | 81 | 79 | 2 | 78 | 1 | 2 | 0.025 | 0.987 | 0.025 | 119251 | 13284 | 105967 | 14161 | 105090 | 12944 | 1217 | 104750 | 340 | 0.974 | 0.914 | 0.937 | - |
| **DHC4C** | 71 | 68 | 1 | 68 | 0 | 1 | 0.015 | 1.000 | 0.014 | 490267 | 13014 | 477253 | 13959 | 476308 | 12675 | 1284 | 475969 | 339 | 0.974 | 0.908 | 0.939 | - |
| **DHC5** | 77 | 79 | 2 | 77 | 2 | 0 | 0.025 | 0.975 | 0.026 | 144290 | 13752 | 130538 | 14829 | 129461 | 13477 | 1352 | 129186 | 275 | 0.980 | 0.909 | 0.938 | - |
| **DHC6** | 82 | 86 | 3 | 85 | 1 | 3 | 0.035 | 0.988 | 0.037 | 138640 | 13371 | 125269 | 15312 | 123328 | 13004 | 2308 | 122961 | 367 | 0.973 | 0.849 | 0.900 | - |
| **DHC7A** | 69 | 64 | 7 | 64 | 0 | 2 | 0.109 | 1.000 | 0.101 | 257605 | 12058 | 245547 | 13351 | 244254 | 11950 | 1401 | 244146 | 108 | 0.991 | 0.895 | 0.940 | - |
| **DHC7B** | 62 | 60 | 2 | 60 | 0 | 2 | 0.033 | 1.000 | 0.032 | 191156 | 11897 | 179259 | 13149 | 178007 | 11744 | 1405 | 177854 | 153 | 0.987 | 0.893 | 0.936 | - |
| **DHC7C** | 72 | 73 | 4 | 72 | 1 | 0 | 0.055 | 0.986 | 0.056 | 205577 | 11880 | 193697 | 12567 | 193010 | 11676 | 891 | 192806 | 204 | 0.983 | 0.929 | 0.953 | - |
| **DHC8** | 76 | 73 | 2 | 73 | 0 | 3 | 0.027 | 1.000 | 0.026 | 84895 | 12190 | 72705 | 14132 | 70763 | 12048 | 2084 | 70621 | 142 | 0.988 | 0.853 | 0.905 | - |
| **DHC9A** | 77 | 75 | 5 | 75 | 0 | 3 | 0.067 | 1.000 | 0.065 | 306568 | 12186 | 294382 | 13920 | 292648 | 12087 | 1833 | 292549 | 99 | 0.992 | 0.868 | 0.927 | - |
| **DHC9B** | 85 | 85 | 2 | 82 | 3 | 3 | 0.024 | 0.965 | 0.024 | 373531 | 13791 | 359740 | 14583 | 358948 | 13258 | 1325 | 358415 | 533 | 0.961 | 0.909 | 0.933 | - |
| **DHC11** | 39 | 43 | 0 | 41 | 2 | 2 | 0.000 | 0.953 | 0.000 | 85205 | 14337 | 70868 | 15171 | 70034 | 13789 | 1382 | 69486 | 548 | 0.962 | 0.909 | 0.922 | - |
| **Others** | **86** | **93** | **5** | **83** | **10** | **11** | **0.054** | **0.892** | **0.058** | **108735** | **14186** | **94549** | **16534** | **92201** | **13613** | **2921** | **91628** | **573** | **0.960** | **0.823** | **0.873** | **-** |
| **NedKinesinJ_fl** | 4 | 3 | 0 | 3 | 0 | 0 | 0.000 | 1.000 | 0.000 | 6820 | 2273 | 4547 | 2331 | 4489 | 2271 | 60 | 4487 | 2 | 0.999 | 0.974 | 0.980 | - |
| **PhsP62_fl** | 6 | 3 | 0 | 3 | 0 | 4 | 0.000 | 1.000 | 0.000 | 5645 | 1506 | 4139 | 1896 | 3749 | 1488 | 408 | 3731 | 18 | 0.988 | 0.785 | 0.835 | - |
| **HsP50_fl** | 14 | 16 | 2 | 14 | 2 | 0 | 0.125 | 0.875 | 0.143 | 20372 | 1218 | 19154 | 1212 | 19160 | 1088 | 124 | 19030 | 130 | 0.893 | 0.898 | 0.889 | - |
| **PugCor_fl** | 6 | 10 | 0 | 8 | 2 | 2 | 0.000 | 0.800 | 0.000 | 6606 | 1617 | 4989 | 2049 | 4557 | 1600 | 449 | 4540 | 17 | 0.989 | 0.781 | 0.838 | - |
| **MmP150_fl** | 26 | 32 | 2 | 27 | 5 | 1 | 0.063 | 0.844 | 0.077 | 23936 | 3843 | 20093 | 4215 | 19721 | 3468 | 747 | 19346 | 375 | 0.902 | 0.823 | 0.835 | - |
| **HsMyo19_fl** | 28 | 24 | 1 | 23 | 1 | 4 | 0.042 | 0.958 | 0.036 | 40271 | 2910 | 37361 | 3808 | 36463 | 2880 | 928 | 36433 | 30 | 0.990 | 0.756 | 0.860 | - |
| **ThCAP1_fl** | 2 | 5 | 0 | 5 | 0 | 0 | 0.000 | 1.000 | 0.000 | 5085 | 819 | 4266 | 1023 | 4062 | 818 | 205 | 4061 | 1 | 0.999 | 0.800 | 0.875 | - |
