# Supplementary material for: Cross-species protein sequence and gene structure prediction with fine-tuned Webscipio 2.0 and Scipio
Source: BMC Res Notes. 2011 Jul 28;4:265. doi: 10.1186/1756-0500-4-265 (PMC3162530; doi:10.1186/1756-0500-4-265)
Supplement: Additional file 5 — Software versions and run parameters of the gene reconstruction and prediction tools. The tables shows the exact versions and run parameters, which were used for the comparison, for each scenario. [file 1756-0500-4-265-S5.PDF]

Detailed versions and parameters of the gene reconstruction and prediction tools

Scenario 1

Tool	Version	Parameters
Scipio v1.0	Scipio: v1.0<br>Blat: v. 34	Scipio: --min_identity=0.6 --min_score=0.3 --max_mismatch=0<br>Blat: -tileSize=7 -minIdentity=54 -minScore=15 -oneOff=1
Scipio v1.5	Scipio: v1.5<br>Blat: v. 34	Scipio: --exhaust_gap_size=30 --max_assemble_size=75000 --min_identity=60 --accepted_intron_penalty=1.0 --min_score=0.3 --multiple_results --gap_to_close=15 --min_intron_len=22 --exhaust_align_size=15000 --max_mismatch=0 --max_move_exon=6 --min_coverage=60<br>Blat: -tileSize=7 -minIdentity=54 -minScore=15 -oneOff=1
Scipio v1.5<br>(1 gene)	Scipio: v1.5<br>Blat: v. 34	Scipio: --exhaust_gap_size=30 --max_assemble_size=75000 --min_identity=60 --accepted_intron_penalty=1.0 --min_score=0.3 --gap_to_close=15 --min_intron_len=22 --exhaust_align_size=15000 --max_mismatch=0 --max_move_exon=6 --min_coverage=60<br>Blat: -tileSize=7 -minIdentity=54 -minScore=15 -oneOff=1
Prosplign	07/09/07	-full -two_stages
Prosplign<br>(Blast)	Prosplign: 07/09/07<br>Blast: 2.2.20<br>Compart: 07/09/07	Prosplign: -full -two_stages<br>Compart: -add 10000
Prosplign<br>(Blat)	Prosplign: 07/09/07<br>Blat: v. 34<br>Compart: 07/09/07	Prosplign: -full -two_stages<br>Blat: -tileSize=7 -minIdentity=54 -minScore=15 -oneOff=1<br>Compart: -add 10000
Exonerate	2.2.0	--model protein2genome
Exonerate<br>(1 gene)	2.2.0	--model protein2genome --bestn 1
Wise2	2.2.0	-both
Wise2 (global)	2.2.0	-both -init global
Augustus	2.5	--species=human --strand=both --singlestrand=false --genemodel=partial --codingseq=on --sample=100 --keep_viterbi=true --alternatives-from-sampling=false --exonnames=on
Blat	v. 34	-tileSize=7 -minIdentity=54 -minScore=15 -oneOff=1
Blast	2.2.20	

Scenario 2

Tool	Version	Parameters
Scipio v1.0	Scipio: v1.0<br>Blat: v. 34	Scipio: --min_identity=0.6 --min_score=0.3 --max_mismatch=0<br>Blat: -tileSize=7 -minIdentity=54 -minScore=15 -oneOff=1
Scipio v1.5	Scipio: v1.5<br>Blat: v. 34	Scipio: --exhaust_gap_size=30 --max_assemble_size=75000 --min_identity=60 --accepted_intron_penalty=1.0 --min_score=0.3 --multiple_results --gap_to_close=15 --min_intron_len=22 --exhaust_align_size=15000 --max_mismatch=0 --max_move_exon=6 --min_coverage=60<br>Blat: -tileSize=7 -minIdentity=54 -minScore=15 -oneOff=1
Scipio v1.5 (Blat tilesize 6)	Scipio: v1.5<br>Blat: v. 34	Scipio: --exhaust_gap_size=30 --max_assemble_size=75000 --min_identity=60 --accepted_intron_penalty=1.0 --min_score=0.3 --multiple_results --gap_to_close=15 --min_intron_len=22 --exhaust_align_size=15000 --max_mismatch=0 --max_move_exon=6 --min_coverage=60<br>Blat: -tileSize=6 -minIdentity=54 -minScore=15 -oneOff=1
Scipio v1.5 (Blat tilesize 5)	Scipio: v1.5<br>Blat: v. 34	Scipio: --exhaust_gap_size=30 --max_assemble_size=75000 --min_identity=60 --accepted_intron_penalty=1.0 --min_score=0.3 --multiple_results --gap_to_close=15 --min_intron_len=22 --exhaust_align_size=15000 --max_mismatch=0 --max_move_exon=6 --min_coverage=60<br>Blat: -tileSize=5 -minIdentity=54 -minScore=15 -oneOff=1
Prospglign	07/09/07	-full -two_stages
Prospglign (Blast)	Prospglign: 07/09/07<br>Blast: 2.2.20<br>Compart: 07/09/07	Prospglign: -full -two_stages<br>Compart: -add 10000
Prospglign (Blat)	Prospglign: 07/09/07<br>Blat: v. 34<br>Compart: 07/09/07	Prospglign: -full -two_stages<br>Blat: -tileSize=7 -minIdentity=54 -minScore=15 -oneOff=1<br>Compart: -add 10000
Exonerate	2.2.0	--model protein2genome
Exonerate (1 gene)	2.2.0	--model protein2genome --bestn 1
Prot_map	24. Jul 2007	Similarity: Weak; Search for one best alignment only; Maximal area covered by alignment on target: 1000000; Remove trailing X; Protein mode: Map whole protein; Join similar aminoacids: no join
Fgenesh+	24. Jul 2007	Organism: Human
Wise2	2.2.0	-both
Wise2 (global)	2.2.0	-both -init global
Augustus	2.5	--species=human --strand=both --singlestrand=false --genemodel=partial --codingseq=on --sample=100 --keep_viterbi=true --alternatives-from-sampling=false --exonnames=on
Augustus (1 gene)	2.5	--species=human --strand=both --singlestrand=false --genemodel=exactlyone --codingseq=on --sample=100 --keep_viterbi=true --alternatives-from-sampling=false --exonnames=on
Fgenesh	24. Jul 2007	Organism: Human
Genscan	November, 2009	Organism: Vertebrate; Suboptimal exon cutoff: 1.00
Blat	v. 34	-tileSize=7 -minIdentity=54 -minScore=15 -oneOff=1
Blat (tilesize 6)	v. 34	-tileSize=6 -minIdentity=54 -minScore=15 -oneOff=1
Blat (tilesize 5)	v. 34	-tileSize=5 -minIdentity=54 -minScore=15 -oneOff=1
Blast	2.2.20	

Scenario 3

Tool	Version	Parameters	Organism specific parameters
Scipio v1.0	Scipio: v1.0<br>Blat: v. 34	Scipio: --min_score=0.3<br>Blat: -minScore=15	Scipio:<br>Ned, Phs: --min_identity=0.6<br>--max_mismatch=0<br>Hs, Pug, Mm, Th: --min_identity=0.9<br>--max_mismatch=7<br>Blat:<br>Ned, Phs: -tileSize=5 -minIdentity=54<br>-oneOff=1<br>Hs, Pug, Mm, Th: -tileSize=7<br>-minIdentity=81
Scipio v1.5	Scipio: v1.5<br>Blat: v. 34	Scipio: --exhaust_gap_size=30<br>--max_assemble_size=75000<br>--accepted_intron_penalty=1.0<br>--min_score=0.3 --multiple_results<br>--min_intron_len=22<br>--exhaust_align_size=15000<br>--max_move_exon=6<br>--min_coverage=60<br>Blat: -minScore=15	Scipio:<br>Ned, Phs: --min_identity=60<br>--gap_to_close=15 --max_mismatch=0<br>Hs, Pug, Mm, Th: --gap_to_close=6<br>--max_mismatch=7<br>Blat:<br>Ned, Phs: -tileSize=5 -minIdentity=54<br>-oneOff=1<br>Hs, Pug, Mm, Th: -tileSize=7<br>-minIdentity=81
Scipio v1.5<br>(1 gene)	Scipio: v1.5<br>Blat: v. 34	Scipio: --exhaust_gap_size=30<br>--max_assemble_size=75000<br>--accepted_intron_penalty=1.0<br>--min_score=0.3 --min_intron_len=22<br>--exhaust_align_size=15000<br>--max_move_exon=6<br>--min_coverage=60<br>Blat: -minScore=15	Scipio:<br>Ned, Phs: --min_identity=60<br>--gap_to_close=15 --max_mismatch=0<br>Hs, Pug, Mm, Th: --gap_to_close=6<br>--max_mismatch=7<br>Blat:<br>Ned, Phs: -tileSize=5 -minIdentity=54<br>-oneOff=1<br>Hs, Pug, Mm, Th: -tileSize=7<br>-minIdentity=81
Prosplign	07/09/07	-full -two_stages	
Prosplign<br>(Blast)	Prosplign:<br>07/09/07<br>Blast: 2.2.20<br>Compart:<br>07/09/07	Prosplign: -full -two_stages<br>Compart: -add 10000	
Prosplign<br>(Blat)	Prosplign:<br>07/09/07<br>Blat: v. 34<br>Compart:<br>07/09/07	Prosplign: -full -two_stages<br>Blat: -minScore=15<br>Compart: -add 10000	Blat:<br>Ned, Phs: -tileSize=5 -minIdentity=54<br>-oneOff=1<br>Hs, Pug, Mm, Th: -tileSize=7<br>-minIdentity=81
Exonerate	2.2.0	--model protein2genome	
Exonerate<br>(1 gene)	2.2.0	--model protein2genome --bestn 1	
Wise2	2.2.0	-both	
Wise2 (global)	2.2.0	-both -init global	
Augustus	2.5	--strand=both --singlestrand=false<br>--genemodel=partial --codingseq=on<br>--sample=100 --keep_viterbi=true<br>--alternatives-from-sampling=false<br>--exonnames=on	Ned: --species=neurospora<br>Phs, Pug, Th: --species=generic<br>Hs, Mm: --species=human
Blat	v. 34	-minScore=15	Ned, Phs: -tileSize=5 -minIdentity=54<br>-oneOff=1<br>Hs, Pug, Mm, Th: -tileSize=7<br>-minIdentity=81
Blast	2.2.20		

Ned: *Neurospora discreta*, Phs: *Phytophthora sojae*, Hs: *Homo sapiens*, Pug: *Puccinia graminis*, Mm: *Mus musculus*, Th: *Thielavia heterothallica*

Scenario 4

Tool	Version	Parameters	Organism specific parameters
Scipio v1.0	Scipio: v1.0<br>Blat: v. 34	Scipio: --min_score=0.3<br>Blat: -minScore=15	Scipio:<br>Ned, Phs: --min_identity=0.6<br>--max_mismatch=0<br>Hs, Pug, Mm, Th: --min_identity=0.9<br>--max_mismatch=7<br>Blat:<br>Ned, Phs: -tileSize=5 -minIdentity=54<br>-oneOff=1<br>Hs, Pug, Mm, Th: -tileSize=7<br>-minIdentity=81
Scipio v1.5	Scipio: v1.5<br>Blat: v. 34	Scipio: --exhaust_gap_size=30<br>--max_assemble_size=75000<br>--accepted_intron_penalty=1.0<br>--min_score=0.3 --multiple_results<br>--min_intron_len=22<br>--exhaust_align_size=15000<br>--max_move_exon=6<br>--min_coverage=60<br>Blat: -minScore=15	Scipio:<br>Ned, Phs: --min_identity=60<br>--gap_to_close=15 --max_mismatch=0<br>Hs, Pug, Mm, Th: --gap_to_close=6<br>--max_mismatch=7<br>Blat:<br>Ned, Phs: -tileSize=5 -minIdentity=54<br>-oneOff=1<br>Hs, Pug, Mm, Th: -tileSize=7<br>-minIdentity=81
Scipio v1.5<br>(Blat tilesize 6)	Scipio: v1.5<br>Blat: v. 34	Scipio: --exhaust_gap_size=30<br>--max_assemble_size=75000<br>--accepted_intron_penalty=1.0<br>--min_score=0.3 --multiple_results<br>--min_intron_len=22<br>--exhaust_align_size=15000<br>--max_move_exon=6<br>--min_coverage=60<br>Blat: -tileSize=6 -minIdentity=81<br>-minScore=15	Scipio:<br>Hs, Pug, Mm, Th: --gap_to_close=6<br>--max_mismatch=7
Scipio v1.5<br>(Blat tilesize 5)	Scipio: v1.5<br>Blat: v. 34	Scipio: --exhaust_gap_size=30<br>--max_assemble_size=75000<br>--accepted_intron_penalty=1.0<br>--min_score=0.3 --multiple_results<br>--min_intron_len=22<br>--exhaust_align_size=15000<br>--max_move_exon=6<br>--min_coverage=60<br>Blat: -tileSize=5 -minIdentity=81<br>-minScore=15	Scipio:<br>Hs, Pug, Mm, Th: --gap_to_close=6<br>--max_mismatch=7
Prospligh	07/09/07	-full -two_stages	
Prospligh<br>(Blast)	Prospligh:<br>07/09/07<br>Blast: 2.2.20<br>Compart:<br>07/09/07	Prospligh: -full -two_stages<br>Compart: -add 10000	
Prospligh<br>(Blat)	Prospligh:<br>07/09/07<br>Blat: v. 34<br>Compart:<br>07/09/07	Prospligh: -full -two_stages<br>Blat: -minScore=15<br>Compart: -add 10000	Blat:<br>Ned, Phs: -tileSize=5 -minIdentity=54<br>-oneOff=1<br>Hs, Pug, Mm, Th: -tileSize=7<br>-minIdentity=81
Exonerate	2.2.0	--model protein2genome	
Exonerate<br>(1 gene)	2.2.0	--model protein2genome --bestn 1	
Prot_map	24. Jul 2007	Similarity: Weak; Search for one best alignment only; Maximal area covered by alignment on target: 1000000; Remove trailing X; Protein mode: Map whole protein; Join similar aminoacids: no join	
Fgenesh+	24. Jul 2007		Ned, Th: Organism: Neurospora crassa<br>Phs: Organism: Phytophtora<br>Hs: Organism: Human<br>Pug: Organism: Puccina<br>Mm: Organism: Mouse
Wise2	2.2.0	-both	

Wise2 (global)	2.2.0	-both -init global	
Augustus	2.5	--strand=both --singlestrand=false<br>--genemodel=partial --codingseq=on<br>--sample=100 --keep_viterbi=true<br>--alternatives-from-sampling=false<br>--exonnames=on	Ned: --species=neurospora<br>Phs, Pug, Th: --species=generic<br>Hs, Mm: --species=human
Augustus<br>(1 gene)	2.5	--strand=both --singlestrand=false<br>--genemodel=exactlyone --codingseq=on<br>--sample=100 --keep_viterbi=true<br>--alternatives-from-sampling=false<br>--exonnames=on	Ned: --species=neurospora<br>Phs, Pug, Th: --species=generic<br>Hs, Mm: --species=human
Fgenesh	24. Jul 2007		Ned, Th: Organism: Neurospora crassa<br>Phs: Organism: Phytophtora<br>Hs: Organism: Human<br>Pug: Organism: Puccina<br>Mm: Organism: Mouse
Genscan	November,<br>2009	Organism: Vertebrate; Suboptimal exon cutoff: 1.00	
Blat	v. 34	-minScore=15	Ned, Phs: -tileSize=5 -minIdentity=54<br>-oneOff=1<br>Hs, Pug, Mm, Th: -tileSize=7<br>-minIdentity=81
Blat<br>(tilesize 6)	v. 34	-tileSize=6 -minIdentity=54<br>-minScore=15 -oneOff=1	
Blat<br>(tilesize 5)	v. 34	-tileSize=5 -minIdentity=54<br>-minScore=15 -oneOff=1	
Blast	2.2.20		

Ned: *Neurospora discreta*, Phs: *Phytophthora sojae*, Hs: *Homo sapiens*, Pug: *Puccinia graminis*, Mm: *Mus musculus*, Th: *Thielavia heterothallica*
